# Role of Vitamin D Supplementation in Chronic Liver Disease: A Systematic Review and Meta-Analysis of Randomized Controlled Trials

**DOI:** 10.1093/nutrit/nuaf117

**Published:** 2025-07-11

**Authors:** Petrana Martinekova, Mahmoud Obeidat, Mihaela Topala, Szilárd Váncsa, Dániel Sándor Veres, Ádám Zolcsák, Miheller Pál, László Földvári-Nagy, Peter Banovcin, Bálint Erőss, Péter Hegyi, Krisztina Hagymasi

**Affiliations:** Centre for Translational Medicine, Semmelweis University, Budapest 1085, Hungary; Institute for Clinical and Experimental Medicine, Prague 140 21, Czech Republic; Centre for Translational Medicine, Semmelweis University, Budapest 1085, Hungary; Centre for Translational Medicine, Semmelweis University, Budapest 1085, Hungary; Carol Davila University of Medicine and Pharmacy, Bucharest 050474, Romania; Centre for Translational Medicine, Semmelweis University, Budapest 1085, Hungary; Institute of Pancreatic Diseases, Semmelweis University, Budapest 1083, Hungary; Institute for Translational Medicine, University of Pécs, Medical School, Pécs 7623, Hungary; Centre for Translational Medicine, Semmelweis University, Budapest 1085, Hungary; Department of Biophysics and Radiation Biology, Semmelweis University, Budapest 1094, Hungary; Centre for Translational Medicine, Semmelweis University, Budapest 1085, Hungary; Department of Biophysics and Radiation Biology, Semmelweis University, Budapest 1094, Hungary; Centre for Translational Medicine, Semmelweis University, Budapest 1085, Hungary; Department of Surgery, Transplantation and Gastroenterology, Semmelweis University, Budapest 1082, Hungary; Centre for Translational Medicine, Semmelweis University, Budapest 1085, Hungary; Department of Morphology and Physiology, Faculty of Health Sciences, Semmelweis University, Budapest 1088, Hungary; Centre for Translational Medicine, Semmelweis University, Budapest 1085, Hungary; Gastroenterology Clinic, University Hospital in Martin, Jessenius Faculty of Medicine in Martin, Comenius University in Bratislava, Martin 036 59, Slovak Republic; Centre for Translational Medicine, Semmelweis University, Budapest 1085, Hungary; Institute of Pancreatic Diseases, Semmelweis University, Budapest 1083, Hungary; Institute for Translational Medicine, University of Pécs, Medical School, Pécs 7623, Hungary; Centre for Translational Medicine, Semmelweis University, Budapest 1085, Hungary; Institute of Pancreatic Diseases, Semmelweis University, Budapest 1083, Hungary; Institute for Translational Medicine, University of Pécs, Medical School, Pécs 7623, Hungary; Centre for Translational Medicine, Semmelweis University, Budapest 1085, Hungary; Department of Surgery, Transplantation and Gastroenterology, Semmelweis University, Budapest 1082, Hungary

**Keywords:** vitamin D, micronutrient, liver cirrhosis, chronic liver disease

## Abstract

**Context:**

Vitamin D deficiency is highly prevalent in chronic liver disease. Although international societies recommend vitamin D supplementation in cases of proven deficiency, the impact of vitamin D on chronic liver disease remains uncertain.

**Objective:**

Our aim was to evaluate the effects of vitamin D supplementation in patients with chronic liver disease by conducting a systematic review and meta-analysis of randomized controlled trials (RCTs).

**Data Sources:**

We systematically searched PubMed, EMBASE and the Cochrane Library on July 2, 2024.

**Data Extraction:**

Our primary outcomes involved survival, controlled attenuation parameter (CAP), liver stiffness measurement (LSM), and effects on changes in liver enzymes. Secondary outcomes included lipid profile and homeostasis model assessment of insulin resistance (HOMA-IR), among others. The pooled risk ratio (RR), mean difference (MD), and corresponding 95% CIs were calculated using the random-effects model.

**Data Analysis:**

Forty-six RCTs were included, comprising 4084 patients. When we compared the vitamin D group with the control, the RR for overall survival was 1.14 (95% CI, 0.85-1.54; 4 RCTs) at 6 months and 0.99 (95% CI, 0.83-1.17; 4 RCTs) at the 12-month follow-up. Vitamin D supplementation did not result in a lower CAP (MD, −23.50 dB/m; 95% CI, −81.72 to 34.72; 3 RCTs) and LSM (MD, −0.65 kPa; 95% CI, −1.98 to 0.68; 3 RCTs). A significant reduction in HOMA-IR was observed in the vitamin D group (MD, −0.31; 95% CI, −0.62 to −0.01; 15 RCTs). Alanine aminotransferase (ALT) (MD, −4.98 IU/L; 95% CI, −8.28 to −1.68; 24 RCTs), aspartate aminotransferase (AST) (MD, −3.33 IU/L; 95% CI, −6.25 to −0.40; 23 RCTs), gamma-glutamyl transferase (GGT) (MD, −5.14 IU/L; −6.40; −3.88; 11 RCTs), triglycerides (MD, −7.59 mg/dL; 95% CI, −15.09 to −0.81), and insulin (MD −0.79 μIU/L; 95% CI, −1.36 to −0.21) were significantly reduced in the patients with vitamin D supplementation.

**Conclusion:**

Our results showed significantly reduced ALT, AST, GGT, triglycerides, insulin, and HOMA-IR in the vitamin D–supplemented group; however, the effect was modest. In addition, there were no differences in survival, CAP, or LSM. Further RCTs with adequate power are warranted to clarify the results.

**Systematic Review Registration:**

PROSPERO registration No. CRD42022370312

KEY POINTS

1. Basic science suggested a possible protective role of vitamin D in hepatic fibrogenesis. The beneficial effect of vitamin D supplementation on liver steatosis and fibrosis was not shown based on the result of our study but further research is warranted.
2. Insulin resistance, determined by HOMA-IR, was significantly reduced after vitamin D supplementation. While insulin levels were reduced in vitamin D patients, there was no evidence of a difference in fasting plasma glucose levels.
3. There was a significant reduction in ALT, AST, and GGT in vitamin D-supplemented patients. The inclusion of eligible participants with liver enzymes above the normal range may ascertain the effect.

## INTRODUCTION

The burden of chronic liver disease, which leads to significant mortality rates, is a global concern. Ye et al demonstrated an increasing trend in global cirrhosis-related mortality of 47.15% from 1990 to 2017.^1^ The most pronounced increase was detected in countries with a middle or high sociodemographic index and Eastern Europe, where unfavorable trends in cirrhosis-related mortality are driven by lifestyle-modifiable etiologies such as alcohol-related liver disease (ALD) or non-alcoholic fatty liver disease (NAFLD).

The nuclear vitamin D receptor (VDR) has been detected in various tissues and cells, involving skeletal muscle, liver, pancreas, or immune cells.^2^ The circulating form of vitamin D, calcifediol (25-hydroxycholecalciferol, 25OHD), can be used by many cells to locally produce its active metabolite owing to the presence of a 1α-hydroxylase enzyme (CYP27B1).^3^ Due to the potential of vitamin D to regulate the expression of numerous genes, intensive research on its extra-skeletal properties has been conducted over the last 2 decades.

Vitamin D deficiency is common in chronic liver disease, despite its endogenous cutaneous production.^4^ There are multifactorial causes, including patient lifestyle (sedentarism, lack of optimal nutritional intake) and the effect of disease (intestinal edema in portal hypertension, hepatic dysfunction, maldigestion, malabsorption, hypercatabolic state).^5^^,^^6^ The association of vitamin D deficiency with liver dysfunction, complications, and mortality, including its role in hepatic fibrogenesis, has been previously described.^7–11^

The European Association for the Study of Liver (EASL), the American Association for the Study of Liver Diseases (AASLD), and the European Society for Clinical Nutrition and Metabolism (ESPEN) recommend vitamin D supplementation in patients with chronic liver disease when a deficiency is detected in order to reach the desired levels of 25-hydroxyvitamin D (above 30 ng/mL or 75 nmol/L ).^12–14^ Nonetheless, it remains unclear whether vitamin D supplementation in deficient chronic liver disease patients yields any impact, as demonstrated by previously published meta-analysis.^15^ However, additional randomized controlled trials (RCTs) have been published. Furthermore, a quantitative summary of the effect on liver steatosis or fibrosis has not been meta-analyzed beforehand. On the basis of the recommendations of international societies and the inconsistent results of vitamin D supplementation in this population, we aimed to assess and clarify the role of vitamin D in managing chronic liver disease.

## METHODS

This study was performed in accordance with the Preferred Reporting Items for Systematic Reviews and Meta-Analyses (PRISMA) 2020 guideline (Supplementary Table S1) and the Cochrane Handbook.^16^^,^^17^ The pre-established protocol was registered in advance on PROSPERO (CRD42022370312), and we adhered to it.^18^

### Eligibility Criteria

We followed the subsequent inclusion criteria: (1) human studies; (2) randomized controlled trials; and (3) fitting the PICO framework (Table 1).^19^ To increase comprehensiveness and decrease publication bias, we also included conference abstracts, as recommended by Scherer et al.^20^

**Table 1.** Detailed PICO Framework

P	Adult patients (≥18 years) with chronic liver disease, irrespective of stage and etiology
I	Standard of care + vitamin D supplementation in any form, dose, duration, route of administration, administered as monotherapy or in combination with calcium
C	Standard of care with or without placebo
O	***Primary:*** Survival, CAP, LSM, ALT, AST, GGT, ALP, CRP, IL-6 ***Secondary:*** HOMA-IR, insulin, FPG, TC, TG, LDL, HDL, adiponectin, INR, albumin, and bilirubin ***Surrogate:*** virologic response, BMD, and sarcopenia
Abbreviations: ALP, alkaline phosphatase; ALT, alanine aminotransferase; AST, aspartate aminotransferase; BMD, bone mineral density; CAP, controlled attenuation parameter; CRP, C-reactive protein; FPG, fasting plasma glucose; GGT, gamma-glutamyl transferase; HDL, high-density lipoprotein cholesterol; HOMA-IR, homeostatic model assessment for insulin resistance; IL-6, interleukin-6; INR, international normalized ratio; LDL, low-density lipoprotein cholesterol; LSM, liver stiffness measurement; TC, total cholesterol; TG, total triglycerides.	

### Information Sources and Search Strategy

A literature search was conducted on the November 8, 2022, in 3 electronic databases: MEDLINE (via PubMed), Embase, and Cochrane Central Register of Controlled Trials (CENTRAL), with no restrictions or filtering options. Forward and backward citation chasing of included articles was performed on January 5, 2023, to identify further relevant studies. In addition, the updated search was run on February 7, 2024, to reveal newly published articles. For a detailed search strategy, see Table S2.

### Selection and Data Collection Process

After automatic and manual duplicate removal, 2 independent review authors (P.M. and M.T.) conducted a selection to identify studies potentially eligible for further assessment. Cohen’s kappa was used to describe the agreement during the selection. A third independent reviewer (M.O.) resolved disagreements in case of any discrepancy. Data from eligible studies were manually extracted and cross-checked by 2 independent investigators (P.M. and M.T.). Differences in extracted data were resolved by discussion.

### Data Items

We collected the following data items: study characteristics (first author, year, geographical location, number of participating centers, study period, and sample size); demographics (etiology of chronic liver disease, age, sex, and body mass index [BMI]); details about the intervention and control groups separately (number of participants, age, sex, BMI, baseline vitamin D level, and the standard of care applied); intervention specifics (form of vitamin D supplements, dosage, brand, duration, monotherapy or in combination with calcium); and outcome measures as reported in each article. Primary and secondary outcomes were assessed at the end of the trial period, and the change (after treatment baseline values) was extracted if data for change were available.

### Study Risk of Bias Assessment

Two authors (P.M. and M.T.) performed the risk of bias assessment independently using the Cochrane Collaboration RoB2 tool with disagreements resolved by consensus.^21^ The subsequent domains were critically evaluated: randomization process, deviation from the intended interventions, missing outcomes, outcome measurement, and selection of reported results. Each domain and each outcome were judged to have a high (red), unclear (yellow), or low (green) risk of bias.

### Quality of Evidence

Each finding was evaluated according to the Grading of Recommendations, Assessment, Development, and Evaluations (GRADE) framework using the GRADEpro tool (software) to estimate the level of evidence.^22^

### Synthesis Method

The minimum number of studies required to perform a meta-analysis was set at 3. As we assumed considerable between-study heterogeneity in all cases, a random-effects model was used to pool effect sizes. When there were 2 treatment or control arms, we combined them to create a single pairwise comparison, as recommended by the Cochrane Handbook.^17^ We calculated the risk ratio (RR) with a 95% CI for dichotomous variables and mean differences (MDs) for continuous variables. Results were considered statistically significant if the pooled CIs did not contain the null or 1 value for MDs and RRs, respectively. We summarized the findings of the meta-analysis in forest plots. In addition to the between-study variance (τ^2^), between-study heterogeneity was described by Higgins and Thompson’s *I*^2^ statistics.^23^ Further details on the statistical analysis can be found in Supplementary File S1, synthesis methods and search key.

## RESULTS

### Search and Selection

**Figure 1.**
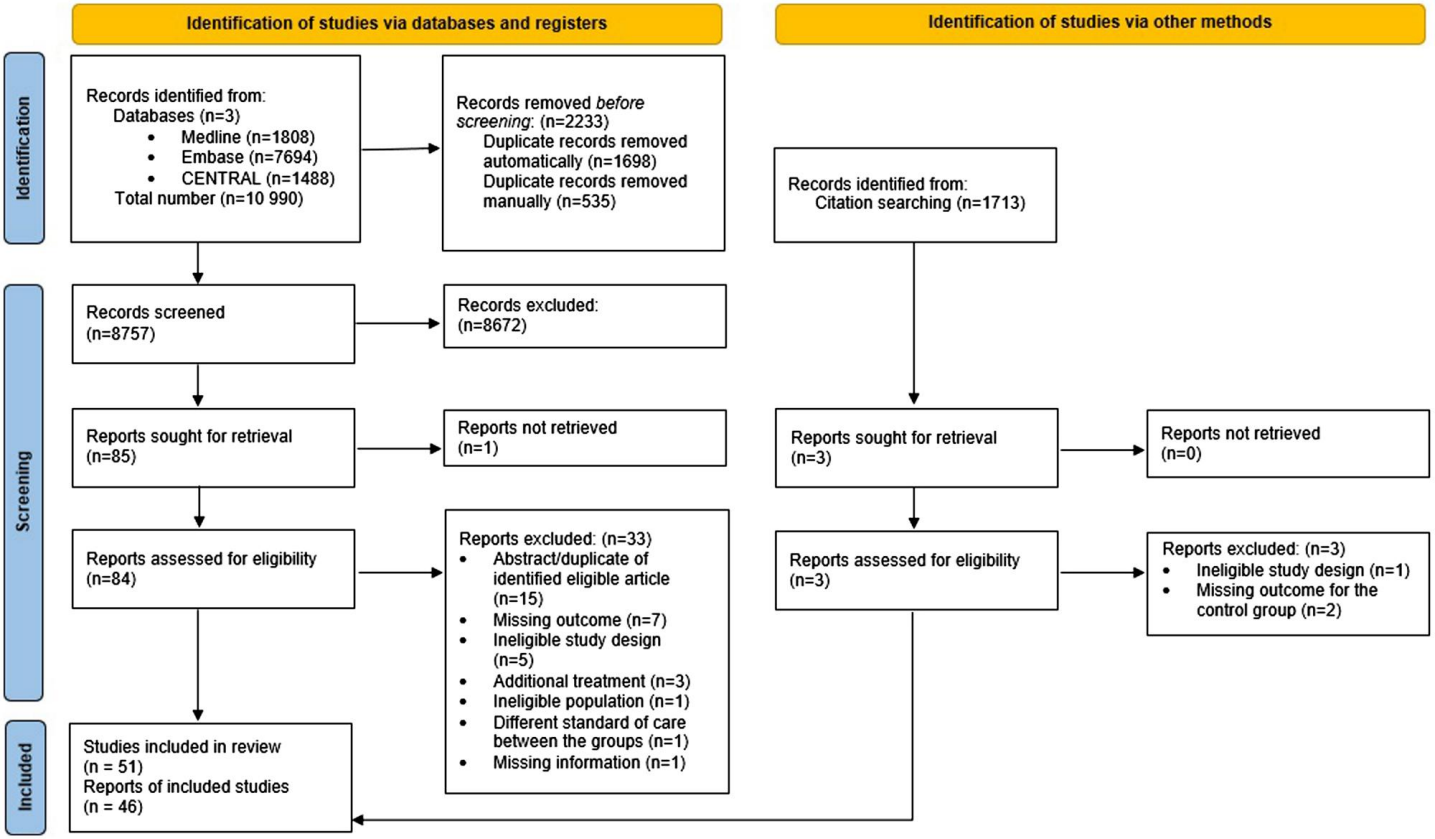
PRISMA Flowchart of the Article Selection Process

Altogether, 10 990 studies were identified. After duplicate removal, 8757 articles remained; titles and abstracts were reviewed for these. Eighty-five studies were found eligible, 51 of them were finally selected (Figure 1). Three RCTs were published only as abstracts,^24–26^ and 1 abstract was a post hoc analysis.^27^^,^^28^

### Baseline Characteristics of Included Studies

Supplementary Table S3 yields baseline characteristics of relevant studies. Thirty-six of the included studies were performed in Asia,^24^^,^^26–65^ 5 in Europe,^25^^,^^66–69^ 4 in Africa,^70–73^ and 1 in the USA.^74^ The most used form of vitamin D supplementation was vitamin D3 (cholecalciferol, *n* = 33), followed by calcitriol (*n* = 7), vitamin D2 (ergocalciferol, *n* = 6), and calcifediol (*n* = 1).

In 20 RCTs patients suffered from NAFLD, 13 RCTs involved patients with chronic viral hepatitis C (CHC), 3 trials were performed on patients with chronic hepatitis B (CHB), and 1 trial included both CHC and CHB patients. Six studies involved patients with liver cirrhosis regardless of etiology, 1 was conducted on patients with alcohol-related liver cirrhosis,^74^ 1 involved liver transplant recipients,^62^ and 1 included only patients with primary biliary cholangitis.^55^ Of 46 included studies, 23 included only patients with proven vitamin D insufficiency/deficiency (<30 ng/mL), as depicted in Table S4.

### Survival

**Figure 2.**
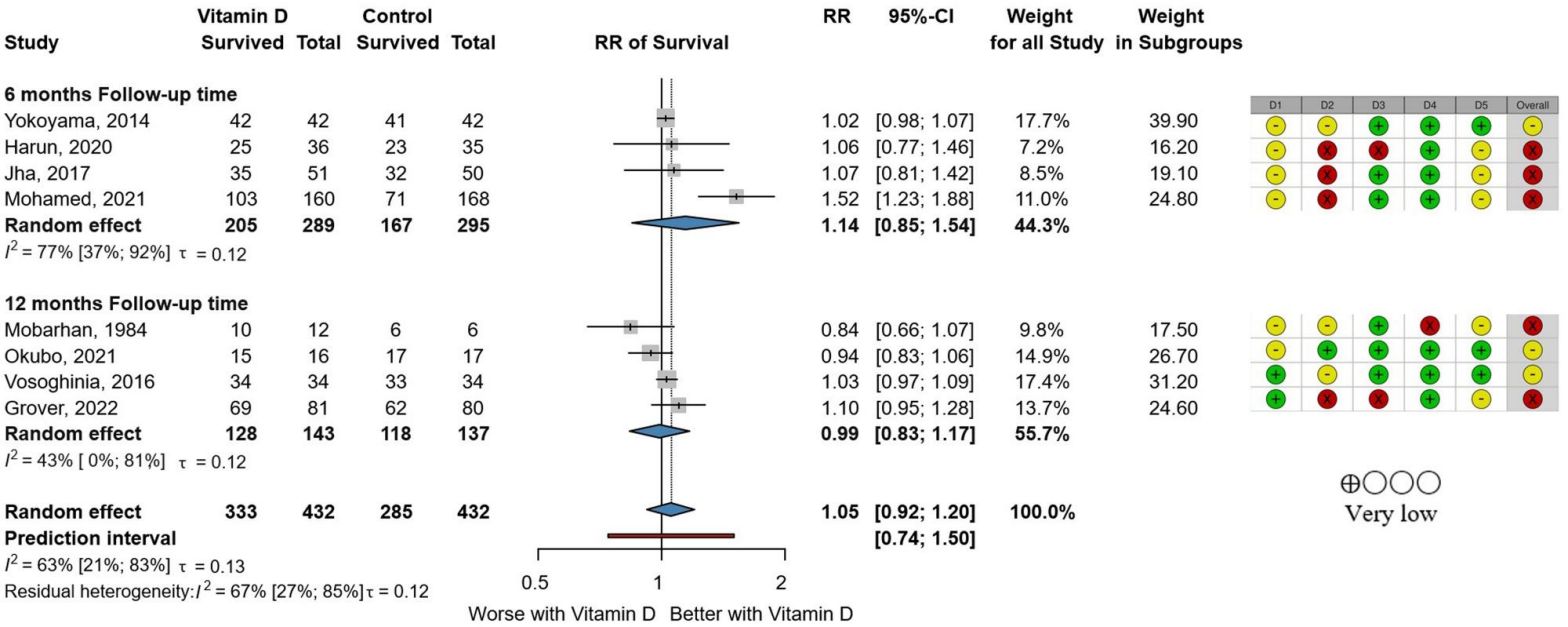
Forest Plot Showing Survival in Vitamin D and Control Groups at 6- and 12-Month Follow-Up. Abbreviation: RR, risk ratio.

Eight RCTs were included in the survival analysis divided into 6- and 12-month periods.^24^^,^^27^^,^^44^^,^^52^^,^^60^^,^^65^^,^^73^^,^^74^ We found no evidence for improved or worse survival in vitamin D-supplemented patients with RR 1.14 (95% CI, 0.85-1.54) and 0.99 (95% CI, 0.83-1.17) at the 6- and 12-month follow-ups, respectively (Figure 2). Additionally, we did not find differences between the chronic hepatitis and cirrhosis groups (Figure S5.26).

### Liver Steatosis (CAP) and Liver Fibrosis (LSM)

**Figure 3.**
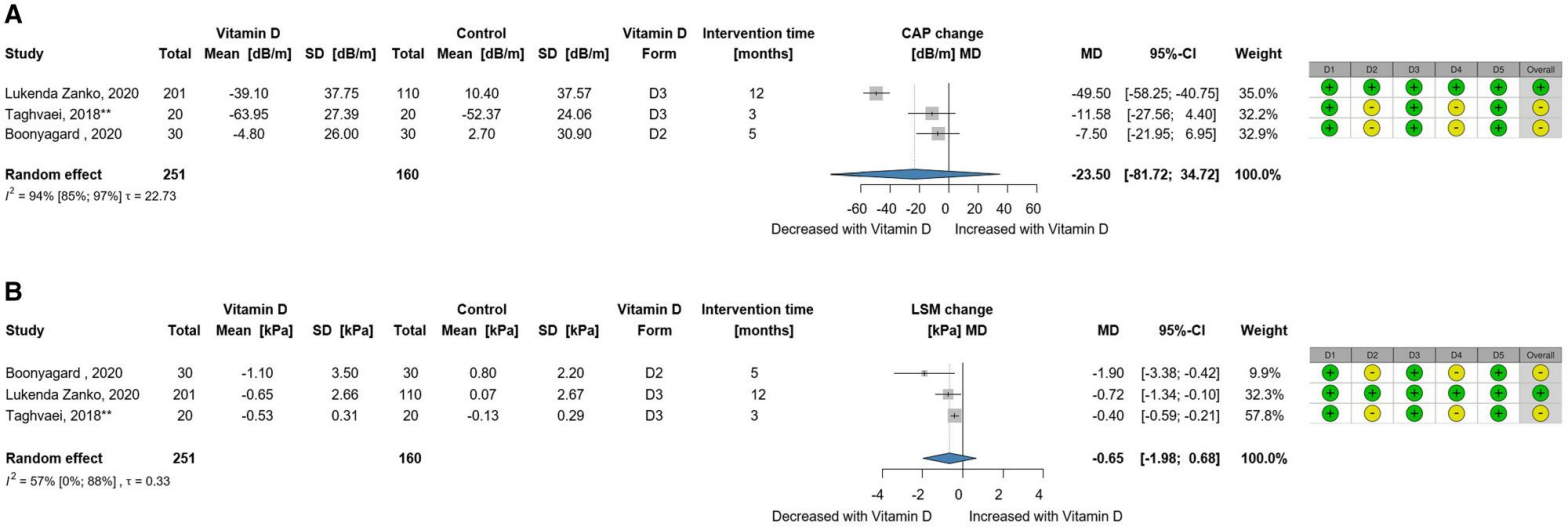
(A) Forest Plot Showing the Change in the Controlled Attenuated Parameter (CAP) Representing Liver Steatosis and (B) Forest Plot Showing the Change in the Liver Stiffness Measurement (LSM) Representing Liver Fibrosis. Abbreviations: MD, mean difference. If the study is indicated with **, then the change value is an estimated change value of that study. β means that the mean and SD are the estimated mean and SD in that study. See Supplementary Material for raw data and synthesis methods

Three RCTs with 411 participants with NAFLD were included in this analysis. Patients receiving vitamin D supplementation showed no significant effects on steatosis assessement by controlled attenuation parameter (CAP) (MD, −23.50 dB/m; 95% CI, −81.72 to 34.72) or fibrosis assessed by liver stiffness measurement (LSM) (MD, −0.65 kPa; 95% CI, −1.98 to 0.68); however, a small sample size may impact these findings (Figure 3A and 3B). A summary of additional findings on liver fibrosis and steatosis is presented in Table 2.

**Table 2.** Summary of Further Findings on Liver Fibrosis and Steatosis

Author and year	Findings
Afsordeh et al 2019^30^	8 wk of aerobic training statistically improved grade of fatty liver in both the AT+vitamin D group and AT group; meanwhile, vitamin D supplementation of 50 000 IU/wk alone did not improve the grade.
Alarfaj et al 2023^70^	No significant differences in the degree of hepatic steatosis were observed between the vitamin D–supplemented and placebo arms.
Barchetta et al 2016^66^	Cholecalciferol for 24 wk did not result in significant changes between the HFF, P3NP, and FLI groups.
Boonyagard et al 2020^33^	No significant differences observed in steatosis grade and fibrosis stage assessed by Fibroscan^®^ after 5 mo of D2 supplementation.
Ebrahimpour-Koujan et al 2024^35^	After 12 wk of intervention, serum hyaluronic acid (−28.7 ng/mL in vitamin D–supplemented vs −3.5 ng/mL in placebo, *P* = .04) and laminin (−10.6 ng/mL in vitamin D–supplemented and 4.9 ng/mL in placebo, *P* = .01) significantly differed between the groups, while there was no difference in collagen type IV levels (*P* = .09).
Geier et al 2018^67^	Disease activity evaluated by NAFLD activity score improved in 3/3 vitamin D and 3/4 placebo patients. Absolute reduction of steatosis by at least 20% was observed in 2/3 vitamin D patients but in none of the placebo patients.
Hoseini et al 2020^40^	Although the grade of fatty liver was reduced in AT+VD, AT, and vitamin D groups, the most pronounced reduction was observed when aerobic training was combined with vitamin D supplementation. Moreover, the grade of fatty liver significantly increased in the control group.
Hosseini et al 2018^41^	Single intramuscular dose of cholecalciferol + vitamin E 400 IU/d reduced the severity of liver steatosis by 1 grade in 8/37 (21.6%). The same standard of care + vitamin E 400 IU without vitamin D caused a grade reduction only in 1/38 (2.6%).
Komolmit et al 2017^47^	6 wk of D2 supplementation significantly increased antifibrogenic MMP-2 and MMP-9 levels while reducing profibrogenic TGF-β.
Pilz et al 2016^69^	Treatment with 2800 IU/d of cholecalciferol for 8 wk showed no difference in ELF score and levels of hyaluronic acid between the groups.
Sharifi et al 2014^56^	No significant differences in grade of hepatic steatosis observed between the vitamin D–supplemented and placebo arms.
Sriphoosanaphan et al 2021^58^	6 wk of vitamin D in CHC patients after curative treatment with DAAs does not improve serum fibrogenesis markers (MMP-9, P3NP, TGF-β) and may not facilitate the healing process of the remaining hepatic fibrosis.
Yaghooti et al 2021^64^	Treatment with calcitriol 0.25 μg/d for 17 wk did not result in better HSI or APRI when compared to the placebo group.

Abbreviations: APRI, AST-to-Platelet Ratio Index; AT, aerobic training; CHC, chronic hepatitis C; DAA, direct antiviral agent; D2, ergocalciferol; ELF, enhanced liver fibrosis; FLI, fatty liver index; HFF, hepatic fat fraction; HSI, hepatic steatosis index; IU, international units; MMP, matrix metalloproteinase; P3NP, procollagen 3 N-terminal peptide; TGF-β, tumor growth factor β.

### Liver Enzymes

**Figure 4.**
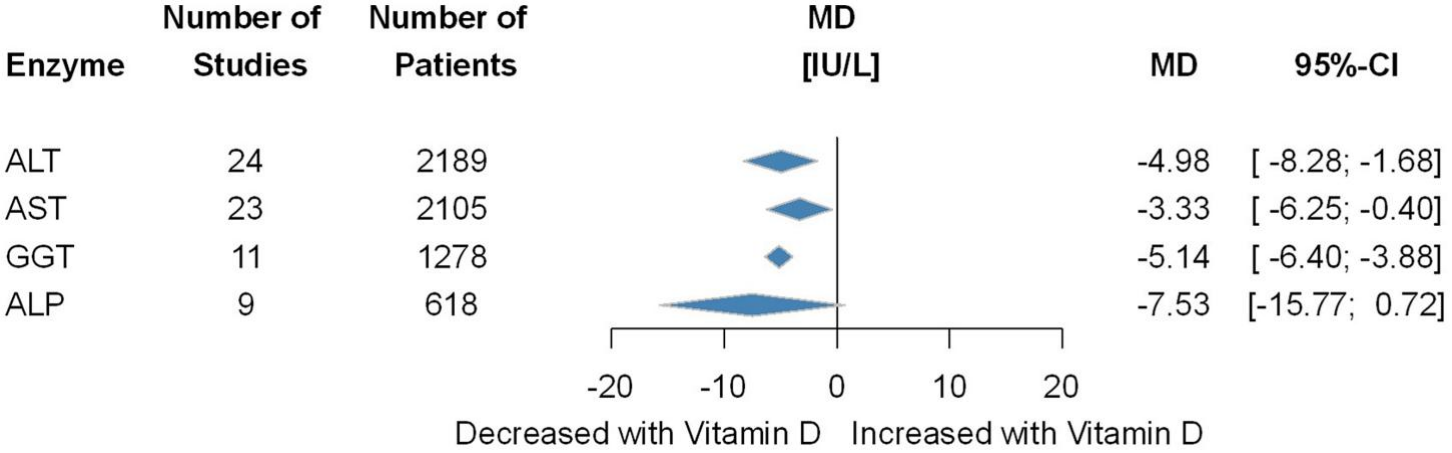
Summary Forest Plot Showing Changes in Alanine Aminotransferase (ALT), Aspartate Aminotransferase (AST), Gamma-Glutamyl Transferase (GGT) and Alkaline Phosphatase (ALP) in Vitamin D and Control Groups. MD, mean difference

The effect of vitamin D supplementation on liver enzymes is illustrated in Figure 4. In the vitamin D group, a significant decrease in ALT was observed with a small change of MD, −4.98 IU/L (95% CI, −8.28 to −1.68). A similar result was found for AST (MD, −3.33; 95% CI, −6.25 to −0.40) and gamma-glutamyl transferase (GGT) (MD, −5.14 IU/L; 95% CI, −6.40 to −3.88). The results for ALP were marginally significant (MD, −7.53; 95% CI, −15.77 to 0.72). No subgroup differences were observed if studies included only vitamin D–deficient patients (*P* = .46 for ALT, *P* = .14 for AST, *P* = .69 for GGT), however, in patients who underwent vitamin D-supplementation for ≥3 months showed a more pronounced decrease in the vitamin D-supplemented group for ALT (*P* = .02) and AST (*P* = .04) but not for ALP (*P* = .83). The results remained significant when excluding high-risk biased studies in cases of AST, ALT, and GGT. Further details regarding subgroups and individual analyses can be found in Supplementary file S2.

### Glucose and Lipid Metabolism

**Figure 5.**
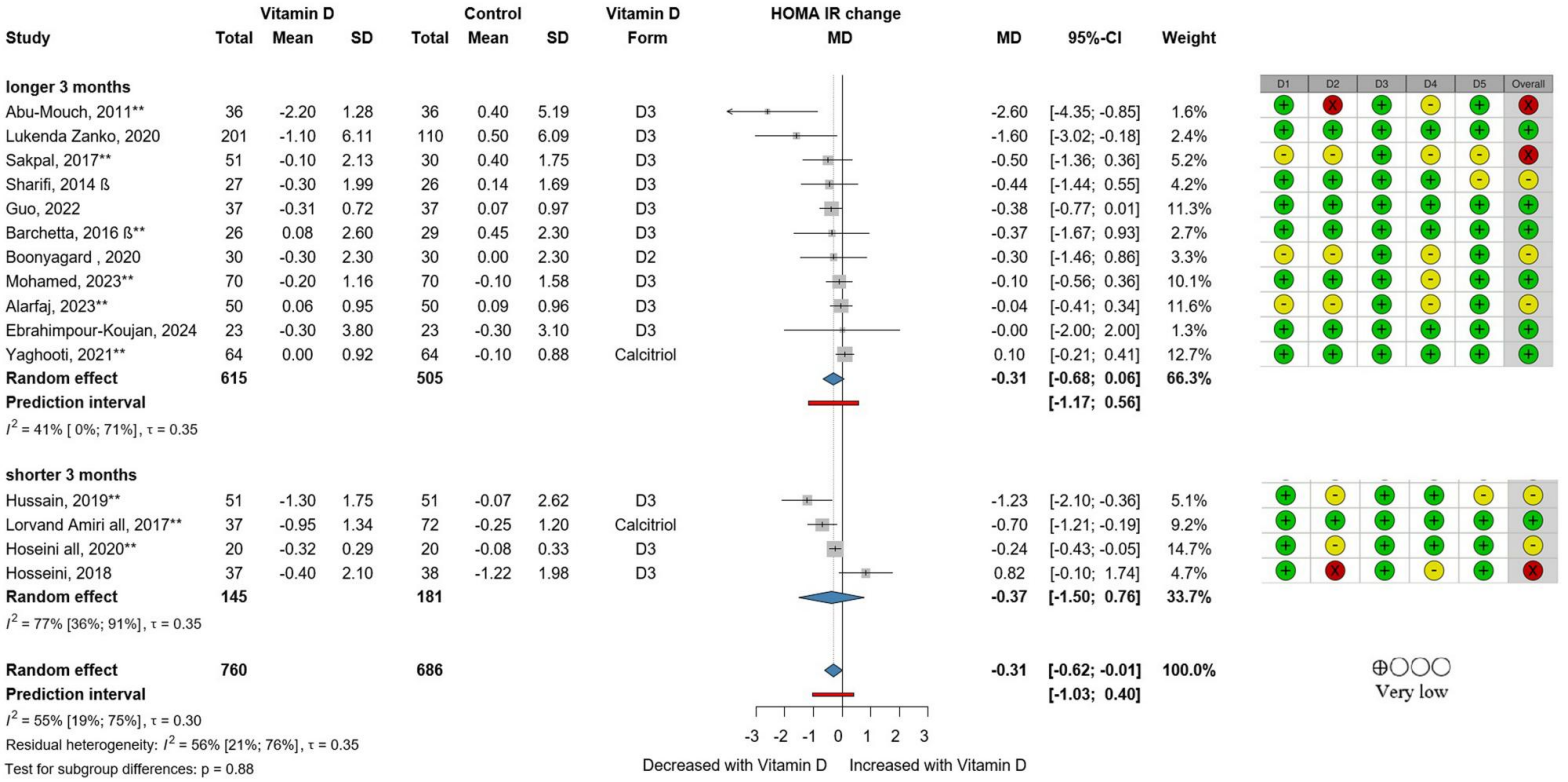
Forest Plot Showing HOMA-IR Change in Vitamin D and Control Groups by Length of Intervention. Abbreviations: MD, mean difference. If the study is indicated with **, then the change value is an estimated change value in that study. The β means that the mean and SD are the estimated mean and SD in that study. See the raw data and the synthesis methods in the Supplementary Material.

Fifteen RCTs with 1426 participants investigated the effect of vitamin D on HOMA-IR. All but 1 study^29^ involved only patients with NAFLD. Vitamin D–supplemented patients showed decreased HOMA-IR (MD, −0.31; 95% CI, −0.62 to −0.01), indicating an improvement (Figure 5). The length of the vitamin D supplementation (≥ or <3 months) did not result in differences between the subgroups (*P* = .88). Additionally, no difference was observed with subgrouping for the presence of vitamin D insufficiency/deficiency or sufficiency (*P* = .47), as visualized in Figure S3.3.

Fasting insulin levels decreased modestly in vitamin D–supplemented patients (MD, −0.79 μIU/L; 95% CI, −1.36 to −0.21), whereas FPG did not vary significantly between the groups. No difference was observed in adiponectin, low density lipoprotein (LDL), and TC. Meanwhile, modestly increased high-density lipoprotein (HDL) (MD, 1.65 mg/dL; 95% CI, 0.08 to 3.23) and lowered triglyceride (TG; MD −7.59 mg/dL; 95% CI, −15.09 to −0.81) were found in the vitamin D–supplemented group (Supplementary File S4). The result remained statistically significant for TG when excluding high-risk biased studies.

### Additional Outcomes

#### Inflammatory Markers.

We did not observe a difference in C-reactive protein (CRP) with an MD change of −0.77 mg/L (95% CI, −1.80 to 0.26). In addition, there was no significant difference in interleukin-6 (IL-6) between the groups with an MD change of −1.42 pg/mL (95% CI, −4.51 to 1.69) (Supplementary File S5). The study by Yang et al^63^ was excluded from the analyses for CRP (estimated change value, −40.22 mg/dL; 95% CI, −42.19 to −38.25) and IL-6 (estimated change value −76.80; 95% CI, −86.79 to −66.81) as they differed in magnitude, which suggested that not only the mean but the individual data of the patients probably highly differed.

#### Bone Mineral Density and Sarcopenia.

Due to insufficient data and differences in reporting bone mineral density (BMD), we were unable to meta-analyze this outcome. Five RCTs evaluated the effect of vitamin D supplementation on BMD, and 2 studies investigated the effect on skeletal muscles (Supplementary File S5).

#### Virologic Response.

The meta-analysis showed no statistical significance for sustained virologic response in vitamin D–supplemented patients (RR, 1.29; 95% CI, 0.96-1.74) (Supplementary file S6). Moreover, Wang et al^61^ detected no differences in quantitative hepatitis B (HB) antigen or hepatitis B virus (HBV) DNA levels in patients in the vitamin D and control groups.

#### International normalized ratio, Albumin, Bilirubin.

No significant differences were observed for the international normalized ratio (INR) (MD, −0.08; 95% CI, −0.59 to 0.43), albumin (MD, 0.01; 95% CI, −0.11 to 0.13 g/dL), and bilirubin (MD, −0.18; 95% CI, −0.62 to 0.26 mg/dL) (Supplementary File S5).

#### Adverse Events.

Details of adverse events are provided in Table S6.

### Subgroup Analyses

When at least 3 studies for each group were present, we performed additional subgroup analyses based on the length of the intervention (<3 or ≥3 months) and the presence of vitamin D insufficiency/deficiency or sufficiency (<30 or ≥30 ng/mL). No analyses were possible for the form of vitamin D and the etiology of chronic liver disease due to a paucity of data. Subgroup analyses demonstrated that longer interventions led to significant differences in ALT and AST (*P* = .02 and .04, respectively). However, such differences were not found (*P* > .05) for ALP, HOMA-IR, FPG, TC, LDL, HDL, and TG.

Surprisingly, subgrouping based on the presence of vitamin D insufficiency/deficiency (<30 ng/mL) did not yield significant results (*P* > .05) for ALT, AST, GGT, HOMA-IR, insulin, FPG, TC, LDL, HDL, TG, and CRP. Excluding high-risk bias studies from the analyses did not have an impact on the findings for any outcome except HDL. Detailed subgroup analysis figures are available in Supplementary Files S2-S5.

### Risk of Bias and Certainty of Evidence

Most studies resulted in an overall high risk of bias (*n* = 21), followed by moderate (*n* = 13) and low (*n* = 12) risk. The high risk of bias was mainly due to deviations from the intended interventions as a result of inadequate blinding (Supplementary File S7). However, the overall risk of bias differed among the outcomes accounting for results from low-moderate in the case of glucose and lipid metabolism to high in the case of survival. The quality of evidence was very low based on the GRADE assessment (Table S7).

### Publication Bias and Heterogeneity

No publication bias was detected for ALT, GGT, HOMA-IR, FPG, insulin, LDL, and TG. Egger’s tests suggested a potential publication bias for AST (*P* = .0946), HDL (*P* = .0963), and TC (*P* = .0291) (Supplementary File S2-S6). The visual inspection of funnel plots did not confirm potential publication bias but rather indicated high heterogeneity; therefore, we believe that publication bias is unlikely, but even if present, it is not relevant based on the data.

## DISCUSSION

We comprehensively assessed the role of vitamin D in chronic liver disease. Current evidence showed no statistical difference between vitamin D supplementation and the standard of care in terms of liver steatosis and fibrosis. Clinically negligible but statistically significant reductions in ALT, AST, GGT, TG, insulin, and HOMA-IR were observed in the vitamin D–supplemented group. On the contrary, we did not find sufficient evidence that supplementation affects survival, CRP, IL-6, LDL, TC, adiponectin, FPG, or liver function tests (INR, albumin, bilirubin).

Vitamin D deficiency, especially severe vitamin D deficiency, has been associated with increased mortality in patients with chronic liver disease.^75^^,^^76^ Furthermore, a recent meta-analysis by Yang et al confirmed the association between severe vitamin D deficiency (25OHD < 10 ng/mL) and mortality risk in patients with liver cirrhosis (RR, 1.79; 95% CI, 1.44-2.22).^77^ Mayr et al reported that cirrhotic ICU patients with vitamin D levels >20 ng/mL at admission had better survival than those with moderate or severe vitamin D deficiency.^78^ Nonetheless, our study failed to demonstrate prolonged survival in the vitamin D–supplemented group for either 6 or 12 months of follow-up. Half of the studies involved only patients with at least moderate vitamin D deficiency (<20 ng/mL).^24^^,^^44^^,^^52^^,^^74^ The severity of chronic liver disease could modify the effect of vitamin D supplementation on survival; however, we did not find differences between the chronic hepatitis and cirrhosis patient groups. Six of 8 RCTs included patients with liver cirrhosis. While 4 of these patients reported being in the decompensated stage of liver cirrhosis,^24^^,^^44^^,^^52^^,^^73^ 1 study was performed on patients with liver cirrhosis irrespective of the compensation,^27^ and no details were given in another report.^74^ Compensation for liver disease is the crucial factor regarding mortality, as the 28-day mortality in decompensated cirrhosis can reach from 5% up to 20% depending on the presence of acute or chronic liver failure.^79^ Our analysis recognized the study of Mohamed et al^73^ in which the vitamin D–supplemented group showed better survival. Interestingly, only patients with decompensated liver cirrhosis and spontaneous bacterial peritonitis (SBP) were included in this trial. Yang et al also observed a lower incidence of SBP in vitamin D–supplemented patients (5.8% vs 30%, *P* < .01).^63^ Further research may help to explain the role of vitamin D in immunity and ascertain its effect on decompensated cirrhotic patients with SBP. Cathelicidin is recognized as 1 of the factors responsible for the antimicrobial effects of vitamin D.^80^ As infections contribute to the high mortality observed in liver diseases, further translational insight into the vitamin D–cathelicidin axis may benefit the current understanding.

Emerging evidence suggests a protective effect of vitamin D against liver fibrogenesis. Although the mechanisms of action on hepatic fibrogenesis remain uncertain, vitamin D has shown antifibrotic properties based on its regulation of hepatic stellate cells (HSCs) via the VDR. Quiescent HSCs are activated upon stimulation by growth factors and cytokines, consequently playing an important profibrogenic role.^11^^,^^81^ Komolmit et al detected reduced levels of profibrotic TGF-β, while some antifibrotic metalloproteinases were significantly increased compared with those in the placebo arm.^47^ On the other hand, neither 6 weeks nor 4 months of vitamin D supplementation resulted in statistical differences between the groups in the remaining RCTs.^56^^,^^58^ Vitamin D has been shown to alleviate hepatic fibrosis in cholecalciferol-treated mice through its effect on histidine-rich calcium-binding protein.^82^ A study by Ding et al demonstrated that VDR-knockout mice develop spontaneous liver fibrosis.^83^ In addition, it has been shown that a noncalcemic vitamin D derivate, calcipotriol, could prevent thioacetamide-induced liver cirrhosis in a mouse model.^84^ Our meta-analysis did not demonstrate a reduction in steatosis or fibrosis measured non-invasively by CAP and LSM in NAFLD. The current EASL guideline recommends liver biopsy as a reference to evaluate nonalcoholic steatohepatitis and liver fibrosis resolution; therefore, these findings must be interpreted with caution.^85^ Given the small sample size and the tendency observed, additional RCTs should be performed. In addition, the length of vitamin D supplementation and adequate time for the outcome assessment must be considered, especially for outcomes such as liver steatosis or fibrosis. Lukenda Zanko et al observed the superiority of vitamin D supplementation in the improvement of CAP and LSM, which gradually decreased over the 6-month to 12-month period in the vitamin D–supplemented group but worsened in the placebo.^68^ Fibrosis progression and resolution develop over months, years, or even decades, suggesting longer intervention and follow-up time may be required to elicit the effect of vitamin D on fibrogenesis.^86^

Insulin resistance is an important pathophysiological factor contributing to the development and progression of NAFLD. In addition, insulin resistance has been found to influence the course of disease in patients with CHC.^87^ Our meta-analysis of 15 RCTs with 1426 participants showed a modest effect of vitamin D on insulin resistance measured by HOMA-IR. This finding is concurrent with a previously published meta-analysis, in which vitamin D improved insulin resistance with the pooled MD −1.06 (95% CI, −1.66 to −0.45; *P* = .0006).^88^ Interestingly, a double-blind trial of 54 participants with NAFLD and concomitant vitamin D deficiency/insufficiency compared the effect of calcitriol (1 μg/d) and cholecalciferol (50 000 IU/wk) and found that calcitriol reduced HOMA-IR 1.8 times more than cholecalciferol.^89^ Due to data limitations, we were unable to compare different forms of vitamin D. Similarly, the present data indicate that insulin levels were significantly reduced in the supplemented group. Although the dogma implies that hyperinsulinemia is a secondary consequence of insulin resistance in the past, current evidence has questioned this belief.^90^^,^^91^ Nevertheless, we found no difference in adiponectin levels, another contributing factor of insulin resistance described in the literature. Meta-analysis of RCTs conducted on adults with diabetes mellitus type 2 and an umbrella review of meta-analysis in adults investigated the effect of vitamin D on lipid metabolism, for which the same improvement of HDL and reduction of TG was found.^92^^,^^93^

In populations at risk for vitamin D deficiency, recommended doses vary widely. The majority of the included RCTs used cholecalciferol as the preferred form, although higher absorption rates of calcifediol have been described.^94^ Recent guidelines recommend against ergocalciferol supplementation, which was chosen in 5 of the studies included in our analysis.^95^ Indeed, a tailored approach to vitamin D supplementation is needed, based on the specific mechanisms underlying the deficiency.^96^ Pludowski et al recommended using calcifediol in patients with chronic liver disease.^96^ However, data on the effects of calcifediol are scarce, as this study shows that only 1 RCT used solely this form. Surprisingly, it has been proposed that the oral route of administration may be preferable for vitamin D deficiency in the loading phase, whereas both oral and parenteral routes may be equivalent in maintenance treatment.^97^ This finding is contradicted by the conclusions of 2 other studies, in which the intramuscular route was more effective in restoring vitamin D levels.^98^^,^^99^ It remains unanswered how much these findings reflect the needs of chronic liver disease.

### Strengths and Limitations

Several strengths of the present study must be acknowledged. A rigorous methodology was strictly applied at each step. We included only RCTs to reduce confounding and increase the level of evidence. In addition, we performed a subgroup analysis for the length of the intervention and etiological factors, whereas subgrouping by a form of vitamin D was not possible due to the low number of studies in different groups. In comparison with the previous meta-analysis,^15^ we were able to include 19 additional trials with 2105 more participants and we assessed the effect of vitamin D supplementation on liver steatosis and fibrosis, while similar eligibility criteria were applied. Moreover, in this article we present dynamic changes in multiple outcomes rather than simple comparisons between the groups and we performed additional subgroup analyses.

On the other hand, limitations should also be considered. The results are based on the limited number of trials performed mainly in Asia (78%); therefore, the conclusions might not be extrapolatable to the general population. Furthermore, differences in the applied interventions in terms of duration, doses, and follow-up also contributed to the high heterogeneity, which was not reduced when subgrouping for the length of the intervention or vitamin D insufficiency/deficiency. However, a limited number of studies for each tested association may weaken the conclusions and limit the feasibility of meaningful subgroup analyses. Although patients with ALD or cholestatic liver diseases have significantly lower vitamin D serum concentrations than individuals with other causes of liver cirrhosis,^75^ only 2 studies included solely this specific population.

### Implications for Practice and Research

Rapid application of scientific results is paramount.^100^^,^^101^ As the burden of liver diseases indicates progressive trends in lifestyle-modifiable diseases and liver disorders steadily contribute to premature deaths, it is necessary to translate mechanistic knowledge into bedside care. Vitamin D supplements are recommended to patients by individual societies; therefore, this meta-analysis provides a bridge to understanding the role of vitamin D in the selected population. Vitamin D may be a cost-effective and simple tool to modestly improve some outcomes in patients with chronic liver disease. The present work showed a more pronounced decrease in liver enzymes of vitamin D–supplemented patients. On the other hand, it should be noted that the magnitude of liver enzyme elevation does not necessarily reflect the severity of liver disease and prognosis, especially in chronic liver disease.^102^ Even though nutritional interventions improve health outcomes, the possible role of a single micronutrient seems to be small. Further RCTs with adequate power and appropriate sample size are warranted to clarify the topic, particularly to evaluate the effects of prolonged vitamin D supplementation (≥6 months) on liver steatosis and fibrosis in chronic liver disease. More studies should be conducted on the survival outcomes based on the disease severity.

## CONCLUSION

This meta-analysis showed that additional use of vitamin D may result in modest improvements in ALT, AST, GGT, HOMA-IR, insulin, TG, and HDL. However, it did not lead to significant differences in survival, CRP, IL-6, TC, LDL, adiponectin, FPG, INR, albumin, and bilirubin. Vitamin D neither improved nor worsened CAP and LSM, and its effect on liver steatosis or fibrosis should be further investigated.

## Supplementary Material

nuaf117_Supplementary_Data

## Data Availability

The datasets can be found in the full-text articles included in the systematic review and meta-analysis.
