# Supplementary material for: Role of Vitamin D Supplementation in Chronic Liver Disease: A Systematic Review and Meta-Analysis of Randomized Controlled Trials
Source: Nutr Rev. 2025 Jul 11;83(11):2043–54. doi: 10.1093/nutrit/nuaf117 (PMC12512233; doi:10.1093/nutrit/nuaf117)
Supplement: nuaf117_Supplementary_Data [file nuaf117_supplementary_data.zip › Supplementary file S5-S6.docx]

**SUPPLEMENTARY MATERIAL – SUPPLEMENTARY FILE S5-S6**

**SUPPLEMENTARY FILE S5.** Miscellaneous (CRP, IL-6, INR, albumin, bilirubin, survival, bone mineral density, sarcopenia)

**SUPPLEMENTARY FILE S6.** Virologic response

**FIGURE AND TABLE LEGENDS**

**SUPPLEMENTARY FILE S5.** Miscellaneous (CRP, IL-6, INR, albumin, bilirubin, survival, bone mineral density, sarcopenia).

Figure S5.1a. Forest plot showing C reactive protein (CRP) change in vitamin D and control groups.

Figure S5.1b. Funnel plot for CRP.

Figure S5.1c. Forest plot with leave-one-out analysis for CRP.

Figure S5.1d. Baujat plot for CRP.

Figure S5.2. Forest plot showing CRP change in vitamin D and control groups by length of intervention.

Figure S5.3. Forest plot showing CRP change in vitamin D and control groups divided into vitamin D deficient/insufficient (< 30 ng/mL) and sufficient (≥ 30 ng/mL) studies.

Figure S5.4. Forest plot showing CRP change in vitamin D and control groups by type of chronic liver disease.

Figure S5.5a. Forest plot showing interleukin-6 (IL-6) change in vitamin D and control groups.

Figure S5.5b. Funnel plot for IL-6.

Figure S5.5c. Forest plot with leave-one-out analysis for IL-6.

Figure S5.5d. Baujat plot for IL-6.

Figure S5.6. Forest plot showing IL-6 change in vitamin D and control groups by length of intervention.

Figure S5.7. Forest plot showing IL-6 change in vitamin D and control groups divided into vitamin D deficient/insufficient (< 30 ng/mL) and sufficient (≥ 30 ng/mL) studies.

Figure S5.8. Forest plot showing IL-6 change in vitamin D and control groups by type of chronic liver disease.

Figure S5.9a. Forest plot showing INR change in vitamin D and control groups.

Figure S5.9b. Funnel plot for INR.

Figure S5.9c. Forest plot with leave-one-out analysis for INR.

Figure S5.9d. Baujat plot for INR.

Figure S5.10. Forest plot showing INR change in vitamin D and control groups by length of intervention.

Figure S5.11. Forest plot showing INR change in vitamin D and control groups divided into vitamin D deficient/insufficient (< 30 ng/mL) and sufficient (≥ 30 ng/mL) studies.

Figure S5.12. Forest plot showing INR change in vitamin D and control groups excluding high-risk biased studies.

Figure S5.13. Forest plot showing INR change in vitamin D and control groups by type of chronic liver disease.

Figure S5.14a. Forest plot showing albumin change in vitamin D and control groups.

Figure S5.14b. Funnel plot for albumin.

Figure S5.14c. Forest plot with leave-one-out analysis for albumin.

Figure S5.14d. Baujat plot for albumin.

Figure S5.15. Forest plot showing albumin change in vitamin D and control groups by length of intervention.

Figure S5.16. Forest plot showing albumin change in vitamin D and control groups divided into vitamin D deficient/insufficient (< 30 ng/mL) and sufficient (≥ 30 ng/mL) studies.

Figure S5.17. Forest plot showing albumin change in vitamin D and control groups excluding high-risk biased studies.

Figure S5.18. Forest plot showing albumin change in vitamin D and control groups by type of chronic liver disease.

Figure S5.19a. Forest plot showing bilirubin change in vitamin D and control groups.

Figure S5.19b. Funnel plot for bilirubin.

Figure S5.19c. Forest plot with leave-one-out analysis for bilirubin.

Figure S5.19d. Baujat plot for bilirubin.

Figure S5.20. Forest plot showing bilirubin change in vitamin D and control groups by length of intervention.

Figure S5.21. Forest plot showing bilirubin change in vitamin D and control groups divided into vitamin D deficient/insufficient (< 30 ng/mL) and sufficient (≥ 30 ng/mL) studies.

Figure S5.22. Forest plot showing bilirubin change in vitamin D and control groups excluding high-risk biased studies.

Figure S5.23. Forest plot showing bilirubin change in vitamin D and control groups by type of chronic liver disease.

Figure S5.24. Forest plot showing survival in vitamin D and control groups excluding high-risk biased studies.

Figure S5.25. Forest plot with leave-one-out analysis for survival.

Figure S5.26. Forest plot showing survival in chronic hepatitis and cirrhosis groups

File S5.27. Bone mineral density and skeletal muscles in vitamin D and control groups.

Figure S5.28. Risk of bias assessment (RoB-2 tool) for bone mineral density.

**SUPPLEMENTARY FILE S6.** Virologic response.

Figure S6.1. Forest plot showing the efficacy of vitamin D supplementation in combination with conventional antiviral therapy in treatment of chronic hepatitis C at 24-weeks after completion of therapy (sustained virologic response = SVR).

Figure S6.2. Forest plot showing the efficacy of vitamin D supplementation in combination with conventional antiviral therapy in treatment of chronic hepatitis C after completion of therapy (end of treatment response = ETR).

Figure S6.3. Forest plot showing the efficacy of vitamin D supplementation in combination with conventional antiviral therapy in treatment of chronic hepatitis C 12-weeks after the initiation of therapy (early virologic response = EVR).

Figure S6.4. Forest plot showing the efficacy of vitamin D supplementation in combination with conventional antiviral therapy in treatment of chronic hepatitis C 4-weeks after the initiation of therapy (rapid virologic response = RVR).

Figure S6.5. Risk of bias assessment (RoB-2 tool) for sustained virologic response.

**SUPPLEMENTARY FILE S5.** CRP, IL-6, INR, albumin, bilirubin, bone mineral density, sarcopenia.


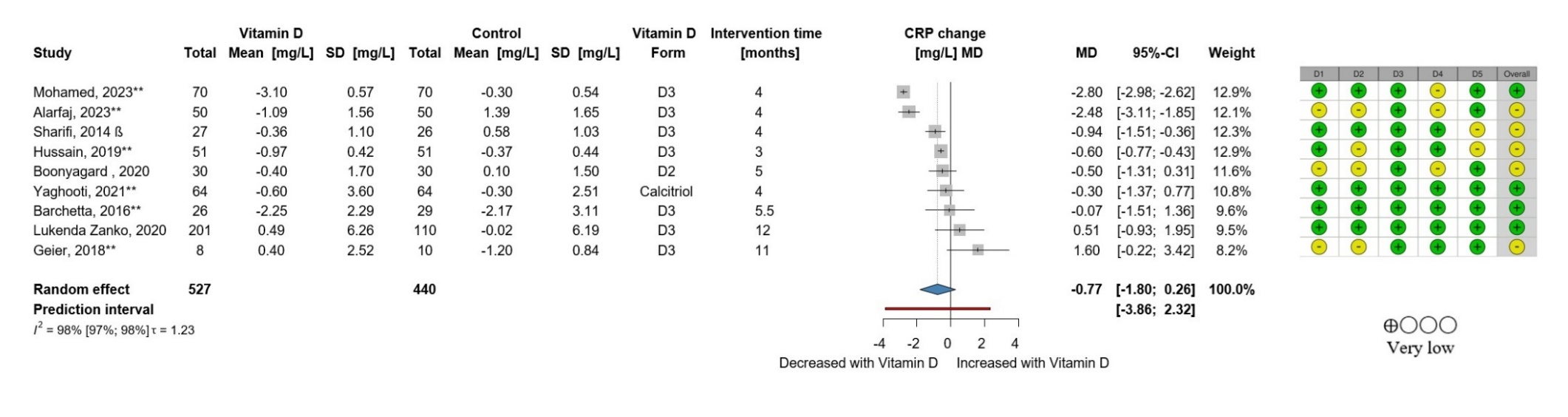


*Figure S5.1a. Forest plot showing C reactive protein (CRP) change in vitamin D and control groups. CI: confidence interval; CRP: C reactive protein; MD: mean difference; SD: standard deviation. If the study is indicated with **, then the change value is an estimated change value in that study. The β means that the mean and SD are estimated mean and SD in that study. See raw data and synthesis methods.*

*Figure S5.1b. Funnel plot for CRP (p = 0.5418). Not enough articles (> 10) for appropriate analysis of publication bias.*

*Not run.*

*Figure S5.1c. Forest plot with leave-one-out analysis for CRP.*

*Figure S5.1d. Baujat plot for CRP.*


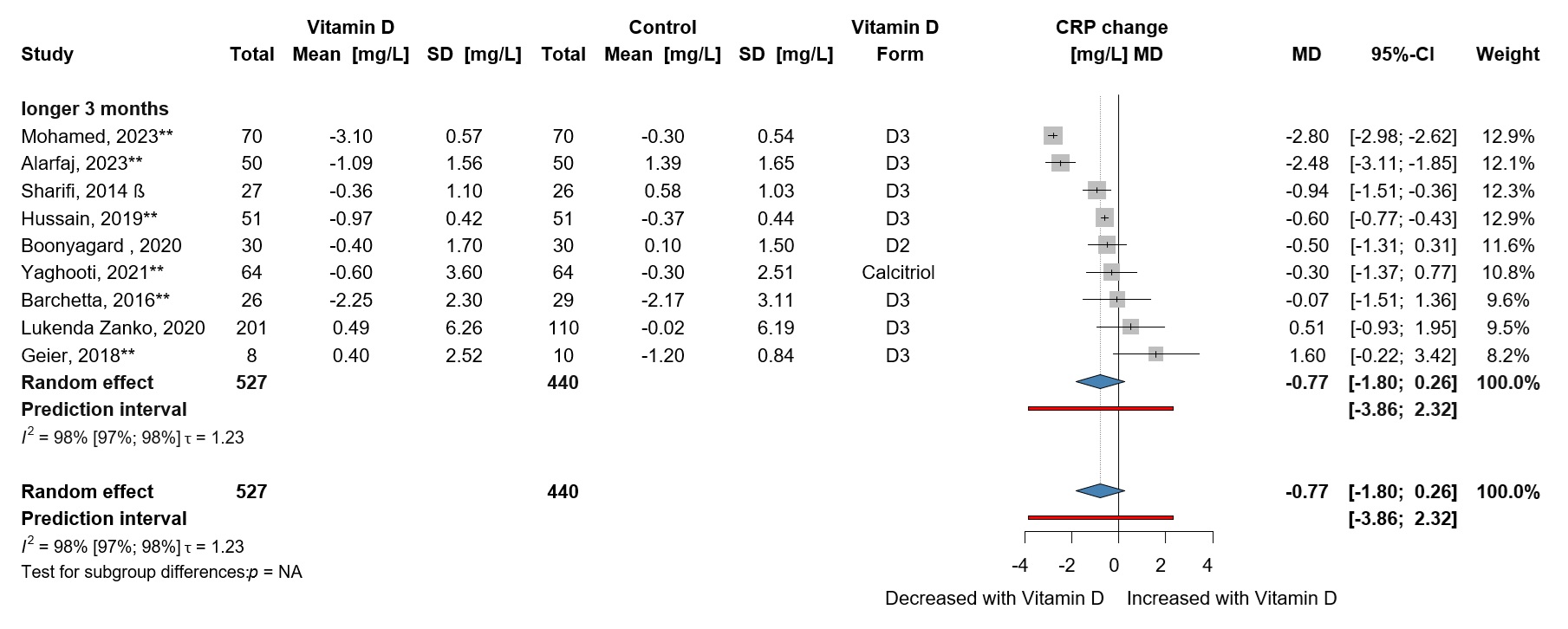


*Figure S5.2. Forest plot showing CRP change in vitamin D and control groups by length of intervention. CI: confidence interval; CRP: C reactive protein; MD: mean difference; SD: standard deviation. If the study is indicated with **, then the change value is an estimated change value in that study. The β means that the mean and SD are estimated mean and SD in that study. See raw data and synthesis methods.*


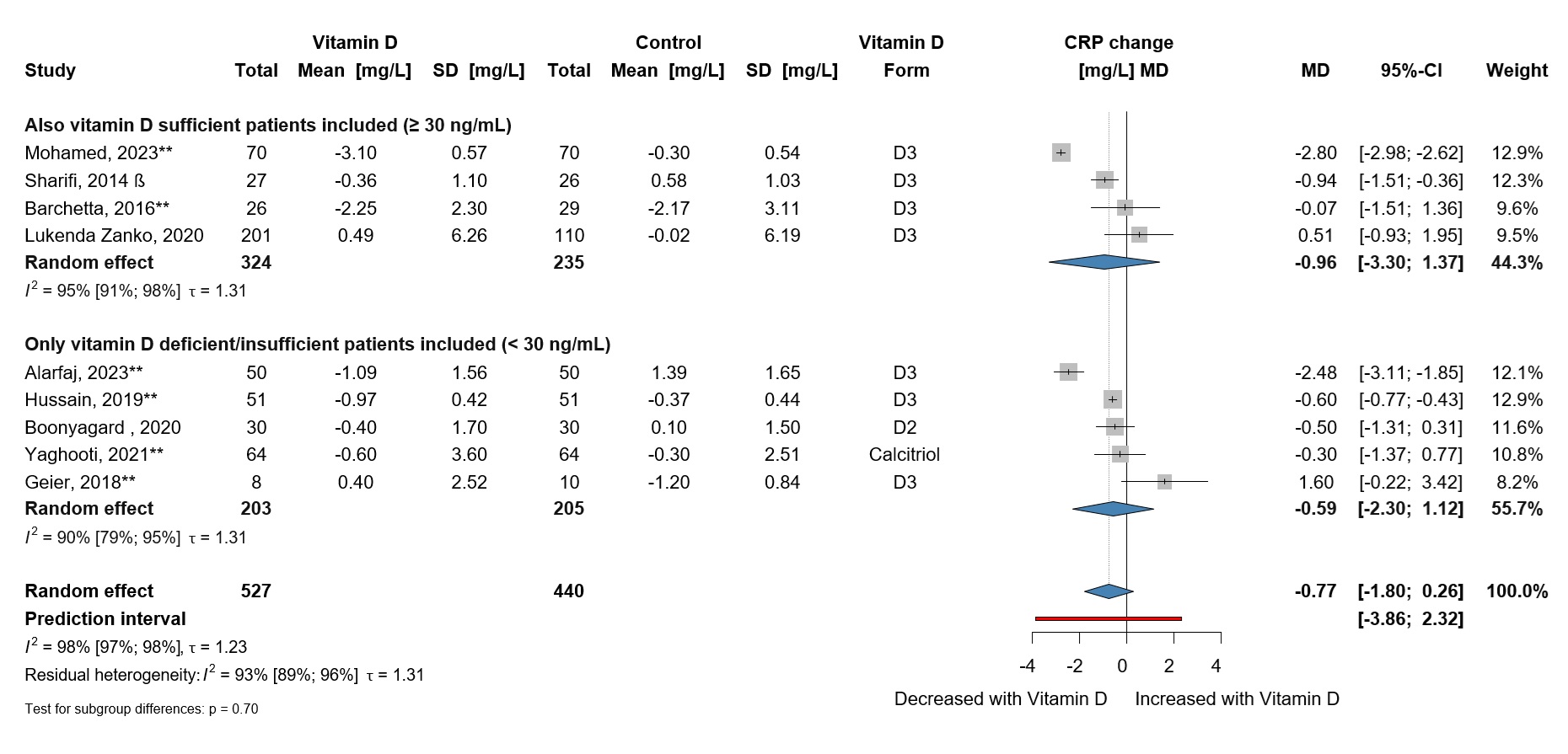


*Figure S5.3. Forest plot showing CRP change in vitamin D and control groups divided into vitamin D deficient/insufficient (< 30 ng/mL) and sufficient (≥ 30 ng/mL) studies. CI: confidence interval; CRP: C reactive protein; MD: mean difference; SD: standard deviation. If the study is indicated with **, then the change value is an estimated change value in that study. The β means that the mean and SD are estimated mean and SD in that study. See raw data and synthesis methods.*


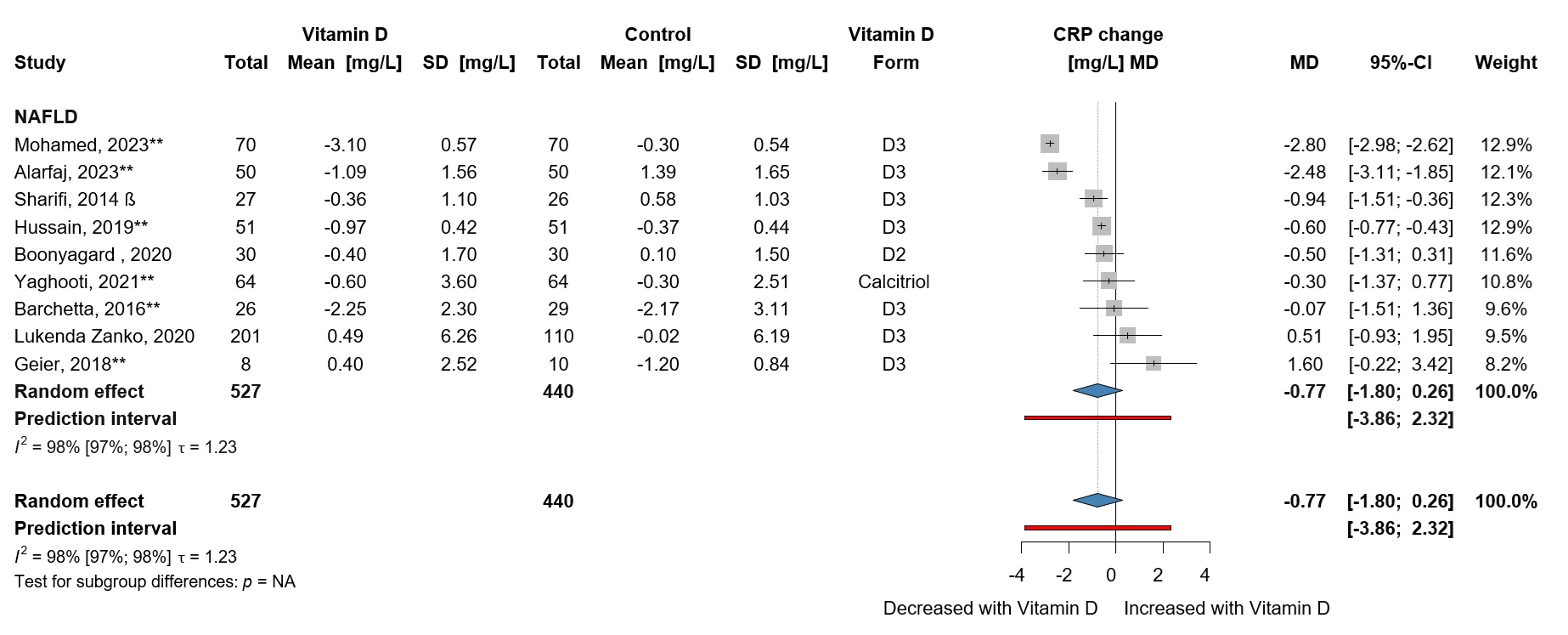


*Figure S5.4. Forest plot showing CRP change in vitamin D and control groups by type of chronic liver disease. CI: confidence interval; CRP: C reactive protein; MD: mean difference; SD: standard deviation. If the study is indicated with **, then the change value is an estimated change value in that study. The β means that the mean and SD are estimated mean and SD in that study. See raw data and synthesis methods.*


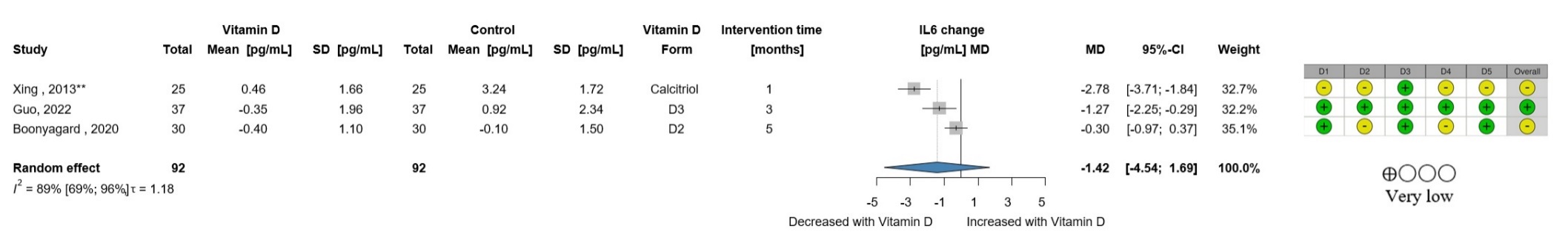


*Figure S5.5a. Forest plot showing interleukin-6 (IL-6) change in vitamin D and control groups. CI: confidence interval; IL-6: interleukin 6; MD: mean difference; SD: standard deviation. If the study is indicated with **, then the change value is an estimated change value in that study. The β means that the mean and SD are estimated mean and SD in that study. See raw data and synthesis methods.*

*Figure S5.5b. Funnel plot for IL-6 (p = 0.4129). Not enough articles (> 10) for appropriate analysis of publication bias.*

*Not run.*

*Figure S5.5c. Forest plot with leave-one-out analysis for IL-6.*

*Not run.*

*Figure S5.5d. Baujat plot for IL-6.*

*Not run.*

*Figure S5.6. Forest plot showing IL-6 change in vitamin D and control groups by length of intervention.*


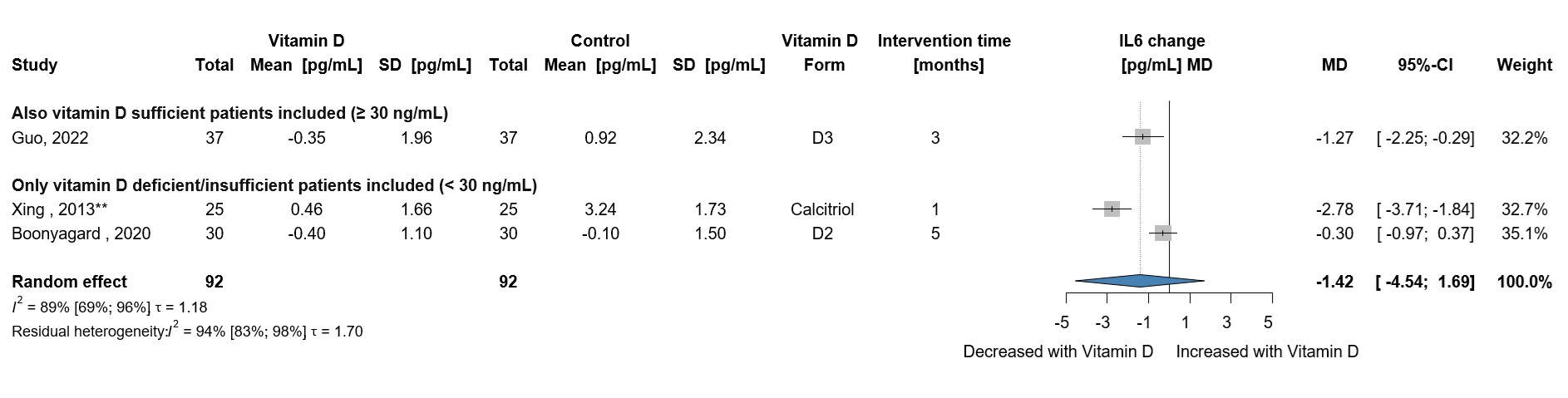


*Figure S5.7. Forest plot showing IL-6 change in vitamin D and control groups divided into vitamin D deficient/insufficient (< 30 ng/mL) and sufficient (≥ 30 ng/mL) studies. CI: confidence interval; IL-6: interleukin 6; MD: mean difference; SD: standard deviation. If the study is indicated with **, then the change value is an estimated change value in that study. The β means that the mean and SD are estimated mean and SD in that study. See raw data and synthesis methods.*


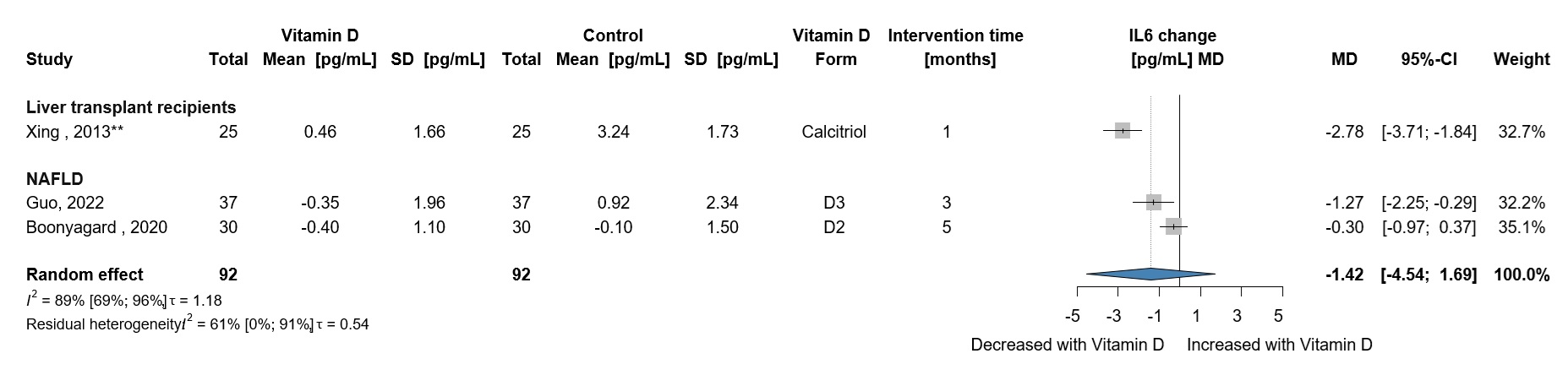


*Figure S5.8. Forest plot showing IL-6 change in vitamin D and control groups by type of chronic liver disease. CI: confidence interval; IL-6: interleukin 6; MD: mean difference; SD: standard deviation. If the study is indicated with **, then the change value is an estimated change value in that study. The β means that the mean and SD are estimated mean and SD in that study. See raw data and synthesis methods.*


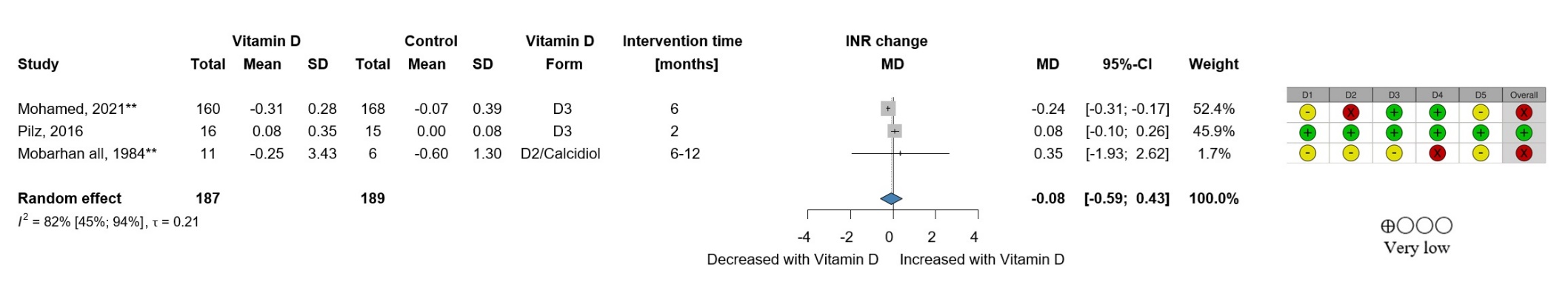


*Figure S5.9a. Forest plot showing INR change in vitamin D and control groups. CI: confidence interval; INR: international normalized ratio; MD: mean difference; SD: standard deviation. If the study is indicated with **, then the change value is an estimated change value in that study. The β means that the mean and SD are estimated mean and SD in that study. See raw data and synthesis methods.*

*Figure S5.9b. Funnel plot for INR (p = 0.5877). Not enough articles (> 10) for appropriate analysis of publication bias.*

*Not run.*

*Figure S5.9c. Forest plot with leave-one-out analysis for INR.*

*Not run.*

*Figure S5.9d. Baujat plot for INR.*

*Not run.*

*Figure S5.10. Forest plot showing INR change in vitamin D and control groups by length of intervention.*


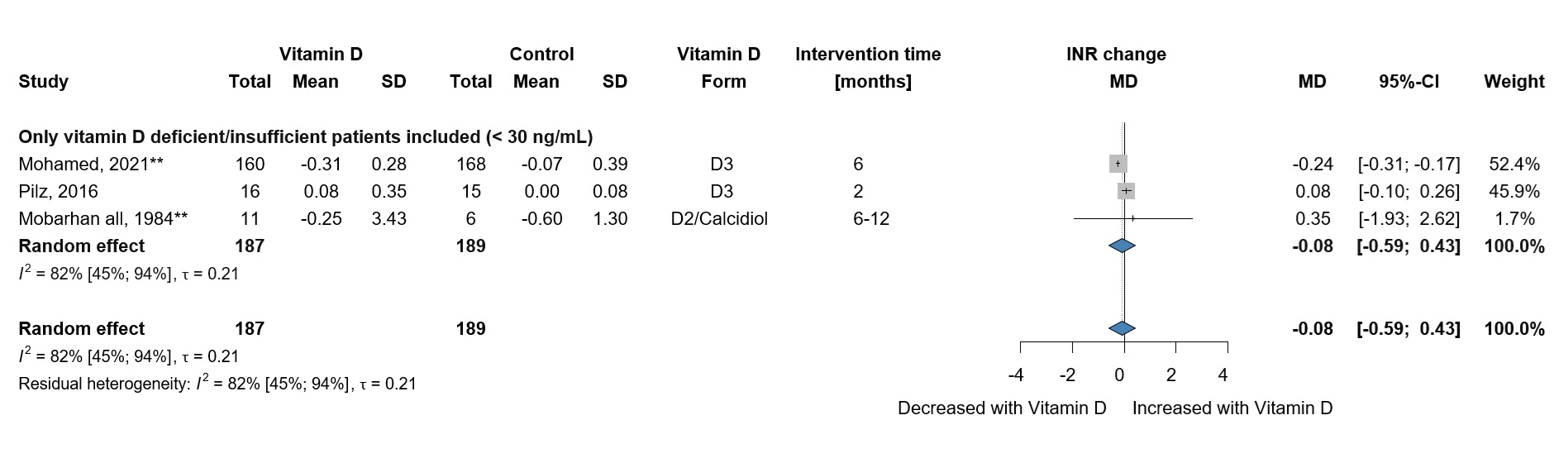


*Figure S5.11. Forest plot showing INR change in vitamin D and control groups divided into vitamin D deficient/insufficient (< 30 ng/mL) and sufficient (≥ 30 ng/mL) studies. CI: confidence interval; INR: international normalized ratio; MD: mean difference; SD: standard deviation. If the study is indicated with **, then the change value is an estimated change value in that study. The β means that the mean and SD are estimated mean and SD in that study. See raw data and synthesis methods.*


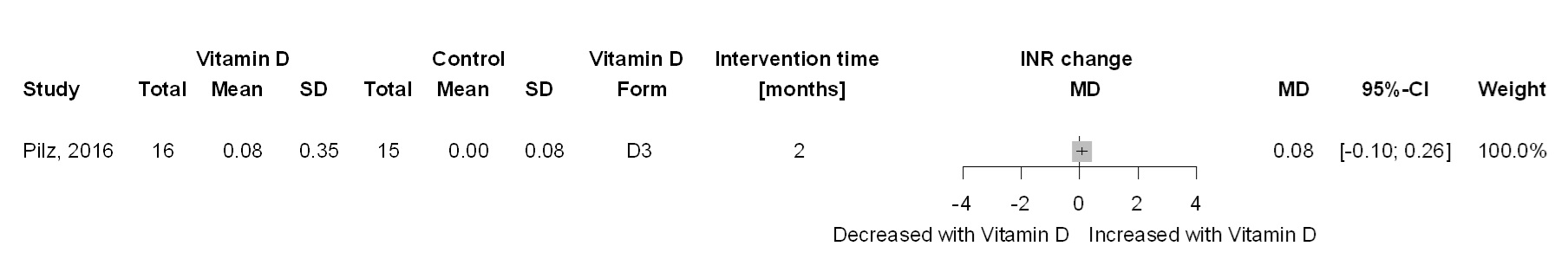


*Figure S5.12. Forest plot showing INR change in vitamin D and control groups excluding high-risk biased studies. CI: confidence interval; INR: international normalized ratio; MD: mean difference; SD: standard deviation. If the study is indicated with **, then the change value is an estimated change value in that study. The β means that the mean and SD are estimated mean and SD in that study. See raw data and synthesis methods.*


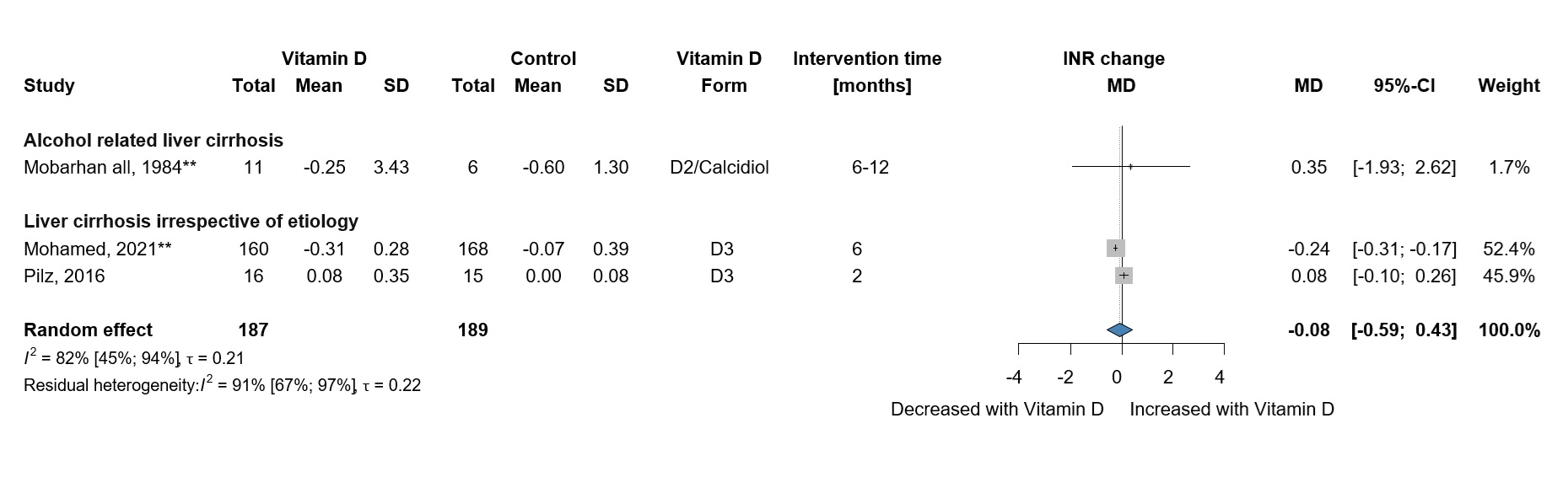


*Figure S5.13. Forest plot showing INR change in vitamin D and control groups by type of chronic liver disease. CI: confidence interval; INR: international normalized ratio; MD: mean difference; SD: standard deviation. If the study is indicated with **, then the change value is an estimated change value in that study. The β means that the mean and SD are estimated mean and SD in that study. See raw data and synthesis methods.*


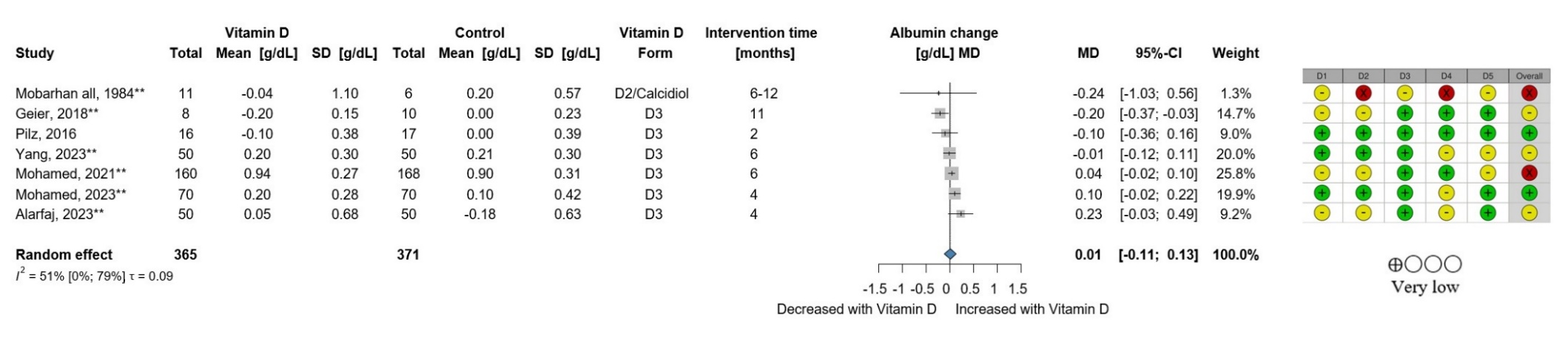


*Figure S5.14a. Forest plot showing albumin change in vitamin D and control groups. CI: confidence interval; MD: mean difference; SD: standard deviation. If the study is indicated with **, then the change value is an estimated change value in that study. The β means that the mean and SD are estimated mean and SD in that study. See raw data and synthesis methods.*

*Figure S5.14b. Funnel plot for albumin (p = 0.5437). Not enough articles (> 10) for appropriate analysis of publication bias.*

*Figure S5.14c. Forest plot with leave-one-out analysis for albumin.*

*Figure S5.14d. Baujat plot for albumin.*


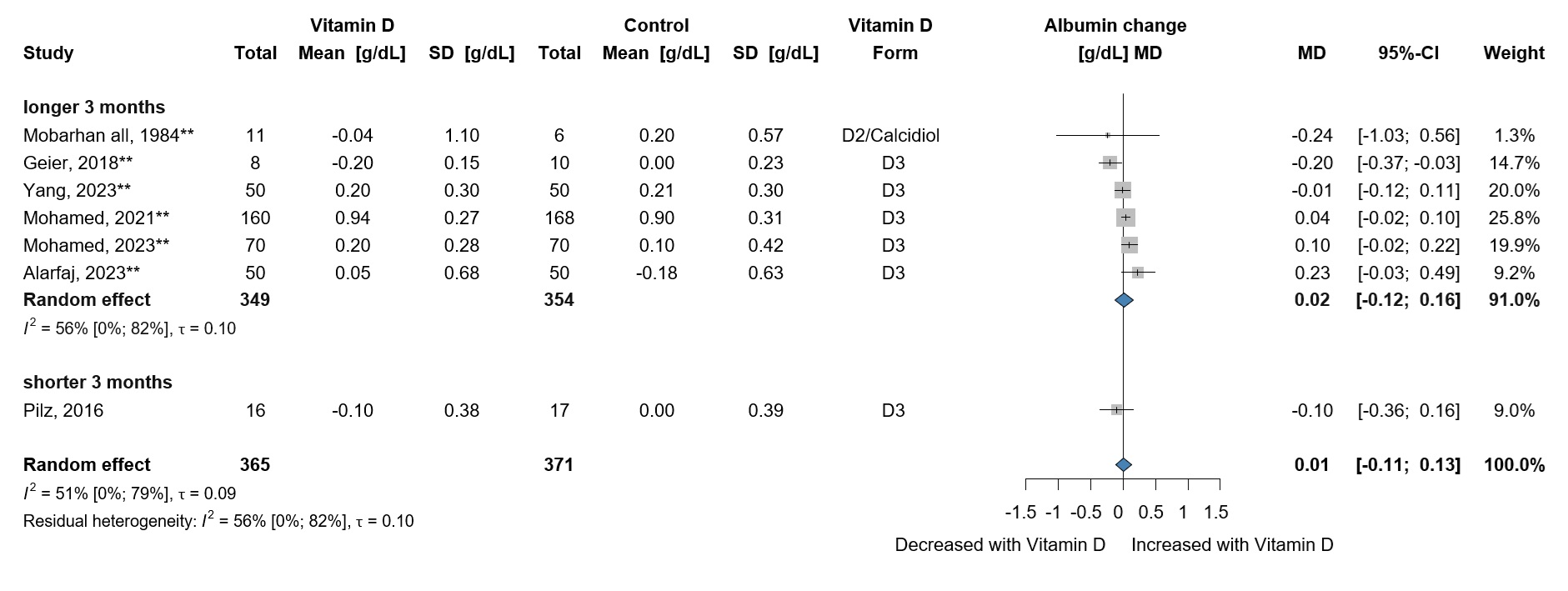


*Figure S5.15. Forest plot showing albumin change in vitamin D and control groups by length of intervention. CI: confidence interval; MD: mean difference; SD: standard deviation. If the study is indicated with **, then the change value is an estimated change value in that study. The β means that the mean and SD are estimated mean and SD in that study. See raw data and synthesis methods.*


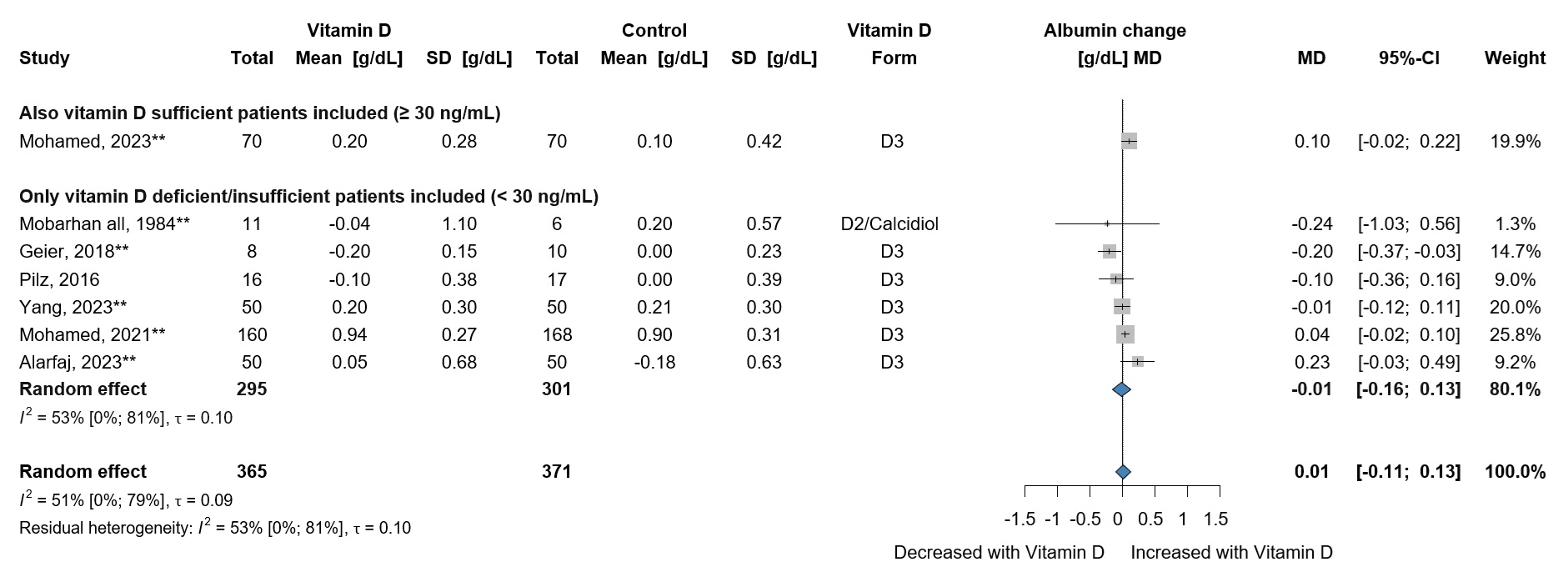


*Figure S5.16. Forest plot showing albumin change in vitamin D and control groups divided into vitamin D deficient/insufficient (< 30 ng/mL) and sufficient (≥ 30 ng/mL) studies. CI: confidence interval; MD: mean difference; SD: standard deviation. If the study is indicated with **, then the change value is an estimated change value in that study. The β means that the mean and SD are estimated mean and SD in that study. See raw data and synthesis methods.*


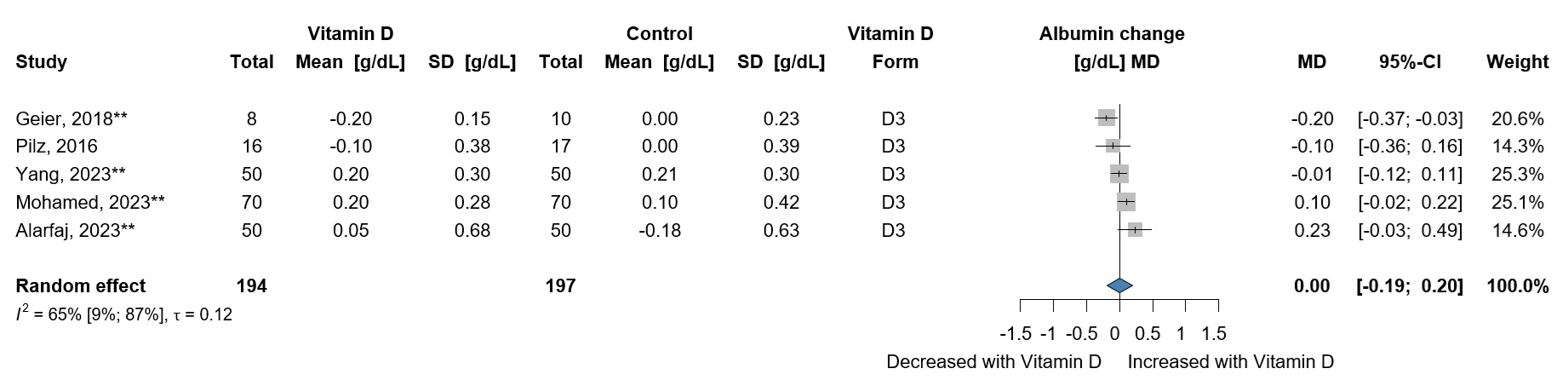


*Figure S5.17. Forest plot showing albumin change in vitamin D and control groups excluding high-risk biased studies. CI: confidence interval; MD: mean difference; SD: standard deviation. If the study is indicated with **, then the change value is an estimated change value in that study. The β means that the mean and SD are estimated mean and SD in that study. See raw data and synthesis methods.*


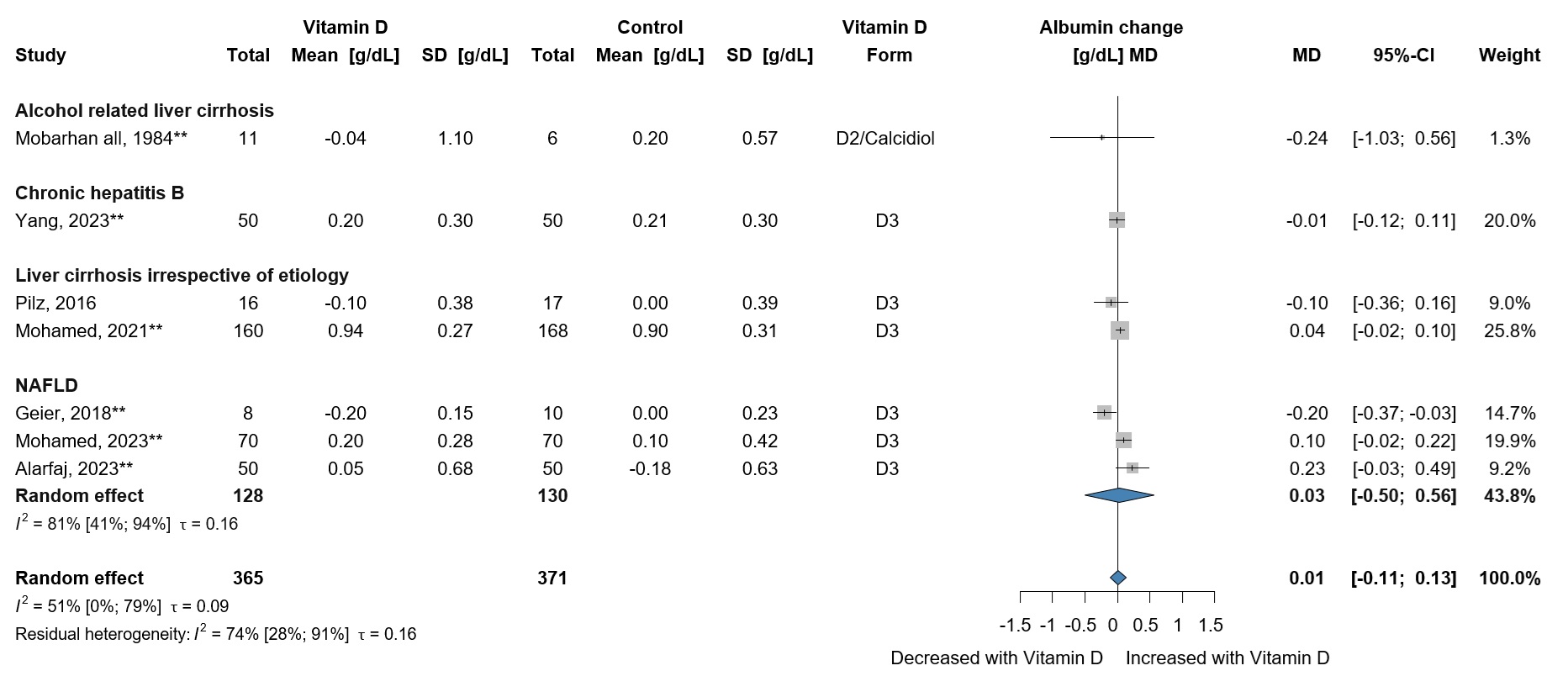


*Figure S5.18. Forest plot showing albumin change in vitamin D and control groups by type of chronic liver disease. CI: confidence interval; MD: mean difference; SD: standard deviation. If the study is indicated with **, then the change value is an estimated change value in that study. The β means that the mean and SD are estimated mean and SD in that study. See raw data and synthesis methods.*


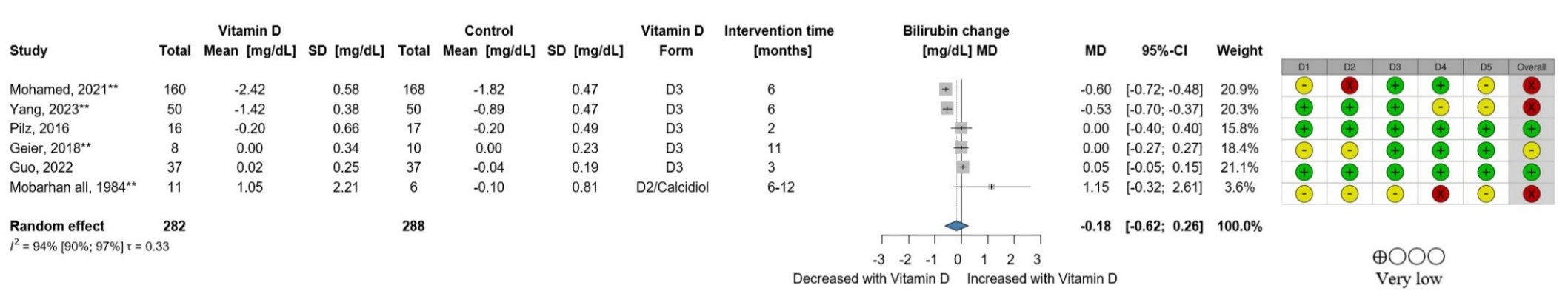


*Figure S5.19a. Forest plot showing bilirubin change in vitamin D and control groups. CI: confidence interval; MD: mean difference; SD: standard deviation. If the study is indicated with **, then the change value is an estimated change value in that study. The β means that the mean and SD are estimated mean and SD in that study. See raw data and synthesis methods.*

*Figure S5.19b. Funnel plot for bilirubin (p = 0.7788). Not enough articles (> 10) for appropriate analysis of publication bias.*

*Figure S5.19c. Forest plot with leave-one-out analysis for bilirubin.*

*Figure S5.19d. Baujat plot for bilirubin.*


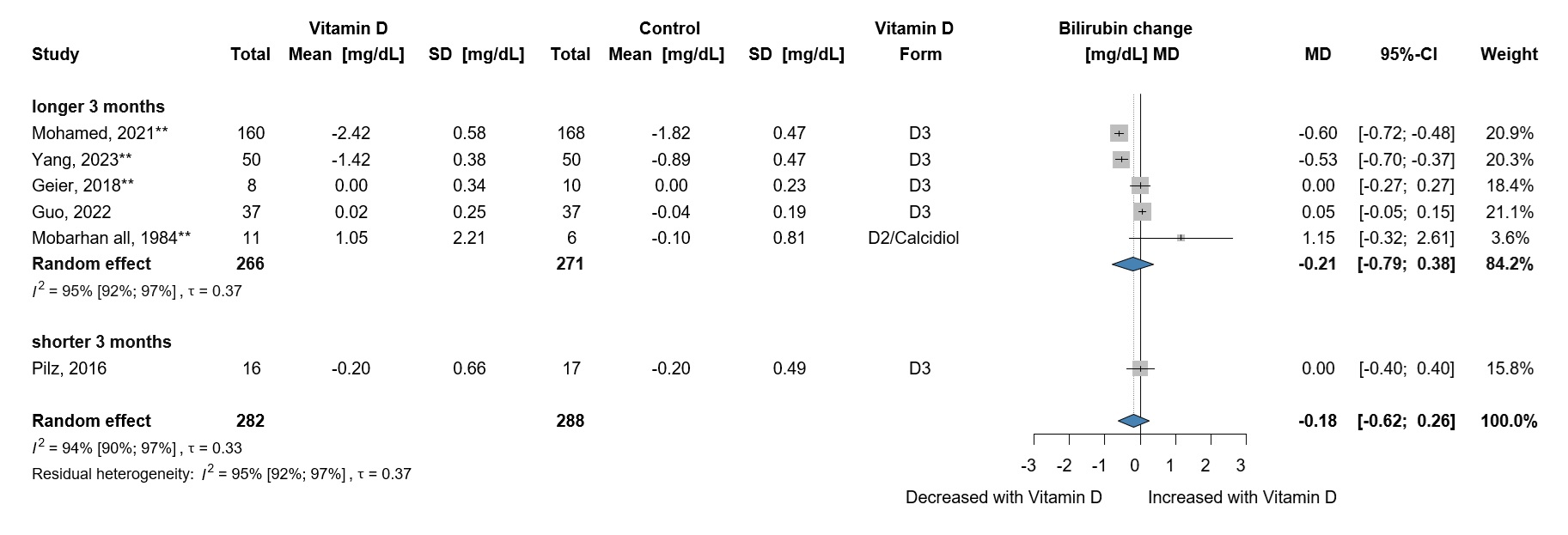


*Figure S5.20. Forest plot showing bilirubin change in vitamin D and control groups by length of intervention. CI: confidence interval; MD: mean difference; SD: standard deviation. If the study is indicated with **, then the change value is an estimated change value in that study. The β means that the mean and SD are estimated mean and SD in that study. See raw data and synthesis methods.*


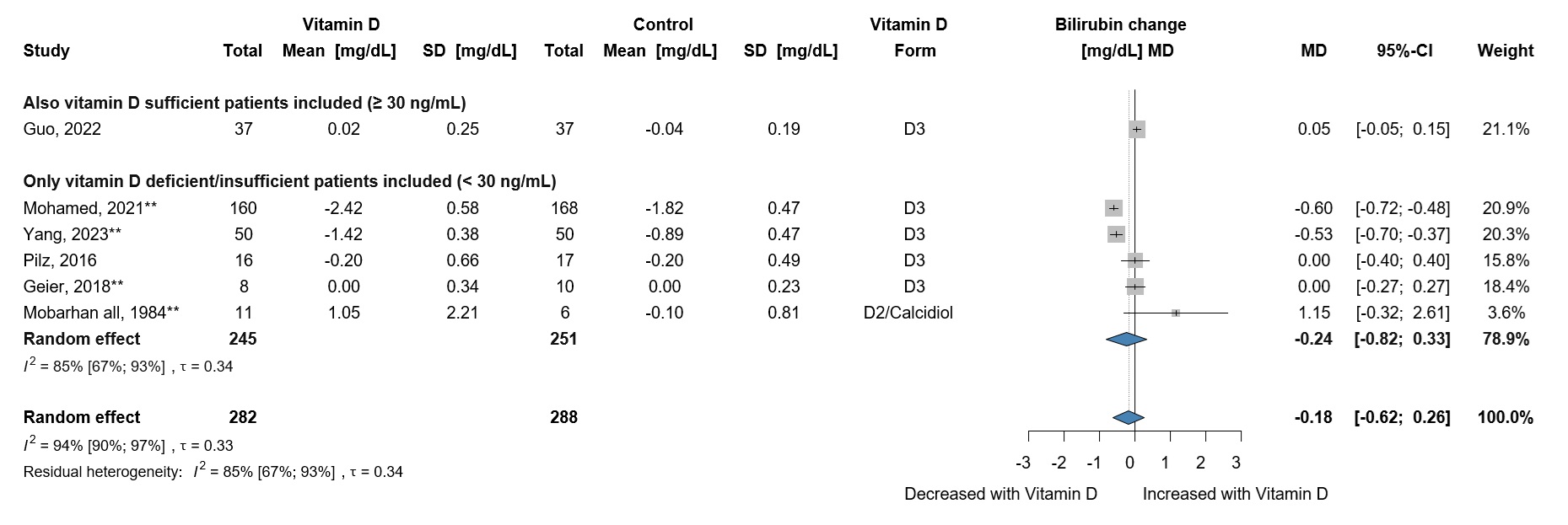


*Figure S5.21. Forest plot showing bilirubin change in vitamin D and control groups divided into vitamin D deficient/insufficient (< 30 ng/mL) and sufficient (≥ 30 ng/mL) studies. CI: confidence interval; MD: mean difference; SD: standard deviation. If the study is indicated with **, then the change value is an estimated change value in that study. The β means that the mean and SD are estimated mean and SD in that study. See raw data and synthesis methods.*


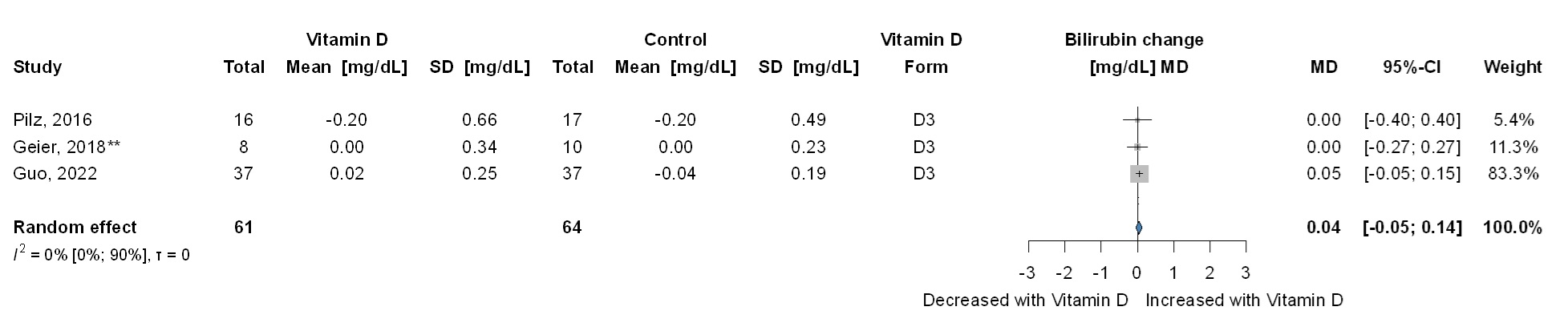


*Figure S5.22. Forest plot showing bilirubin change in vitamin D and control groups excluding high-risk biased studies. CI: confidence interval; MD: mean difference; SD: standard deviation. If the study is indicated with **, then the change value is an estimated change value in that study. The β means that the mean and SD are estimated mean and SD in that study. See raw data and synthesis methods.*


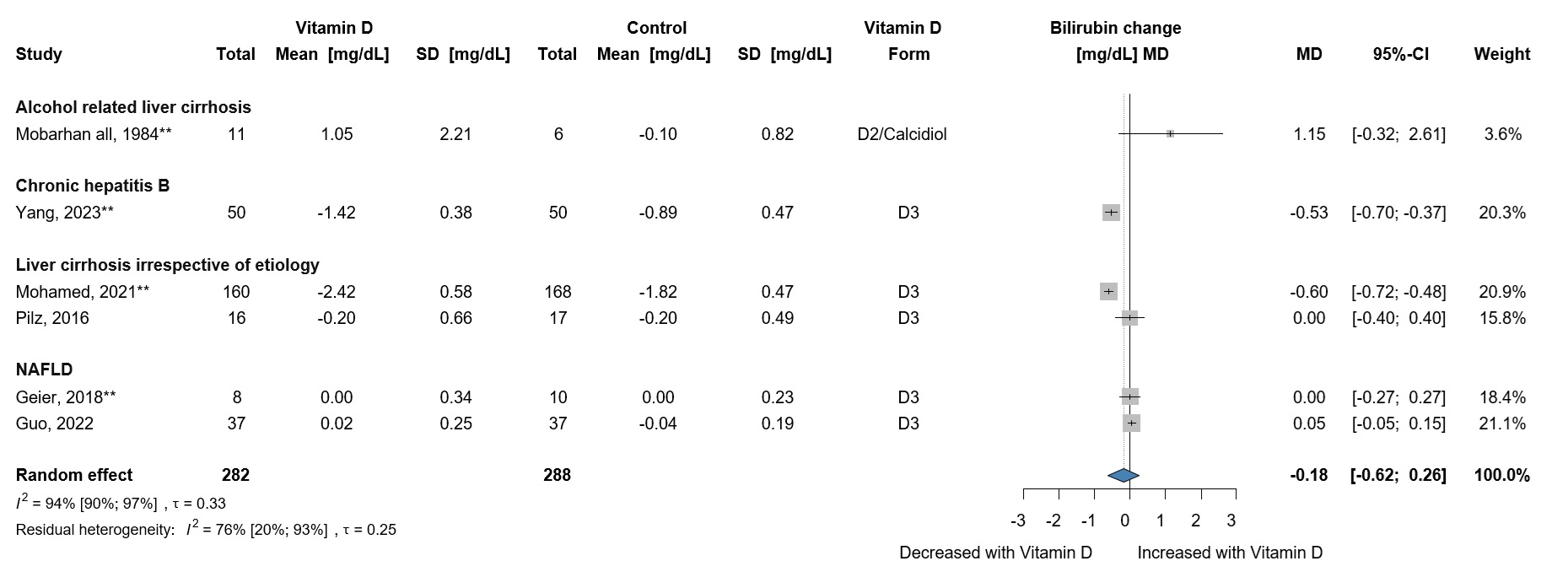


*Figure S5.23. Forest plot showing bilirubin change in vitamin D and control groups by type of chronic liver disease. CI: confidence interval; MD: mean difference; SD: standard deviation. If the study is indicated with **, then the change value is an estimated change value in that study. The β means that the mean and SD are estimated mean and SD in that study. See raw data and synthesis methods.*


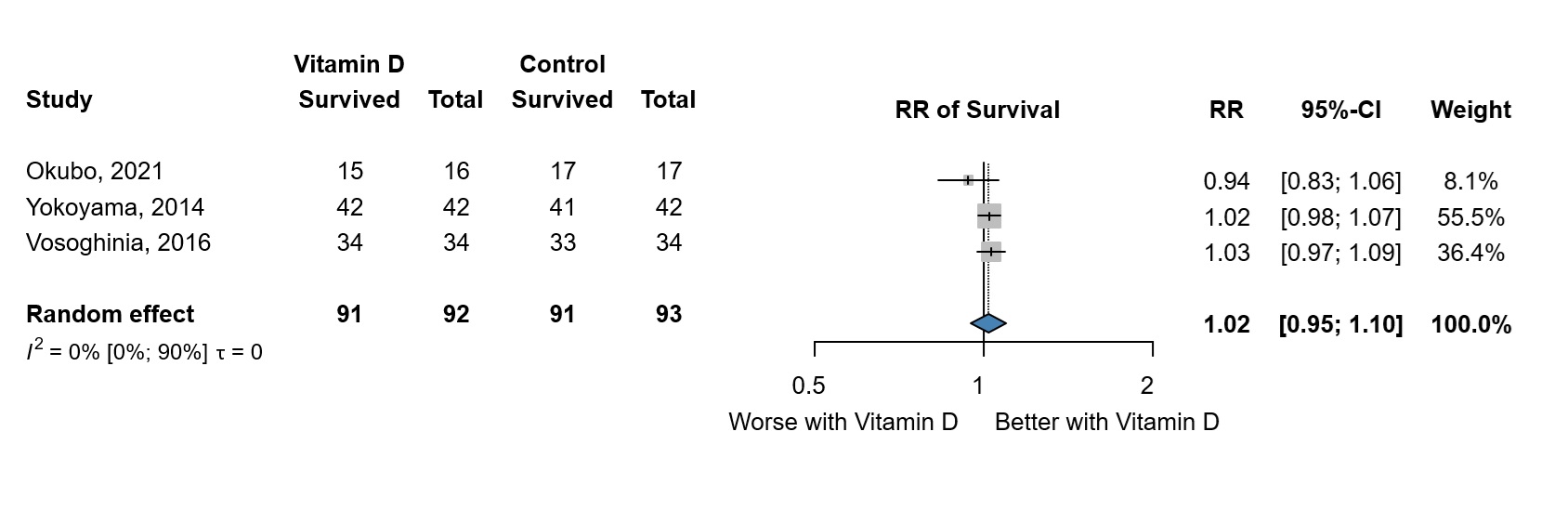


*Figure S5.24. Forest plot showing survival in vitamin D and control groups excluding high-risk biased studies. CI: confidence interval; RR: risk ratio.*

*Figure S5.25. Forest plot with leave-one-out analysis for survival.*

*
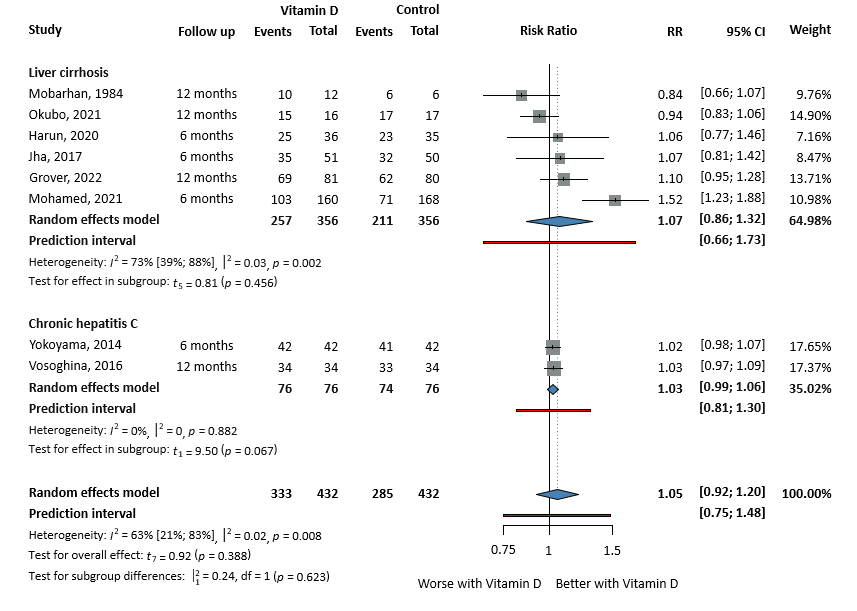
*

*Figure S5.26. Forest plot showing survival in chronic hepatitis and cirrhosis groups. CI: confidence interval; RR: risk ratio.*

**Bone mineral density (BMD) and skeletal muscles:**

We were unable to meta-analyze data on BMD as our surrogate outcome due to insufficient data and differences in reporting the outcome. In total, five RCTs evaluated the effect of VD on BMD. Recently, Grover et al. (41) did not find improvement in the BMD of the lumbar spine and left hip neck in patients after one year of D3 supplementation, despite an increased level of 25-hydroxyvitamin-D. Even though the number of patients with a decrease in BMD was lower than in the placebo arm, this result did not reach statistical significance.

Contrarily, Mobarhan et al. (8) concluded that supplementation either with D2 or calcidiol for 6 to 12 months improved BMD of the distal radius, whereas this improvement was not observed in the control group. Shiomi et al. (63) compared the effect of calcitriol on lumbar BMD separately for males and females with chronic hepatitis C (CHC) or chronic hepatitis B (CHB). Overall, 9/13 (69%) of treated males improved BMD after at least 12 months of intervention, while only 11/25 (44%) of treated women showed this effect. In the untreated group, just 5/13 (39%) of males and 3/25 (12%) of females increased lumbar BMD. Furthermore, another RCT (64) suggested calcitriol may inhibit the usual decrease in lumbar BMD in women with primary biliary cholangitis. Lastly, Atthakitmongkol et al. (32) showed that in patients with CHB treated with tenofovir disoproxil fumarate, D2 and calcium carbonate supplementation could prevent a decrease in BMD of total hip T-score, however, no difference was seen between VD-supplemented and the control group in BMD of the lumbar spine T-score.

Only two RCTs examined the role of VD on skeletal muscles. Okubo et al. suggested D3 may prevent a decrease in skeletal muscle index or restore muscle volume in cirrhotic patients (60). Boonyagard et al. indicated the elevation of skeletal muscle mass in the VD group (p=0.023) may have potential effect benefiting NAFLD patients (35).

*File S5.27. Bone mineral density and skeletal muscles in vitamin D and control groups.*


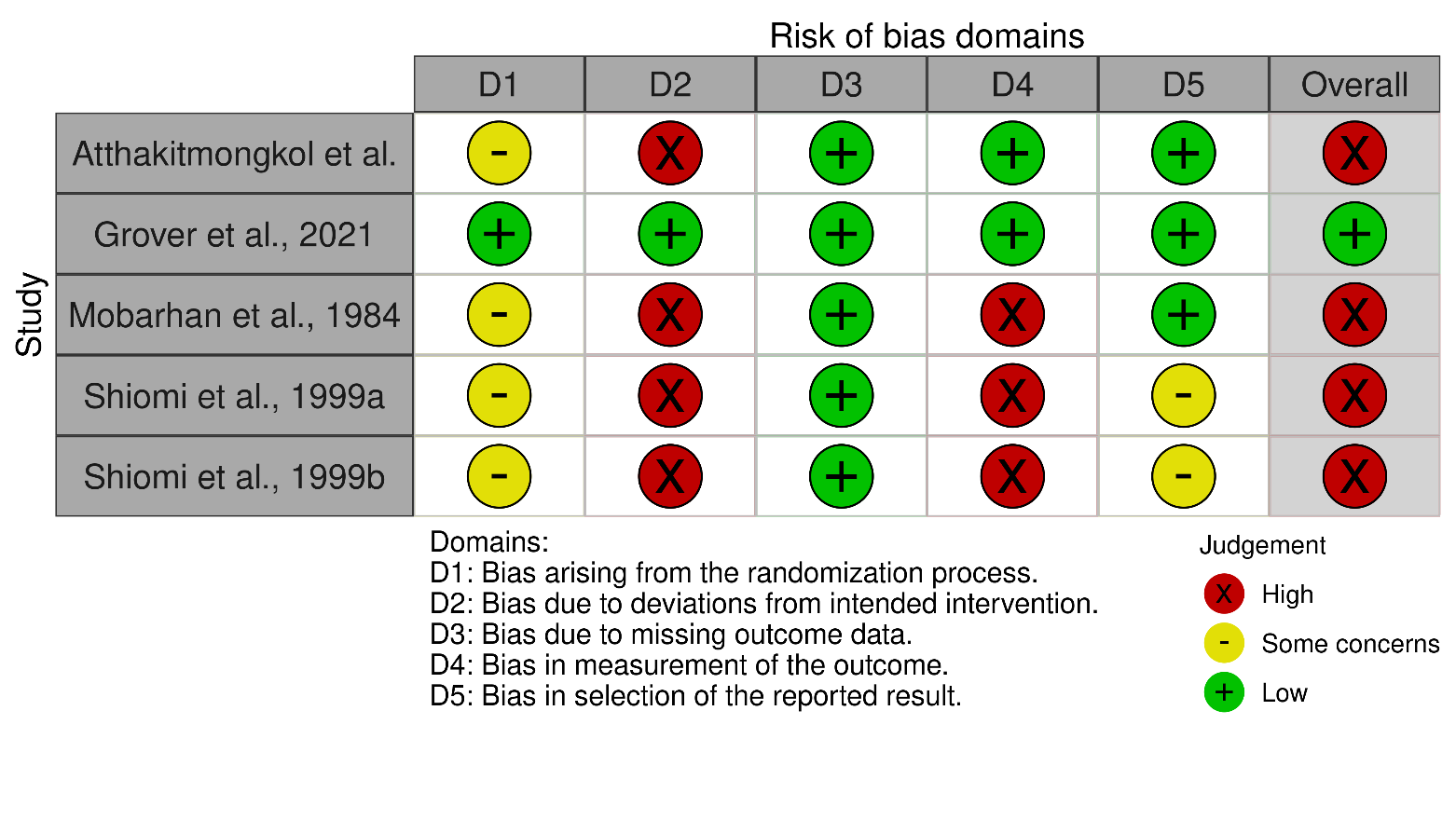


*Figure S5.28. Risk of bias assessment (RoB-2 tool) for bone mineral density.*

**SUPPLEMENTARY FILE S6.** Virologic response.


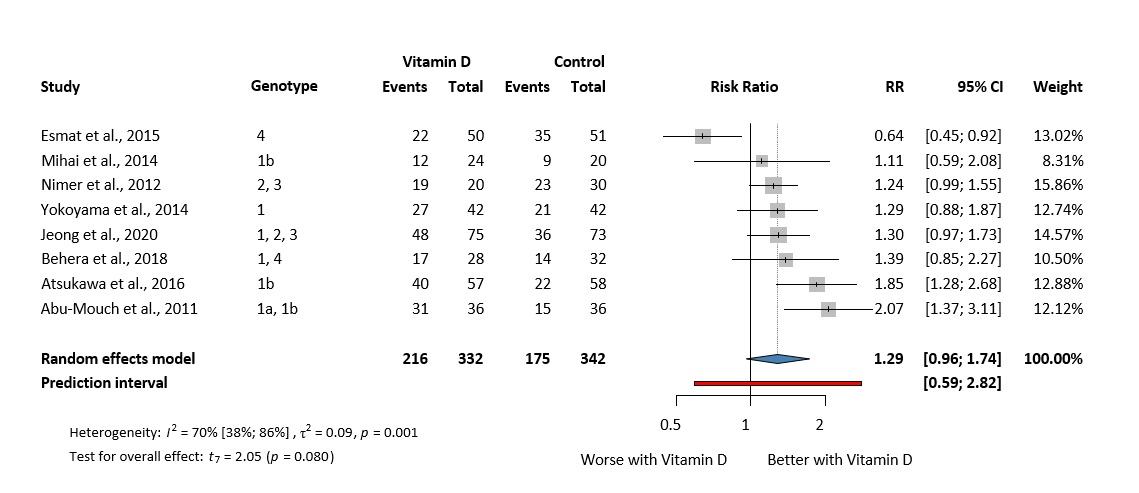


*Figure S6.1: Forest plot showing the efficacy of vitamin D supplementation in combination with conventional antiviral therapy in treatment of chronic hepatitis C at 24-weeks after completion of therapy (sustained virologic response = SVR). CI: confidence interval; RR: risk ratio.*


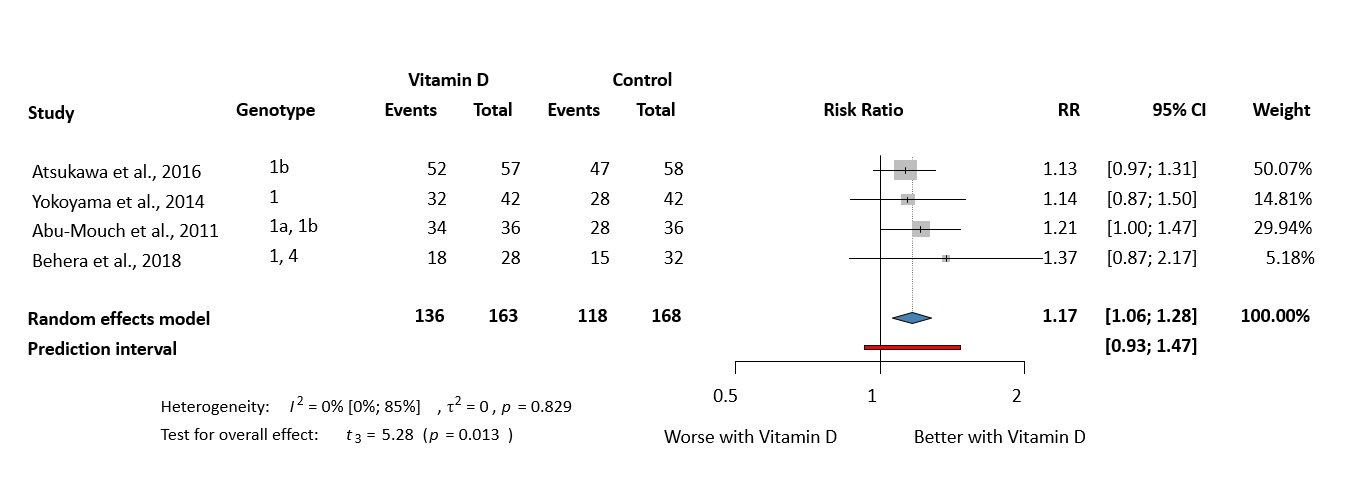


*Figure S6.2: Forest plot showing the efficacy of vitamin D supplementation in combination with conventional antiviral therapy in treatment of chronic hepatitis C after completion of therapy (end of treatment response = ETR). CI: confidence interval; RR: risk ratio.*


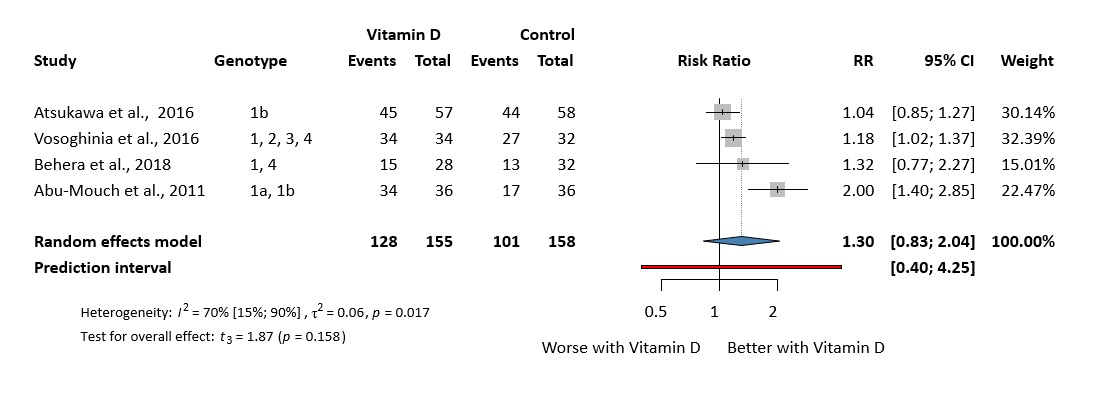


*Figure S6.3: Forest plot showing the efficacy of vitamin D supplementation in combination with conventional antiviral therapy in treatment of chronic hepatitis C 12-weeks after the initiation of therapy (early virologic response = EVR). CI: confidence interval; RR: risk ratio.*


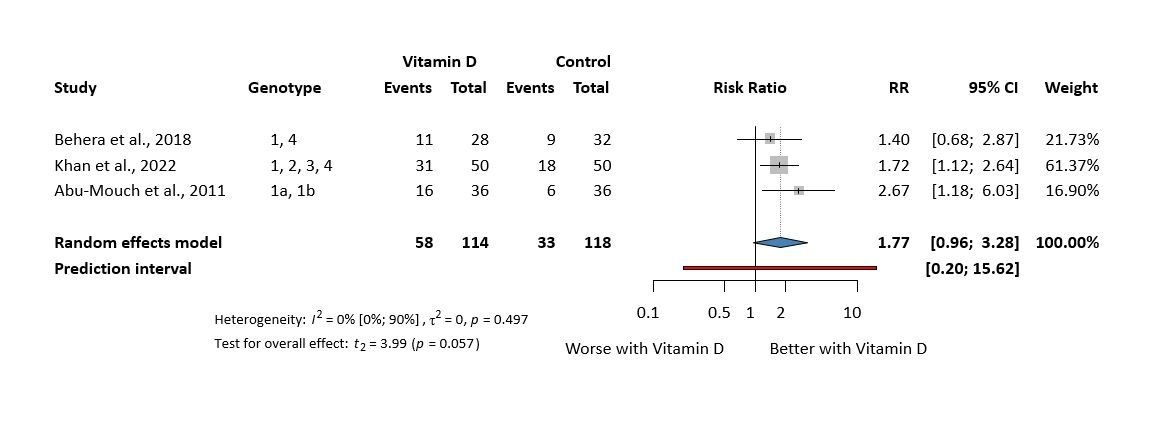


*Figure S6.4: Forest plot showing the efficacy of vitamin D supplementation in combination with conventional antiviral therapy in treatment of chronic hepatitis C 4-weeks after the initiation of therapy (rapid virologic response = RVR). CI: confidence interval; RR: risk ratio.*


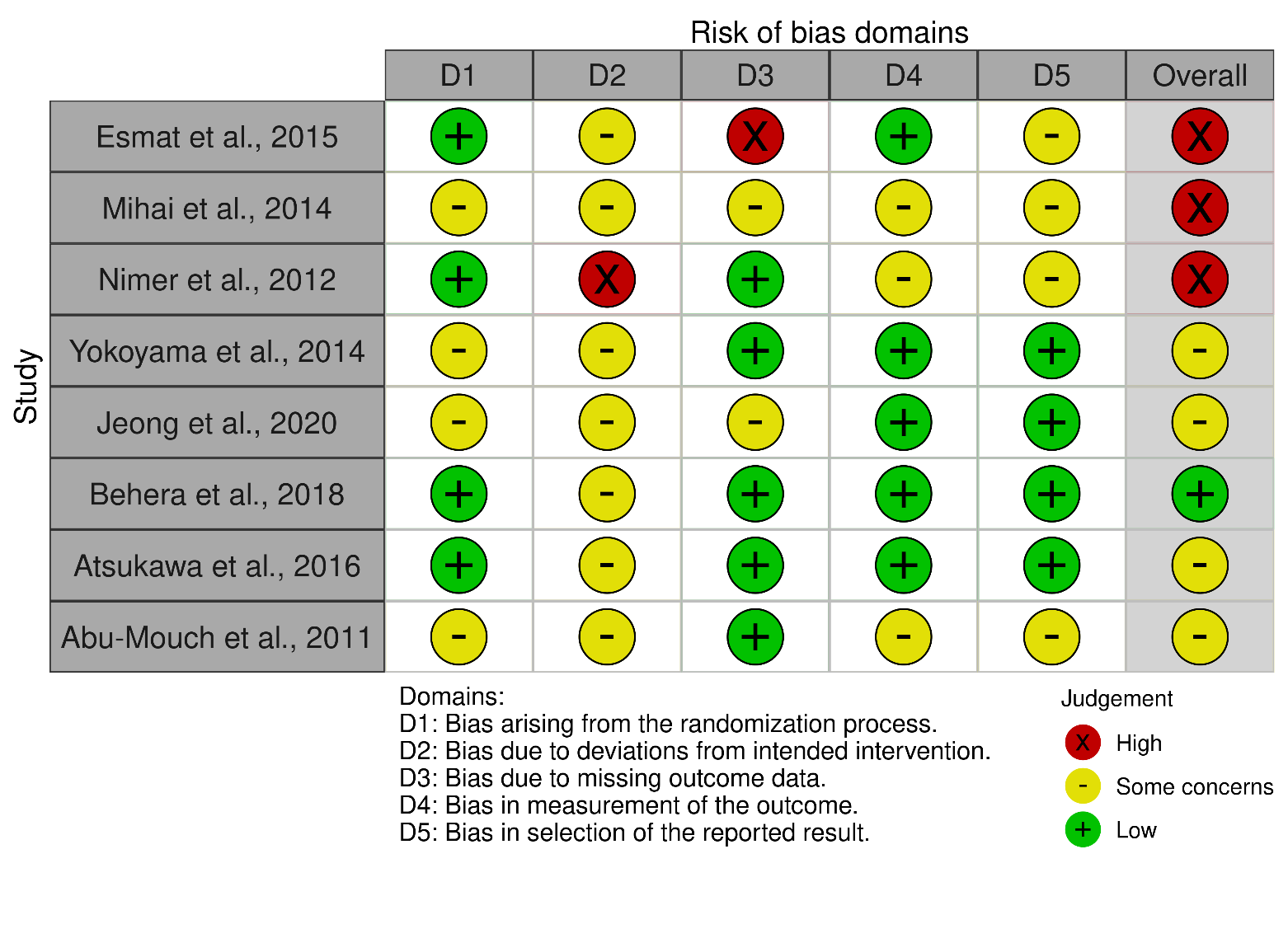


*Figure S6.5. Risk of bias assessment (RoB-2 tool) for sustained virologic response.*
