# Supplementary material for: Role of Vitamin D Supplementation in Chronic Liver Disease: A Systematic Review and Meta-Analysis of Randomized Controlled Trials
Source: Nutr Rev. 2025 Jul 11;83(11):2043–54. doi: 10.1093/nutrit/nuaf117 (PMC12512233; doi:10.1093/nutrit/nuaf117)
Supplement: nuaf117_Supplementary_Data [file nuaf117_supplementary_data.zip › Supplementary file S1.docx]

**SUPPLEMENTARY MATERIAL – SUPPLEMENTARY FILE 1**

**TITLE**

Role of Vitamin D Supplementation in Chronic Liver Disease: A Systematic Review and Meta-analysis of Randomized Controlled Trials

**AUTHORS**

Petrana Martineková^1,2^, Mahmoud Obeidat^1^, Mihaela Topala^1,3^, Szilárd Váncsa^1,4,5^, Dániel Sándor Veres^1,6^, Ádám Zolcsák^1,6^, Miheller Pál^1,7^, László Földvári-Nagy^1,8^, Peter Banovcin^1,9^, Bálint Erőss^1,4,5^, Péter Hegyi^1,4,5^, Krisztina Hagymasi^1,7^

**AFFILIATIONS**

1. Centre for Translational Medicine, Semmelweis University, Budapest, Hungary
2. Institute for Clinical and Experimental Medicine, Prague, Czech Republic
3. Carol Davila University of Medicine and Pharmacy, Bucharest, Romania
4. Institute of Pancreatic Diseases, Semmelweis University, Budapest, Hungary.
5. Institute for Translational Medicine, University of Pécs, Medical School, Pécs, Hungary
6. Department of Biophysics and Radiation Biology, Semmelweis University, Budapest, Hungary
7. Department of Surgery, Transplantation and Gastroenterology, Semmelweis University, Budapest, Hungary
8. Department of Morphology and Physiology, Faculty of Health Sciences, Semmelweis University, Budapest, Hungary
9. Gastroenterology Clinic, University Hospital in Martin, Jessenius Faculty of Medicine in Martin, Comenius University in Bratislava, Martin, Slovak Republic

**CORRESPONDING AUTHOR**

Dr. Hagymási Krisztina Ph.D.

Postal address: H-1082 Budapest, Üllői út 78, Hungary

Tel.: +36 1 459-1500

E-mail address: hagymasi.krisztina@semmelweis.hu

**FIGURE AND TABLE LEGENDS**

**TABLE S1.** PRISMA checklist.

**TABLE S2.** Search key.

**SUPPLEMENTARY FILE S1.** Synthesis methods in detail.

**TABLE S3.** Baseline characteristics of included studies.

**TABLE S4.** Vitamin D status of patients in included studies.

**SUPPLEMENTARY FILE S7.** Risk of bias assessment (RoB-2 tool) about each risk of bias item for each included study.

**TABLE S6.** Adverse events reported in the included studies.

**TABLE S7.** Summary finding table of quality of evidence.

**TABLE S8.** Excluded studies based on the full-text selection.

**TABLE S1.** PRISMA checklist.

| **Section and Topic** | **Item #** | **Checklist item** | **Location where item is reported** |
| --- | --- | --- | --- |
| **TITLE** | | |  |
| Title | 1 | Identify the report as a systematic review. | Page 1 |
| **ABSTRACT** | | |  |
| Abstract | 2 | See the PRISMA 2020 for Abstracts checklist. | Abstract, Page 3 |
| **INTRODUCTION** | | |  |
| Rationale | 3 | Describe the rationale for the review in the context of existing knowledge. | Introduction, Page 8 |
| Objectives | 4 | Provide an explicit statement of the objective(s) or question(s) the review addresses. | Introduction, Page 8 |
| **METHODS** | | |  |
| Eligibility criteria | 5 | Specify the inclusion and exclusion criteria for the review and how studies were grouped for the syntheses. | Methods, Page 9 |
| Information sources | 6 | Specify all databases, registers, websites, organisations, reference lists and other sources searched or consulted to identify studies. Specify the date when each source was last searched or consulted. | Methods, Page 10 |
| Search strategy | 7 | Present the full search strategies for all databases, registers and websites, including any filters and limits used. | Methods, Page 10, Supplementary S2 |
| Selection process | 8 | Specify the methods used to decide whether a study met the inclusion criteria of the review, including how many reviewers screened each record and each report retrieved, whether they worked independently, and if applicable, details of automation tools used in the process. | Methods, Page 10-11 |
| Data collection process | 9 | Specify the methods used to collect data from reports, including how many reviewers collected data from each report, whether they worked independently, any processes for obtaining or confirming data from study investigators, and if applicable, details of automation tools used in the process. | Methods, Pages 10-11 |
| Data items | 10a | List and define all outcomes for which data were sought. Specify whether all results that were compatible with each outcome domain in each study were sought (e.g. for all measures, time points, analyses), and if not, the methods used to decide which results to collect. | Methods, Pages 9-10 |
|  | 10b | List and define all other variables for which data were sought (e.g. participant and intervention characteristics, funding sources). Describe any assumptions made about any missing or unclear information. | Methods, Pages 9-10  Supplementary file S1 |
| Study risk of bias assessment | 11 | Specify the methods used to assess risk of bias in the included studies, including details of the tool(s) used, how many reviewers assessed each study and whether they worked independently, and if applicable, details of automation tools used in the process. | Methods, Page 11-12 |
| Effect measures | 12 | Specify for each outcome the effect measure(s) (e.g. risk ratio, mean difference) used in the synthesis or presentation of results. | Methods, Pages 12  Supplementary file S1 |
| Synthesis methods | 13a | Describe the processes used to decide which studies were eligible for each synthesis (e.g. tabulating the study intervention characteristics and comparing against the planned groups for each synthesis (item #5)). | Supplementary file S1 |
|  | 13b | Describe any methods required to prepare the data for presentation or synthesis, such as handling of missing summary statistics, or data conversions. | Supplementary file S1 |
|  | 13c | Describe any methods used to tabulate or visually display results of individual studies and syntheses. | Supplementary file S1 |
|  | 13d | Describe any methods used to synthesize results and provide a rationale for the choice(s). If meta-analysis was performed, describe the model(s), method(s) to identify the presence and extent of statistical heterogeneity, and software package(s) used. | Supplementary file S1 |
|  | 13e | Describe any methods used to explore possible causes of heterogeneity among study results (e.g. subgroup analysis, meta-regression). | Supplementary file S1 |
|  | 13f | Describe any sensitivity analyses conducted to assess robustness of the synthesized results. | Supplementary file S1 |
| Reporting bias assessment | 14 | Describe any methods used to assess risk of bias due to missing results in a synthesis (arising from reporting biases). | Methods, Page 11-12, Supplementary file S1 |
| Certainty assessment | 15 | Describe any methods used to assess certainty (or confidence) in the body of evidence for an outcome. | Methods, Page 12 |
| **RESULTS** | | |  |
| Study selection | 16a | Describe the results of the search and selection process, from the number of records identified in the search to the number of studies included in the review, ideally using a flow diagram. | Results, Pages 12-13 |
|  | 16b | Cite studies that might appear to meet the inclusion criteria, but which were excluded, and explain why they were excluded. | Supplementary table S7 |
| Study characteristics | 17 | Cite each included study and present its characteristics. | Supplementary table S3 |
| Risk of bias in studies | 18 | Present assessments of risk of bias for each included study. | Supplementary file S7 |
| Results of individual studies | 19 | For all outcomes, present, for each study: (a) summary statistics for each group (where appropriate) and (b) an effect estimate and its precision (e.g. confidence/credible interval), ideally using structured tables or plots. | Results Pages 15-22, Supplementary file S2-S6 |
| Results of syntheses | 20a | For each synthesis, briefly summarise the characteristics and risk of bias among contributing studies. | Supplementary file S7 |
|  | 20b | Present results of all statistical syntheses conducted. If meta-analysis was done, present for each the summary estimate and its precision (e.g. confidence/credible interval) and measures of statistical heterogeneity. If comparing groups, describe the direction of the effect. | Results Pages 9-12, Supplementary file S2-S5 |
|  | 20c | Present results of all investigations of possible causes of heterogeneity among study results. | Results Pages 15-22, Supplementary file S2-S6 |
|  | 20d | Present results of all sensitivity analyses conducted to assess the robustness of the synthesized results. | Results Pages 15-22, Supplementary file S2-S6 |
| Reporting biases | 21 | Present assessments of risk of bias due to missing results (arising from reporting biases) for each synthesis assessed. | Supplementary file S7 |
| Certainty of evidence | 22 | Present assessments of certainty (or confidence) in the body of evidence for each outcome assessed. | Supplementary table S7 |
| **DISCUSSION** | | |  |
| Discussion | 23a | Provide a general interpretation of the results in the context of other evidence. | Discussion, Pages 24-27 |
|  | 23b | Discuss any limitations of the evidence included in the review. | Discussion, Page 27 |
|  | 23c | Discuss any limitations of the review processes used. | Discussion, Page 27 |
|  | 23d | Discuss implications of the results for practice, policy, and future research. | Discussion, Page 27-28 |
| **OTHER INFORMATION** | | |  |
| Registration and protocol | 24a | Provide registration information for the review, including register name and registration number, or state that the review was not registered. | Methods, Page 8 |
|  | 24b | Indicate where the review protocol can be accessed, or state that a protocol was not prepared. | Methods, Page 8 |
|  | 24c | Describe and explain any amendments to information provided at registration or in the protocol. | Methods, Page 8 |
| Support | 25 | Describe sources of financial or non-financial support for the review, and the role of the funders or sponsors in the review. | Page 3 |
| Competing interests | 26 | Declare any competing interests of review authors. | Page 3 |
| Availability of data, code and other materials | 27 | Report which of the following are publicly available and where they can be found: template data collection forms; data extracted from included studies; data used for all analyses; analytic code; any other materials used in the review. | Page 3, Methods Pages 8-9 |

From: Page MJ, McKenzie JE, Bossuyt PM, Boutron I, Hoffmann TC, Mulrow CD, et al. The PRISMA 2020 statement: an updated guideline for reporting systematic reviews. BMJ 2021;372:n71. doi: 10.1136/bmj.n71. For more information, visit: http://www.prisma-statement.org/

PRISMA: Preferred Reporting Items for Systematic Reviews and Meta-analyses.

**Table S2. Search key**

| Search key |
| --- |
| MEDLINE:  (vitamin d OR (vitamin AND d) OR cholecalciferol OR colecalciferol OR calcidiol OR calcitriol OR 25ohd OR 25-hydroxyvitamin OR 25hydroxycholecalciferol OR ergocalciferol OR 1,25-dihydroxyvitamin OR "vitamin D3") AND (liver OR liver diseases OR CLD OR cirrhosis OR steatosis OR steatohepatitis OR nash OR nafld OR nafl OR ald OR alcoh* OR pbc OR psc OR cholang* OR hemochromatosis OR chc OR hcv OR hbv OR hdv OR wilson OR antitrypsin OR hepat*) AND (random* or blind* or placebo or RCT) |
| COCHRANE:  (vitamin d OR (vitamin AND d) OR cholecalciferol OR colecalciferol OR calcidiol OR calcitriol OR 25ohd OR 25-hydroxyvitamin OR 25hydroxycholecalciferol OR ergocalciferol OR 1,25-dihydroxyvitamin OR "vitamin D3") AND (liver OR liver diseases OR CLD OR cirrhosis OR steatosis OR steatohepatitis OR nash OR nafld OR nafl OR ald OR alcoh* OR pbc OR psc OR cholang* OR hemochromatosis OR chc OR hcv OR hbv OR hdv OR wilson OR antitrypsin OR hepat*) AND (random* or blind* or placebo or RCT) in Title Abstract Keyword |
| EMBASE:  ('vitamin d'/exp OR 'vitamin d' OR (('vitamin'/exp OR vitamin) AND d) OR 'cholecalciferol'/exp OR cholecalciferol OR 'colecalciferol'/exp OR colecalciferol OR 'calcidiol'/exp OR calcidiol OR 'calcitriol'/exp OR calcitriol OR 25ohd OR '25 hydroxyvitamin' OR 25hydroxycholecalciferol OR 'ergocalciferol'/exp OR ergocalciferol OR '1,25 dihydroxyvitamin' OR 'vitamin d3'/exp OR 'vitamin d3') AND ('liver'/exp OR liver OR 'liver diseases'/exp OR 'liver diseases' OR (('liver'/exp OR liver) AND ('diseases'/exp OR diseases)) OR cld OR 'cirrhosis'/exp OR cirrhosis OR 'steatosis'/exp OR steatosis OR 'steatohepatitis'/exp OR steatohepatitis OR nash OR nafld OR nafl OR ald OR alcoh* OR pbc OR psc OR cholang* OR 'hemochromatosis'/exp OR hemochromatosis OR chc OR 'hcv'/exp OR hcv OR 'hbv'/exp OR hbv OR hdv OR wilson OR 'antitrypsin'/exp OR antitrypsin OR hepat*) AND (random* OR blind* OR 'placebo'/exp OR placebo OR rct) |

**Supplementary File S1.** Synthesis methods in detail.

**Synthesis methods in details**

As we assumed considerable between-study heterogeneity in all cases, a random-effects model was used to pool effect sizes. When there were two treatment or control arms, we combined (see the details later) them to create a single pair-wise comparison, as recommended by Cochrane Handbook.^1,2^

We calculated risk ratio (RR) with a 95% confidence interval (CI) for dichotomous variables and mean differences (MD) for continuous variables. To calculate the study MDs and the pooled MDs, the sample size, the mean, and the corresponding standard deviation (SD) were extracted or calculated from each study. To calculate the study RRs and the pooled RR, the total number of patients and those with the event of interest in each group separately was extracted or calculated from the studies.

The mean or the standard deviation was not reported in some studies, but the standard error of mean (SEM) or the 95% confidence interval of mean was given. In these cases, the SD was calculated as SEM multiplied by the square root of the sample size. Based on the confidence interval of mean, we calculated the SEM using t-distribution method. If instead of the mean, SD, SEM, or CI, the quartiles were given, for estimating the mean and standard deviation from the quartiles Luo and Shi methods were used (as implemented in the used meta R package).^3,4^ As a limitation, we should highlight that this is an estimation. Although, based on the other publications in the literature about these outcomes and the data in the used publications, we could assume that the distribution of these outcomes is not relevantly differ from a normal or log-normal distribution, therefore this estimation might give only a small bias. We labelled the studies where this estimation was used with β on the forest plots.

In a few articles,^5,6^ 2 separate interventions in 2 independent group of patients using 2 independent group of control population was reported. In our meta-analysis we calculated the sum of weighted mean and SD of these independent groups according to the Cochrane handbook.^1^ In other few articles,^7-9^ for 2 independent intervention groups, the same control group was used, therefore we calculated the sum of intervention as mentioned before, but we used the common control group as the “sum” of control groups. In case of Sharifi et al.,^10,11^ the quartiles were reported in 2 independent group (male, female): here we first estimated the mean and SD and thereafter we estimated the combined mean and SD.

As on the one hand, several studies reported the observed values before (baseline) and after the treatment, but the change was not reported, and on the other hand, several studies reported the change without reporting the baseline and after treatment values, the syntheses of all together were not possible without additional assumptions. Regarding this situation, we performed different analyses to estimate the effect and its significance. We used the given, calculated or estimated mean and SD values for the following analyses.

1. As we have RCT designs, we assumed that the baseline values are equal in the experimental and control group, therefore the effect could be expressed by using only the “directly given” (e.g., no assumption, estimation needed) after treatment values (for the articles where it is given).
2. To pool together all the study results, we estimated the mean and SD of change from baseline to after treatment where it is not reported. For this, we should estimate the correlation coefficient (Rest) between baseline and after values. The estimation of R was based on the extracted or calculated SD of change if the SD for baseline and after value was also given in the study - from where the correlation coefficient is calculable. The mean of the calculated R values was used as estimand of R for estimating change values. After estimating change mean and SD where it is not given (imputing the R as described), we pool together the directly given and estimated change values. Additionally, we made this estimation with different R values – as influential analyses - to see how it influences the pooled effect. If more study contains information for Rest, we calculated the mean of it. If no study contained this information, we used the value 0.5. We labelled with ** the studies on the forest plots where this estimation was used.

We used inverse variance weighting method for pooling MDs. To estimate the heterogeneity variance measure τ^2^, the restricted maximum-likelihood estimator with the Q profile method for confidence interval was applied.^12^ Pooled RR was calculated by the Mantel-Haenszel method^13,14^ with the Paule-Mandel method,^15^ recommended by Veroniki et al.^12^ and with Q profile for confidence interval. The exact Mantel-Haenszel method (without continuity correction) was used to handle zero (or “total”) cell counts as recommended.^16,17^

We used a Hartung-Knapp adjustment^18,19^ for CIs (if it is more conservative then the classical one, as recommended by Jackson et al.^20^ as hybrid method 2) and for prediction intervals.

In the case of subgroup (e.g., categorical moderator, as intervention type and categorized intervention time) analysis, we used a fixed effects “plural” model (aka. mixed-effects model). We assumed that all subgroups share a different τ^2^ value as we anticipate difference in the between-study heterogeneity in the subgroups and the study number is not too small in subgroups. If at least one of the subgroups contains less than 6 studies, we use an assumption of same τ at subgroups (recommended in Harrer et al.^21^).

In case of meta-regression (e.g., continuous moderator, as time of intervention) analysis, a linear relation was assumed between the MD and the moderator. A weighted least square method was used. The confidence interval and prediction interval estimate for the slope was based on t-distribution. A Wald-type p-value for the slope and the meta-regression coefficient of determination (R2∗) was also given.

The intervention type and intervention time was predefined as moderator in our PROSPERO.

To assess the difference between the subgroups, a Cochran’s Q test (an omnibus test) was used between subgroups.^21^ The null hypothesis was rejected on a 5% significance level.

Forest plots were used to graphically summarize the results. In case of zero (or “total”) cell counts, individual study RR with 95% CI was calculated by adding 0.5 as continuity correction (it was used only for visualization on forest plot). For meta-regression, bubble plots were used. The square or dot sizes on the plots refers to the weight in the random effects meta-analysis. The t-distribution based method used for CI of MD calculation of individual studies. Results were considered statistically significant if the pooled CI does not contain the null or one value for MDs and RR respectively. We summarized the findings related to the meta-analysis on forest plots. Additionally, to the between-study variance (τ^2^), between-study heterogeneity was described by Higgins & Thompson’s I^2^ statistics.^22^

Small study publication bias was assessed by visual inspection of Funnel-plots and calculating Egger’s test p-value for MD effect size.^23^ For dichotomous data, we used the Harbord modified test.^24^ We planned to assume possible small study bias if the p-value is less than 10%. Potential outlier publications were explored using different influence measures and plots following the recommendation of Harrer et al.^21^ As additional sensitivity and exploratory analysis, we performed the following calculations. We subsetted the articles based on ROB - excluding high risk of bias studies. Subgroup analyses were conducted considering the vitamin D level making two subgroups: if only vitamin D deficient patients were involved in the studies or not. Additional subgroup analyses were performed based on etiology.

All statistical analyses were made with R ^25^ (v4.3.0) using the meta ^26^ (v6.5.0) package for basic meta-analysis calculations and plots, and dmetar ^27^ (v0.0.9000) package for additional influential analysis calculations and plots. For meta-regression, package metafor ^28^ (v4.2.0) was also used.

**TABLE S3.** Basic characteristics of included studies.

|  | | | Vitamin D group | | | | Control group | | | |
| --- | --- | --- | --- | --- | --- | --- | --- | --- | --- | --- |
|  |  |  | *SOC + Vitamin D supplementation* | | | | *SOC with or without placebo* | | | |
| Author, year | Country  (n° of centers) | Chronic liver disease | Number of patients (female %) | Mean age (years) ± SD ‡ | Mean BMI (kg/m^2^) ± SD ‡ | Vitamin D intervention | Number of patients (female %) | Mean age (year) ± SD ‡ | Mean BMI (kg/m2) ± SD ‡ | Standard of care with or without placebo |
| Abu-Mouch et al. 2011 ^29^ | Israel (1) | CHC genotype 1 | 36 (50%) | 47 ±11 | 27 ± 4 | Vitamin D3 2000 IU/d p.o. 4 weeks before antiviral therapy until target VD level > 32 ng/mL or for 48 weeks with antivirals | 36 (40%) | 49 ± 7 | 24 ± 3 | SOC (PEG-IFN/RBV) |
| Afsordeh et al. 2019 ^6^ | Iran (1) | NAFLD | 10 (100%) | 30-45 ^b^ | 31 ± 7 | Vitamin D3 50 000 IU/w p.o. + Aerobic training for 8 weeks | 10 (100%) | 30-45 ^b^ | 32 ± 7 | SOC + Aerobic training |
|  |  |  | 10 (100%) | 30-45 ^b^ | 31 ± 8 | Vitamin D3 50 000 IU/w p.o. for 8 weeks | 10 (100%) | 30-45 ^b^ | 32 ± 2 | SOC |
| Alarfaj et al. 2023 ^30^ | Egypt (1) | NAFLD | 50 (52%) | 46 ± 17 | 26 ± 4 | Vitamin D3 300 000 IU p.o. loading dose followed by 800 IU/d p.o. for 4 months | 50 (48%) | 42 ± 14 | 27 ± 3 | SOC + Placebo |
| Atsukawa et al. 2016 ^31^ | Japan (12) | CHC genotype 1b | 57 (53%) | 63  (33-82) ^a^ | 23 (18-35) ^a^ | Vitamin D3 2000 IU/d p.o. for 4 weeks before antiviral therapy followed by 12 weeks with antiviral therapy | 58 (47%) | 64  (31-80) ^a^ | 22 (18-30) ^a^ | SOC (PEG-IFN/RBV) |
| Atthakitmongkol et al. 2023 ^32^ | Thailand (1) | CHB | 32 (NA) | NA | NA | Vitamin D2 + Calcium carbonate for 48 weeks | 32 (NA) | NA | NA | SOC (TDF) |
| Barchetta et al. 2016 ^33^ | Italy (1) | NAFLD | 26 (30%) | 57 ± 11 | 29 ± 4 | Vitamin D3 2000 IU/d p.o. for 24 weeks | 29 (40%) | 60 ± 9 | 31 ± 5 | SOC + Placebo |
| Behera et al. 2018 ^34^ | India (1) | CHC genotype 1, 4 | 28 (43%) | 41 ± 11 | 23 ± 2 | Vitamin D3 2000 IU/d p.o. for 48 weeks with antiviral therapy | 32 (37%) | 42 ± 12 | 23 ± 4 | SOC (PEG-IFN/RBV) |
| Boonyagard et al. 2020 ^35^ | Thailand (1) | NAFLD | 30 (63%) | 56 ± 8 | 28 ± 7 | Vitamin D2 40 000 IU/w p.o. for 5 months | 30 (40%) | 52 ± 11 | 28 ± 4 | SOC + Placebo |
| Dabbaghmanesh et al. 2018 ^7^ | Iran (1) | NAFLD | 35 (NA) | 45 ± 8 | 29 ± 5 | Vitamin D3 50 000 IU/w p.o. for 12 weeks | 36 (NA) | 46 ± 15 | 29 ± 5 | SOC + Placebo |
|  |  |  | 35 (NA) | 44 ± 11 | 30 ± 5 | Calcitriol 0.25 mg/d p.o. for 12 weeks |  |  |  |  |
| Ebrahimpour-Koujan et al. 2024 ^36^ | Iran (1) | NAFLD | 23 (53%) | 47 ± 9 | 30 ± 4 | Vitamin D3 4000 IU/d p.o. for 12 weeks | 23 (44%) | 44 ± 11 | 33 ± 11 | SOC + Placebo |
| Esmat et al. 2015 ^37^ | Egypt (1) | CHC genotype 4 | 50 (26%) | 40 ± 10 | 26 ± 4 | Vitamin D3 15 000 IU/w p.o. for 48 weeks | 51 (24%) | 40 ± 9 | 27 ± 3 | SOC (PEG-IFN/RBV) |
| Foroughi et al. 2014 ^38^ †  *Foroughi et al. 2016 ^39^* | Iran (1) | NAFLD | 30 (NA) | NA | 31 ± 4 | Vitamin D3 50 000 IU/w p.o. for 10 weeks | 30 (NA) | NA | 32 ± 6 | SOC + Placebo |
| Geier et al. 2018 ^40^ | Switzerland (3) | NAFLD | 10 (NA) | 39 ± 16 | 31 ± 4 | Vitamin D3 2100 IU/d p.o. for 48 weeks | 10 (NA) | 50 ± 13 | 30 ± 3 | SOC + Placebo |
| Grover et al. 2021 ^41^  *Grover et al. 2022 ^42^ †* | India (1) | Liver cirrhosis irrespective of etiology | 82 (27%)  In abstract reported 81 (NA) | 44 ± 12 | NA | Vitamin D3 60 000 IU/w loading dose for 2 months followed by 60 000 IU/m p.o. maintenance dose for 10 months + Calcium carbonate 1000 mg/d p.o. | 82 (18%)  In abstract reported 80 (NA) | 41 ± 12 | NA | SOC + Placebo |
| Guo et al. 2022 ^43^ | China (2) | NAFLD | 37 (46%) | 57 ± 16 | 26 ± 2 | Vitamin D3 1680 IU/d p.o. + Fish oil for 3 months | 37 (41%) | 55 ± 17 | 28 ± 4 | SOC +  Fish oil (identical appearance) |
| Hajiaghamohammadi et al. 2019 ^44^ | Iran (1) | NAFLD | 40 (68%) | 35 ±13 | 30 ± 2 | Vitamin D3 50 000 IU/w p.o. for 10 weeks | 40 (63%) | 40 ± 11 | 30 ± 2 | SOC |
| Harun et al. 2020 ^45^ | Bangladesh (1) | Liver cirrhosis irrespective of etiology | 36 (17%) | 35 ± 11 | NA | Vitamin D3 50 000 IU/w p.o. loading dose for 7 weeks followed by 2000 IU/d p.o. maintenance dose + Calcium 1000 mg/d p.o. for 6 months | 35 (20%) | 40 ± 10 | NA | SOC |
| Hoseini et al. 2020 ^5^ | Iran (1) | NAFLD | 10 (100%) | 63 ± 2 | 36 ± 4 | Vitamin D3 50 000 IU/w p.o. + Aerobic training for 2 months | 10 (100%) | 63 ± 2 | 34 ± 2 | SOC + Aerobic training + Placebo |
|  |  |  | 10 (100%) | 61 ± 1 | 34 ± 2 | Vitamin D3 50 000 IU/w p.o. for 2 months | 10 (100%) | 62 ± 2 | 35 ± 2 | SOC + Placebo |
| Hosseini et al. 2018 ^46^ | Iran (1) | NAFLD | 37 (100%) | 34 ± 7 | 35 ± 5 | Vitamin D3 60 000 IU single dose i.m. + Vitamin E 400 IU/d p.o. for 1 month | 38 (100%) | 34 ± 7 | 33 ± 4 | SOC + Vitamin E 400 IU/d p.o |
| Hussain et al. 2019 ^47^ | Pakistan (1) | NAFLD | 54 (37%) | 27 ± 2 | 29 ± 2 | Vitamin D3 50 000 IU/w p.o. for 12 weeks | 55 (35%) | 29 ± 19 | 29 ± 1 | SOC + Placebo |
| Jeong et al. 2020 ^48^ | Korea (11) | CHC genotype 1, 2, 3 | 77 (46%) | 52 ± 9 | 25 ± 3 | Vitamin D3 800 IU/d p.o. for 24 weeks (genotype 2,3) or 48 weeks (genotype 1,4) | 71 (58%) | 52 ± 10 | 24 ± 4 | SOC (PEG-IFN/RBV) |
| Jha et al. 2017 ^49^ | India (1) | Liver cirrhosis irrespective of etiology | 51 (22%) | 46 ± 15 | NA | Vitamin D3 300 000 IU single dose i.m. followed by 800 IU/d p.o. maintenance dose + Calcium 1000 mg/d p.o. for 6 months | 50 (26%) | 43 ± 13 | NA | SOC |
| Khan et al. 2022 ^50^ | Pakistan (1) | CHC genotype 1, 2, 3, 4 | 50 (40%) | 39 ± 13 | NA | Form and schedule of vitamin D supplementation for 6 months not specified | 50 (32%) | 37 ± 14 | NA | SOC (ribavirin and sofosbuvir) |
| Komolmit et al. 2017 ^51^ | Thailand (1) | CHC irrespective of genotype | 40 (43%) | 53 ± 9 | 25 ± 3 | Vitamin D2 60 000 – 100 000 IU/w p.o. for 6 weeks | 40 (50%) | 52 ± 11 | 25 ± 4 | SOC + Placebo |
| Komolmit et al. 2017 ^52^ | Thailand (1) | CHC irrespective of genotype | 29 (28%) | 50 ± 12 | 25 ± 4 | Vitamin D2 60 000 -100 000 IU/w p.o. for 6 weeks | 29 (48%) | 50 ± 10 | 25 ± 3 | SOC + Placebo |
| Lorvand Amiri et al. 2016 ^53^  *Lorvand Amiri et al. 2017 ^9^*  *Shidfar et al. 2019 ^54^* | Iran (1) | NAFLD | 37 (38%) | 38 ± 10 | 31 ± 1 | Calcitriol 25 μg/d p.o. + Calcium carbonate 500 mg/d p.o. for 12 weeks | 36 (35%) | 44 ± 11 | 31 ± 1 | SOC + Placebo for Vitamin D and Calcium |
|  |  |  | 37 (41%) | 40 ± 11 | 30 ± 1 | Calcitriol 25 μg/d p.o. + Placebo for calcium for 12 weeks |  |  |  |  |
| Lukenda Zanko et al. 2020 ^55^ | Croatia (1) | NAFLD | 201 (42%) | 64  (20-85) ^c^ | 31 (22-56) ^c^ | Vitamin D3 1000 IU/d p.o. for 12 months | 110 (44%) | 66  (23-83) ^c^ | 31 (20-45) ^c^ | SOC + Placebo |
| Mihai et al. 2014 ^56^ | Romania (1) | CHC | 24 (NA) | NA | NA | Vitamin D3 1000 IU/d p.o. | 20 (NA) | NA | NA | SOC (PEG-IFN/RBV) |
| Mobarhan et al. 1984 ^8^ | USA (1) | Liver cirrhosis due to  AALD | 6 (0%) | 55 ± 3 | NA | Vitamin D2 100 000 -150 000 IU/w p.o. for 6-12 months | 6 (0%) | 48 ± 9 | NA | SOC |
|  |  |  | 6 (0%) | 53 ± 5 | NA | Calcidiol  20-50 μg/d p.o. for 6-12 months |  |  |  |  |
| Mohamed et al. 2021 ^57^ | Egypt (1) | Liver cirrhosis irrespective of etiology | 160 (47%) | 51 ± 12 | 26 ± 2 | Vitamin D3 300 000 IU single dose i.m. loading dose followed by 800 IU/d p.o. maintenance dose + Calcium 1000 mg/d p.o. for 6 months | 168 (51%) | 55 ± 9 | 26 ± 2 | SOC |
| Mohamed et al. 2023 ^58^ | Egypt (1) | NAFLD | 70 (59%) | 52 ± 10 | 32 ± 8 | Vitamin D3 200 000 IU p.o. loading dose followed by 800 IU/d p.o. for 4 months | 70 (64%) | 54 ± 10 | 31 ± 5 | SOC + Placebo |
| Nimer et al. 2012 ^59^ | Israel (2) | CHC genotype 2,3 | 20 (35%) | 48 ± 14 | 30 ± 6 | Vitamin D3 2000 IU/d p.o. 12 weeks before antiviral therapy until target 25OHD level > 32 ng/mL | 30 (40%) | 45 ± 10 | 26 ± 3 | SOC (PEG-IFN/RBV) |
| Okubo et al. 2021 ^60^ | Japan (1) | Liver cirrhosis irrespective of etiology | 15 (60%) | 73  (64-86) ^c^ | 22 (18-27) ^c^ | Vitamin D3 2000 IU/d p.o. + BCAA for 12 months | 17 (59%) | 70  (55-88) ^c^ | 24 (15-32) ^c^ | SOC + BCAA |
| Pilz et al. 2016 ^61^ | Austria (2) | Liver cirrhosis irrespective of etiology | 18 (33%) | 63 ± 9 | 28 ± 5 | Vitamin D3 2800 IU/d p.o. for 8 weeks | 18 (17%) | 58 ± 9 | 26 ± 3 | SOC + Placebo |
| Sakpal et al. 2017 ^62^ | India (1) | NAFLD | 51 (30%) | 37 ± 10 | 28 ± 6 | Vitamin D3 600 000 IU single dose i.m. | 30 (37%) | 40 ± 10 | 27 ± 4 | SOC |
| Sharifi et al. 2014 ^10^  *Sharifi et al. 2016 ^11^* | Iran (1) | NAFLD | 27 (52%) | 40 ± 9 | 31  (29, 33) ^a^ | Vitamin D3 50 000 IU twice per month p.o. for 4 months | 26 (50%) | 44 ± 10 | 29  (27, 32) ^a^ | SOC + Placebo |
| Shiomi et al. 1999 ^63^ | Japan (1) | CHB and CHC irrespective of genotype | 38 (66%) | *M:*  62 ± 11  *F:*  59 ± 9 | NA | Calcitriol 1 μg/d p.o. divided in two doses for at least 12 months | 38 (66%) | *M:*  62 ± 9  *F:*  62 ± 7 | NA | SOC |
| Shiomi et al. 1999 ^64^ | Japan (1) | PBC | 17 (100%) | 55 ± 10 | NA | Calcitriol 1 μg/d p.o. divided in two doses for at least 12 months | 17 (100%) | 57 ± 7 | NA | SOC |
| Sriphoosanaphan et al. 2021 ^65^ | Thailand (1) | CHC genotype 1,3,6 | 37 (57%) | 61 ± 8 | 25 ± 4 | Vitamin D2 60 000 – 100 000 IU/w p.o. for 6 weeks | 38 (63%) | 59 ± 8 | 24 ± 4 | SOC + Placebo |
| Taghvaei et al. 2018 ^66^ | Iran (1) | NAFLD | 20 (50%) | 41 ± 14 | 29 ± 3 | Vitamin D3 50 000 IU/w p.o. for 12 weeks | 20 (50%) | 44 ± 11 | 29 ± 4 | SOC |
| Vosoghinia et al. 2016 ^67^ | Iran (1) | CHC genotype 1,2,3,4 | 34 (12%) | 42 ± 10 | 24 ± 3 | Vitamin D3 50 000 IU/w p.o. before antiviral therapy in those with VD level < 30 ng/mL until level > 30 ng/mL, followed by 50 000 IU/m p.o. for 24 weeks (genotype 2,3) or 48 weeks (genotype 1,4) | 34 (10%) | 42 ± 12 | 26 ± 6 | SOC (PEG-IFN/RBV) |
| Wang et al. 2020 ^68^ † | Taiwan (1) | CHB | 75 (57%) | 52 ± 11 | NA | Vitamin D3 2000 IU/d p.o. for 2 months | 74 (54%) | 51 ± 9 | NA | SOC |
| Xing et al. 2013 ^69^ | China (1) | Liver transplant recipients | 25 (24%) | 49 *median* | 22  *median* | Calcitriol 0.25 μg/d p.o. + Calcium gluconate i.v. for 1 week | 25 (12%) | 48 *median* | 21  *median* | SOC + Placebo + Calcium gluconate i.v. |
| Yaghooti et al. 2021 ^70^ | Iran (1) | NAFLD | 67 (NA) | 40  *mean* | 29 ± 5 | Calcitriol 0.25 μg/d p.o. for 17 weeks | 67 (NA) | 40  *mean* | 29 ± 4 | SOC + Placebo |
| Yang et al. 2023 ^71^ | China (1) | CHB | 86 (26%) | 61 ± 11 | NA | Vitamin D3 800 IU/d p.o. for 6 months | 86 (22%) | 58 ± 10 | NA | SOC (TAF, TDF, ETV, etc.) |
| Yokoyama et al. 2014 ^72^ | Japan (14) | CHC genotype 1b | 42 (52%) | 60  (30-78) ^c^ | 22 (18-28) ^c^ | Vitamin D3 1000 IU/d p.o. started after 8 weeks of antiviral therapy in patients who achieved RVR and lasted 16 weeks | 42 (45%) | 59  (36-70) ^c^ | 23 (19-32) ^c^ | SOC (PEG-IFN/RBV) |

**‡ parameters represented as mean with standard deviation, or median with range (minimum and maximum)**

† study included only in systematic review

^a^ Data are expressed as median (25^th^, 75^th^ percentiles)

^b^ Data are expressed as min-max range

^c^ Data are expressed as median (min-max range)

AALD: Alcohol-associated liver disease; BCAA: Branched-chain amino acids; CHC: Chronic hepatitis C; CHB: Chronic hepatitis B; ETV: Entecavir; F: Female; i.v.: intravenous; NAFLD: Non-alcoholic fatty liver disease; NA: Not available; M: Male; PEG-IFN/RBV: pegylated interferon and ribavirin; PBC: Primary biliary cholangitis; SOC: Standard of care; p.o.: Per os; RVR: Rapid virological response; TAF: Tenofovir alafenamide fumarate; TDF: Tenofovir disoproxil fumarate; VD: Vitamin D

**TABLE S4.** Vitamin D status of patients in included studies.

| Author, year | Study period | Only vitamin D deficient/insufficient (< 30 ng/mL) patients included | Baseline vitamin D level, ng/mL  (Mean ± SD) | |
| --- | --- | --- | --- | --- |
|  |  |  | VD-supplemented patients | Control group |
| Abu-Mouch et al. 2011 ^29^ | NA | No, 20% had sufficient VD levels (> 32 ng/mL) | 20.5 ± 9 | 19 ± 6 |
| Afsordeh et al. 2019 ^6^ | NA | NA | NA | NA |
| Alarfaj et al. 2023 ^30^ | 10/2022 to 02/2023 | Yes, all patients had subnormal VD levels (< 30 ng/mL) | 18.4 ± 4.9 | 18.6 ± 5.3 |
| Atsukawa et al. 2016 ^31^ | 12/2013 to 05/2014 | Unclear, 22 patients had VD levels < 21 ng/mL and 36 patients had VD levels ≥ 21 ng/mL | 23 (8-48) ^a^ | 22 (7-43) ^a^ |
| Atthakitmongkol et al. 2023 ^32^ | NA | No, 10 VD-supplemented patients and seven control patients had VD levels > 30 ng/mL | 26 ± 9.6 | 24.6 ± 8 |
| Barchetta et al. 2016 ^33^ | 03/2012 to 09/2014 | No, four patients had VD levels > 30 ng/mL | 19.3 ± 9.5 ^b^ | 16.1 ± 10 ^b^ |
| Behera et al. 2018 ^34^ | 09/2012 to 01/2015 | Unclear, 33 patients had VD levels < 20 ng/mL and 27 patients had VD levels ≥ 20 ng/mL | 20.9 ± 10.3 | 23.4 ± 12.5 |
| Boonyagard et al. 2020 ^35^ | 01/2015 to 12/2018 | Yes, inclusion criterium was VD level < 30 ng/mL | 19.6 ± 5.8 | 21.3 ± 5.5 |
| Dabbaghmanesh et al. 2018 ^7^ | 04/2011 to 10/2013 | Yes, all included patients had subnormal VD levels (< 30 ng/mL) | *Vitamin D3:* 18.9 ± 6.2  *Calcitriol:*  18.6 ± 5.5 | 21.1 ± 5.2 |
| Ebrahimpour-Koujan et al. 2024 ^36^ | 10/2018 to 04/2019 | No, 13 VD-supplemented patients and 11 control patients had VD levels > 30 ng/mL | 31.7 ± 10.3 | 31.4 ± 8.4 |
| Esmat et al. 2015 ^37^ | NA | No, eight patients had VD level > 30 ng/mL | 11 ± 16 | 6 ± 4.6 |
| Foroughi et al. 2014 ^38^  *Foroughi et al. 2016 ^39^* | NA | NA | 19.6 ± 0.4 ^b^ | 18.8 ± 0.8 ^b^ |
| Geier et al. 2018 ^40^ | 2011 to 2016 | Yes, inclusion criterium was VD level < 30 ng/mL | 21 ± 12 | 20 ± 15 |
| Grover et al. 2021 ^41^  *Grover et al. 2022 ^42^* | 02/2015 to 03/2018 | Unclear, 137 patients had VD levels ≤ 20 ng/mL and 27 patients had VD levels > 20 ng/mL | 10.4 (6.5-16.3) ^a^ | 11.2 (7.1-16.3) ^a^ |
| Guo et al. 2022 ^43^ | 08/2019 to 01/2021 | No, all patients had VD level > 30 ng/mL | 35.2 ± 17.8 ^b^ | 38.9 ± 26.7 ^b^ |
| Hajiaghamohammadi et al. 2019 ^44^ | NA | Yes, exclusion criterium was VD level > 30 ng/mL | 15.2 ± 4.7 | 14.3 ± 4.7 |
| Harun et al. 2020 ^45^ | NA | Yes, all patients had VD level < 20 ng/mL | 9.7 (8.6-10.7) ^c^ | 9.2 (8.4-9.9) ^c^ |
| Hoseini et al. 2020 ^5^ | NA | Yes, patients were excluded if they did not have VD insufficiency (< 30 ng/mL) | *AT+VD:* 23 ± 4.4  *VD:*  22.9 ± 4.3 | *AT + placebo:*  25.2 ± 4.8  *Placebo only:*  24 ± 4.4 |
| Hosseini et al. 2018 ^46^ | 10/2015 to 03/2016 | Yes, inclusion criterium was VD level < 30 ng/mL | 13.8 ± 7.7 | 11.3 ± 6.7 |
| Hussain et al. 2019 ^47^ | NA | Yes, 13 screened patients were excluded due to normal VD level (threshold not specified) | 12.5 ± 4.2 | 15.4 ± 2.8 |
| Jeong et al. 2020 ^48^ | 09/2011 to 04/2015 | Unclear, 18 VD-supplemented patients and 17 control patients had VD levels ≥ 20 ng/mL | 15.5 ± 8.1 | 15.6 ± 8.7 |
| Jha et al. 2017 ^49^ | NA | Yes, inclusion criterium was VD level < 20 ng/mL | 9.7 (8.6-10.7) ^c^ | 9.2 (8.4-9.9) ^c^ |
| Khan et al. 2022 ^50^ | 11/2017 to 04/2018 | NA | NA | NA |
| Komolmit et al. 2017 ^51^ | 04/2013 to 04/2014 | Yes, inclusion criterium was VD level < 30 ng/mL | 20.9 ± 5.4 | 20.3 ± 4.8 |
| Komolmit et al. 2017 ^52^ | 02/2014 to 12/2014 | Yes, inclusion criterium was VD level < 30 ng/mL | 19.9 ± 5.3 | 19 ± 5.4 |
| Lorvand Amiri et al. 2016 ^53^  *Lorvand Amiri et al. 2017 ^9^*  *Shidfar et al. 2019 ^54^* | 06/2015 to 03/2016 | Yes, inclusion criterium was VD level < 15 ng/mL | *VD + calcium:*  9.9 ± 0.64  *VD:*  9.9 ± 0.64 | 10 ± 0.63 |
| Lukenda Zanko et al. 2020 ^55^ | 01/2015 to 03/2019 | No, 30 VD-supplemented patients and 16 control patients had VD levels ≥ 100 ng/mL | 23.7 (4.9-380.4) ^a, b^ | 18.9 (3.2-242.4) ^a, b^ |
| Mobarhan et al. 1984 ^8^ | NA | Yes, only patients with VD level < 20 ng/mL were included | *Vitamin D2:*  6 ± 6  *Calcidiol:*  9 ± 6 | 6 ± 6 |
| Mohamed et al. 2021 ^57^ | 06/2019 to 12/2019 | Yes, only patients with VD level < 25 ng/mL were included | 22.5 ± 11.5 | 20.2 ± 11 |
| Mohamed et al. 2023 ^58^ | 03/2022 to 08/2022 | No, 13 VD-supplemented and 15 control patients had VD level > 30 ng/mL | 15 ± 1.2 | 17 ± 1.4 |
| Nimer et al. 2012 ^59^ | NA | No, 20% had sufficient VD levels (> 32 ng/mL) | 20 ± 8 | 19 ± 6 |
| Okubo et al. 2021 ^60^ | 03/2017 to 03/2019 | Yes, exclusion criterium was VD level ≥ 30 ng/mL | 13.2 (6.1-19.2) ^a^ | 15 (5.4-25.5) ^a^ |
| Pilz et al. 2016 ^61^ | 12/2013 to 05/2014 | Yes, inclusion criterium was VD level < 30 ng/mL | 15.9 ± 7.5 | 15.5 ± 7 |
| Sakpal et al. 2017 ^62^ | NA | Yes, only patients with VD level < 32 ng/mL were included | 12.8 ± 6.4 | 12.3 ± 4.8 |
| Sharifi et al. 2014 ^10^  *Sharifi et al. 2016 ^11^* | 11/2012 to 09/2013 | No, three VD-supplemented and six control patients had VD level > 30 ng/mL | 11.5 (8.8, 28.4) ^d^ | 16.9 (11.7, 24.8) ^d^ |
| Shiomi et al. 1999 ^63^ | 1992 to 1996 | NA | *Men:*  14 (11, 19) ^d^  *Women:*  10 (8, 17) ^d^ | *Men:*  11 (6, 14) ^d^  *Women:*  12 (10, 16) ^d^ |
| Shiomi et al. 1999 ^64^ | NA | NA | 11 (8, 17) ^d^ | 12 (6, 14) ^d^ |
| Sriphoosanaphan et al. 2021 ^65^ | 02/2018 to 08/2018 | Yes, inclusion criterium was VD level < 30 ng/mL | 17.2 ± 4.8 | 16.6 ± 4.1 |
| Taghvaei et al. 2018 ^66^ | NA | Yes, inclusion criterium was VD level < 30 ng/mL | 19.2 ± 5.5 | 19.8 ± 4.4 |
| Vosoghinia et al. 2016 ^67^ | 02/2012 to 04/2015 | No | 22 ± 15 | 23 ± 13 |
| Wang et al. 2020 ^68^ | 08/2017 to 09/2018 | Yes, exclusion criterium was VD level ≥ 30 ng/mL | 18.9 ± 6.2 | 18.3 ± 6 |
| Xing et al. 2013 ^69^ | 03/2010 to 03/2011 | Unclear, 37 patients had VD level > 12.5 ng/mL | 12.5 ± 3 | 12.5 ± 3 |
| Yaghooti et al. 2021 ^70^ | 2017 to 2018 | Yes, all included patients were VD deficient (threshold not specified) | 19.1 ± 9.6 | 17.5 ± 7.7 |
| Yang et al. 2023 ^71^ | 01/2021 to 12/2021 | Yes, all patients had varying degrees of VD deficiency < 20 ng/mL | 15.8 ± 3.6 | 15.3 ± 3.6 |
| Yokoyama et al. 2014 ^72^ | NA | No | 22 (12-29) ^a^ | 25 (14-40) ^a^ |

^a^ Data are expressed as median (range min-max)

^b^ Units were converted from nmol/L to ng/mL

^c^ Data are expressed as mean (confidence intervals)

^d^ Data are expressed as median (25^th^, 75^th^ percentiles)

AT: Aerobic training; NA: Not available; VD: Vitamin D

**TABLE S6.** Adverse events reported in the included studies.

| **Author and year** | **Adverse events** | **Details** |
| --- | --- | --- |
| Abu-Mouch et al. 2011 ^29^ | Mild adverse events, similar in both groups, consistent with typical antiviral-induced symptoms. | Nausea (n=4), headache (n=4), insomnia (n=5), chills (n=4), myalgia (n=3), pyrexia (n=3), pruritus (n=2), mild neutropenia (n=3), mild thrombocytopenia (n=5), mild anemia (n=3) |
| Afsordeh et al. 2019 ^6^ | NA | NA |
| Alarfaj et al. 2023 ^30^ | None of the patients in vitamin D group reported side effects. | NA |
| Atsukawa et al. 2016 ^31^ | No patients complained of vitamin D related symptoms or developed signs of vitamin D related adverse reaction such as hypercalcemia. | Vitamin D3: malaise (n=3), epilepsy (n=1), hemorrhagic gastric ulcer (n=1), poor response (n=1)  Control: anemia (n=1), malaise (n=1), UTI (n=1), poor response (n=1), viral breakthrough (n=3) |
| Atthakitmongkol et al. 2023 ^32^ | NA | NA |
| Barchetta et al. 2016 ^33^ | No major adverse events occurred | Vitamin D3: mild glossitis (n=1) |
| Behera et al. 2018 ^34^ | None of the patients had stopped treatment due to adverse events. | NA |
| Boonyagard et al. 2020 ^35^ | At the end of this study, there was any adverse events including hypervitaminosis D or hypercalcemia. | NA |
| Dabbaghmanesh et al. 2018 ^7^ | Not specified: 7 patients had not followed the study protocol or had discontinued drugs due to side effects. | NA |
| Ebrahimpour-Koujan et al. 2024 ^36^ | Participants did not report any adverse events or symptoms related to vitamin D supplementation. | NA |
| Esmat et al. 2015 ^37^ | None of the missed patients had stopped the treatment due to adverse events. | NA |
| Foroughi et al. 2014 ^38^  Foroughi et al. 2016 ^39^ | Compliance with the treatments was good in both groups and no side‑effects were presented. | NA |
| Geier et al. 2018 ^40^ | Treatment with 2100 IU vitamin D over 48 weeks was well tolerated. All observed adverse events, including two serious ones, were rated as unrelated or unlikely related to study medication. | Vitamin D3: depression (n=1), lower back pain (n=1), GERD (n=1), abdominal menstrual cramps (n=1), tubular colon adenoma (n=1), gastric motility disturbance (n=1), irritable bowel syndrome (n=2), knee pain (n=1), circular hemorrhoidal prolapse (n=1)  Control: abdominal bloating (n=1), cold (n=1), constipation (n=1), sore throat (n=1), sour taste in mouth (n=1), contused lacerated wound (n=1), multiple white matter lesions (n=1), bronchopneumonia (n=1) |
| Grover et al. 2021 ^41^  Grover et al. 2022 ^42^ | No serious adverse events were observed or attributed that require discontinuation of treatments. Symptomatic fractures did not occur in any participant during the study duration. | Vitamin D3: asymptomatic nephrolithiasis (n=2)  Control: asymptomatic nephrolithiasis (n=1) |
| Guo et al. 2022 ^43^ | Dropouts from the treatment arms were not associated with adverse events. | NA |
| Hajiaghamohammadi et al. 2019 ^44^ | NA | NA |
| Harun et al. 2020 ^45^ | NA | NA |
| Hoseini et al. 2020 ^5^ | NA | NA |
| Hosseini et al. 2018 ^46^ | No side effects were reported. | NA |
| Hussain et al. 2019 ^47^ | Overall safety and tolerability profile of vitamin D supplementation was good. | Vitamin D3: severe allergy (n= 2) |
| Jeong et al. 2020 ^48^ | A total of 115 patients (77.7%) experienced at least one adverse event, 52 (73.2%) of them in the control group and 63 (81.8%) in the vitamin D group. Most adverse events were mild and similar in both the groups. Serious adverse events occurred in 10 patients, but all recovered. Dropout rate was similar in both groups (vitamin D: 10.4% vs. control: 19.7%, p=0.111). There was no adverse event related to vitamin D supplements. | NA |
| Jha et al. 2017 ^49^ | NA | NA |
| Khan et al. 2022 ^50^ | NA | NA |
| Komolmit et al. 2017 ^51^ | No adverse events related to vitamin D or placebo supplement were reported in all patients during study period. | NA |
| Komolmit et al. 2017 ^52^ | During the 6-week follow-up period, no adverse events were reported. | NA |
| Lorvand Amiri et al. 2016 ^53^  Lorvand Amiri et al. 2017 ^9^  Shidfar et al. 2019 ^54^ | NA | NA |
| Lukenda Zanko et al. 2020 ^55^ | No episodes of hypercalcemia or hypercalciuria were observed in either treatment arm. | NA |
| Mobarhan et al. 1984 ^8^ | NA | Vitamin D2: death due to cardiac arrest unrelated to vitamin D (n=1), mild transient hypercalcemia (n=2)  Calcidiol: myocardial infarction (n=1), death due to progressive liver failure (n=1) |
| Mohamed et al. 2021 ^57^ | NA | NA |
| Mohamed et al. 2023 ^58^ | None of the VD-supplemented patients reported any side effects after 4 months of daily cholecalciferol administration. | NA |
| Nimer et al. 2012 ^59^ | The most common adverse events were mild, similar in both groups, and consistent with typical PEG-INF/RBV induced systemic symptoms including nausea, headache, insomnia, myalgia, pyrexia, mild neutropenia, thrombocytopenia, and anemia. No serious adverse events were seen. | NA |
| Okubo et al. 2021 ^60^ | The patients demonstrated no symptoms due to hypercalcemia, such as anorexia, diarrhea, constipation, nausea, vomiting, sleepiness, headache, muscle pain, thirstiness, weakness, and renal calculi. | Vitamin D3: 1 patient died of liver failure at 6 months due to hepatorenal syndrome type 1 caused by cholecystitis. It was not considered to be related to vitamin D administration. |
| Pilz et al. 2016 ^61^ | No patient died during the study and there was no excess of adverse events in the vitamin D group. | NA |
| Sakpal et al. 2017 ^62^ | NA | NA |
| Sharifi et al. 2014 ^10^  Sharifi et al. 2016 ^11^ | Participants did not report any adverse or side effects such as hypercalcemia. | NA |
| Shiomi et al. 1999 ^63^ | NA | Calcitriol: Asymptomatic hypercalcemia (n=2) |
| Shiomi et al. 1999 ^64^ | NA | NA |
| Sriphoosanaphan et al. 2021 ^65^ | NA | NA |
| Taghvaei et al. 2018 ^66^ | NA | NA |
| Vosoghinia et al. 2016 ^67^ | NA | Control: 1 patient died due to myocardial infarction; 1 patient discontinued treatment due to thyroiditis |
| Wang et al. 2020 ^68^ | The frequency of adverse events was 4% (3/75) in vitamin D group and 8.1% (6/74) in control group. No serious adverse events or death was reported in this trial. | Vitamin D3: urticaria, constipation, microscopic hematuria; 1 patient in vitamin D group dropped out due to hematuria |
| Xing et al. 2013 ^69^ | NA | NA |
| Yaghooti et al. 2021 ^70^ | NA | NA |
| Yang et al. 2023 ^71^ | NA | NA |
| Yokoyama et al. 2014 ^72^ | The adverse effects in both groups were those common to PEG-INF/RBV treatment, including fever, headache, malaise, nausea, insomnia, myalgia, anemia, neutropenia, thrombocytopenia. No serious adverse side effects were observed. There were no adverse effects associated with vitamin D supplementation. | Vitamin D3: general fatigue (n=1)  Control: anxiety neurosis (n=1), death due to lung cancer (n=1) |

GERD: gastroesophageal reflux disease; IU: international unit; NA: not available; PEG-INF/RBV: pegylated interferon plus ribavirin; VD: Vitamin D; UTI: urinary tract infection

**TABLE S7.** Summary finding table of quality of evidence.

| **Summary of findings:** | | | | | | |
| --- | --- | --- | --- | --- | --- | --- |
| **Standard of care + Vitamin D compared to Standard of care ± placebo in chronic liver disease** | | | | | | |
| **Patient or population:** chronic liver disease  **Setting:** Randomized controlled trials  **Intervention:** Standard of care + Vitamin D  **Comparison:** Standard of care ± placebo | | | | | | |
| Outcome № of participants (studies) | Relative effect (95% CI) | **Anticipated absolute effects (95% CI)** | | | Certainty | What happens |
|  |  | **Standard of care ± placebo** | **Standard of care + Vitamin D** | **Difference** |  |  |
| Survival assessed with: RR follow-up: range 6 months to 12 months № of participants: 864 (8 RCTs) | **RR 1.05** (0.92 to 1.20) | 66.0% | **69.3%** (60.7 to 79.2) | **3.3% more** (5.3 fewer to 13.2 more) | ⨁◯◯◯ Very low ^a,b,c^ | The evidence is very uncertain about the effect of Vitamin D on survival. |
| Controlled attenuation parameter (CAP) follow-up: range 3 months to 12 months № of participants: 411 (3 RCTs) | - |  | - | MD **23.5 dB/m lower** (81.72 lower to 34.72 higher) | ⨁◯◯◯ Very low ^b,d^ | Vitamin D may result in little to no difference in controlled attenuation parameter. This result should be interpreted cautiously due to small number of RCTs and patients investigated. |
| Liver stiffness measurement (LSM) follow-up: range 3 months to 12 months № of participants: 411 (3 RCTs) | - |  | - | MD **0.65 kPa lower** (1.98 lower to 0.68 higher) | ⨁◯◯◯ Very low ^b,e^ | Vitamin D probably results in little to no difference in liver stiffness measurement. This result should be interpreted cautiously due to small number of RCTs and patients investigated. |
| Alanine aminotransferase (ALT) assessed with: IU/L № of participants: 2189 (24 RCTs) | - |  | - | MD **4.98 IU/L lower** (8.24 lower to 1.68 lower) | ⨁◯◯◯ Very low ^a,b,d,f^ | The evidence is very uncertain about the effect of Vitamin D on ALT. |
| Aspartate aminotransferase (AST) assessed with: IU/l № of participants: 2105 (23 RCTs) | - |  | - | MD **3.33 IU/L lower** (6.25 lower to 0.4 higher) | ⨁◯◯◯ Very low ^a,b,c^ | The evidence is very uncertain about the effect of Vitamin D on AST. |
| Gamma-glutamyl transferase (GGT) assessed with: IU/L № of participants: 1278 (11 RCTs) | - |  | - | MD **5.14 IU/L lower** (6.4 lower to 3.88 lower) | ⨁◯◯◯ Very low ^a,b,d,e,g^ | The evidence is very uncertain about the effect of Vitamin D on GGT. |
| Alkaline phosphatase (ALP) assessed with: IU/L № of participants: 618 (9 RCTs) | - |  | - | MD **7.53 IU/L lower** (15.77 lower to 0.72 higher) | ⨁◯◯◯ Very low ^a,b,d,g^ | The evidence is very uncertain about the effect of Vitamin D on ALP. |
| Homeostasis model assessment of insulin resistance (HOMA-IR) № of participants: 1446 (15 RCTs) | - |  | - | MD **0.31 lower** (0.62 lower to 0.01 lower) | ⨁◯◯◯ Very low ^a,b,g^ | The evidence is very uncertain about the effect of Vitamin D on HOMA-IR. |
| Fasting insulin level assessed with: uIU/mL № of participants: 1112 (11 RCTs) | - |  | - | MD **0.79 uIU/mL lower** (1.36 lower to 0.21 lower) | ⨁◯◯◯ Very low ^a,g^ | The evidence suggests that Vitamin D results in little to no difference in fasting insulin level. |
| Fasting plasma glucose (FPG) assessed with: mg/dL № of participants: 1378 (14 RCTs) | - |  | - | MD **1.69 mg/dL lower** (5.85 lower to 2.46 higher) | ⨁◯◯◯ Very low ^a,b,g^ | The evidence is very uncertain about the effect Vitamin D on FPG. |
| Low density lipoprotein (LDL ) assessed with: mg/dL № of participants: 1388 (15 RCTs) | - |  | - | MD **1.86 mg/dL lower** (8.9 lower to 5.18 higher) | ⨁◯◯◯ Very low ^a,b,d,g^ | The evidence is very uncertain about the effect of Vitamin D on LDL. |
| High density lipoprotein (HDL) assessed with: mg/dL № of participants: 1388 (15 RCTs) | - |  | - | MD **0.82 mg/dL higher** (0.71 lower to 2.36 higher) | ⨁◯◯◯ Very low ^a,b,d,e,g^ | Vitamin D may increase/have little to no effect on HDL but the evidence is very uncertain. |
| Total cholesterol (TC) assessed with: mg/dL № of participants: 1370 (14 RCTs) | - |  | - | MD **1.29 mg/dL lower** (9.33 lower to 6.76 higher) | ⨁◯◯◯ Very low ^a,b,d,e,g^ | Vitamin D may increase/have little to no effect on TC but the evidence is very uncertain. |
| Total triglycerides (TG) assessed with: mg/dL № of participants: 1370 (14 RCTs) | - |  | - | MD **7.95 mg/dL lower** (15.09 lower to 0.81 lower) | ⨁◯◯◯ Very low ^a,d,e,g^ | The evidence is very uncertain about the effect of Vitamin D on TG. |
| Adiponectin assessed with: ug/mL № of participants: 431 (6 RCTs) | - |  | - | MD **0.5 ug/mL higher** (1.14 lower to 2.14 higher) | ⨁◯◯◯ Very low ^a,b,d,e,g^ | The evidence is very uncertain about the effect of Vitamin D on adiponectin. |
| C reactive protein (CRP) assessed with: mg/L № of participants: 967 (9 RCTs) | - |  | - | MD **0.77 mg/L lower** (1.8 lower to 0.26 higher) | ⨁◯◯◯ Very low ^c,e,g^ | Vitamin D may result in little to no difference in CRP. |
| Interleukin 6 (IL-6) № of participants: 184 (3 RCTs) | - |  | - | MD **1.42 pg/mL lower** (4.54 lower to 1.69 higher) | ⨁◯◯◯ Very low ^a,b,e,g^ | Vitamin D may have little to no effect on IL-6. |
| Bilirubin assessed with: mg/dL № of participants: 570 (6 RCTs) | - |  | - | MD **0.18 mg/dL lower** (0.62 lower to 0.26 higher) | ⨁◯◯◯ Very low ^a,b,d,g^ | The evidence is very uncertain about the effect Vitamin D on bilirubin. |
| Albumin assessed with: g/dL № of participants: 736 (7 RCTs) | - |  | - | MD **0.01 g/dL higher** (0.11 lower to 0.13 higher) | ⨁◯◯◯ Very low ^a,b,c,e^ | The evidence is very uncertain about the effect of Vitamin D on albumin. |
| INR № of participants: 376 (3 RCTs) | - |  | - | MD **0.08 lower** (0.59 lower to 0.43 higher) | ⨁◯◯◯ Very low ^a,b,c,e^ | Vitamin D may reduce/have little to no effect on iNR but the evidence is very uncertain. |
| Sustained Virologic Response (SVR) № of participants: 674 (8 RCTs) | **RR 1.29** (0.96 to 1.74) | 51.2% | **66.0%** (49.1 to 89) | **14.8% more** (2 fewer to 37.9 more) | ⨁◯◯◯ Very low ^a,b,e^ | The evidence is very uncertain about the effect of Vitamin D on SVR. |
| ***The risk in the intervention group** (and its 95% confidence interval) is based on the assumed risk in the comparison group and the **relative effect** of the intervention (and its 95% CI).  **CI:** confidence interval; **MD:** mean difference; **RR:** risk ratio | | | | | | |
| **GRADE Working Group grades of evidence** **High certainty:** we are very confident that the true effect lies close to that of the estimate of the effect. **Moderate certainty:** we are moderately confident in the effect estimate: the true effect is likely to be close to the estimate of the effect, but there is a possibility that it is substantially different. **Low certainty:** our confidence in the effect estimate is limited: the true effect may be substantially different from the estimate of the effect. **Very low certainty:** we have very little confidence in the effect estimate: the true effect is likely to be substantially different from the estimate of effect. | | | | | | |

#### Explanations: a. Overall risk of bias is unclear or high concerning the eligible studies; b. Heterogeneity; c. Differences among studies; d. Width of the CI; e. Small sample size; f. Egger's test p value; g. Surrogate outcome.

**SUPPLEMENTARY FILE S7.** Risk of bias assessment (RoB-2 tool) about each risk of bias item for each included study.


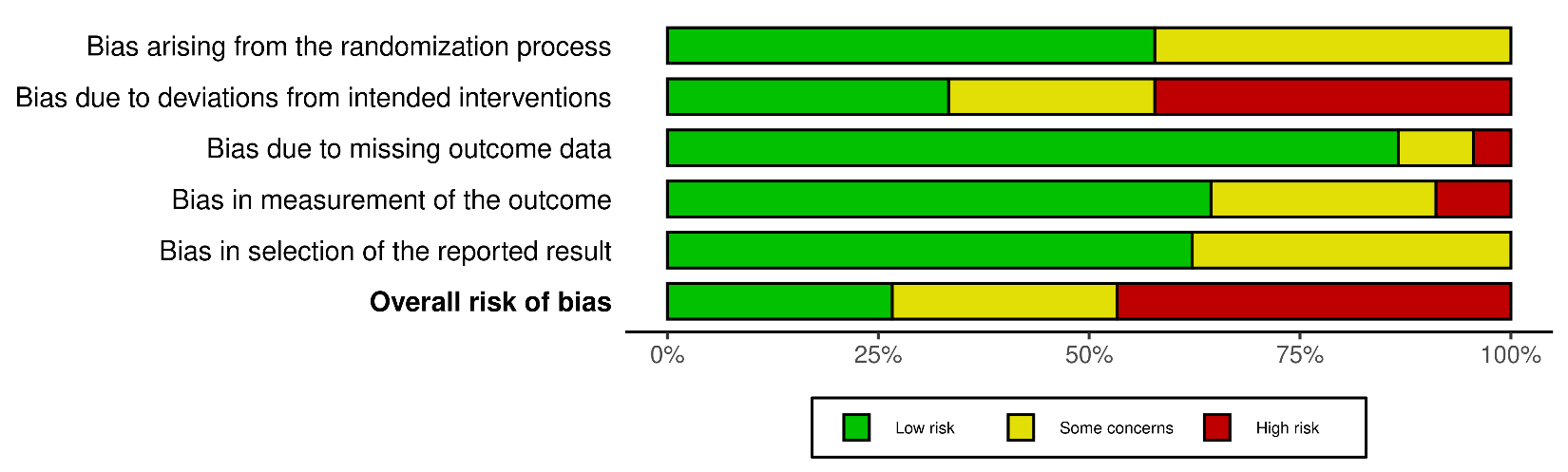


*Figure S7.1. Risk of bias assessment (RoB-2 tool) about each risk of bias item for each included study presented as percentages.*

**
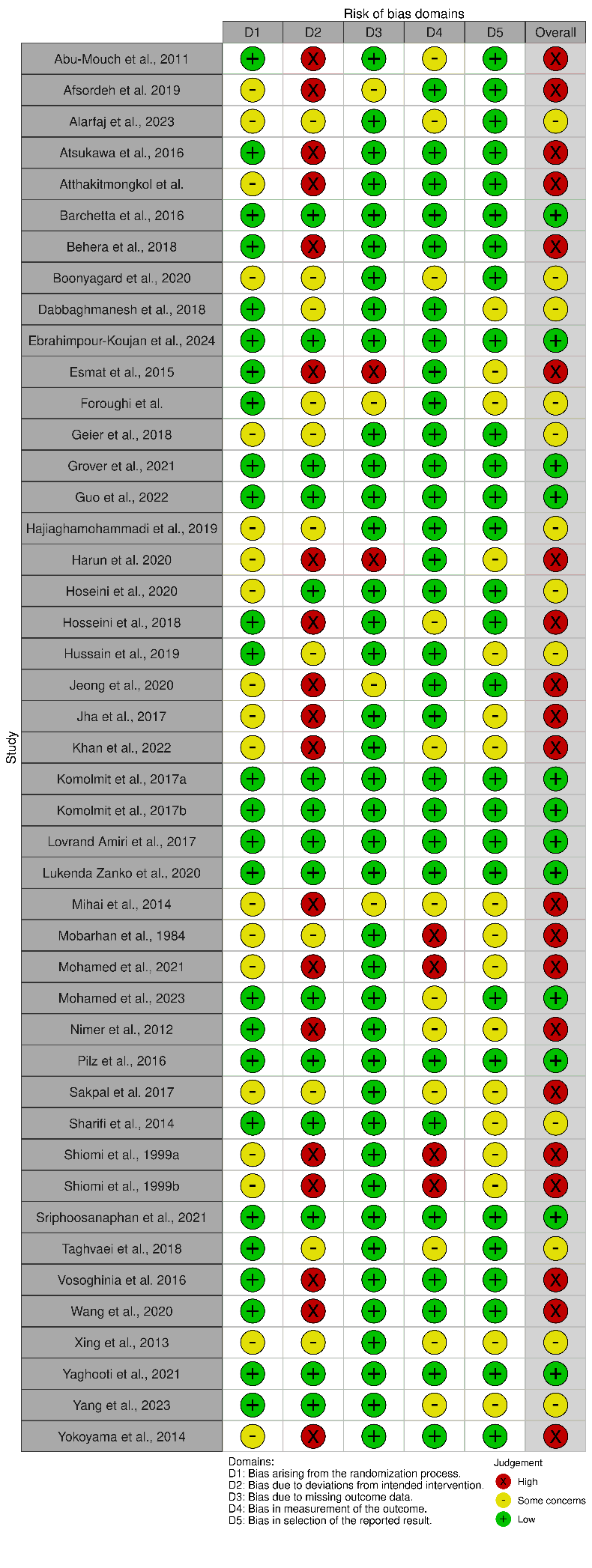
**

*Figure S7.2. Risk of bias assessment (RoB-2 tool) about each risk of bias item for each included study.*

**TABLE S8.** Excluded studies based on the full text selection.

| Author and year | Title | Reason for exclusion |
| --- | --- | --- |
| Abu Mouch et al. 2010 | Vitamin D supplement improves SVR in chronic hepatitis C (genotype 1) naive patients treated with PEG interferon and ribavirin | Abstract or duplicate of identified eligible article |
| Agarwal et al. 2024 | Longitudinal changes in bone mineral density may be associated with long-term survival in patients with cirrhosis: A proof of concept of study | Missing information about treatment allocation |
| Al-Bayyari et al. 2021 | Vitamin D (3) reduces risk of cardiovascular and liver diseases by lowering homocysteine levels: double-blinded, randomised, placebo-controlled trial | Ineligible population |
| Amani et al. 2017 | Gender differences in response to vitamin D supplementation regarding serum cardiometabolic biomarkers among patients with non alcoholic fatty liver disease | Abstract or duplicate of identified eligible article |
| Amiri et al. 2016 | Regression of Non-Alcoholic Fatty Liver by Vitamin D Supplement: A Double-Blind Randomized Controlled Clinical Trial | Abstract or duplicate of identified eligible article |
| Barchetta et al. 2016 | Effects of oral high-dose vitamin d supplementation on non-alcoholic fatty liver disease in patients with type 2 diabetes: A randomised, double-blind, placebo-controlled trial | Abstract or duplicate of identified eligible article |
| Basu et al. 2015 | Interferon ineligible naive chronic hepatitis C genotype i subjects treated with simeprevir and sofosbuvir in special population (PSYCHIATRIC). An open label prospective clinical pilot study; inspire c study | Ineligible study design |
| Boonyagard et al. 2017 | Impact of vitamin D replacement on liver enzymes in nonalcoholic fatty liver disease | Abstract or duplicate of identified eligible article |
| Charoensuk et al. 2014 | Correction of vitamin D deficiency correlated with suppression of soluble CD26 levels (SCD26) and interferon-gamma-inducible protein 10 (IP-10) in patients with chronic hepatitis C: A randomized, double-blinded, placebo-controlled pilot study | Missing outcome |
| Charoensuk et al. 2013 | Correction of vitamin D deficiency in patients with chronic hepatitis C resulting in suppression of serum interferon-gamma-inducible protein 10 (IP-10) and soluble CD26 (sCD26) levels: A randomized, double-blinded, placebo-controlled study | Missing outcome |
| Charoensuk et al. 2013 | Correction of vitamin D deficiency in patients with chronic hepatitis C facilitated suppression of interferon-gamma-inducible protein 10 (IP-10): A randomized, double-blinded, placebo-controlled study | Missing outcome |
| Charoensuk et al. 2013 | Effect of vitamin D supplement on T helper1/2 cytokine levels in chronic hepatitis C patients; A randomized, double-blind, placebo-controlled pilot study | Missing outcome |
| Fan et al. 2022 | Elevated serum phosphatidylcholine (16:1/22:6) levels promoted by fish oil and vitamin D(3) are highly correlated with biomarkers of non-alcoholic fatty liver disease in Chinese subjects | Missing outcome |
| Foroughi et al. 2014 | The effect of vitamin D supplementation on lipid profile in patients with non-alcoholic fatty liver (NAFLD) | Missing outcome for the control group |
| Foroughi et al. 2015 | The effect of vitamin D supplementation on insulin resistance in patients with nonalcoholic fatty liver | Abstract or duplicate of identified eligible article |
| Grover et al. 2019 | Effect of calcium and vitamin D supplementation on bone density in patients with cirrhosis- a double-blind randomized controlled trial | Abstract or duplicate of identified eligible article |
| Grover et al. 2022 | Longitudinal Changes in Bone Density Assessed by DEXA Scan Predicts Outcomes in Patients with Cirrhosis: A 5-Year Follow-up Study | Abstract or duplicate of identified eligible article |
| Grünhage et al. 2015 | Effects of vitamin D supplementation on liver stiffness in patients with chronic liver diseases | Ineligible study design |
| Kimtrakool et al. 2015 | Correction of vitamin D deficiency in chronic hepatitis c patients suppressed serum fibrotic markers: A randomized, double-blinded, placebo-controlled study | Abstract or duplicate of identified eligible article |
| Kondo et al. 2013 | 1(OH)vit D3 supplementation improves the sensitivity of the immune-response during Peg-IFN/RBV therapy in chronic hepatitis C (CH-C) and CH-C with severe fibrosis | Ineligible study design |
| König et al. 2015 | Effects of vitamin d supplementation on liver stiffness in patients with chronic liver diseases | Ineligible study design |
| Kukhareva et al. 2017 | The influence of correction of vitamin D deficiency on the formation of the stable virological response during therapy of genotype-1 chronic hepatitis C with PEGylated interferon-alpha-2 and ribavirin | Ineligible study design |
| Li et al. 2024 | Effects of dietary supplementation of fish oil plus vitamin D3 on gut microbiota and fecal metabolites, and their correlation with nonalcoholic fatty liver disease risk factors: a randomized controlled trial | Missing full text |
| Luger et al. 2016 | Vitamin D loading dose supplementation after metabolic surgery and impact of biopsy-proven liver fibrosis and impaired renal function in morbidly obese patients | Missing outcome |
| Luger et al. 2017 | Vitamin D3 Loading Is Superior to Conventional Supplementation After Weight Loss Surgery in Vitamin D-Deficient Morbidly Obese Patients: a Double-Blind Randomized Placebo-Controlled Trial | Missing outcome |
| Mansourian et al. 2017 | The effect of a single intramuscular injection of cholecalciferol on serum levels of vitamin D, liver enzymes and severity of steatosis in vitamin D deficit women with non-alcoholic fatty liver disease (NAFLD): a randomized controlled clinical trial | Abstract or duplicate of identified eligible article |
| Mikolasevic et al. 2019 | Treatment of nonalcoholic fatty liver disease with vitamin D: A double-blinded, randomized, placebo-controlled pilot study | Abstract or duplicate of identified eligible article |
| Mikolasevic et al. 2020 | The efficacy of vitamin D supplementation on NAFLD: a randomized, double-blind, placebo-controlled 12- month trial on 311 patients | Abstract or duplicate of identified eligible article |
| Morvaridzadeh et al. 2021 | Probiotic Yogurt Fortified with Vitamin D Can Improve Glycemic Status in Non-Alcoholic Fatty Liver Disease Patients: a Randomized Clinical Trial | Different standard of care practice between the groups |
| Mouch et Assy 2010 | Vitamin D supplementation improves viral response in chronic hepatitis C genotype 2/3 patients treated with Peg interferon alpha and ribavirin | Abstract or duplicate of identified eligible article |
| Rahimpour et al. 2022 | Alterations of liver enzymes and lipid profile in response to exhaustive eccentric exercise: vitamin D supplementation trial in overweight females with non-alcoholic fatty liver disease | Additional treatment |
| Rahimpour et al. 2022 | Effect of Short-term Vitamin D Supplementation on the Alterations of Glycemic Variables in Response to Exhaustive Eccentric Exercise in Patients with Non-alcoholic Fatty Liver | Additional treatment |
| Sabry et al. 2015 | Effect of vitamin D therapy on interleukin-6, visfatin, and hyaluronic acid levels in chronic hepatitis C Egyptian patients | Missing outcome for the control group |
| Sakpal et al. 2015 | Vitamin D supplementation in patients with nonalcoholic fatty liver disease-A randomized controlled trial | Abstract or duplicate of identified eligible article |
| Sriphoosanaphan et al. 2019 | Effect of vitamin D supplement on hepatic fibrogenesis markers in chronic hepatitis C patients after direct-acting antiviral therapy: A randomized, double-blinded, placebo-controlled trial | Abstract or duplicate of identified eligible article |
| Stokes et al. 2011 | Vitamin D and depression in chronic liver disease | Ineligible study design |
| Yurci et al. 2011 | Efficacy of different therapeutic regimens on hepatic osteodystrophy in chronic viral liver disease | Additional treatment |

**REFERENCE**

1. Higgins JPT TJ, Chandler J, Cumpston M, Li T, Page MJ, Welch VA (editors). Cochrane Handbook for Systematic Reviews of Interventions version 6.3 (updated February 2022). Cochrane, 2022. Available from <www.training.cochrane.org/handbook>.

2. Higgins JPT ES, Li T (editors). Chapter 23: Including variants on randomized trials. In: Higgins JPT, Thomas J, Chandler J, Cumpston M, Li T, Page MJ, Welch VA (editors). Cochrane Handbook for Systematic Reviews of Interventions version 6.3 (updated February 2022). Cochrane, 2022. Available from <www.training.cochrane.org/handbook>.

3. Luo D, Wan X, Liu J, Tong T. Optimally estimating the sample mean from the sample size, median, mid-range, and/or mid-quartile range. *Stat Methods Med Res*. Jun 2018;27(6):1785-1805. doi:10.1177/0962280216669183

4. Shi J, Luo D, Weng H, et al. Optimally estimating the sample standard deviation from the five-number summary. *Res Synth Methods*. Sep 2020;11(5):641-654. doi:10.1002/jrsm.1429

5. Hoseini Z, Behpour N, Hoseini R. Co-treatment with Vitamin D Supplementation and Aerobic Training in Elderly Women with Vit D Deficiency and NAFLD: A Single-blind Controlled Trial. *Hepatitis Monthly*. 2020;20(2)doi:10.5812/hepatmon.96437

6. Afsordeh Kolsoum RR, Alizadeh Aliakbar. Effect of aerobic training and vitamin D supplements on fatty liver and lipid profiles in women with fatty liver. Original. *Pejouhesh dar Pezeshki (Research in Medicine)*. 2019;43(1):8-14.

7. Dabbaghmanesh MH, Danafar F, Eshraghian A, Omrani GR. Vitamin D supplementation for the treatment of non-alcoholic fatty liver disease: A randomized double blind placebo controlled trial. *Diabetes Metab Syndr*. Jul 2018;12(4):513-517. doi:10.1016/j.dsx.2018.03.006

8. Mobarhan SA, Russell RM, Recker RR, Posner DB, Iber FL, Miller P. Metabolic bone disease in alcoholic cirrhosis: a comparison of the effect of vitamin D2, 25-hydroxyvitamin D, or supportive treatment. *Hepatology*. Mar-Apr 1984;4(2):266-73. doi:10.1002/hep.1840040216

9. Lorvand Amiri H, Agah S, Tolouei Azar J, Hosseini S, Shidfar F, Mousavi SN. Effect of daily calcitriol supplementation with and without calcium on disease regression in non-alcoholic fatty liver patients following an energy-restricted diet: Randomized, controlled, double-blind trial. *Clin Nutr*. Dec 2017;36(6):1490-1497. doi:10.1016/j.clnu.2016.09.020

10. Sharifi N, Amani R, Hajiani E, Cheraghian B. Does vitamin D improve liver enzymes, oxidative stress, and inflammatory biomarkers in adults with non-alcoholic fatty liver disease? A randomized clinical trial. *Endocrine*. Sep 2014;47(1):70-80. doi:10.1007/s12020-014-0336-5

11. Sharifi N, Amani R, Hajiani E, Cheraghian B. Women may respond different from men to vitamin D supplementation regarding cardiometabolic biomarkers. Article. *Experimental Biology and Medicine*. 2016;241(8):830-838. doi:10.1177/1535370216629009

12. Veroniki AA, Jackson D, Viechtbauer W, et al. Methods to estimate the between-study variance and its uncertainty in meta-analysis. *Res Synth Methods*. Mar 2016;7(1):55-79. doi:10.1002/jrsm.1164

13. N MANTEL WH. Statistical aspects of the analysis of data from retrospective studies of disease. *J Natl Cancer Inst*. 1959;22(4):719-48.

14. J Robins SG, N E Breslow. A general estimator for the variance of the Mantel-Haenszel odds ratio. *Am J Epidemiol*. 1986;124(5):719-23. doi:10.1093/oxfordjournals.aje.a114447.

15. Robert C Paule JM. Consensus Values and Weighting Factors. *J Res Natl Bur Stand (1977)*. 1982;87(5):377-385. doi:10.6028/jres.087.022.

16. Cooper H, Hedges, L. V., & Valentine, J. C. . *The Handbook of Research Synthesis and Meta-Analysis (2nd edition)*. 2009:632.

17. Michael J Sweeting AJS, Paul C Lambert. What to add to nothing? Use and avoidance of continuity corrections in meta-analysis of sparse data. *Stat Med*. 15.05.2004 2004;23(9):1351-75. doi:10.1002/sim.1761

18. Guido Knapp JH. Improved tests for a random effects meta-regression with a single covariate. *Stat Med*. 15.09.2003 2003;22(17):2693-710. doi:2693-710.

19. IntHout J, Ioannidis JP, Borm GF. The Hartung-Knapp-Sidik-Jonkman method for random effects meta-analysis is straightforward and considerably outperforms the standard DerSimonian-Laird method. *BMC Med Res Methodol*. Feb 18 2014;14:25. doi:10.1186/1471-2288-14-25

20. Jackson D, Turner R. Power analysis for random-effects meta-analysis. *Res Synth Methods*. Sep 2017;8(3):290-302. doi:10.1002/jrsm.1240

21. Harrer M, Cuijpers P, Furukawa TA, Ebert DD. *Doing Meta-Analysis with R*. 2021.

22. Higgins JP, Thompson SG. Quantifying heterogeneity in a meta-analysis. *Stat Med*. Jun 15 2002;21(11):1539-58. doi:10.1002/sim.1186

23. Egger M, Davey Smith G, Schneider M, Minder C. Bias in meta-analysis detected by a simple, graphical test. *BMJ*. Sep 13 1997;315(7109):629-34. doi:10.1136/bmj.315.7109.629

24. Harbord RM, Harris RJ, Sterne JAC. Updated Tests for Small-study Effects in Meta-analyses. *The Stata Journal: Promoting communications on statistics and Stata*. 2009;9(2):197-210. doi:10.1177/1536867x0900900202

25. R Core Team. R: A language and environment for statistical computing. Vienna ARFfSC, 2023 <https://www.R-project.org/>.

26. *Schwarzer G. Meta: General package for meta-analysis. 2023* [*https://github.com/guido-s/meta/*](https://github.com/guido-s/meta/) [*https://link.springer.com/book/10.1007/978-3-319-21416-0*](https://link.springer.com/book/10.1007/978-3-319-21416-0)*.*

27. Cuijpers P, Furukawa T, Ebert DD. Dmetar: Companion r package for the guide doing meta-analysis in r. 2023 <https://dmetar.protectlab.org>.

28. Viechtbauer W. Metafor: Meta-analysis package for r. 2023 <https://CRAN.R-project.org/package=metafor>.

29. Abu-Mouch S, Fireman Z, Jarchovsky J, Zeina AR, Assy N. Vitamin D supplementation improves sustained virologic response in chronic hepatitis C (genotype 1)-naïve patients. *World J Gastroenterol*. Dec 21 2011;17(47):5184-90. doi:10.3748/wjg.v17.i47.5184

30. Alarfaj SJ, Bahaa MM, Yassin HA, et al. A randomized placebo-controlled, double-blind study to investigate the effect of a high oral loading dose of cholecalciferol in non-alcoholic fatty liver disease patients, new insights on serum STAT-3 and hepassocin. *Eur Rev Med Pharmacol Sci*. Aug 2023;27(16):7607-7619. doi:10.26355/eurrev_202308_33413

31. Atsukawa M, Tsubota A, Shimada N, et al. Effect of native vitamin D3 supplementation on refractory chronic hepatitis C patients in simeprevir with pegylated interferon/ribavirin. *Hepatol Res*. Mar 2016;46(5):450-8. doi:10.1111/hepr.12575

32. Atthakitmongkol T, Chotiyaputta W, Lertwattanarak R, Sritippayawan S, Tanwandee T. Vitamin D and calcium supplementation prevent bone loss in vitamin d-deficient chronic hepatitis B patients treated with tenofovir disoproxil fumarate: a randomized controlled study. Conference Abstract. *Gastroenterology*. 2023;164(6):S-1360-S-1361. doi:10.1016/S0016-5085(23)04168-9

33. Barchetta I, Del Ben M, Angelico F, et al. No effects of oral vitamin D supplementation on non-alcoholic fatty liver disease in patients with type 2 diabetes: a randomized, double-blind, placebo-controlled trial. *BMC Med*. Jun 29 2016;14:92. doi:10.1186/s12916-016-0638-y

34. Behera MK, Shukla SK, Dixit VK, et al. Effect of vitamin D supplementation on sustained virological response in genotype 1/4 chronic hepatitis C treatment-naïve patients from India. *Indian J Med Res*. Aug 2018;148(2):200-206. doi:10.4103/ijmr.IJMR_1295_15

35. Boonyagard S, Techathuvanan K. Impact of Vitamin D Replacement on Liver Enzymes in Non-Alcoholic Fatty Liver Disease Patients: A Randomized, Double-blind, Placebo-controlled Trial. Article. *Journal of the Medical Association of Thailand*. 2020;103(12):A105-A112. doi:10.35755/jmedassocthai.2020.S08.12052

36. Ebrahimpour-Koujan S, Sohrabpour AA, Giovannucci E, Vatannejad A, Esmaillzadeh A. Effects of vitamin D supplementation on liver fibrogenic factors, vitamin D receptor and liver fibrogenic microRNAs in metabolic dysfunction-associated steatotic liver disease (MASLD) patients: an exploratory randomized clinical trial. *Nutr J*. Feb 27 2024;23(1):24. doi:10.1186/s12937-024-00911-x

37. Esmat G, El Raziky M, Elsharkawy A, et al. Impact of vitamin D supplementation on sustained virological response in chronic hepatitis C genotype 4 patients treated by pegylated interferon/ribavirin. *J Interferon Cytokine Res*. Jan 2015;35(1):49-54. doi:10.1089/jir.2014.0060

38. Foroughi M, Maghsoudi Z, Ghiasvand R, Iraj B, Askari G. Effect of vitamin D supplementation on C-reactive protein in patients with nonalcoholic fatty liver. Article. *International Journal of Preventive Medicine*. 2014;5(8):969-975.

39. Foroughi M, Maghsoudi Z, Askari G. The effect of vitamin D supplementation on blood sugar and different indices of insulin resistance in patients with non-alcoholic fatty liver disease (NAFLD). *Iran J Nurs Midwifery Res*. Jan-Feb 2016;21(1):100-4. doi:10.4103/1735-9066.174759

40. Geier A, Eichinger M, Stirnimann G, et al. Treatment of non-alcoholic steatohepatitis patients with vitamin D: a double-blinded, randomized, placebo-controlled pilot study. *Scand J Gastroenterol*. Sep 2018;53(9):1114-1120. doi:10.1080/00365521.2018.1501091

41. Grover I, Gunjan D, Singh N, et al. Effect of Vitamin D Supplementation on Vitamin D Level and Bone Mineral Density in Patients With Cirrhosis: A Randomized Clinical Trial. *Am J Gastroenterol*. Oct 1 2021;116(10):2098-2104. doi:10.14309/ajg.0000000000001272

42. Grover I, Sharma S, Madhu D, et al. Effect of vitamin d supplementation on clinical outcomes in patients with cirrhosis: A post hoc analysis of a randomized controlled trial. Conference Abstract. *Hepatology*. 2021;74(SUPPL 1):1213A-1214A. doi:10.1002/hep.32188

43. Guo XF, Wang C, Yang T, et al. The effects of fish oil plus vitamin D(3) intervention on non-alcoholic fatty liver disease: a randomized controlled trial. *Eur J Nutr*. Jun 2022;61(4):1931-1942. doi:10.1007/s00394-021-02772-0

44. Hajiaghamohammadi A, Shafikhani AA, Bastani A, Gaemi N. Effect of Vitamin D replacement on liver enzymes in patients with non-alcoholic fatty liver disease. Article. *Journal of Gastroenterology and Hepatology Research*. 2019;8(3):2907-2910. doi:10.17554/j.issn.2224-3992.2019.08.819

45. Harun M, Rashid. Effect of vitamin D treatment in patients with decompensated cirrhosis of liver-report from a Tertiary Centre, Bangladesh. Conference Abstract. *Hepatology International*. 2020;14:S407. doi:10.1007/s12072-020-10030-4

46. Hosseini S, Aliashrafi S, Ebrahimi-Mameghani M. The Effect of a Single Intramuscular Injection of Cholecalciferol on the Serum Levels of Vitamin D, Adiponectin, Insulin Resistance, and Liver Function in Women with Non-Alcoholic Fatty Liver Disease (NAFLD): A Randomized, Controlled Clinical Trial. *Iranian Red Crescent Medical Journal*. 09/18 2018;In Pressdoi:10.5812/ircmj.60746

47. Hussain M, Iqbal J, Malik SA, et al. Effect of vitamin D supplementation on various parameters in non-alcoholic fatty liver disease patients. Article. *Pakistan journal of pharmaceutical sciences*. 2019;32(3):1343-1348.

48. Jeong JY, Jun DW, Park SJ, et al. Effects of vitamin D supplements in patients with chronic hepatitis C: a randomized, multi-center, open label study. *Korean J Intern Med*. Sep 2020;35(5):1074-1083. doi:10.3904/kjim.2018.273

49. Jha AK, Jha SK, Kumar A, Dayal VM, Jha SK. Effect of replenishment of vitamin D on survival in patients with decompensated liver cirrhosis: A prospective study. *World J Gastrointest Pathophysiol*. Aug 15 2017;8(3):133-141. doi:10.4291/wjgp.v8.i3.133

50. Khan J, Adil N, Naseer A, Asif M, Sabir A, Fatima S. Comparison of Frequency of Rapid Virological Response in Patients of Hepatitis C being Treated with 25-OH Vitamin D along with Sofosbuvir/Ribavirin with Those Treated with Sofosbuvir/Ribavirin only. *Pakistan Journal of Medical and Health Sciences*. 06/29 2022;16:342-343. doi:10.53350/pjmhs22166342

51. Komolmit P, Charoensuk K, Thanapirom K, et al. Correction of vitamin D deficiency facilitated suppression of IP-10 and DPP IV levels in patients with chronic hepatitis C: A randomised double-blinded, placebo-control trial. *PLoS One*. 2017;12(4):e0174608. doi:10.1371/journal.pone.0174608

52. Komolmit P, Kimtrakool S, Suksawatamnuay S, et al. Vitamin D supplementation improves serum markers associated with hepatic fibrogenesis in chronic hepatitis C patients: A randomized, double-blind, placebo-controlled study. *Sci Rep*. Aug 21 2017;7(1):8905. doi:10.1038/s41598-017-09512-7

53. Lorvand Amiri H, Agah S, Mousavi SN, Hosseini AF, Shidfar F. Regression of non-alcoholic fatty liver by vitamin D supplement: A double-blind randomized controlled clinical trial. Article. *Archives of Iranian Medicine*. 2016;19(9):631-638.

54. Shidfar F, Mousavi SN, Lorvand Amiri H, Agah S, Hoseini S, Hajimiresmail SJ. Reduction of Some Atherogenic Indices in Patients with Non-Alcoholic Fatty Liver by Vitamin D and Calcium Co-Supplementation: A Double Blind Randomized Controlled Clinical Trial. *Iran J Pharm Res*. Winter 2019;18(1):496-505.

55. Lukenda Zanko V, Domislovic V, Trkulja V, et al. Vitamin D for treatment of non-alcoholic fatty liver disease detected by transient elastography: A randomized, double-blind, placebo-controlled trial. *Diabetes Obes Metab*. Nov 2020;22(11):2097-2106. doi:10.1111/dom.14129

56. Mihai C, Dranga M, Drug V, Prelipcean CC. Su1077 Vitamin D and B12 Supplementation and Sustained Virologic Response in Chronic Hepatitis C Patients Treated With Pegylated Interferon and Ribavirin. *Gastroenterology*. 2014;146(5)doi:10.1016/s0016-5085(14)63568-x

57. Mohamed AA, Al-Karmalawy AA, El-Kholy AA, et al. Effect of Vitamin D supplementation in patients with liver cirrhosis having spontaneous bacterial peritonitis: a randomized controlled study. *Eur Rev Med Pharmacol Sci*. Nov 2021;25(22):6908-6919. doi:10.26355/eurrev_202111_27239

58. Mohamed AA, Halim AA, Mohamed S, et al. The effect of high oral loading dose of cholecalciferol in non-alcoholic fatty liver disease patients. A randomized placebo controlled trial. *Front Pharmacol*. 2023;14:1149967. doi:10.3389/fphar.2023.1149967

59. Nimer A, Mouch A. Vitamin D improves viral response in hepatitis C genotype 2-3 naïve patients. *World J Gastroenterol*. Feb 28 2012;18(8):800-5. doi:10.3748/wjg.v18.i8.800

60. Okubo T, Atsukawa M, Tsubota A, et al. Effect of Vitamin D Supplementation on Skeletal Muscle Volume and Strength in Patients with Decompensated Liver Cirrhosis Undergoing Branched Chain Amino Acids Supplementation: A Prospective, Randomized, Controlled Pilot Trial. *Nutrients*. May 30 2021;13(6)doi:10.3390/nu13061874

61. Pilz S, Putz-Bankuti C, Gaksch M, et al. Effects of Vitamin D Supplementation on Serum 25-Hydroxyvitamin D Concentrations in Cirrhotic Patients: A Randomized Controlled Trial. *Nutrients*. May 10 2016;8(5)doi:10.3390/nu8050278

62. Sakpal M, Satsangi S, Mehta M, et al. Vitamin D supplementation in patients with nonalcoholic fatty liver disease: A randomized controlled trial. *JGH Open*. Oct 2017;1(2):62-67. doi:10.1002/jgh3.12010

63. Shiomi S, Masaki K, Habu D, et al. Calcitriol for bone disease in patients with cirrhosis of the liver. *J Gastroenterol Hepatol*. Jun 1999;14(6):547-52. doi:10.1046/j.1440-1746.1999.01913.x

64. Shiomi S, Masaki K, Habu D, et al. Calcitriol for bone loss in patients with primary biliary cirrhosis. *J Gastroenterol*. Apr 1999;34(2):241-5. doi:10.1007/s005350050250

65. Sriphoosanaphan S, Thanapirom K, Kerr SJ, et al. Effect of vitamin D supplementation in patients with chronic hepatitis C after direct-acting antiviral treatment: a randomized, double-blind, placebo-controlled trial. *PeerJ*. 2021;9:e10709. doi:10.7717/peerj.10709

66. Taghvaei T, Akha O, Mouodi M, Tirgar Fakheri H, Kashi Z, Maleki I. Effects of vitamin d supplementation on patients with non-alcoholic fatty liver disease (nafld). *Acta Medica Mediterranea*. 01/01 2018;34:415-422. doi:10.19193/0393-6384_2018_2_66

67. Vosoghinia H, Esmaeilzadeh A, Ganji A, et al. Vitamin D in standard HCV regimen (PEG-interferon plus ribavirin), its effect on the early virologic response rate: A clinical trial. Article. *Razavi International Journal of Medicine*. 2016;4(2)doi:10.17795/rijm36632

68. Wang CC, Tzeng IS, Su WC, et al. The association of vitamin D with hepatitis B virus replication: Bystander rather than offender. *J Formos Med Assoc*. Nov 2020;119(11):1634-1641. doi:10.1016/j.jfma.2019.12.004

69. Xing T, Qiu G, Zhong L, Ling L, Huang L, Peng Z. Calcitriol reduces the occurrence of acute cellular rejection of liver transplants: a prospective controlled study. *Pharmazie*. Oct 2013;68(10):821-6.

70. Yaghooti H, Ghanavati F, Seyedian SS, Cheraghian B, Mohammadtaghvaei N. The efficacy of calcitriol treatment in non-alcoholic fatty liver patients with different genotypes of vitamin D receptor FokI polymorphism. *BMC Pharmacol Toxicol*. Apr 7 2021;22(1):18. doi:10.1186/s40360-021-00485-y

71. Yang Z, Haijun C, Yejin X, Dehe Z, Jing Z, Shengnan L. Efficacy of vitamin D adjuvant therapy for prevention of spontaneous bacterial peritonitis in patients with decompensated cirrhosis of hepatitis B. Article. *Chinese Journal of Clinical Infectious Diseases*. 2023;16(3):215-219. doi:10.3760/cma.j.issn.1674-2397.2023.03.009

72. Yokoyama S, Takahashi S, Kawakami Y, et al. Effect of vitamin D supplementation on pegylated interferon/ribavirin therapy for chronic hepatitis C genotype 1b: a randomized controlled trial. *J Viral Hepat*. May 2014;21(5):348-56. doi:10.1111/jvh.12146
