# Supplementary material for: Role of Vitamin D Supplementation in Chronic Liver Disease: A Systematic Review and Meta-Analysis of Randomized Controlled Trials
Source: Nutr Rev. 2025 Jul 11;83(11):2043–54. doi: 10.1093/nutrit/nuaf117 (PMC12512233; doi:10.1093/nutrit/nuaf117)
Supplement: nuaf117_Supplementary_Data [file nuaf117_supplementary_data.zip › Supplementary file S2.docx]

**SUPPLEMENTARY MATERIAL – SUPPLEMENTARY FILE S2**

**SUPPLEMENTARY FILE S2.** Liver enzymes.

**FIGURE AND TABLE LEGENDS**

Figure S2.1a. Forest plot showing alanine aminotransferase (ALT) change in vitamin D and control groups.

Figure S2.1b. Funnel plot for alanine aminotransferase.

Figure S2.1c. Forest plot with leave-one-out analysis for alanine aminotransferase.

Figure S2.1d. Baujat plot for alanine aminotransferase.

Figure S2.2. Forest plot showing ALT change in vitamin D and control groups by length of intervention.

Figure S2.3. Forest plot showing ALT change in vitamin D and control groups divided into vitamin D deficient/insufficient (< 30 ng/mL) and sufficient (≥ 30 ng/mL) studies.

Figure S2.4. Forest plot showing ALT change in vitamin D and control groups excluding high-risk biased studies.

Figure S2.5. Forest plot showing ALT change in vitamin D and control groups by type of chronic liver disease.

Figure S2.6a. Forest plot showing aspartate aminotransferase (AST) change in vitamin D and control groups.

Figure S2.6b. Funnel plot for aspartate aminotransferase.

Figure S2.6c. Forest plot with leave-one-out analysis for aspartate aminotransferase.

Figure S2.6d. Baujat plot for aspartate aminotransferase.

Figure S2.7. Forest plot showing AST change in vitamin D and control groups by length of intervention.

Figure S2.8. Forest plot showing AST change in vitamin D and control groups divided into vitamin D deficient/insufficient (< 30 ng/mL) and sufficient (≥ 30 ng/mL) studies.

Figure S2.9. Forest plot showing AST change in vitamin D and control groups excluding high-risk biased studies.

Figure S2.10. Forest plot showing AST change in vitamin D and control groups by type of chronic liver disease.

Figure S2.11a: Forest plot showing gamma-glutamyl transferase (GGT) change in vitamin D and control groups.

Figure S2.11b. Funnel plot for gamma-glutamyl transferase.

Figure S2.11c. Forest plot with leave-one-out analysis for gamma-glutamyl transferase.

Figure S2.11d. Baujat plot for gamma-glutamyl transferase.

Figure S2.12. Forest plot showing GGT change in vitamin D and control groups by length of intervention.

Figure S2.13. Forest plot showing GGT change in vitamin D and control groups divided into vitamin D deficient/insufficient (< 30 ng/mL) and sufficient (≥ 30 ng/mL) studies.

Figure S2.14. Forest plot showing GGT change in vitamin D and control groups excluding high-risk biased studies.

Figure S2.15. Forest plot showing GGT change in vitamin D and control groups by type of chronic liver disease.

Figure S2.16a: Forest plot showing alkaline phosphatase (ALP) change in vitamin D and control groups.

Figure S2.16b. Funnel plot for alkaline phosphatase.

Figure S2.16c. Forest plot with leave-one-out analysis for alkaline phosphatase.

Figure S2.16d. Baujat plot for alkaline phosphatase.

Figure S2.17. Forest plot showing ALP change in vitamin D and control groups by length of intervention.

Figure S2.18. Forest plot showing ALP change in vitamin D and control groups divided into vitamin D deficient/insufficient (< 30 ng/mL) and sufficient (≥ 30 ng/mL) studies.

Figure S2.19. Forest plot showing ALP change in vitamin D and control groups excluding high-risk biased studies.

Figure S2.20. Forest plot showing ALP change in vitamin D and control groups by type of chronic liver disease.

**TABLE S5.** Baseline level of liver enzymes in the included studies.

**Supplementary File S2**. Liver enzymes

***ALT***


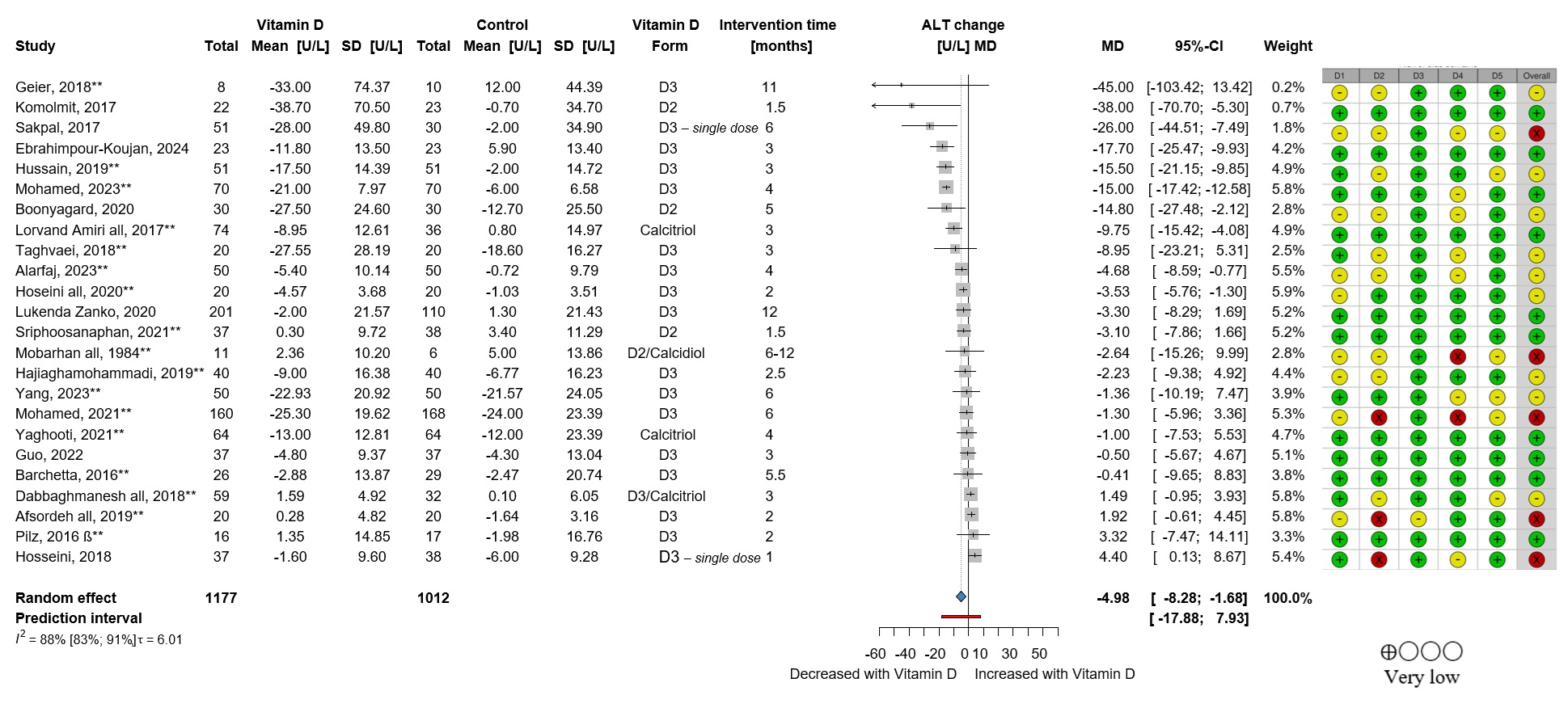


Figure S2.1a: Forest plots showing alanine aminotransferase (ALT) change in vitamin D and control groups. CI: confidence interval; MD: mean difference; SD: standard deviation. If the study is indicated with **, then the change value is an estimated change value in that study. The β means that the mean and SD are estimated mean and SD in that study. See raw data and synthesis methods.

Figure S2.1b: Funnel plot for alanine aminotransferase (p = 0.3943).

Figure S2.1c: Forest plot with leave-one-out analysis for alanine aminotransferase.

Figure S2.1d: Baujat plot for alanine aminotransferase.


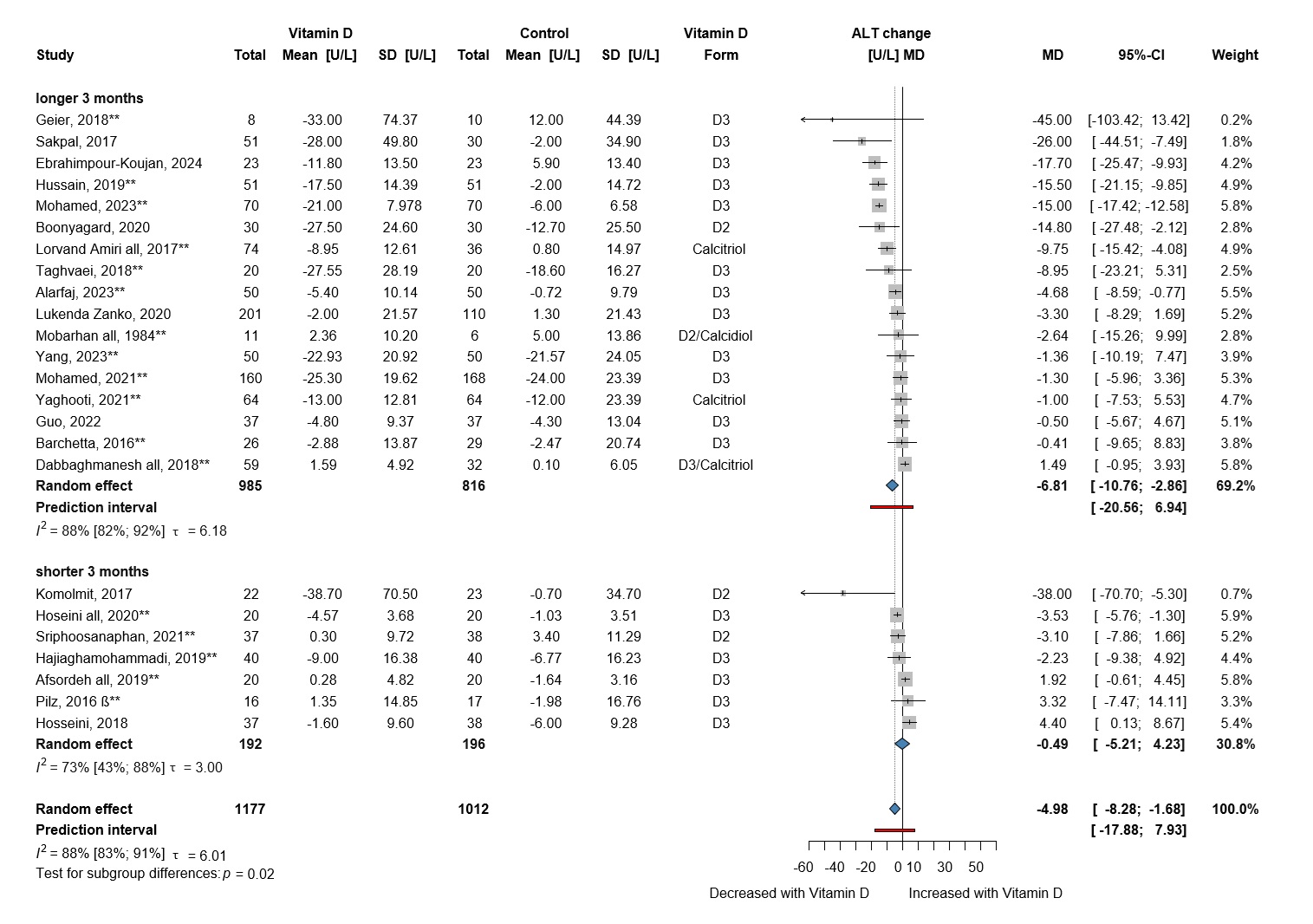


*Figure S2.2: Forest plots showing ALT change in vitamin D and control groups by length of intervention. ALT: alanine aminotransferase; CI: confidence interval; MD: mean difference; SD: standard deviation. If the study is indicated with **, then the change value is an estimated change value in that study. The β means that the mean and SD are estimated mean and SD in that study. See raw data and synthesis methods.*


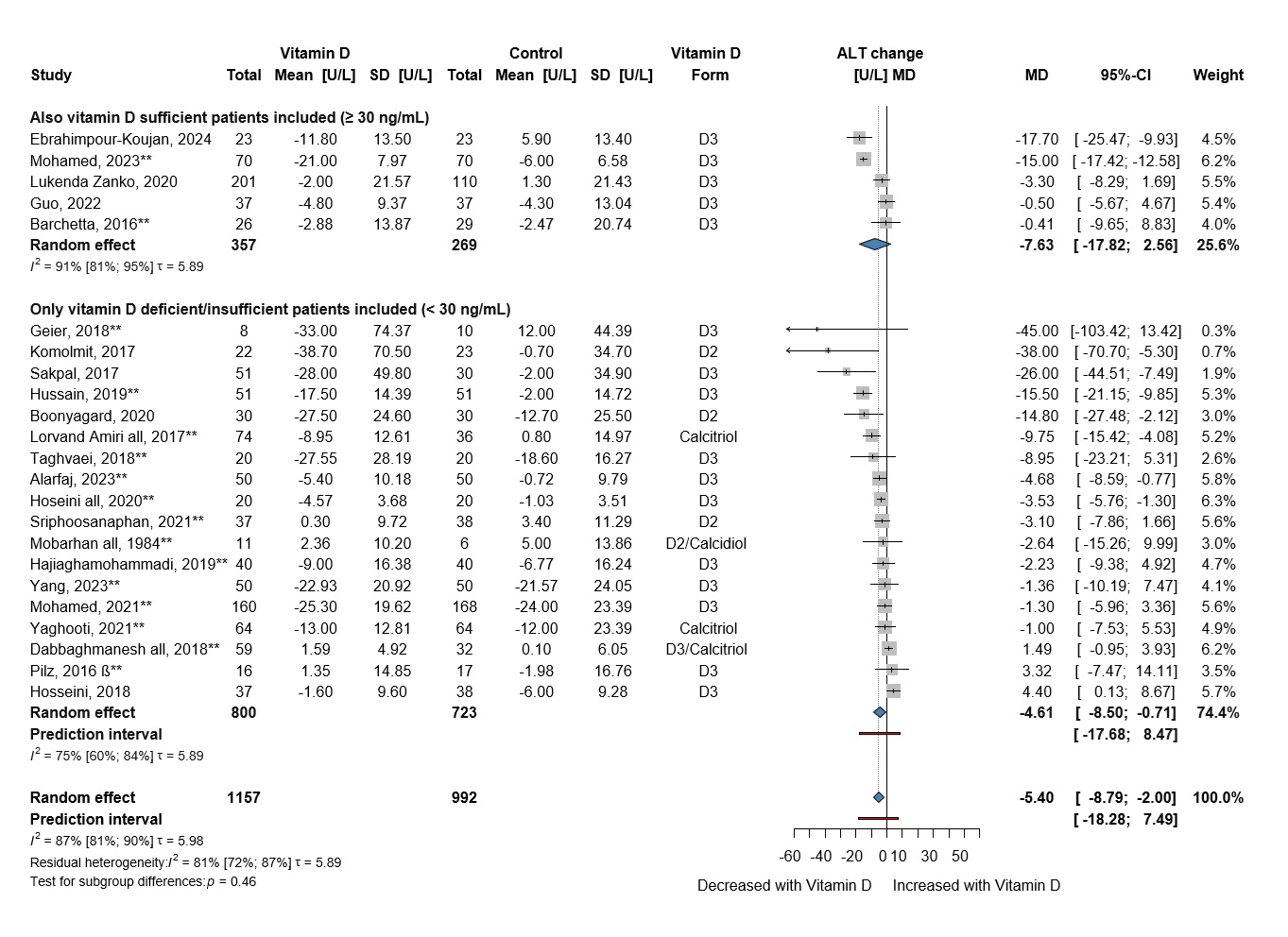


Figure S2.3: Forest plots showing ALT change in vitamin D and control groups divided into vitamin D deficient/insufficient (> 30 ng/mL) and sufficient (≥ 30 ng/mL) studies. ALT: alanine aminotransferase; CI: confidence interval; MD: mean difference; SD: standard deviation. If the study is indicated with **, then the change value is an estimated change value in that study. The β means that the mean and SD are estimated mean and SD in that study. See raw data and synthesis methods.


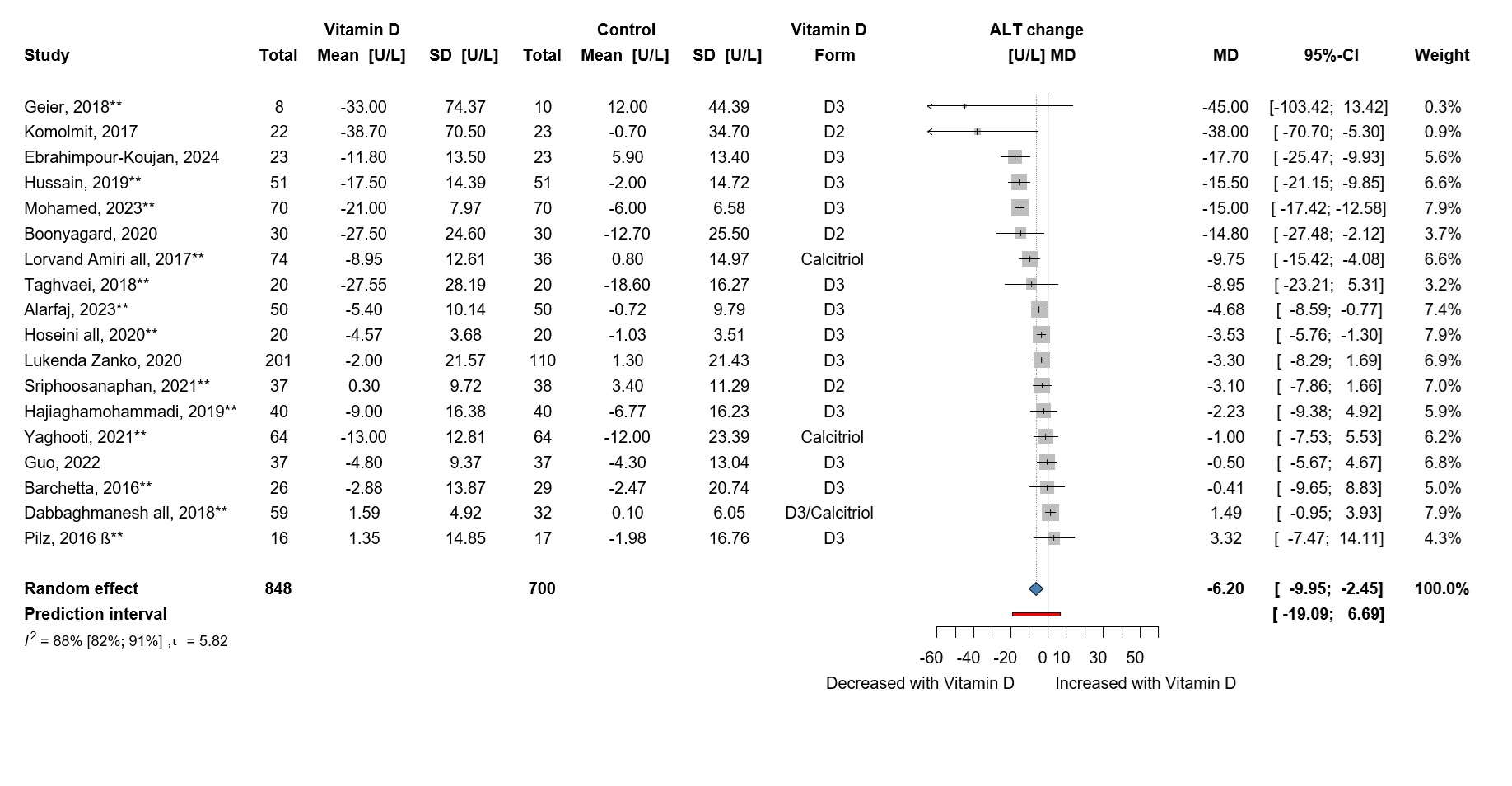


Figure S2.4: Forest plots showing ALT change in vitamin D and control groups excluding high-risk biased studies. ALT: alanine aminotransferase; CI: confidence interval; MD: mean difference; SD: standard deviation. If the study is indicated with **, then the change value is an estimated change value in that study. The β means that the mean and SD are estimated mean and SD in that study. See raw data and synthesis methods.


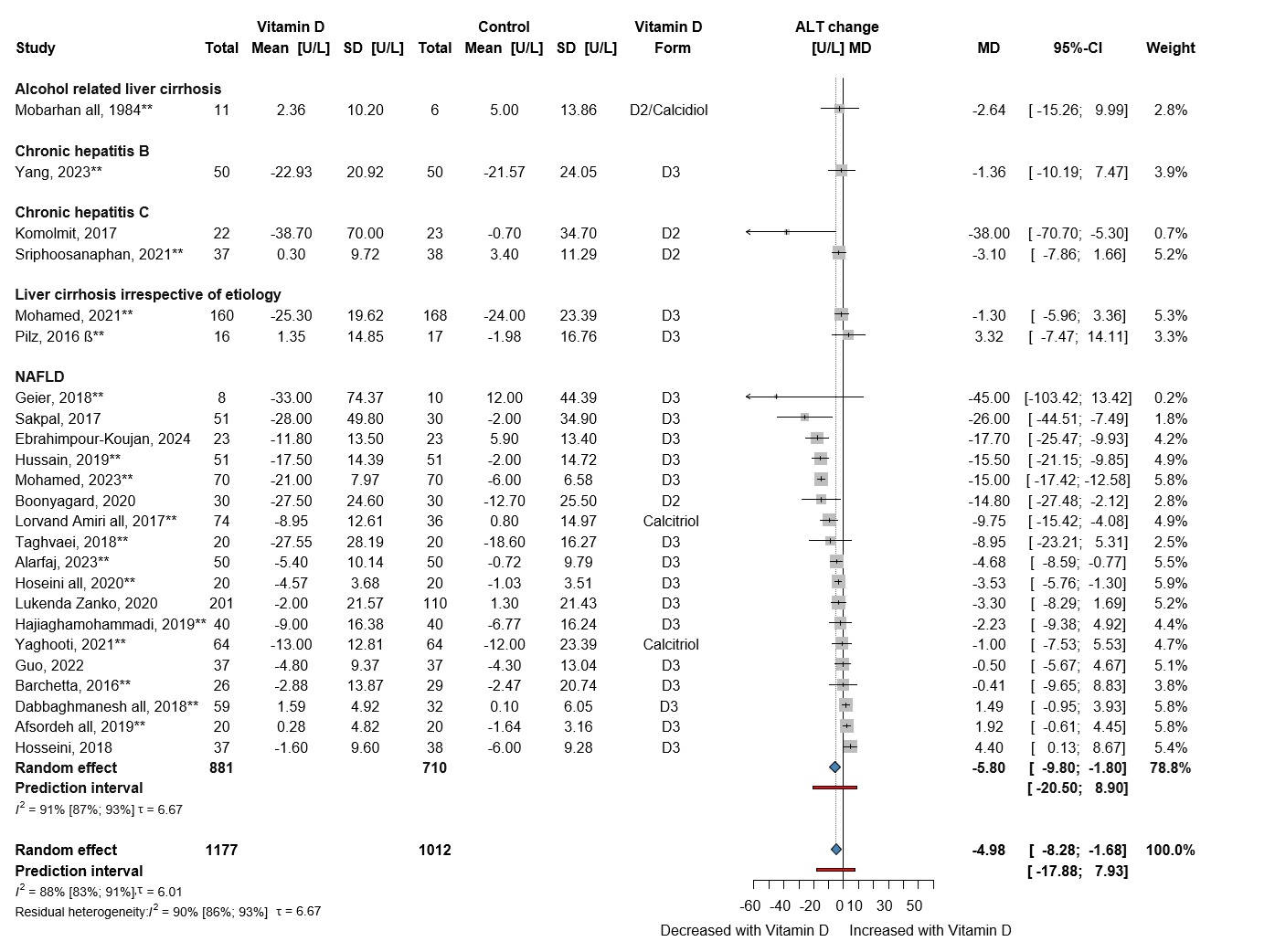


Figure S2.5: Forest plots showing ALT change in vitamin D and control groups by type of chronic liver disease. ALT: alanine aminotransferase; CI: confidence interval; MD: mean difference; NAFLD: Non-alcoholic fatty liver disease; SD: standard deviation. If the study is indicated with **, then the change value is an estimated change value in that study. The β means that the mean and SD are estimated mean and SD in that study. See raw data and synthesis methods.

***AST***


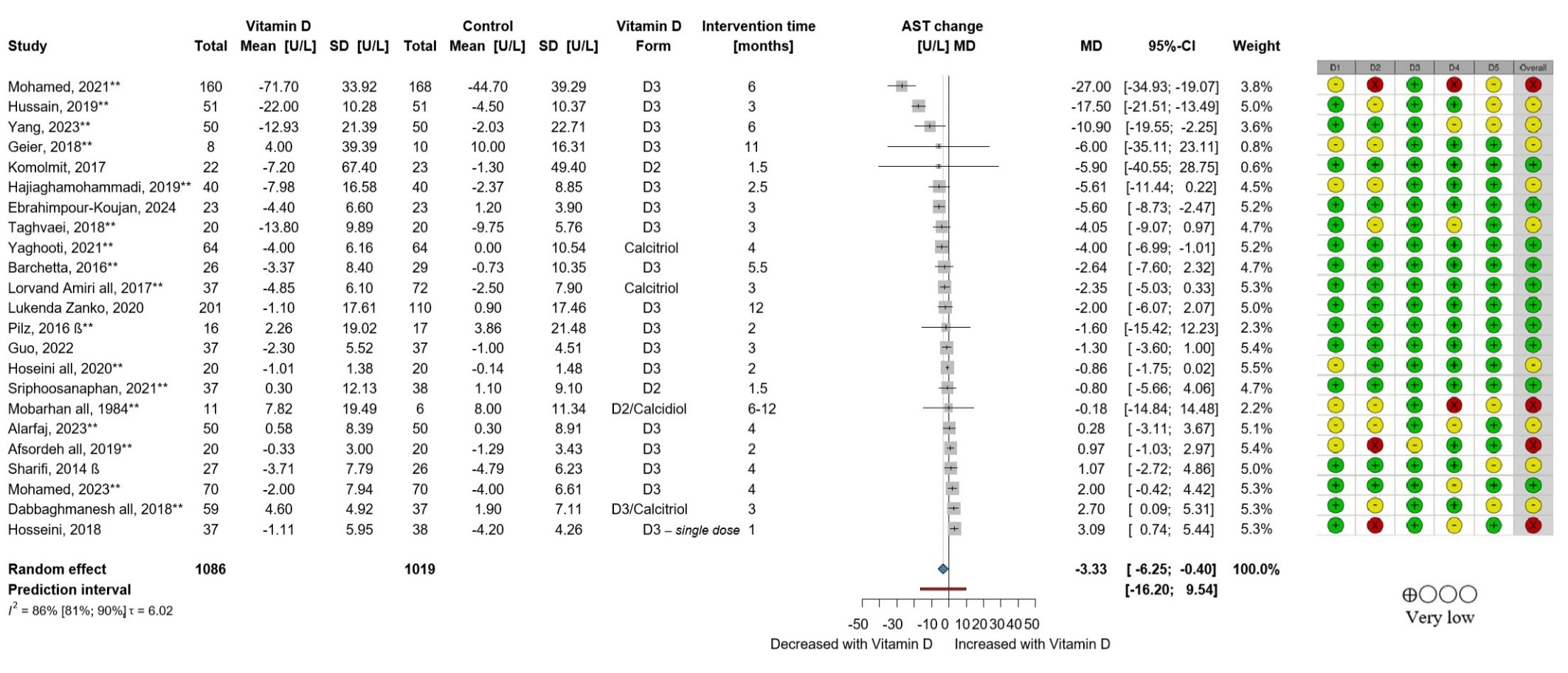


Figure S2.6a: Forest plots showing aspartate aminotransferase (AST) change in vitamin D and control groups. CI: confidence interval; MD: mean difference; SD: standard deviation. If the study is indicated with **, then the change value is an estimated change value in that study. The β means that the mean and SD are estimated mean and SD in that study. See raw data and synthesis methods.

Figure S2.6b: Funnel plot for aspartate aminotransferase (p = 0.0946).

In the case of AST, visual inspection did not confirm potential publication bias but rather indicated high heterogeneity: the effect sizes of large and medium-sized studies with small standard errors varied widely, resulting in an Egger's test p-value of less than 1%.

Figure S2.6c: Forest plot with leave-one-out analysis for aspartate aminotransferase.

Figure S2.6d: Baujat plot for aspartate aminotransferase.


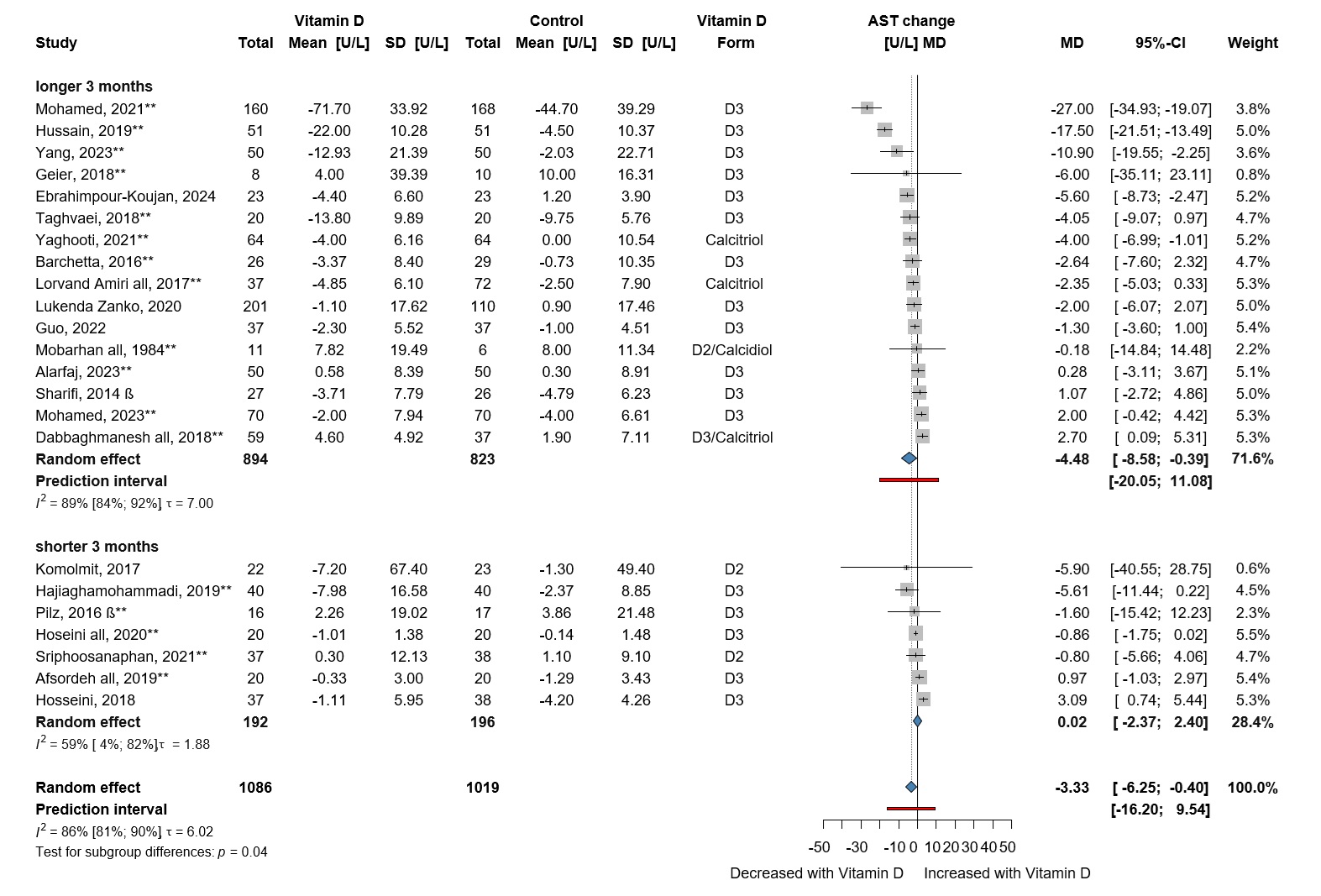


*Figure S2.7: Forest plots showing AST change in vitamin D and control groups by length of intervention. AST: aspartate aminotransferase; CI: confidence interval; MD: mean difference; SD: standard deviation. If the study is indicated with **, then the change value is an estimated change value in that study. The β means that the mean and SD are estimated mean and SD in that study. See raw data and synthesis methods.*


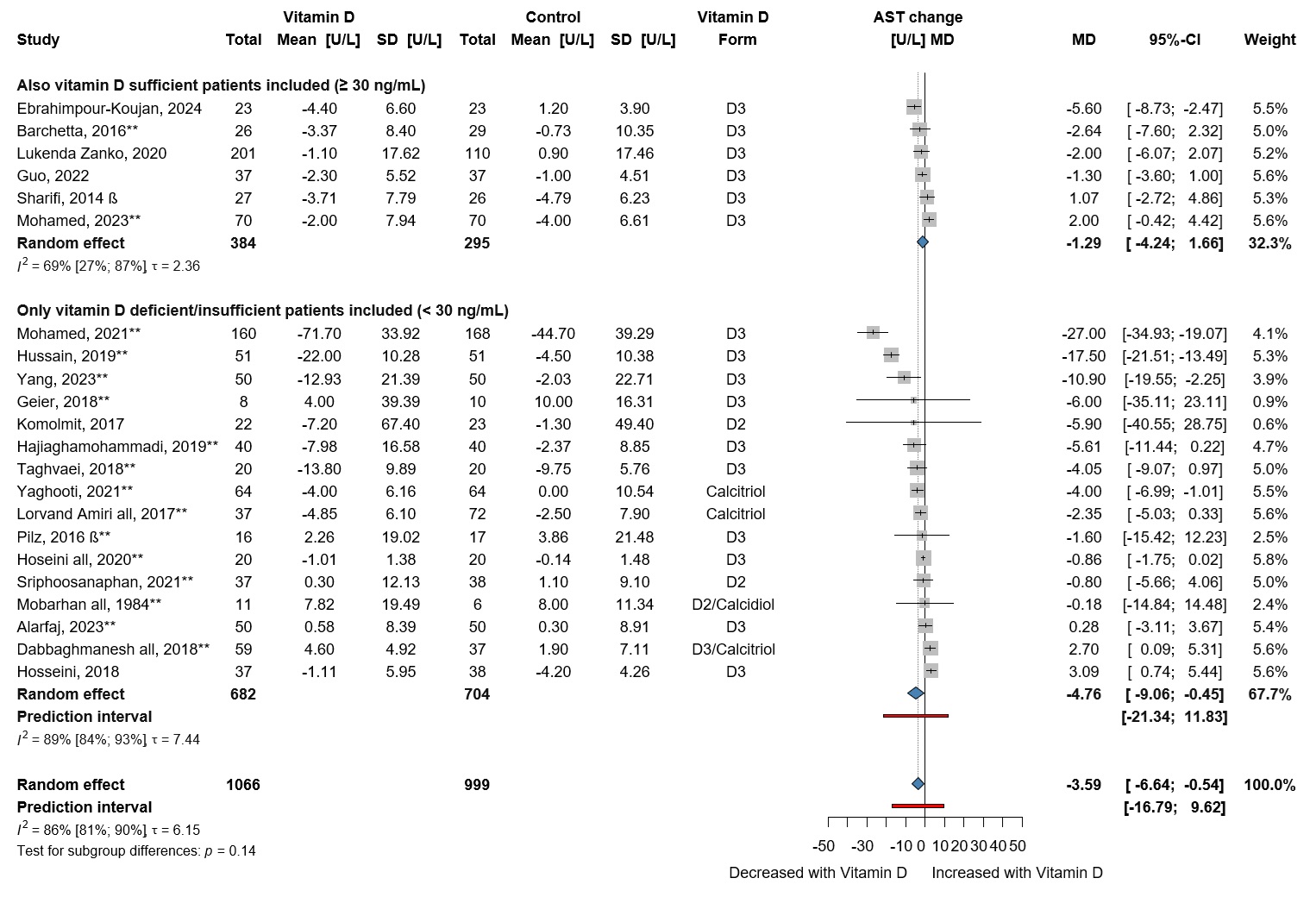


Figure S2.8: Forest plots showing AST change in vitamin D and control groups divided into vitamin D deficient/insufficient (> 30 ng/mL) and sufficient (≥ 30 ng/mL) studies. AST: aspartate aminotransferase; CI: confidence interval; MD: mean difference; SD: standard deviation. If the study is indicated with **, then the change value is an estimated change value in that study. The β means that the mean and SD are estimated mean and SD in that study. See raw data and synthesis methods.


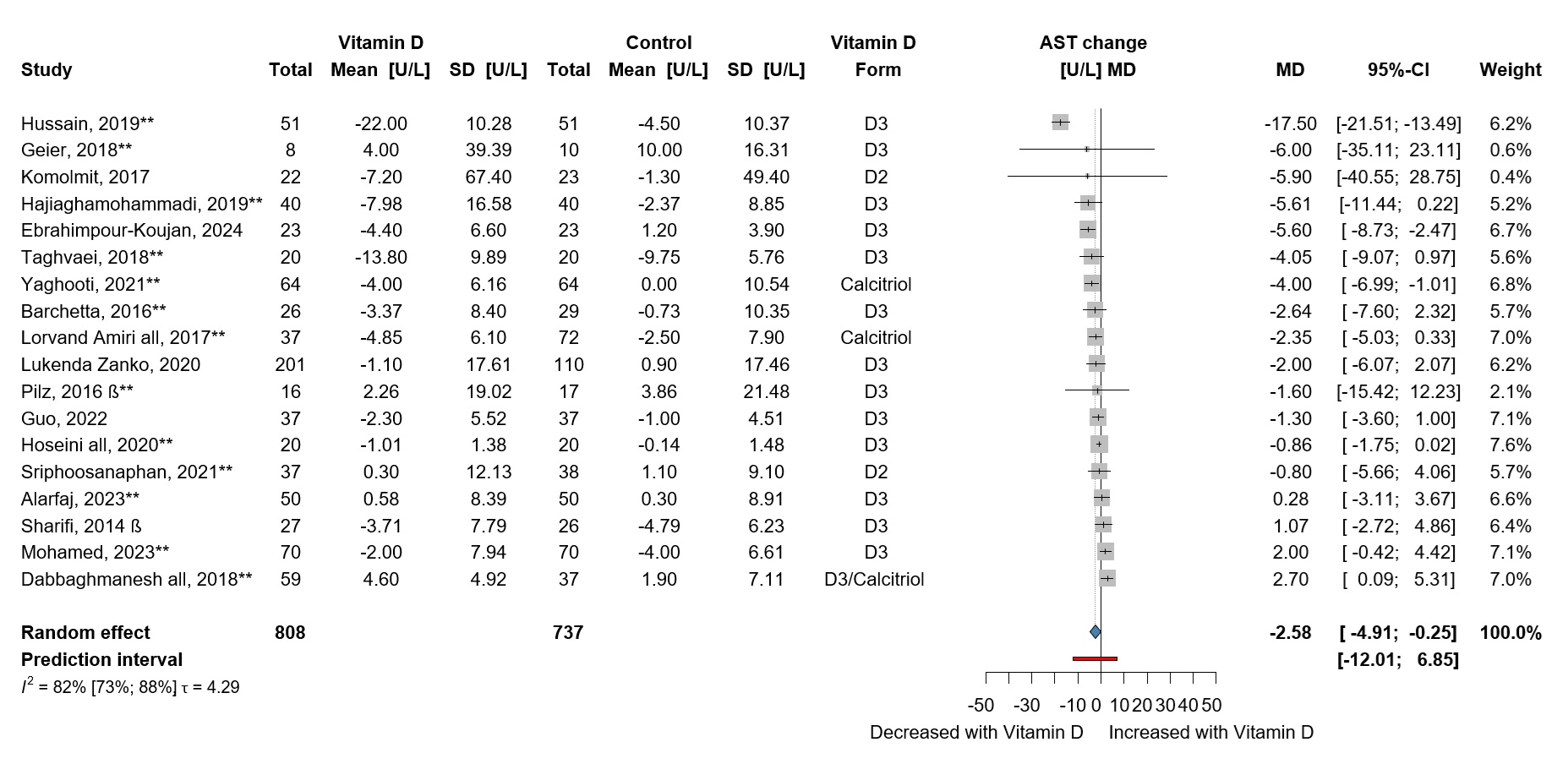


Figure S2.9: Forest plots showing AST change in vitamin D and control groups excluding high-risk biased studies. AST: aspartate aminotransferase; CI: confidence interval; MD: mean difference; SD: standard deviation. If the study is indicated with **, then the change value is an estimated change value in that study. The β means that the mean and SD are estimated mean and SD in that study. See raw data and synthesis methods.


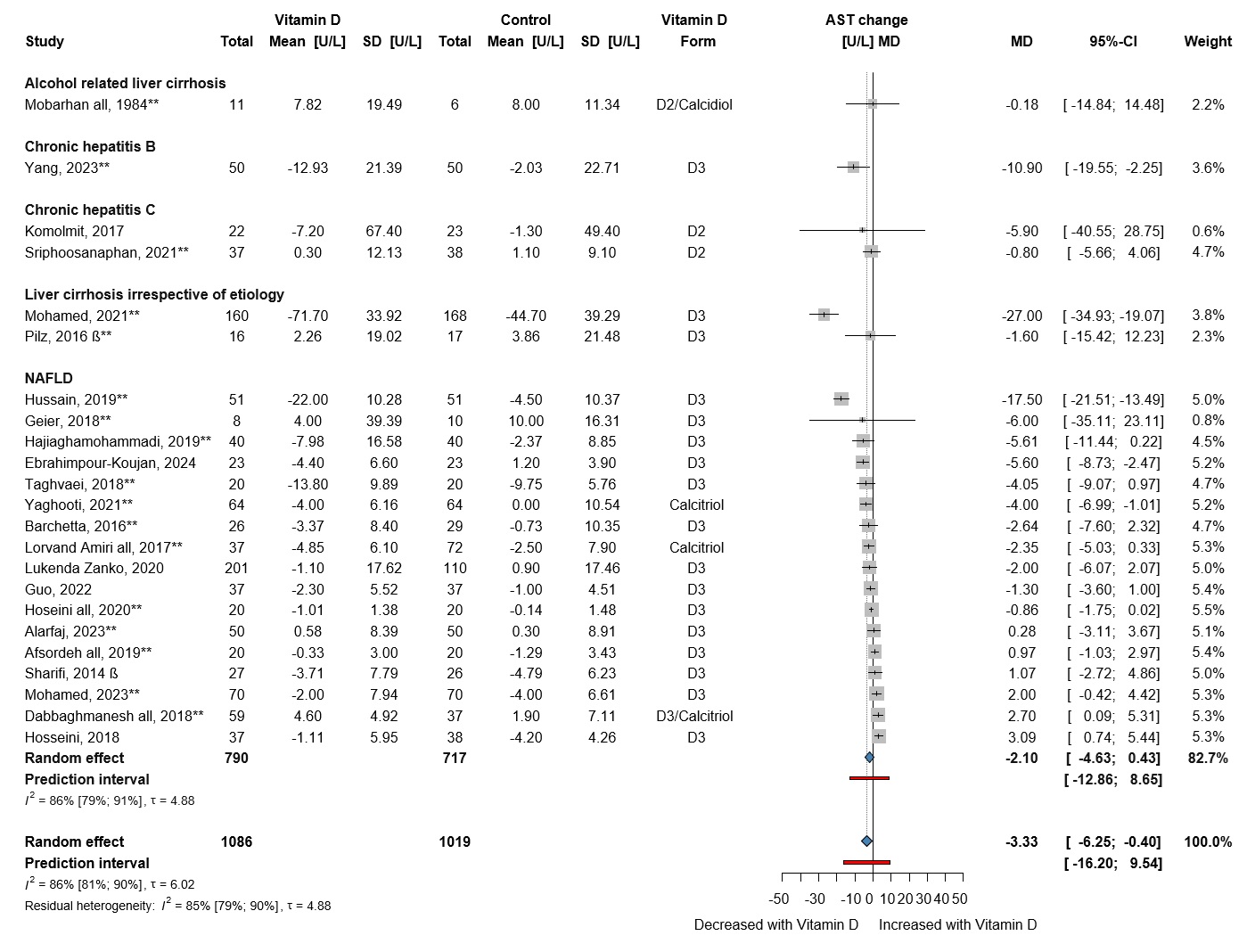


Figure S2.10: Forest plots showing AST change in vitamin D and control groups by type of chronic liver disease. AST: aspartate aminotransferase; CI: confidence interval; MD: mean difference; NAFLD: Non-alcoholic fatty liver disease; SD: standard deviation. If the study is indicated with **, then the change value is an estimated change value in that study. The β means that the mean and SD are estimated mean and SD in that study. See raw data and synthesis methods.

***GGT***


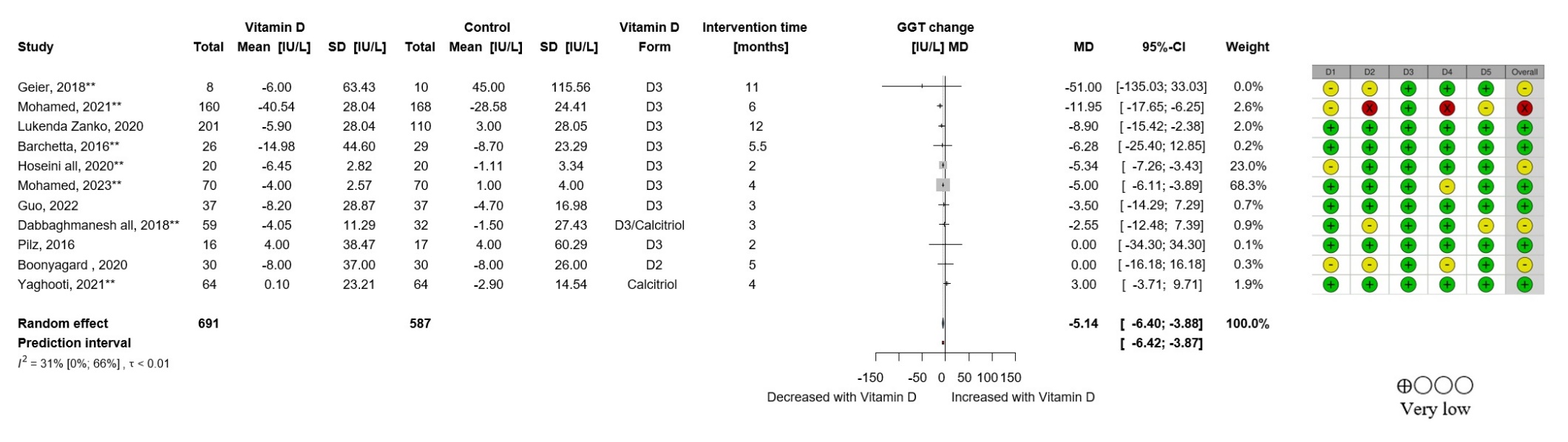


Figure S2.11a: Forest plots showing gamma-glutamyl transferase (GGT) change in vitamin D and control groups. CI: confidence interval; MD: mean difference; SD: standard deviation. If the study is indicated with **, then the change value is an estimated change value in that study. The β means that the mean and SD are estimated mean and SD in that study. See raw data and synthesis methods.

Figure S2.11b: Funnel plot for gamma-glutamyl transferase (p = 0.883).

Figure S2.11c: Forest plot with leave-one-out analysis for gamma-glutamyl transferase.

Figure S2.11d: Baujat plot for gamma-glutamyl transferase.


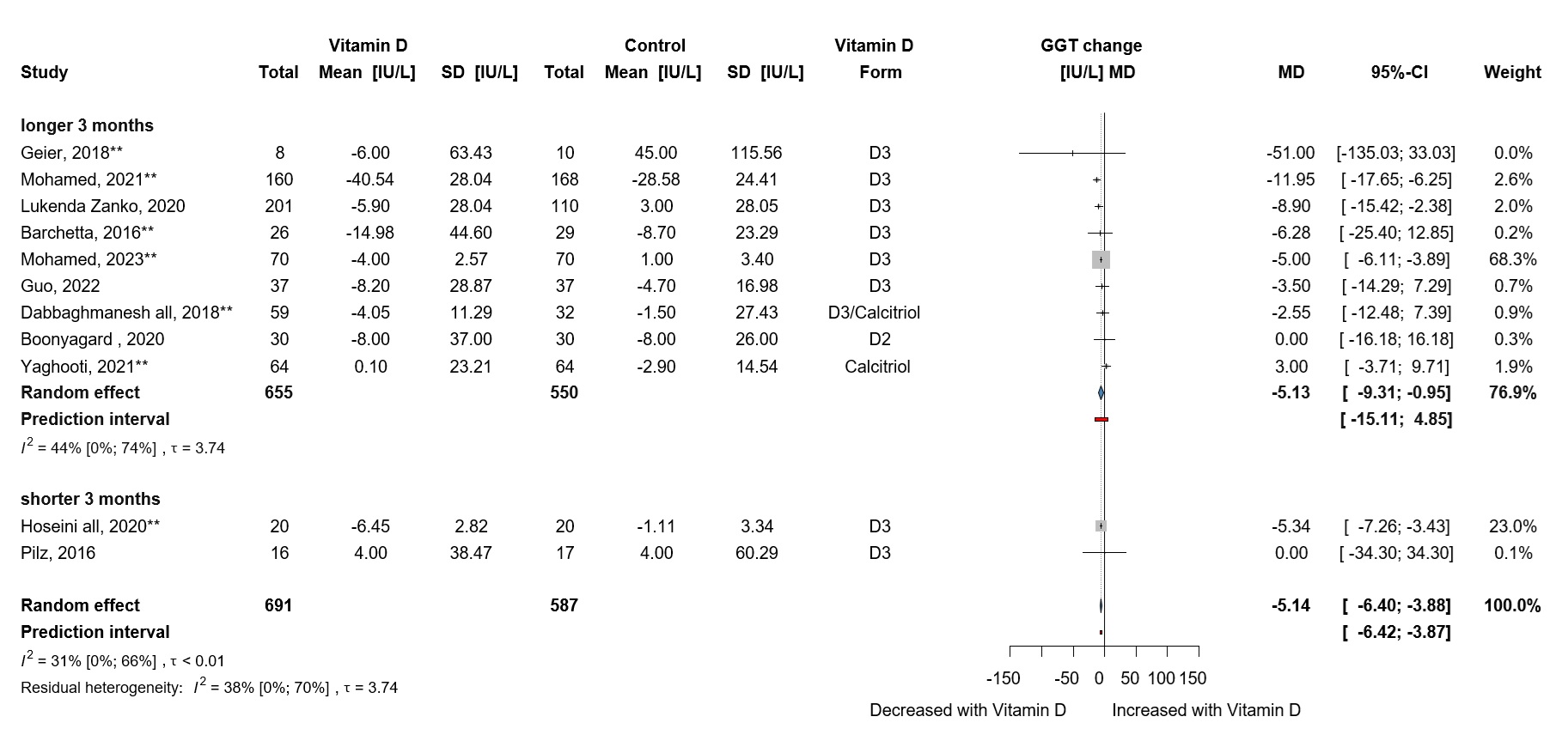


*Figure S2.12: Forest plots showing GGT change in vitamin D and control groups by length of intervention. CI: confidence interval;* *GGT: gamma-glutamyl transferase; MD: mean difference; SD: standard deviation. If the study is indicated with **, then the change value is an estimated change value in that study. The β means that the mean and SD are estimated mean and SD in that study. See raw data and synthesis methods.*


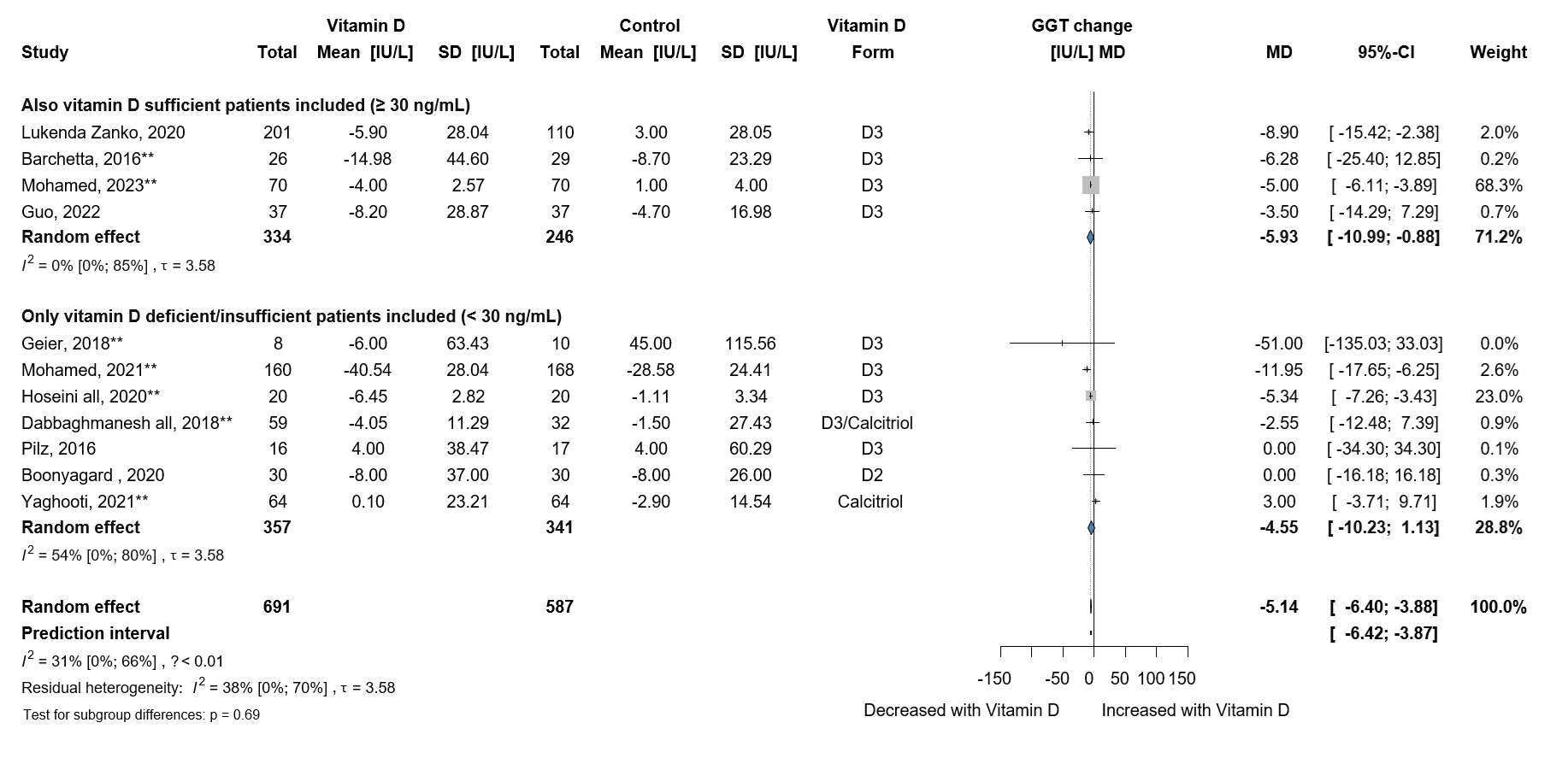


Figure S2.13: Forest plots showing GGT change in vitamin D and control groups divided into vitamin D deficient/insufficient (> 30 ng/mL) and sufficient (≥ 30 ng/mL) studies. CI: confidence interval; GGT: gamma-glutamyl transferase; MD: mean difference; SD: standard deviation. If the study is indicated with **, then the change value is an estimated change value in that study. The β means that the mean and SD are estimated mean and SD in that study. See raw data and synthesis methods.


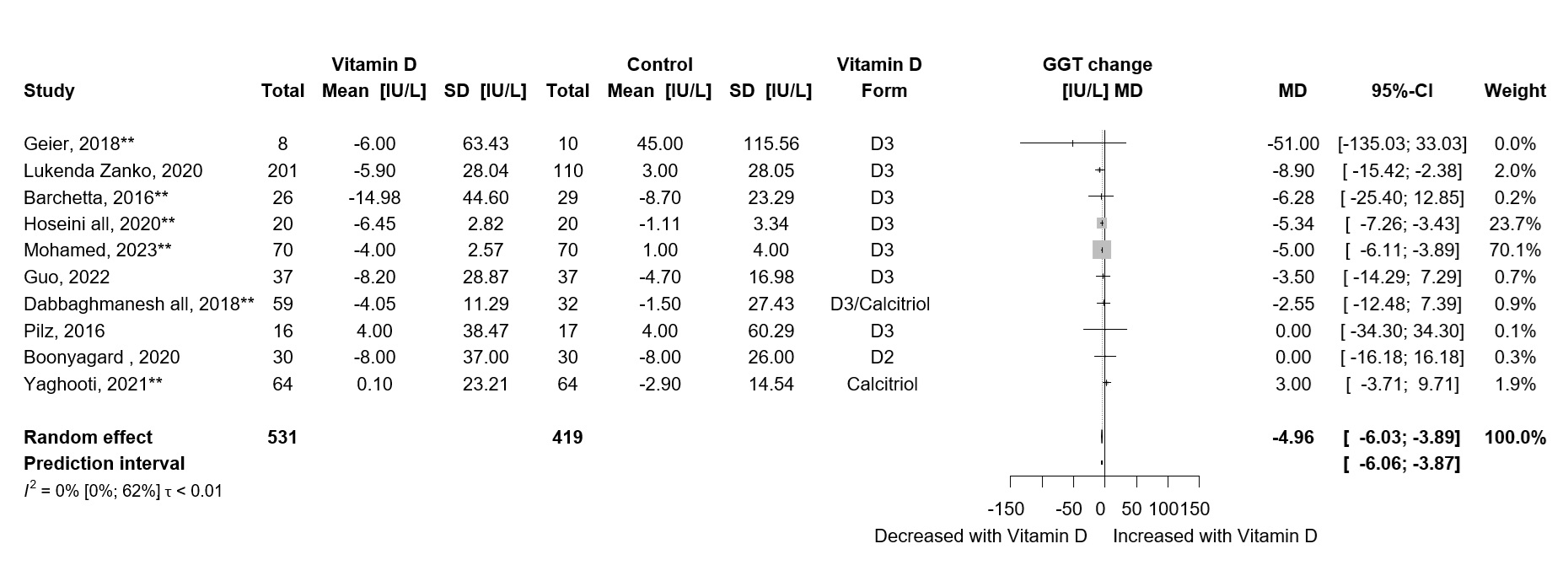


Figure S2.14: Forest plots showing GGT change in vitamin D and control groups excluding high-risk biased studies. CI: confidence interval; GGT: gamma-glutamyl transferase; MD: mean difference; SD: standard deviation. If the study is indicated with **, then the change value is an estimated change value in that study. The β means that the mean and SD are estimated mean and SD in that study. See raw data and synthesis methods.


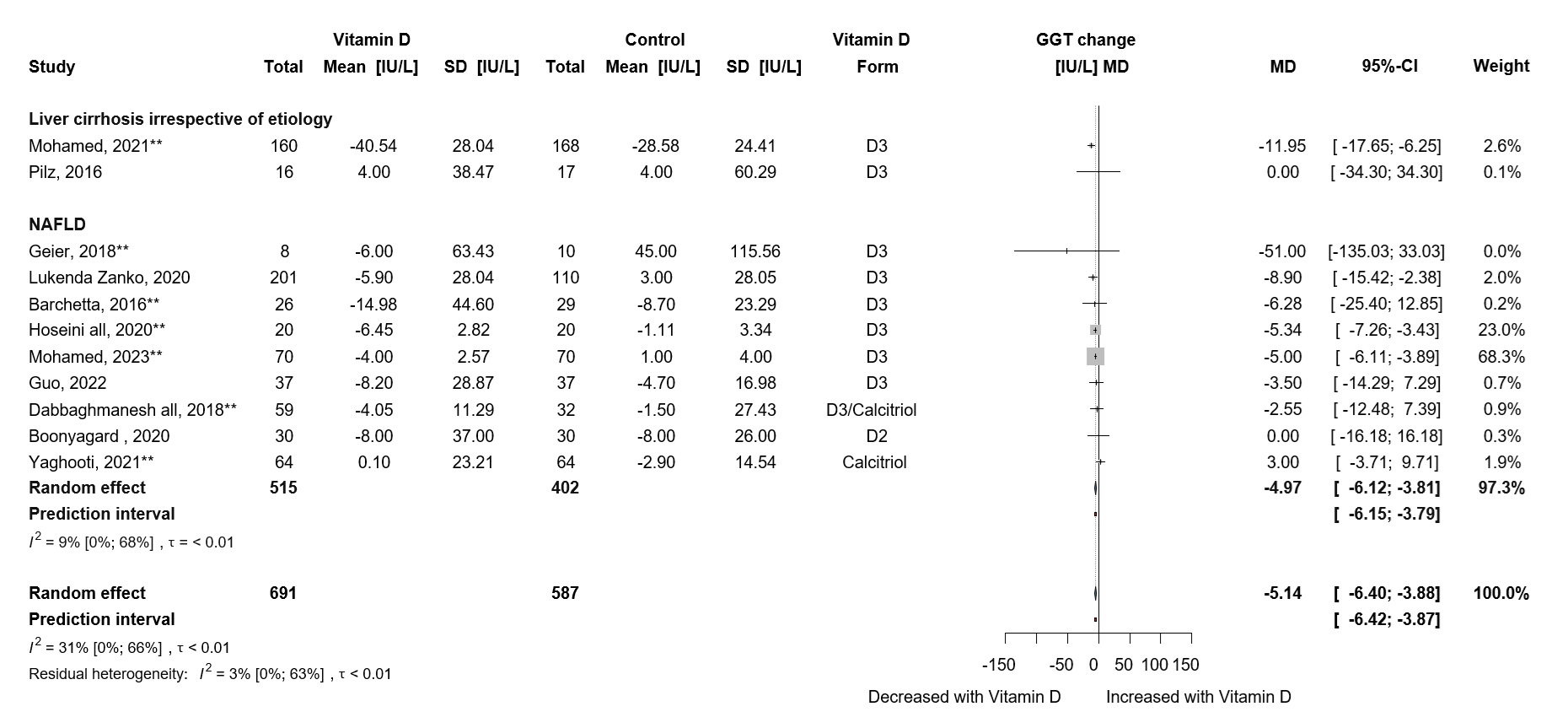


Figure S2.15: Forest plots showing GGT change in vitamin D and control groups by type of chronic liver disease. CI: confidence interval; GGT: gamma-glutamyl transferase; MD: mean difference; NAFLD: Non-alcoholic fatty liver disease; SD: standard deviation. If the study is indicated with **, then the change value is an estimated change value in that study. The β means that the mean and SD are estimated mean and SD in that study. See raw data and synthesis methods.

***ALP***


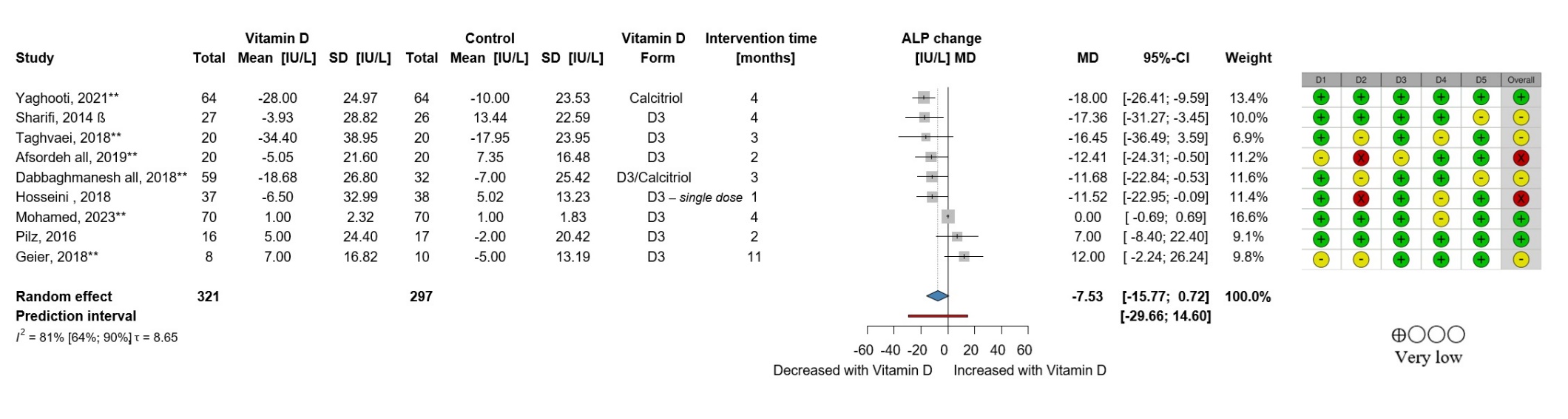


Figure S2.16a: Forest plots showing alkaline phosphatase (ALP) change in vitamin D and control groups. CI: confidence interval; MD: mean difference; SD: standard deviation. If the study is indicated with **, then the change value is an estimated change value in that study. The β means that the mean and SD are estimated mean and SD in that study. See raw data and synthesis methods.

Figure S2.16b: Funnel plot for alkaline phosphatase (p = 0.0693).

Figure S2.16c: Forest plot with leave-one-out analysis for alkaline phosphatase.

Figure S2.16d: Baujat plot for alkaline phosphatase.


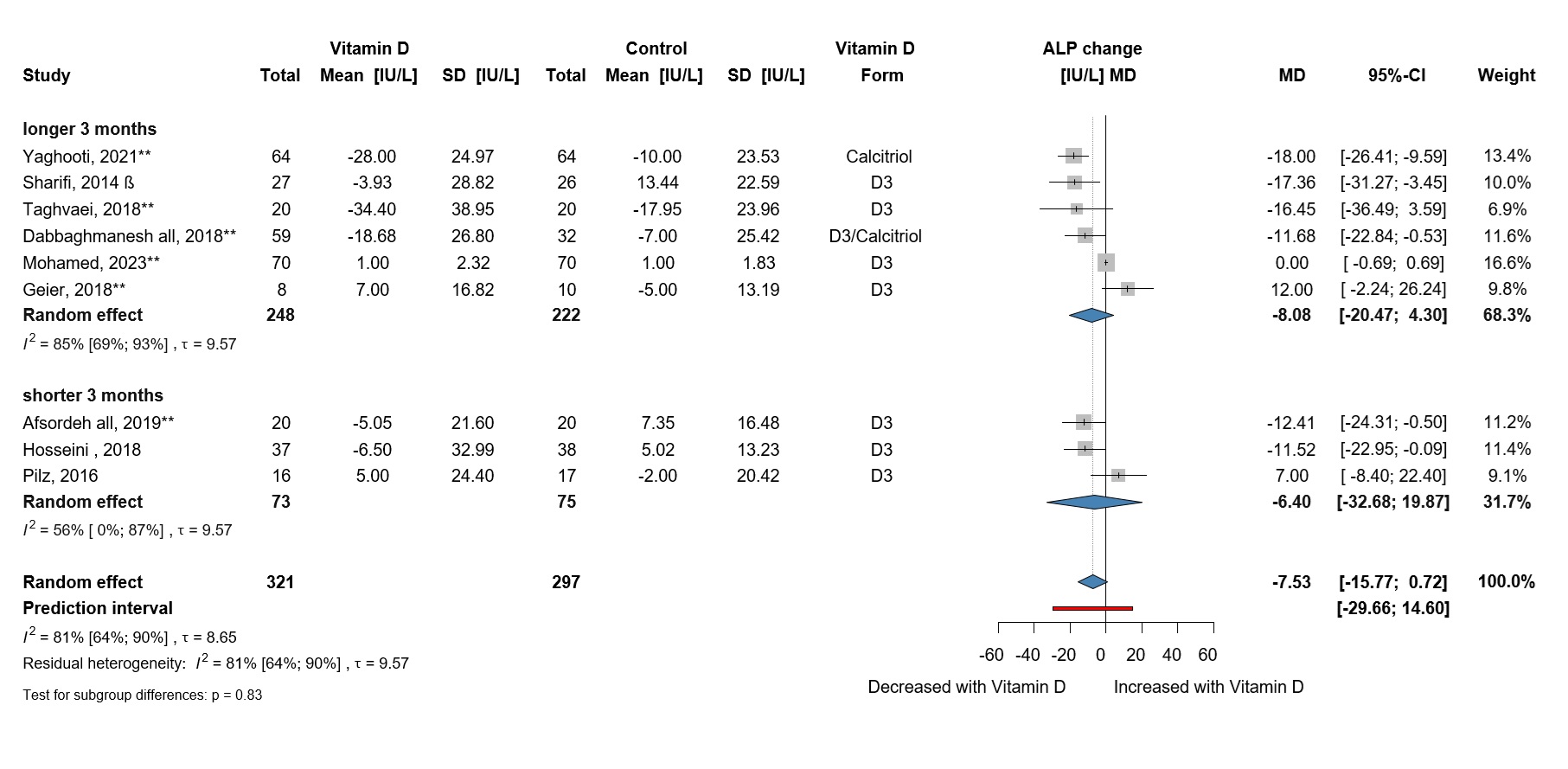


Figure S2.17: Forest plots showing ALP change in vitamin D and control groups by length of intervention. ALP: alkaline phosphatase; CI: confidence interval; MD: mean difference; SD: standard deviation. If the study is indicated with **, then the change value is an estimated change value in that study. The β means that the mean and SD are estimated mean and SD in that study. See raw data and synthesis methods.


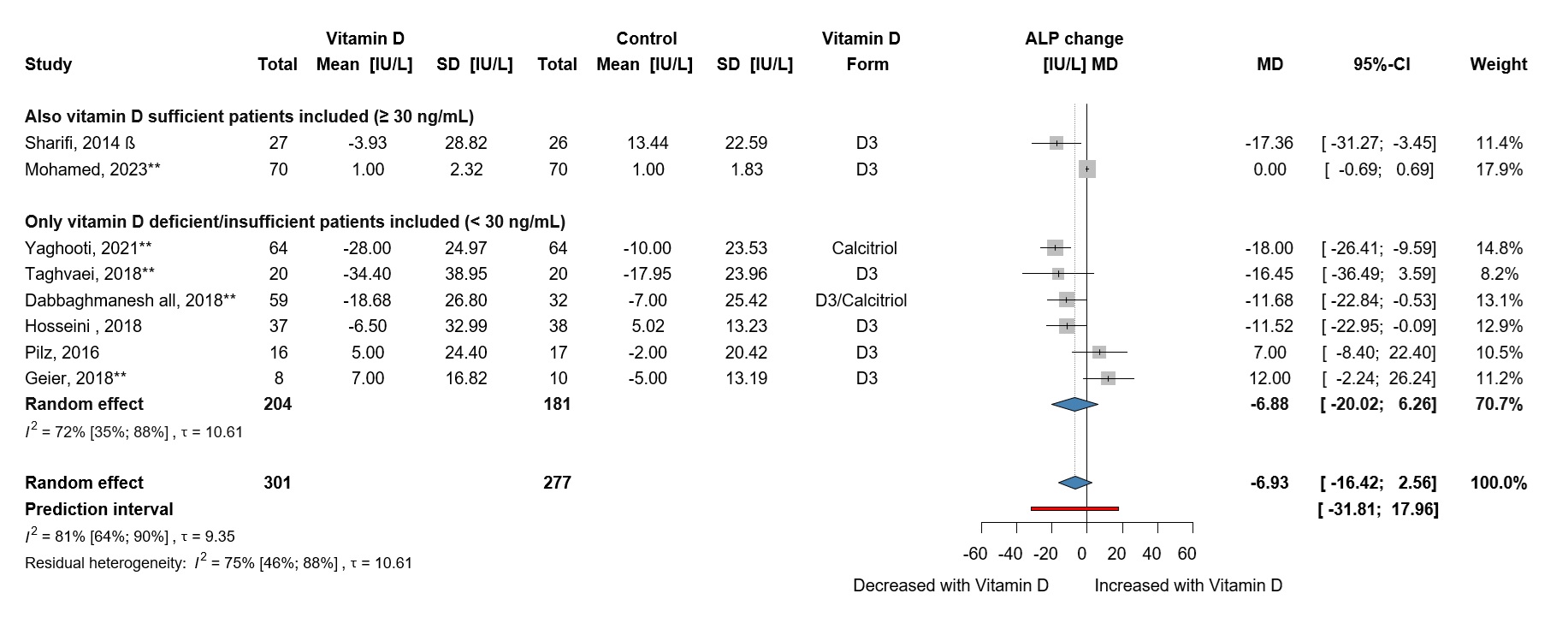


Figure S2.18: Forest plots showing ALP change in vitamin D and control groups divided into vitamin D deficient/insufficient (> 30 ng/mL) and sufficient (≥ 30 ng/mL) studies. ALP: alkaline phosphatase; CI: confidence interval; MD: mean difference; SD: standard deviation. If the study is indicated with **, then the change value is an estimated change value in that study. The β means that the mean and SD are estimated mean and SD in that study. See raw data and synthesis methods.


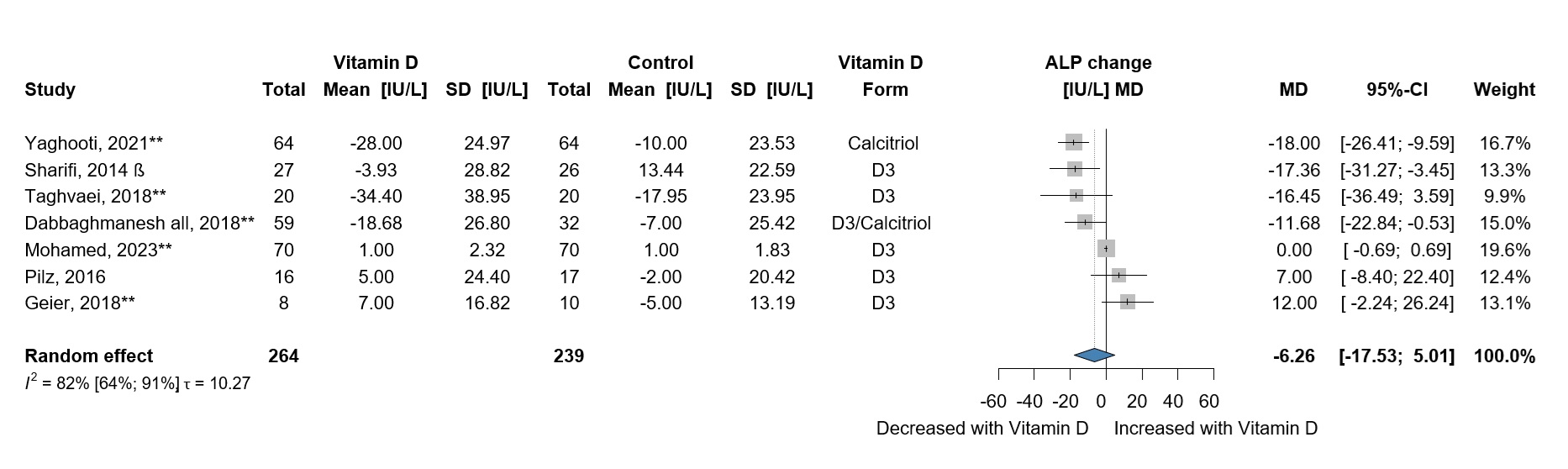


Figure S2.19: Forest plot showing ALP change in vitamin D and control groups excluding high-risk biased studies. ALP: alkaline phosphatase; CI: confidence interval; MD: mean difference; SD: standard deviation. If the study is indicated with **, then the change value is an estimated change value in that study. The β means that the mean and SD are estimated mean and SD in that study. See raw data and synthesis methods.


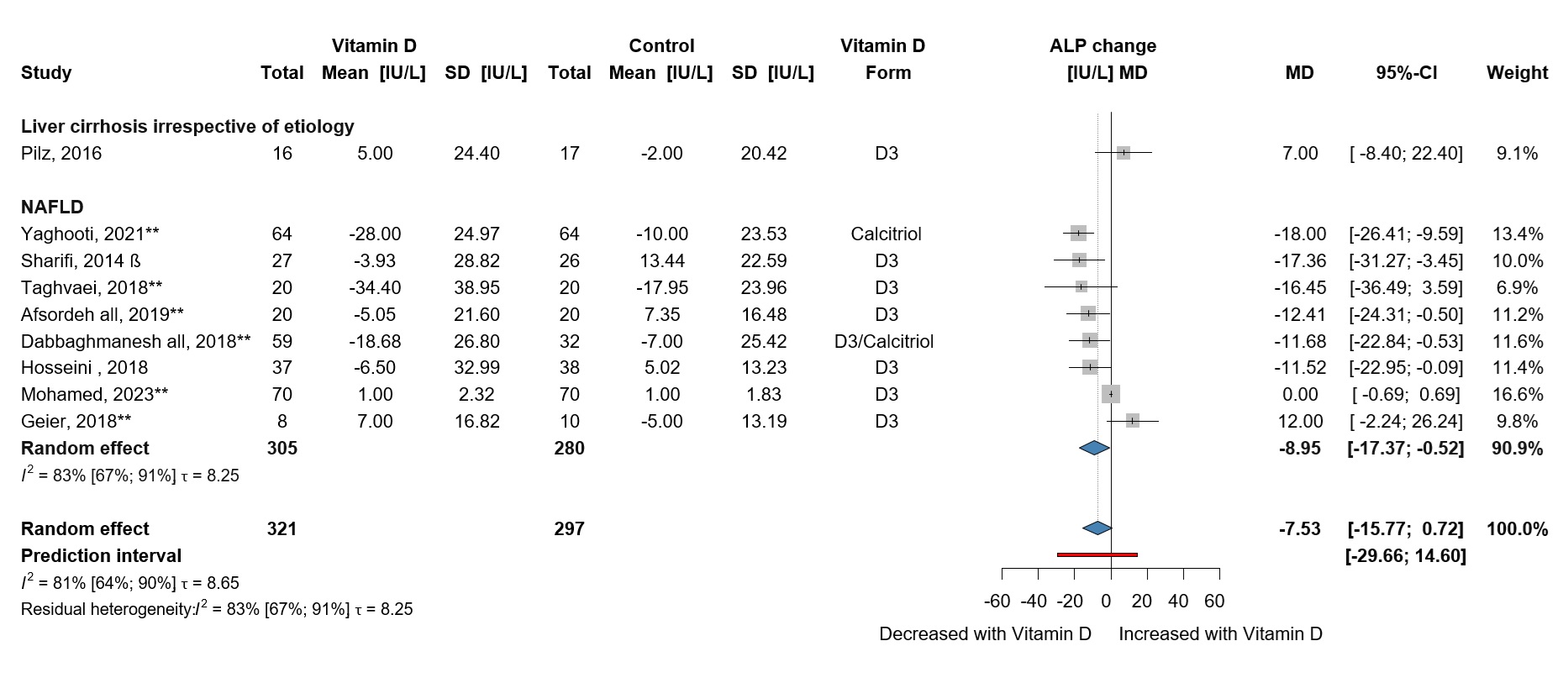


Figure S2.20: Forest plot showing ALP change in vitamin D and control groups by type of chronic liver disease. ALP: alkaline phosphatase; CI: confidence interval; MD: mean difference; NAFLD: Non-alcoholic fatty liver disease; SD: standard deviation. If the study is indicated with **, then the change value is an estimated change value in that study. The β means that the mean and SD are estimated mean and SD in that study. See raw data and synthesis methods.

**Table S5**. Baseline level of liver enzymes in the included studies.


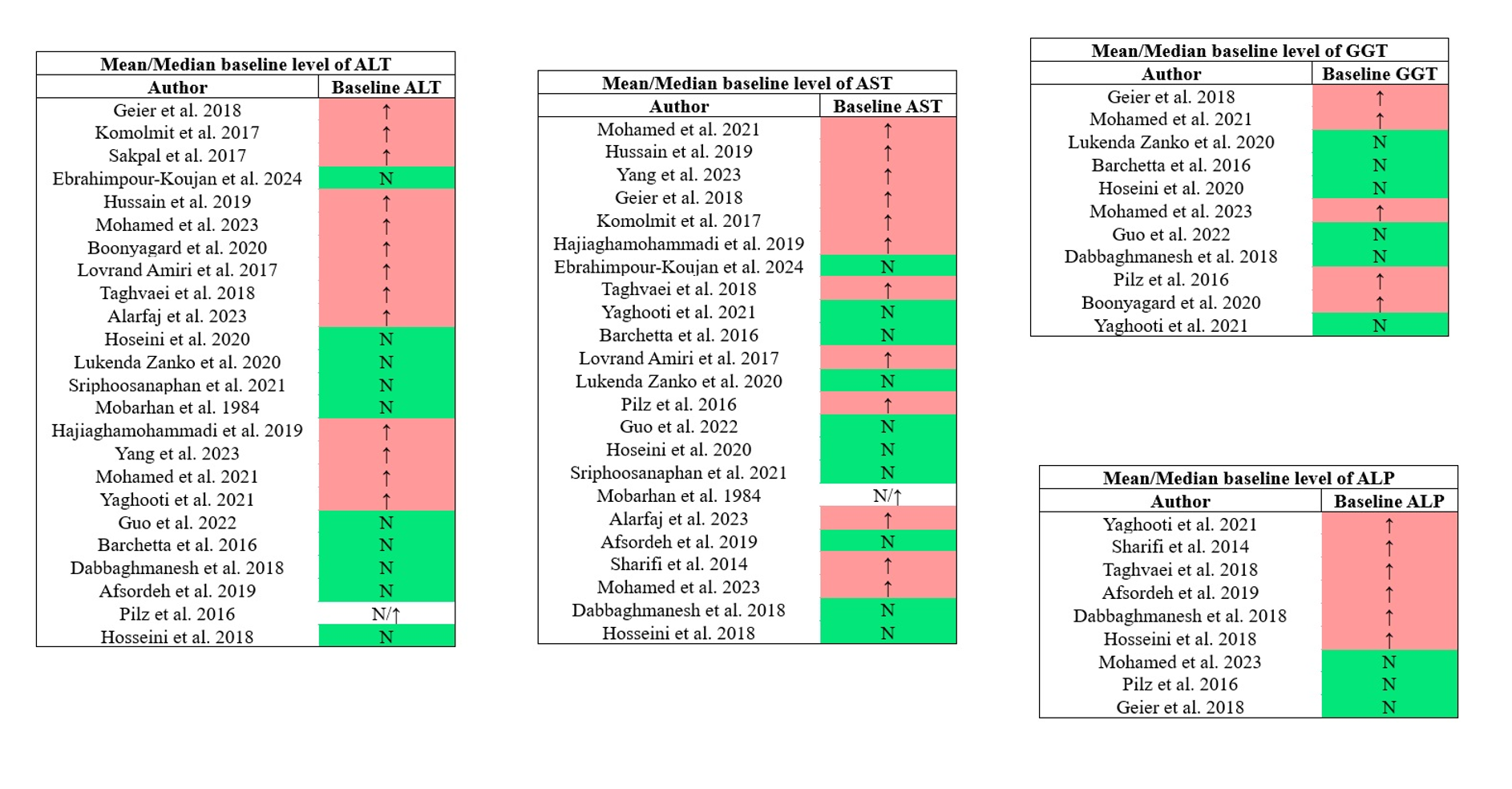


**Table S5.** Baseline ALT/AST/GGT/ALP level expressed as mean or median as reported in the included studies.

*N: Normal liver enzyme level (ALT: ≤ 36 IU/L; AST: ≤ 30 IU/L; GGT: ≤ 50 IU/L; ALP: ≤ 120 IU/L) in both groups; ↑: Increased liver enzyme level (ALT: > 36 IU/L; AST: > 30 IU/L; GGT: > 50 IU/L; ALP: > 120 IU/L) in both groups; N/↑: Increased liver enzyme level (ALT: > 36 IU/L; AST: > 30 IU/L; GGT: > 50 IU/L; ALP: > 120 IU/L) in one group and normal liver enzyme level (ALT: ≤ 36 IU/L; AST: ≤ 30 IU/L; GGT: ≤ 50 IU/L; ALP: ≤ 120 IU/L) in another group.*
