# Supplementary material for: Role of Vitamin D Supplementation in Chronic Liver Disease: A Systematic Review and Meta-Analysis of Randomized Controlled Trials
Source: Nutr Rev. 2025 Jul 11;83(11):2043–54. doi: 10.1093/nutrit/nuaf117 (PMC12512233; doi:10.1093/nutrit/nuaf117)
Supplement: nuaf117_Supplementary_Data [file nuaf117_supplementary_data.zip › Supplementary file S3-S4.docx]

**SUPPLEMENTARY MATERIAL – SUPPLEMENTARY FILE S3-S4**

**SUPPLEMENTARY FILE S3-S4.** Glucose and lipid metabolism

**FIGURE AND TABLE LEGENDS**

**SUPPLEMENTARY FILE S3.** Glucose metabolism.

Figure S3.1a. Forest plot showing homeostatic model assessment for insulin resistance (HOMA-IR) change in vitamin D and control groups.

Figure S3.1b. Funnel plot for HOMA-IR.

Figure S3.1c. Forest plot with leave-one-out analysis for HOMA-IR.

Figure S3.1d. Baujat plot for HOMA-IR.

Figure S3.2. Forest plot showing HOMA-IR change in vitamin D and control groups by length of intervention.

Figure S3.3. Forest plot showing HOMA-IR change in vitamin D and control groups divided into vitamin D deficient/insufficient (< 30 ng/mL) and sufficient (≥ 30 ng/mL) studies.

Figure S3.4. Forest plot showing HOMA-IR change in vitamin D and control groups excluding high-risk biased studies.

Figure S3.5. Forest plot showing HOMA-IR change in vitamin D and control groups by type of chronic liver disease.

Figure S3.6a. Forest plot showing insulin change in vitamin D and control groups excluding Alarfaj article.

Figure S3.6b. Forest plot showing insulin change in vitamin D and control groups including Alarfaj article.

Figure S3.6c. Funnel plot for insulin.

Figure S3.6d. Forest plot with leave-one-out analysis for insulin.

Figure S3.6e. Baujat plot for insulin.

Figure S3.7. Forest plot showing insulin change in vitamin D and control groups by length of intervention.

Figure S3.8. Forest plot showing insulin change in vitamin D and control groups divided into vitamin D deficient/insufficient (< 30 ng/mL) and sufficient (≥ 30 ng/mL) studies.

Figure S3.9. Forest plot showing insulin change in vitamin D and control groups excluding high-risk biased studies.

Figure S3.10. Forest plot showing insulin change in vitamin D and control groups by type of chronic liver disease.

Figure S3.11a. Forest plot showing fasting plasma glucose (FPG) change in vitamin D and control groups.

Figure S3.11b. Funnel plot for FPG.

Figure S3.11c. Forest plot with leave-one-out analysis for FPG.

Figure S3.11d. Baujat plot for FPG.

Figure S3.12. Forest plot showing FPG change in vitamin D and control groups by length of intervention.

Figure S3.13. Forest plot showing FPG change in vitamin D and control groups divided into vitamin D deficient/insufficient (< 30 ng/mL) and sufficient (≥ 30 ng/mL) studies.

Figure S3.14. Forest plot showing FPG change in vitamin D and control groups excluding high-risk biased studies.

Figure S3.15. Forest plot showing FPG change in vitamin D and control groups by type of chronic liver disease.

**SUPPLEMENTARY FILE S4.** Lipid metabolism.

Figure S4.1a. Forest plot showing total cholesterol change in vitamin D and control groups.

Figure S4.1b. Funnel plot for total cholesterol.

Figure S4.1c. Forest plot with leave-one-out analysis for total cholesterol.

Figure S4.1d. Baujat plot for total cholesterol.

Figure S4.2. Forest plot showing total cholesterol change in vitamin D and control groups by length of intervention.

Figure S4.3. Forest plot showing total cholesterol change in vitamin D and control groups divided into vitamin D deficient/insufficient (< 30 ng/mL) and sufficient (≥ 30 ng/mL) studies.

Figure S4.4. Forest plot showing total cholesterol change in vitamin D and control groups excluding high-risk biased studies.

Figure S4.5. Forest plot showing total cholesterol change in vitamin D and control groups by type of chronic liver disease.

Figure S4.6a. Forest plot showing low-density cholesterol (LDL) change in vitamin D and control groups.

Figure S4.6b. Funnel plot for LDL.

Figure S4.6c. Forest plot with leave-one-out analysis for LDL.

Figure S4.6d. Baujat plot for LDL.

Figure S4.7. Forest plot showing LDL change in vitamin D and control groups by length of intervention.

Figure S4.8. Forest plot showing LDL change in vitamin D and control groups divided into vitamin D deficient/insufficient (< 30 ng/mL) and sufficient (≥ 30 ng/mL) studies.

Figure S4.9. Forest plot showing LDL change in vitamin D and control groups excluding high-risk biased studies.

Figure S4.10. Forest plot showing LDL change in vitamin D and control groups by type of chronic liver disease.

Figure S4.11a. Forest plot showing high-density cholesterol (HDL) change in vitamin D and control groups.

Figure S4.11b. Funnel plot for HDL.

Figure S4.11c. Forest plot with leave-one-out analysis for HDL.

Figure S4.11d. Baujat plot for HDL.

Figure S4.12. Forest plot showing HDL change in vitamin D and control groups by length of intervention.

Figure S4.13. Forest plot showing HDL change in vitamin D and control groups divided into vitamin D deficient/insufficient (< 30 ng/mL) and sufficient (≥ 30 ng/mL) studies.

Figure S4.14. Forest plot showing HDL change in vitamin D and control groups excluding high-risk biased studies.

Figure S4.15. Forest plot showing HDL change in vitamin D and control groups by type of chronic liver disease.

Figure S4.16a. Forest plot showing total triglycerides change in vitamin D and control groups.

Figure S4.16b. Funnel plot for total triglycerides.

Figure S4.16c. Forest plot with leave-one-out analysis for total triglycerides.

Figure S4.16d. Baujat plot for total triglycerides.

Figure S4.17. Forest plot showing total triglycerides change in vitamin D and control groups by length of intervention.

Figure S4.18. Forest plot showing total triglycerides change in vitamin D and control groups divided into vitamin D deficient/insufficient (< 30 ng/mL) and sufficient (≥ 30 ng/mL) studies.

Figure S4.19. Forest plot showing total triglycerides change in vitamin D and control groups excluding high-risk biased studies.

Figure S4.20. Forest plot showing total triglycerides change in vitamin D and control groups by type of chronic liver disease.

Figure S4.21a. Forest plot showing adiponectin change in vitamin D and control groups.

Figure S.21b. Funnel plot for adiponectin.

Figure S4.21c. Forest plot with leave-one-out analysis for adiponectin.

Figure S4.21d. Baujat plot for adiponectin.

Figure S4.22. Forest plot showing adiponectin change in vitamin D and control groups by length of intervention.

Figure S4.23. Forest plot showing adiponectin change in vitamin D and control groups divided into vitamin D deficient/insufficient (< 30 ng/mL) and sufficient (≥ 30 ng/mL) studies.

Figure S4.24. Forest plot showing adiponectin change in vitamin D and control groups by type of chronic liver disease.

**Supplementary File S3.** Glucose metabolism.


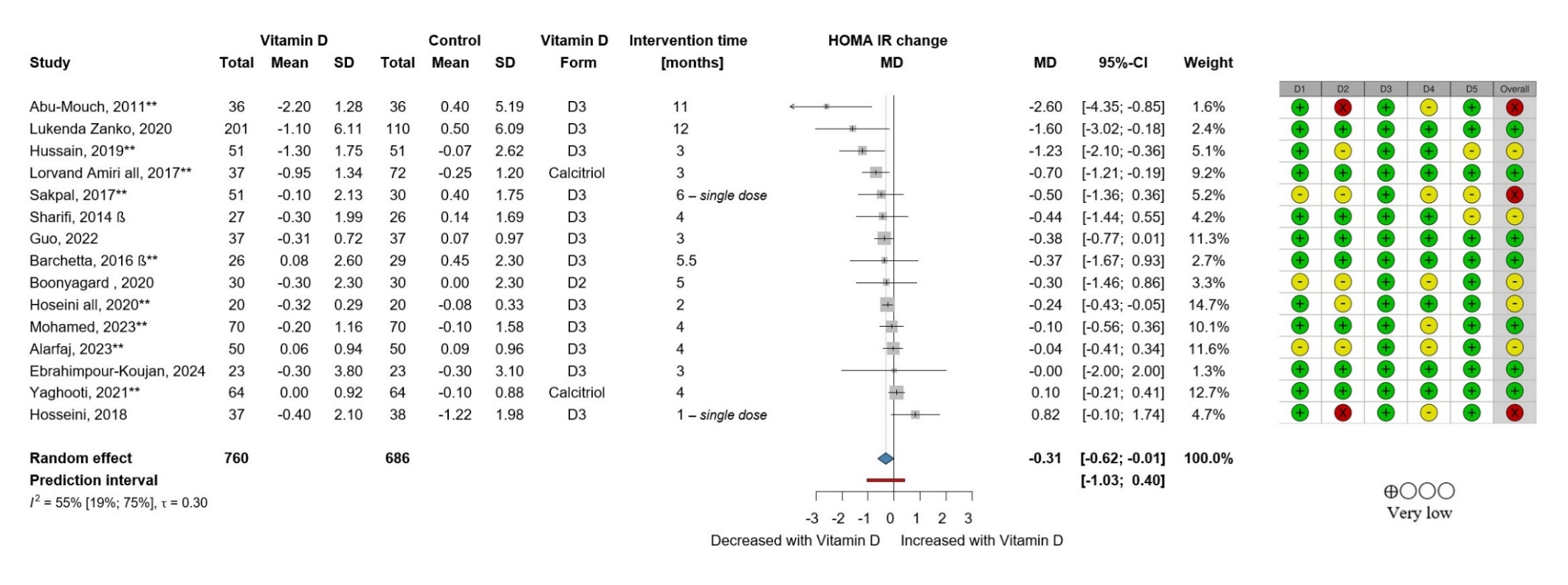


*Figure S3.1a. Forest plot showing homeostatic model assessment for insulin resistance (HOMA-IR) change in vitamin D and control groups. CI: confidence interval; HOMA-IR: homeostatic model assessment for insulin resistance; MD: mean difference; SD: standard deviation. If the study is indicated with **, then the change value is an estimated change value in that study. The β means that the mean and SD are estimated mean and SD in that study. See raw data and synthesis methods.*

*Figure S3.1b. Funnel plot for HOMA-IR (p = 0.1617).*

*Figure S3.1c. Forest plot with leave-one-out analysis for HOMA-IR.*

**

*Figure S3.1d. Baujat plot for HOMA-IR.*


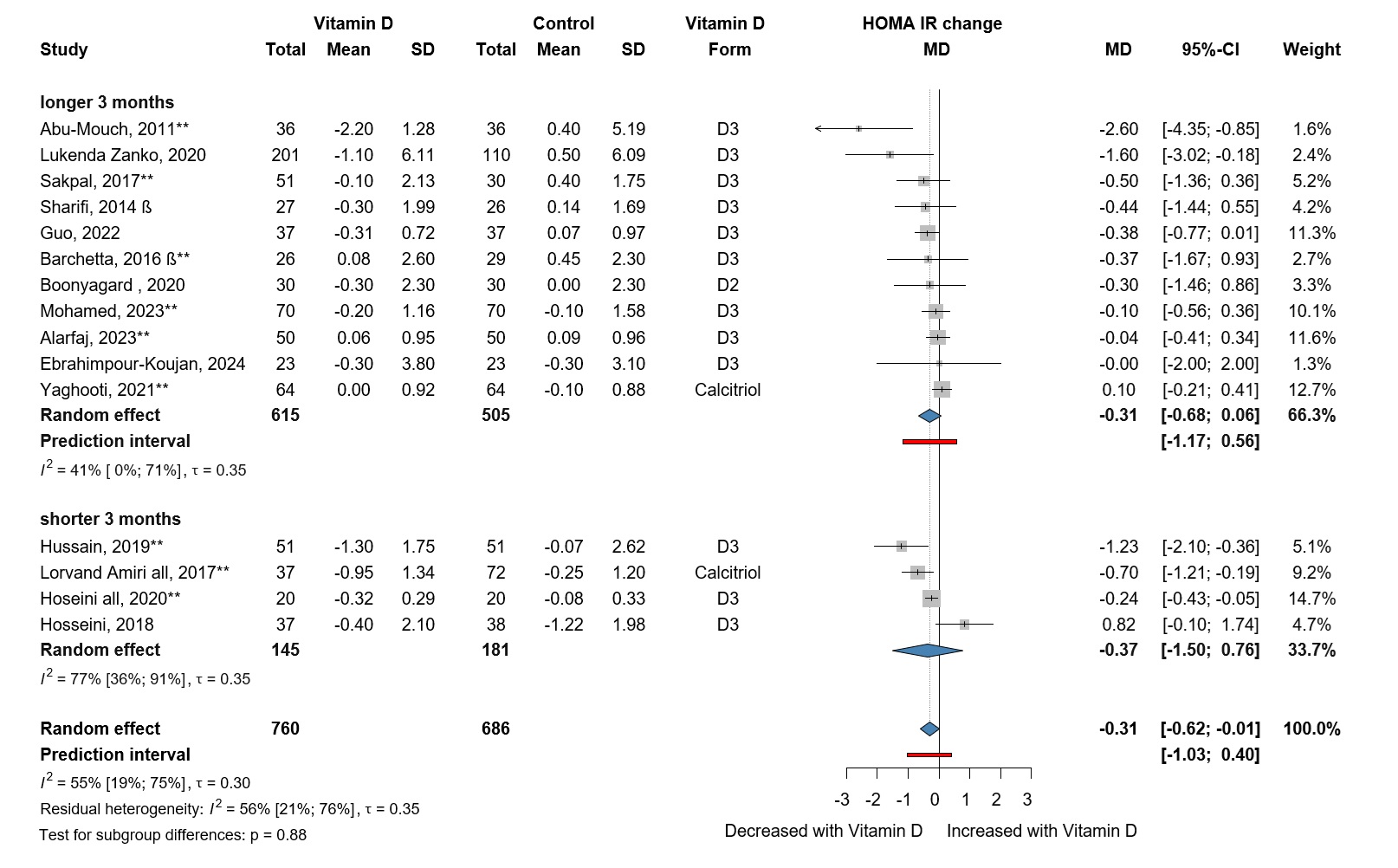


*Figure S3.2. Forest plot showing HOMA-IR change in vitamin D and control groups by length of intervention. CI: confidence interval; HOMA-IR: homeostatic model assessment for insulin resistance; MD: mean difference; SD: standard deviation. If the study is indicated with **, then the change value is an estimated change value in that study. The β means that the mean and SD are estimated mean and SD in that study. See raw data and synthesis methods.*


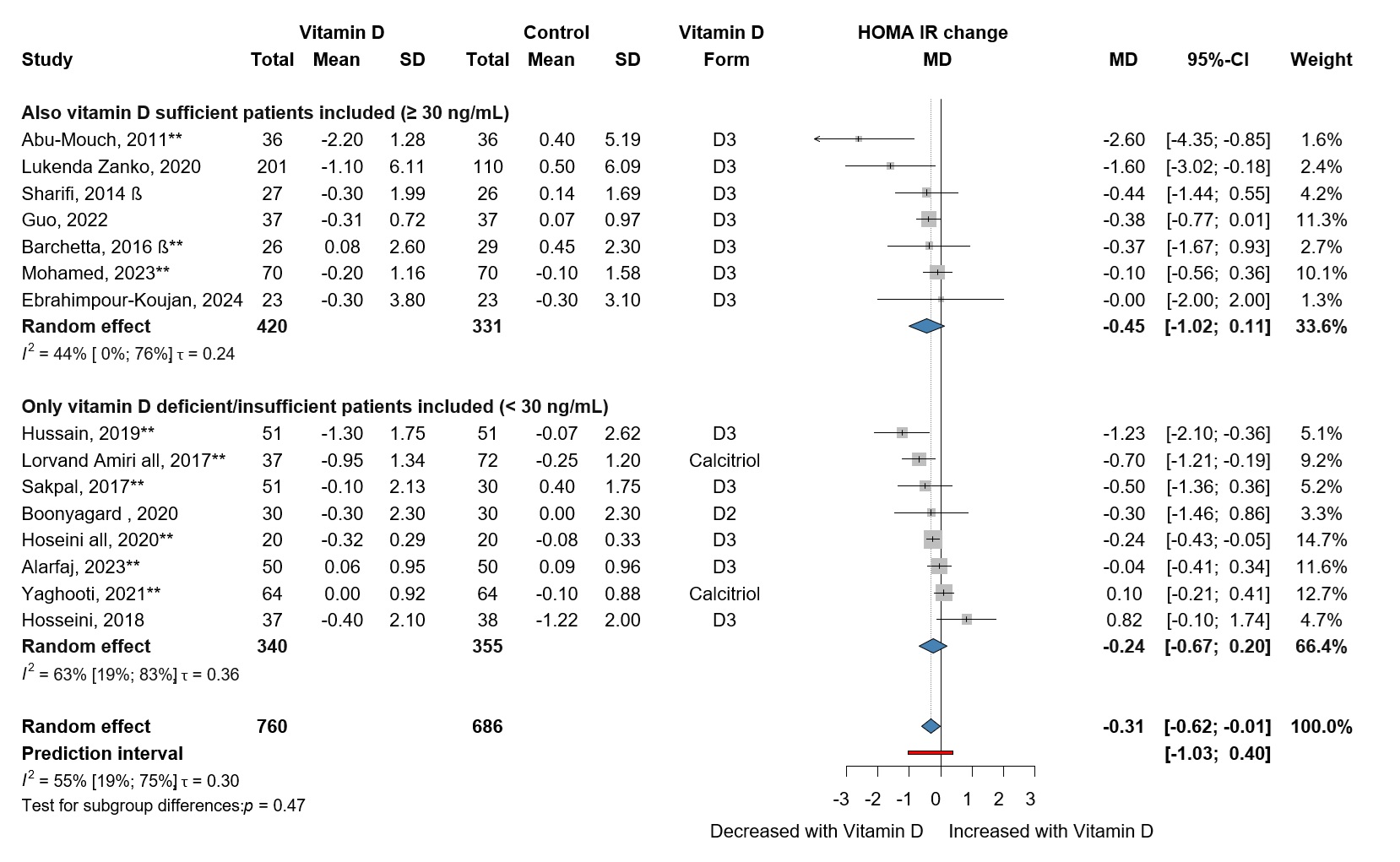


*Figure S3.3. Forest plot showing HOMA-IR change in vitamin D and control groups divided into vitamin D deficient/insufficient (< 30 ng/mL) and sufficient (≥ 30 ng/mL) studies. CI: confidence interval; HOMA-IR: homeostatic model assessment for insulin resistance; MD: mean difference; SD: standard deviation. If the study is indicated with **, then the change value is an estimated change value in that study. The β means that the mean and SD are estimated mean and SD in that study. See raw data and synthesis methods.*


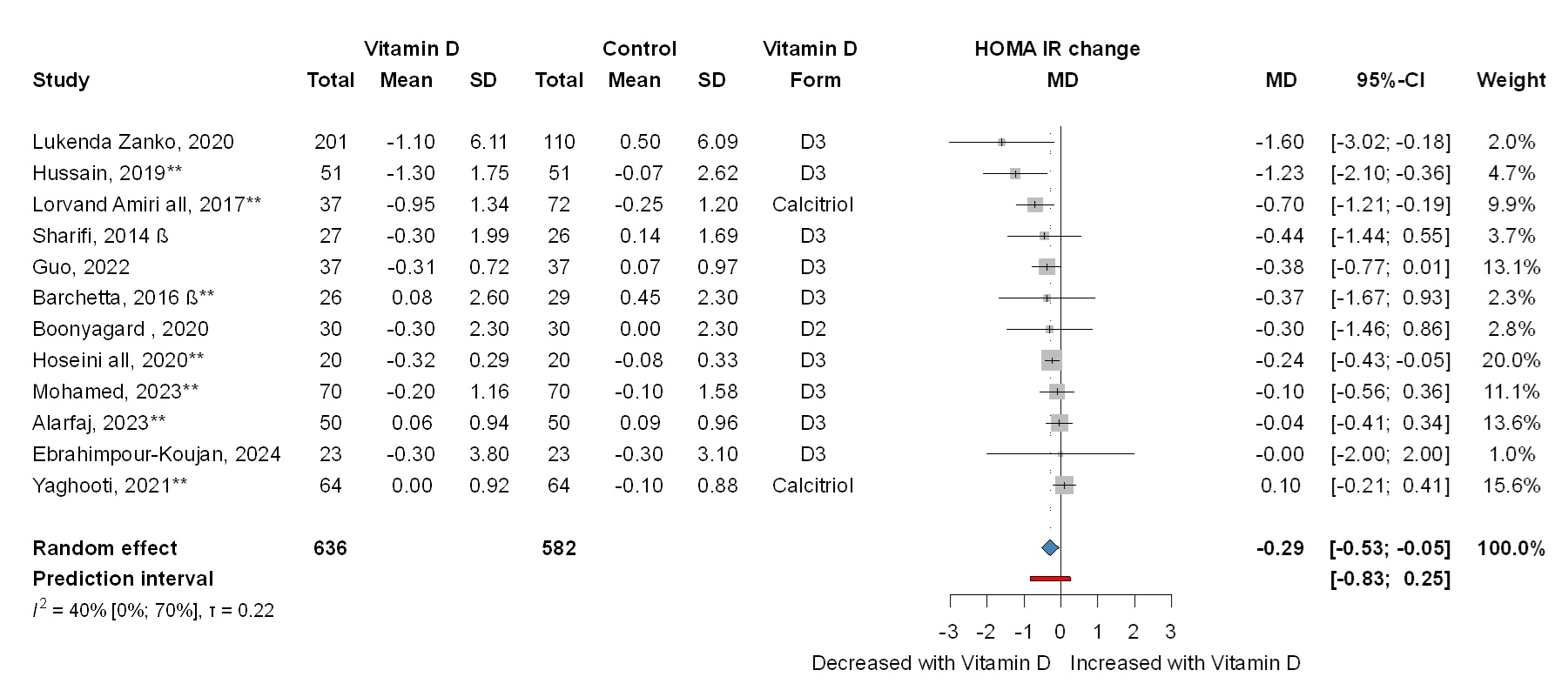


*Figure S3.4. Forest plot showing HOMA-IR change in vitamin D and control groups excluding high-risk biased studies. CI: confidence interval; HOMA-IR: homeostatic model assessment for insulin resistance; MD: mean difference; SD: standard deviation. If the study is indicated with **, then the change value is an estimated change value in that study. The β means that the mean and SD are estimated mean and SD in that study. See raw data and synthesis methods.*


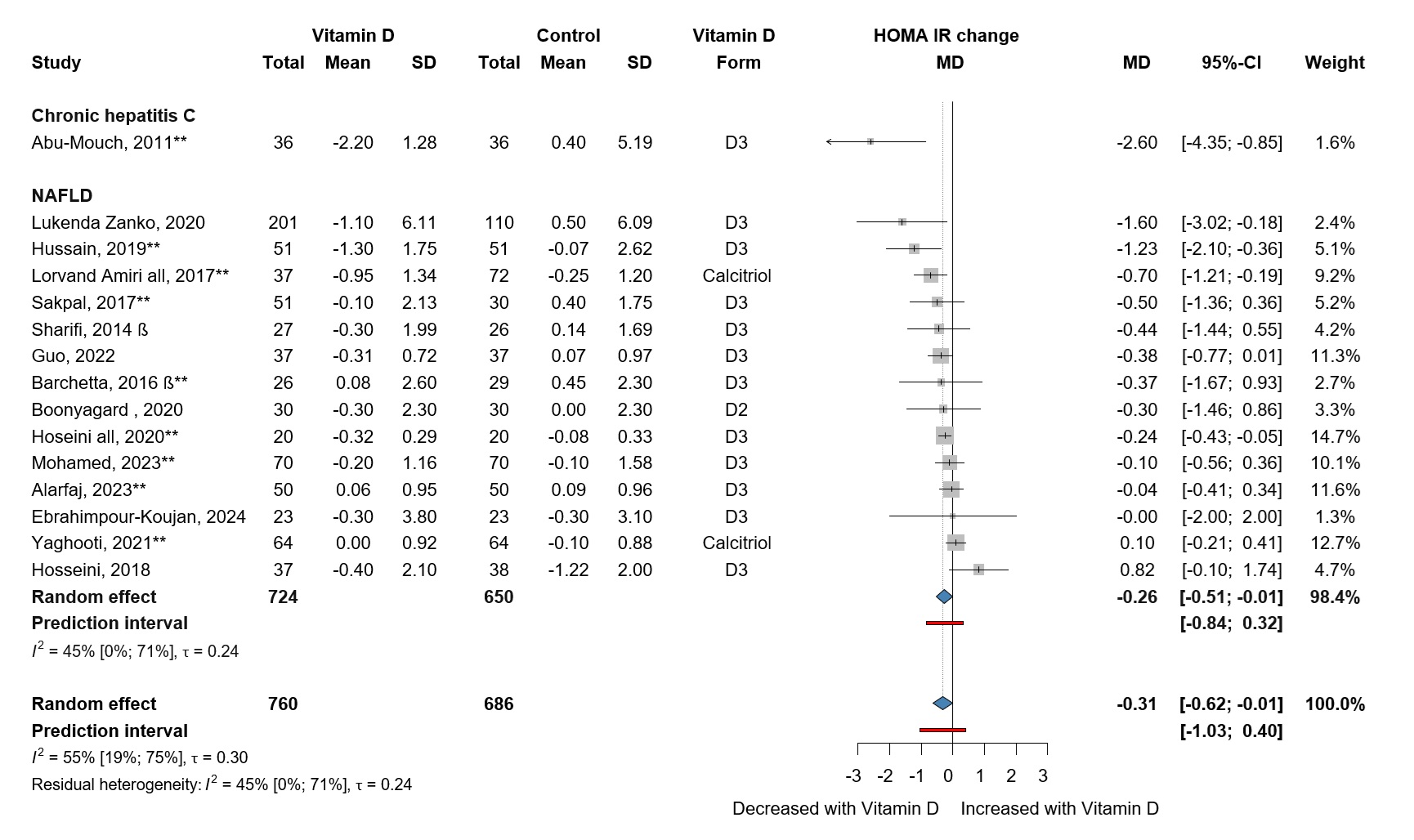


*Figure S3.5. Forest plot showing HOMA-IR change in vitamin D and control groups by type of chronic liver disease. CI: confidence interval; HOMA-IR: homeostatic model assessment for insulin resistance; MD: mean difference; NAFLD: Non-alcoholic fatty liver disease; SD: standard deviation. If the study is indicated with **, then the change value is an estimated change value in that study. The β means that the mean and SD are estimated mean and SD in that study. See raw data and synthesis methods.*

***Insulin***


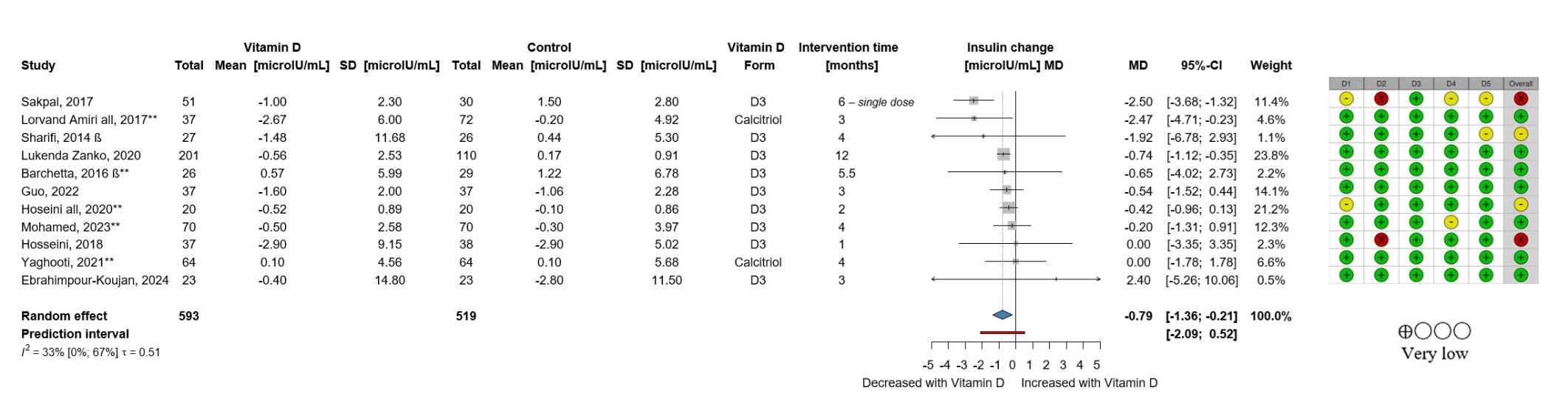


*Figure S3.6a. Forest plot showing insulin change in vitamin D and control groups excluding Alarfaj article (30).* *CI: confidence interval; MD: mean difference; SD: standard deviation. If the study is indicated with **, then the change value is an estimated change value in that study. The β means that the mean and SD are estimated mean and SD in that study. See raw data and synthesis methods.*


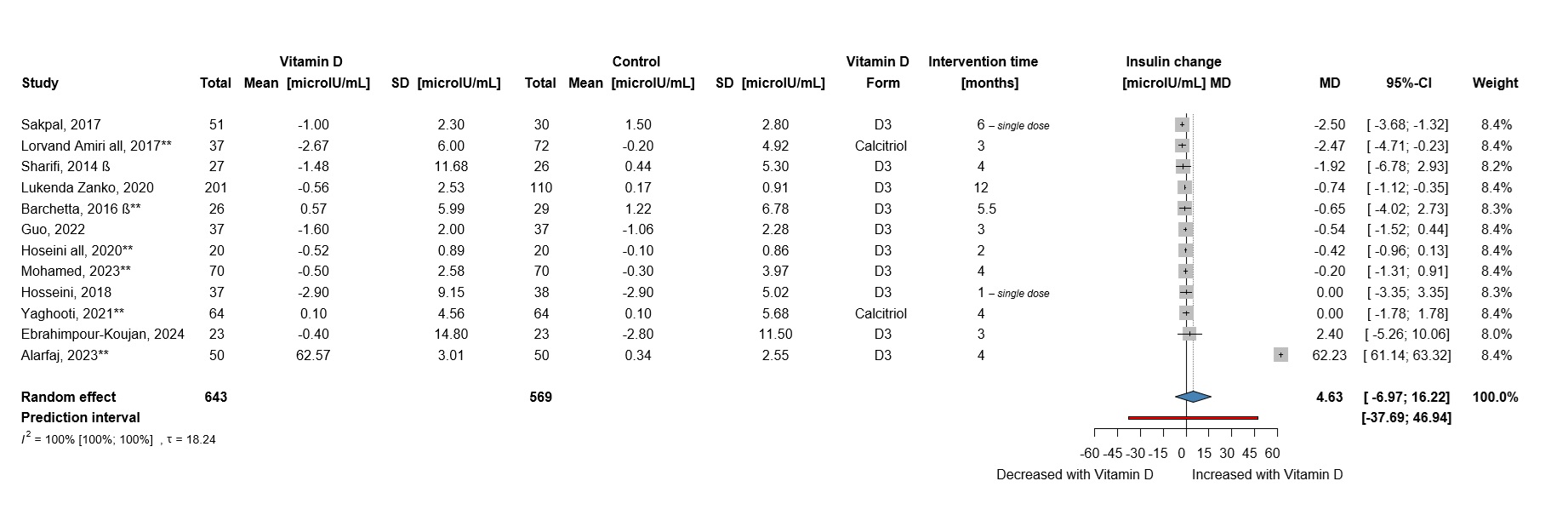


*Figure S3.6b. Forest plot showing insulin change in vitamin D and control groups including Alarfaj article (30). CI: confidence interval; MD: mean difference; SD: standard deviation. If the study is indicated with **, then the change value is an estimated change value in that study. The β means that the mean and SD are estimated mean and SD in that study. See raw data and synthesis methods.*

*Figure S3.6c. Funnel plot for insulin (p=* *0.7514).*

*Not run.*

*Figure S3.6d. Forest plot with leave-one-out analysis for insulin.*

*Figure S3.6e. Baujat plot for insulin.*


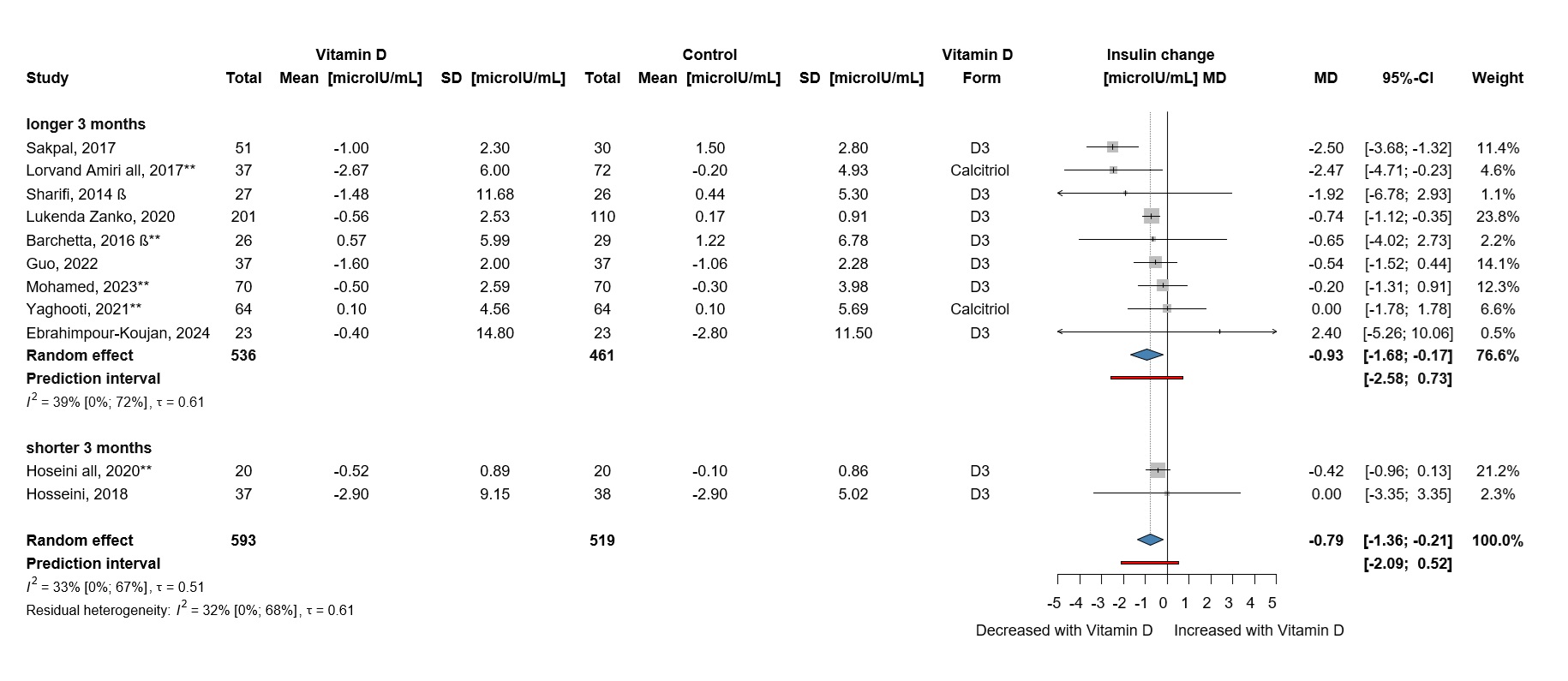


*Figure S3.7. Forest plot showing insulin change in vitamin D and control groups by length of intervention. CI: confidence interval; MD: mean difference; SD: standard deviation. If the study is indicated with **, then the change value is an estimated change value in that study. The β means that the mean and SD are estimated mean and SD in that study. See raw data and synthesis methods.*


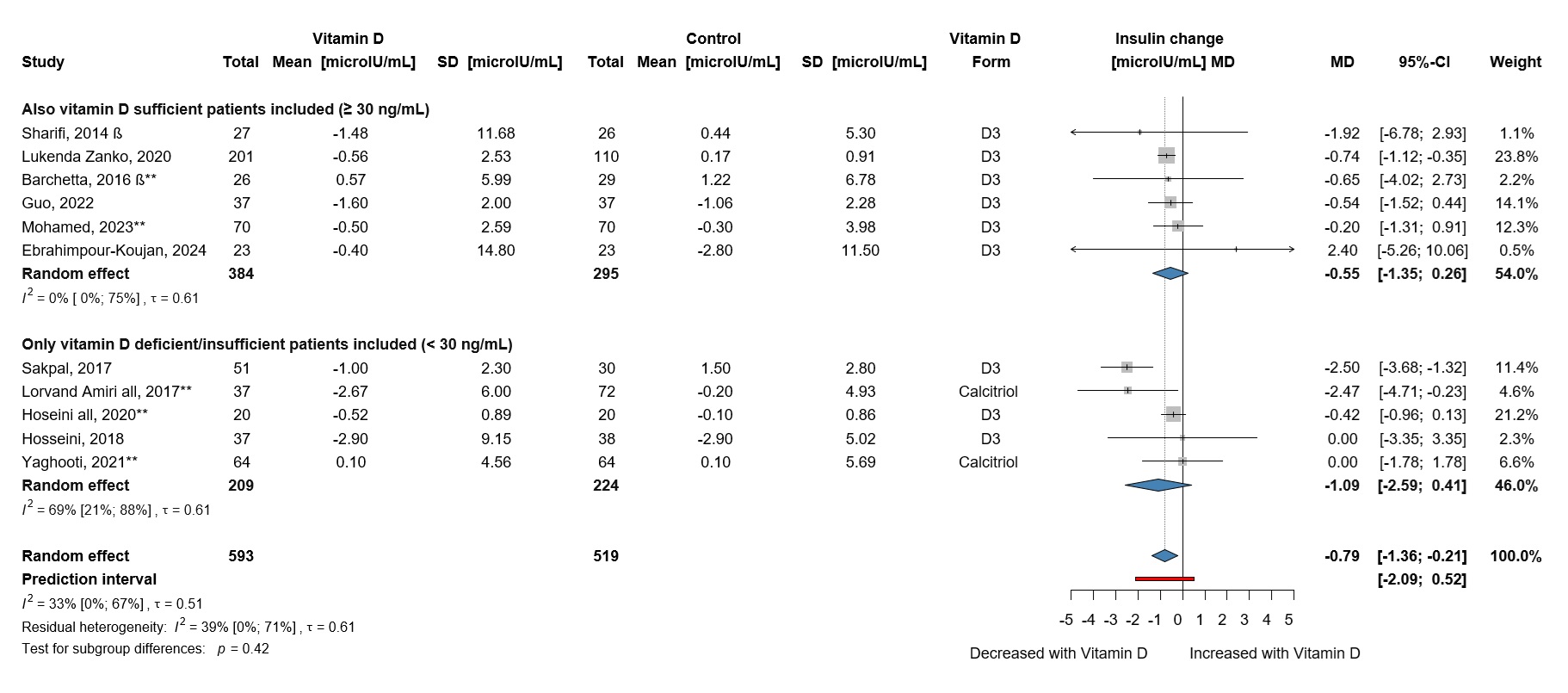


*Figure S3.8. Forest plot showing insulin change in vitamin D and control groups divided into vitamin D deficient/insufficient (< 30 ng/mL) and sufficient (≥ 30 ng/mL) studies. CI: confidence interval; MD: mean difference; SD: standard deviation. If the study is indicated with **, then the change value is an estimated change value in that study. The β means that the mean and SD are estimated mean and SD in that study. See raw data and synthesis methods.*


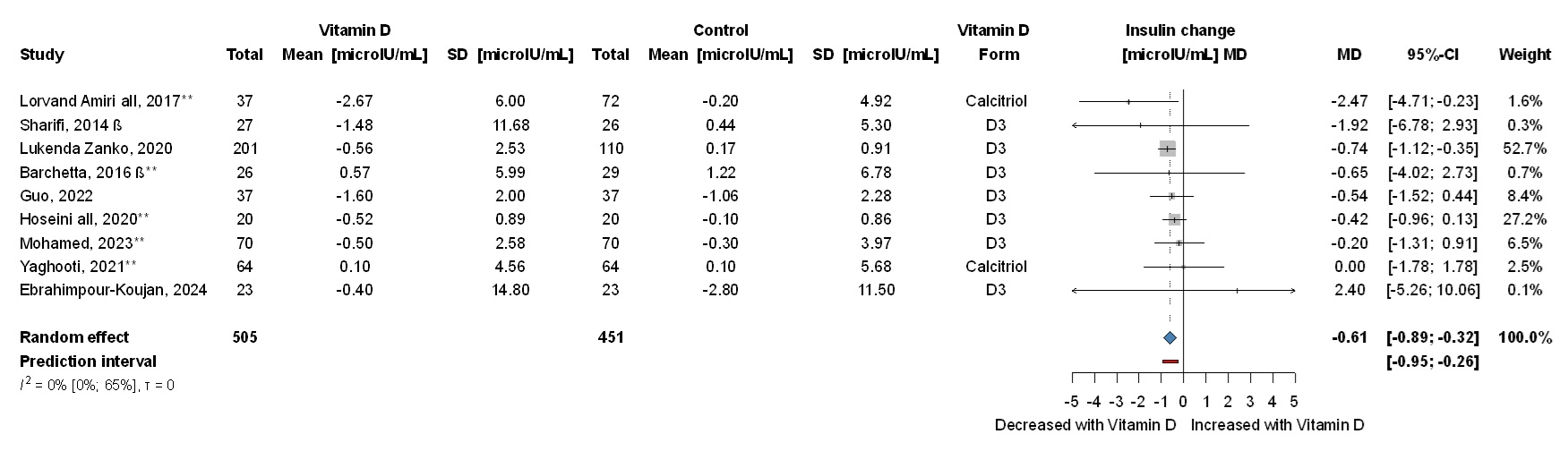


*Figure S3.9. Forest plot showing insulin change in vitamin D and control groups excluding high-risk biased studies. CI: confidence interval; MD: mean difference; SD: standard deviation. If the study is indicated with **, then the change value is an estimated change value in that study. The β means that the mean and SD are estimated mean and SD in that study. See raw data and synthesis methods.*


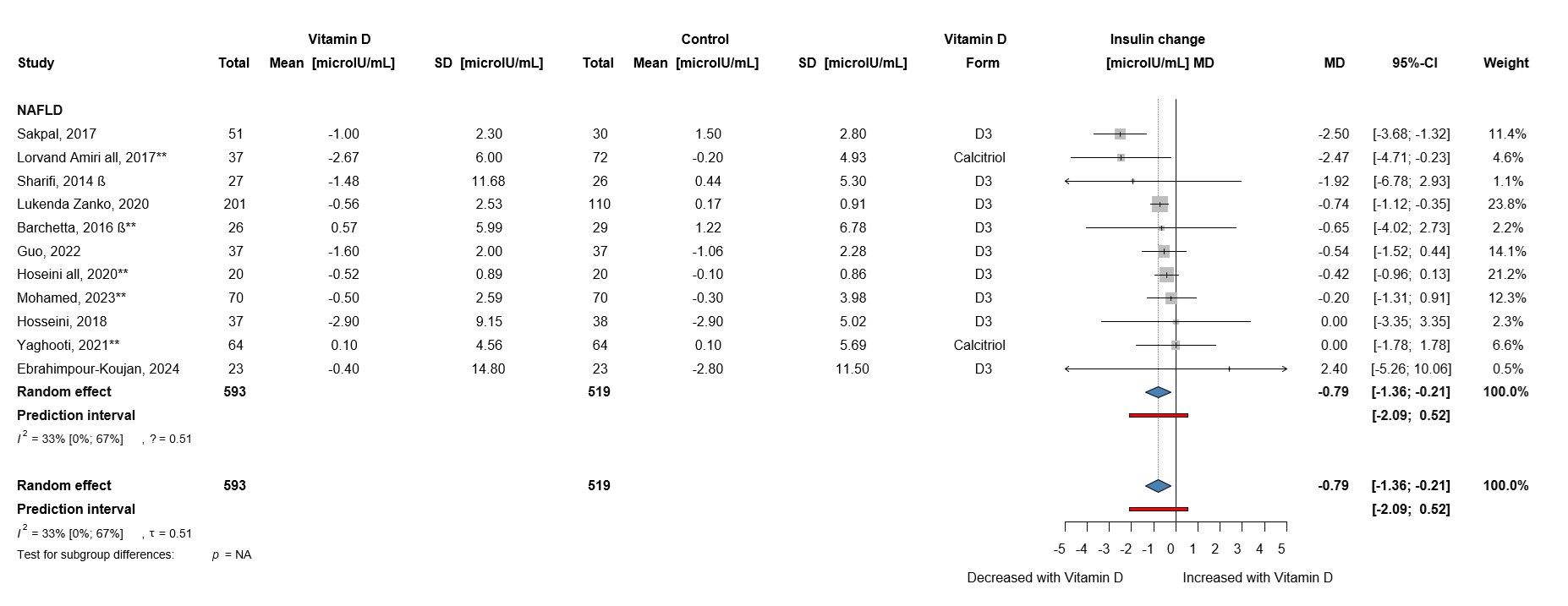


*Figure S3.10. Forest plot showing insulin change in vitamin D and control groups by type of chronic liver disease. CI: confidence interval; MD: mean difference; SD: standard deviation. If the study is indicated with **, then the change value is an estimated change value in that study. The β means that the mean and SD are estimated mean and SD in that study. See raw data and synthesis methods.*

***Fasting plasma glucose (FPG)***


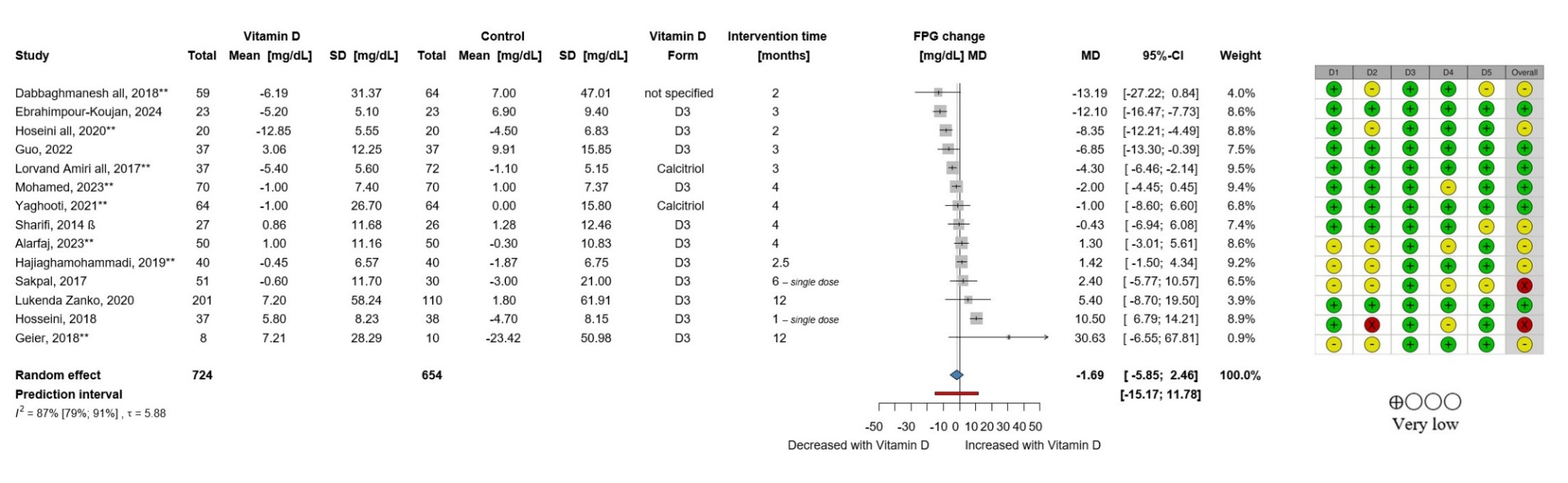


*Figure S3.11a. Forest plot showing fasting plasma glucose (FPG) change in vitamin D and control groups. CI: confidence interval; FPG: fasting plasma glucose; MD: mean difference; SD: standard deviation. If the study is indicated with **, then the change value is an estimated change value in that study. The β means that the mean and SD are estimated mean and SD in that study. See raw data and synthesis methods.*

*Figure S3.11b. Funnel plot for FPG (p=0.755).*

*Figure S3.11c. Forest plot with leave-one-out analysis for FPG.*

*Figure S3.11d. Baujat plot for FPG.*

*
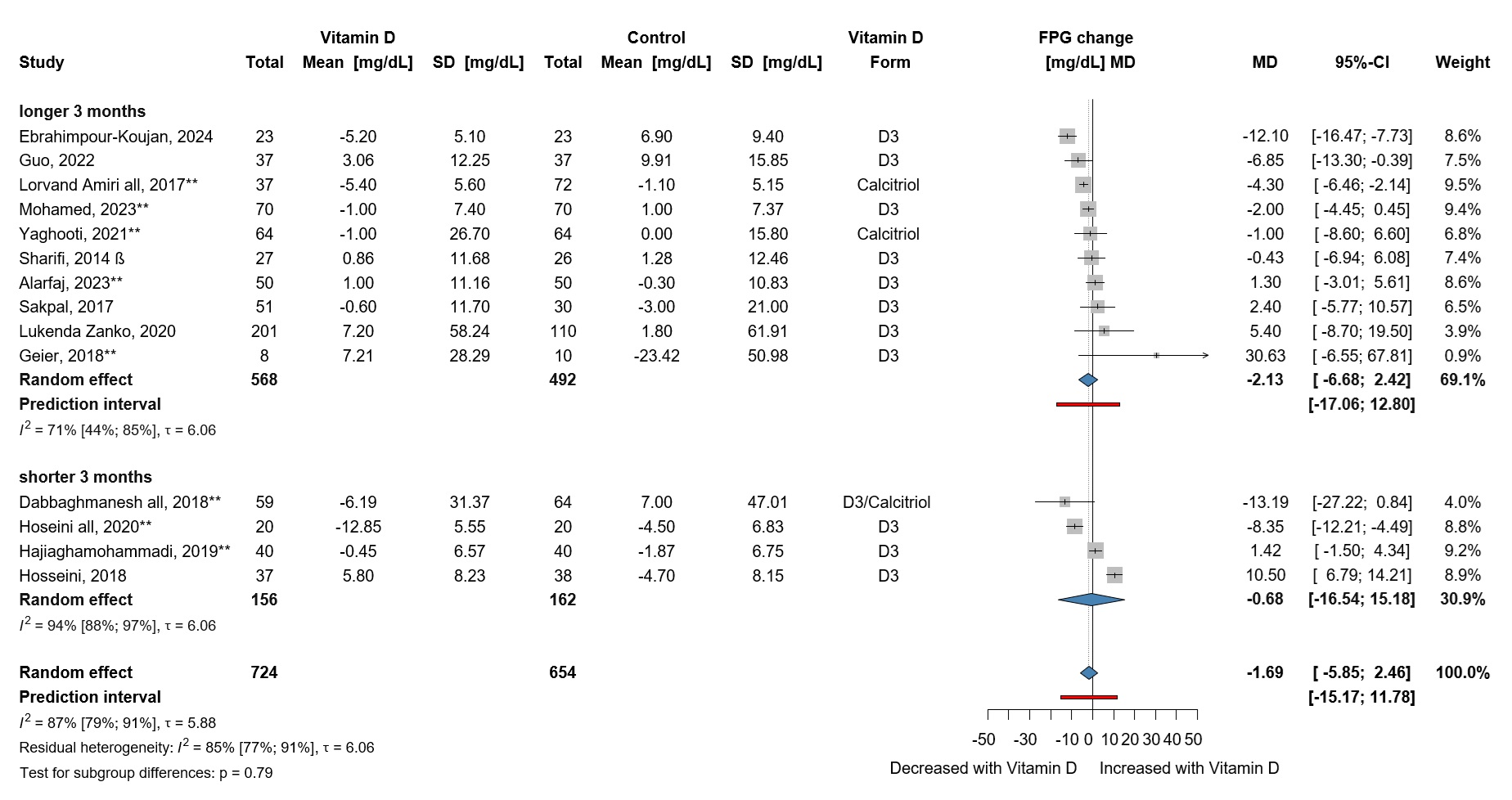
*

*Figure S3.12. Forest plot showing FPG change in vitamin D and control groups by length of intervention. CI: confidence interval; FPG: fasting plasma glucose; MD: mean difference; SD: standard deviation. If the study is indicated with **, then the change value is an estimated change value in that study. The β means that the mean and SD are estimated mean and SD in that study. See raw data and synthesis methods.*

*
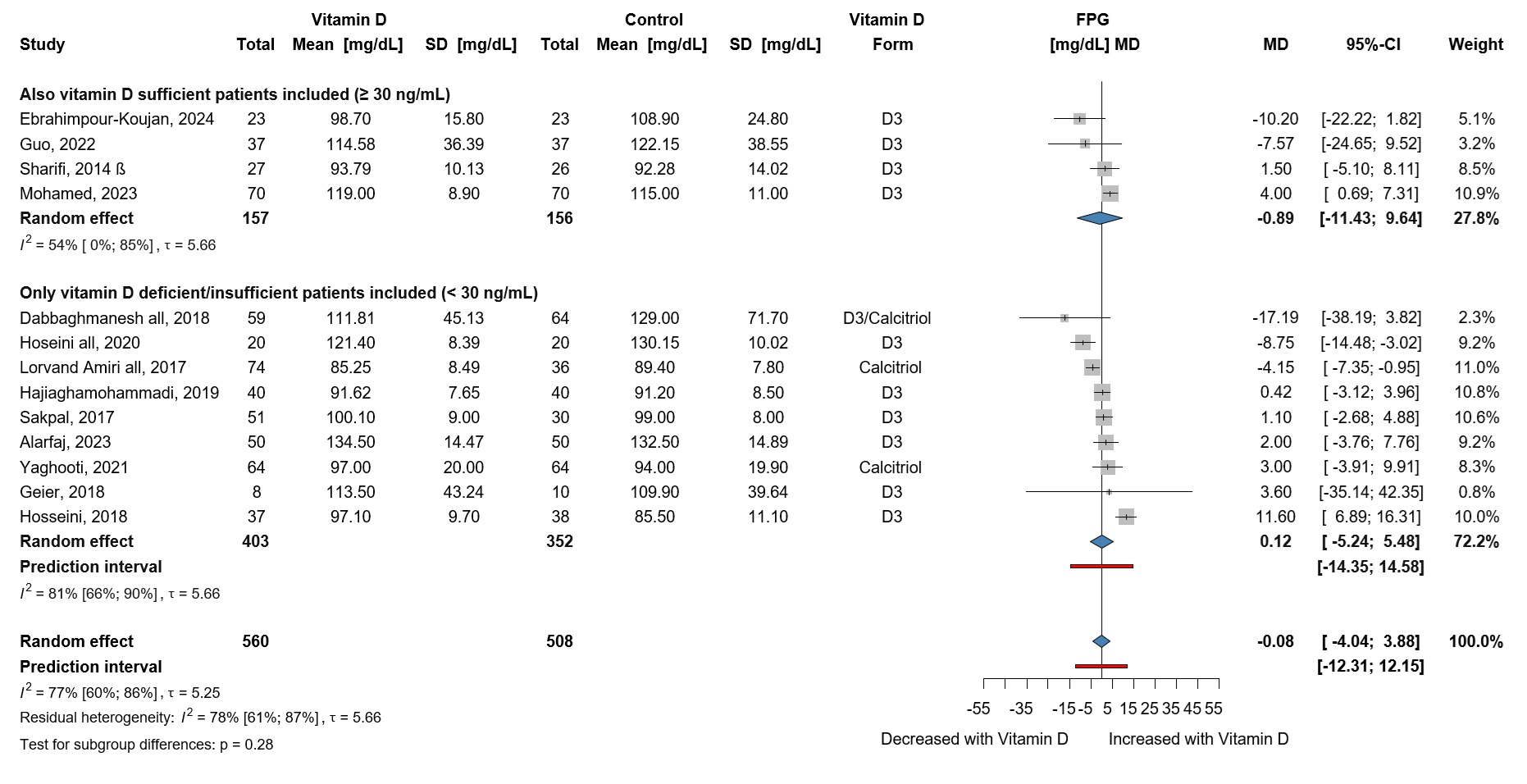
*

*Figure S3.13. Forest plot showing FPG change in vitamin D and control groups divided into vitamin D deficient/insufficient (< 30 ng/mL) and sufficient (≥ 30 ng/mL) studies. CI: confidence interval; FPG: fasting plasma glucose; MD: mean difference; SD: standard deviation. If the study is indicated with **, then the change value is an estimated change value in that study. The β means that the mean and SD are estimated mean and SD in that study. See raw data and synthesis methods.*

*
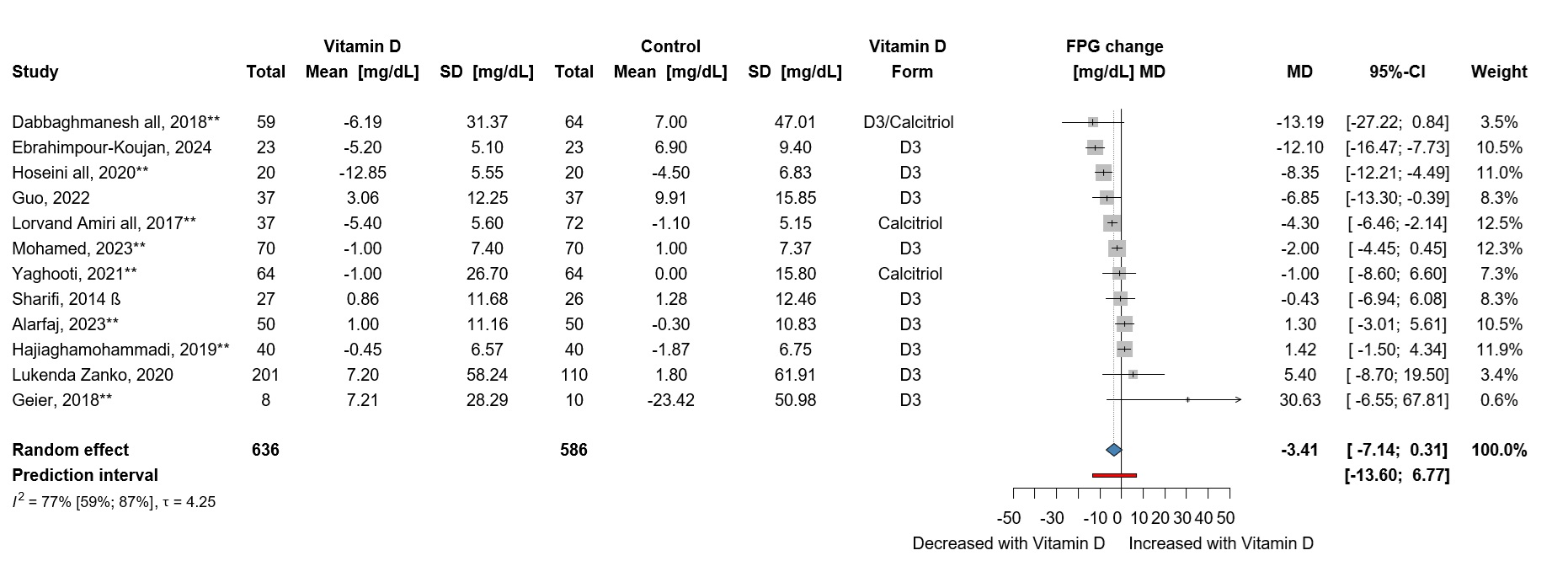
*

*Figure S3.14. Forest plot showing FPG change in vitamin D and control groups excluding high-risk biased studies. CI: confidence interval; FPG: fasting plasma glucose; MD: mean difference; SD: standard deviation. If the study is indicated with **, then the change value is an estimated change value in that study. The β means that the mean and SD are estimated mean and SD in that study. See raw data and synthesis methods.*


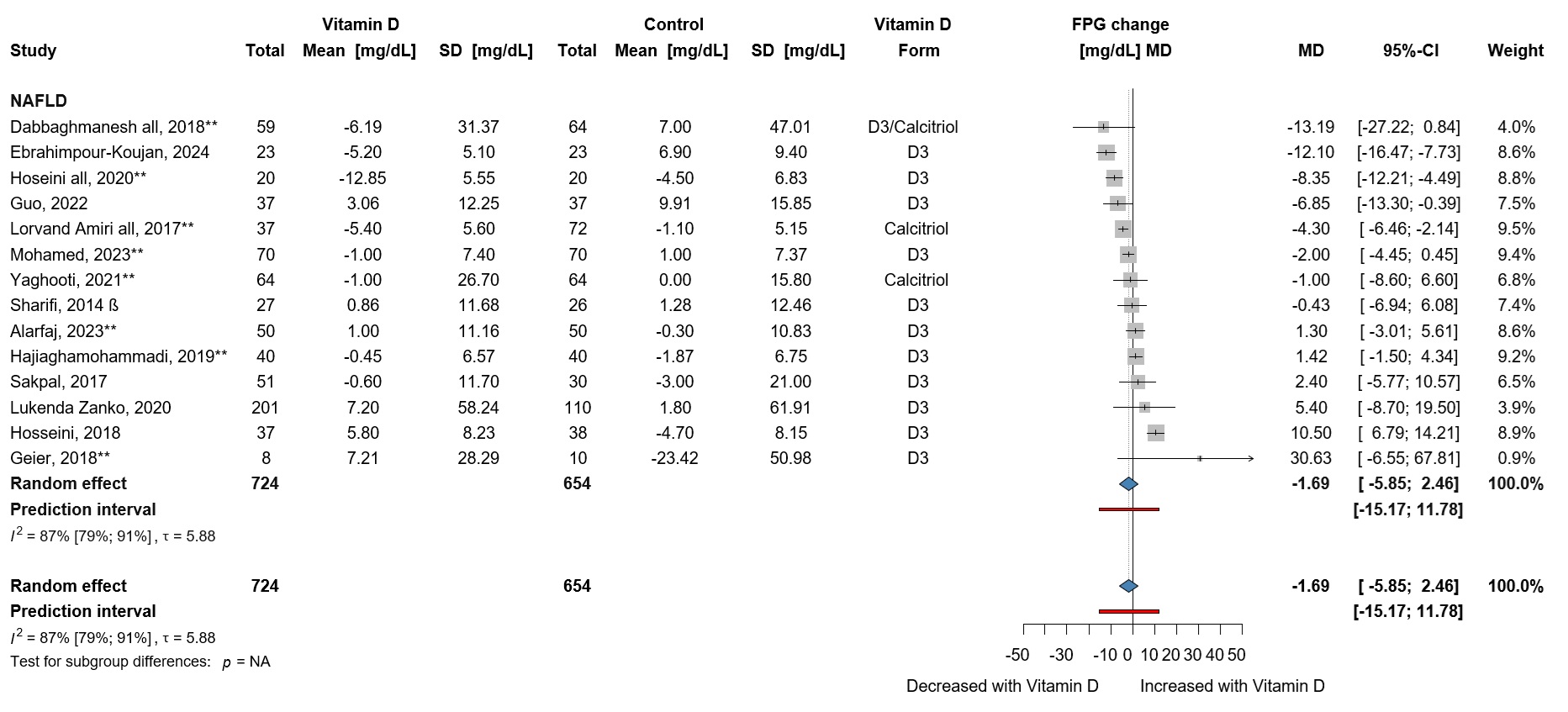


*Figure S3.15. Forest plot showing FPG change in vitamin D and control groups by type of chronic liver disease. CI: confidence interval; FPG: fasting plasma glucose; MD: mean difference; SD: standard deviation. If the study is indicated with **, then the change value is an estimated change value in that study. The β means that the mean and SD are estimated mean and SD in that study. See raw data and synthesis methods.*

**Supplementary File S4.** Lipid metabolism.

***Total cholesterol (TC)***


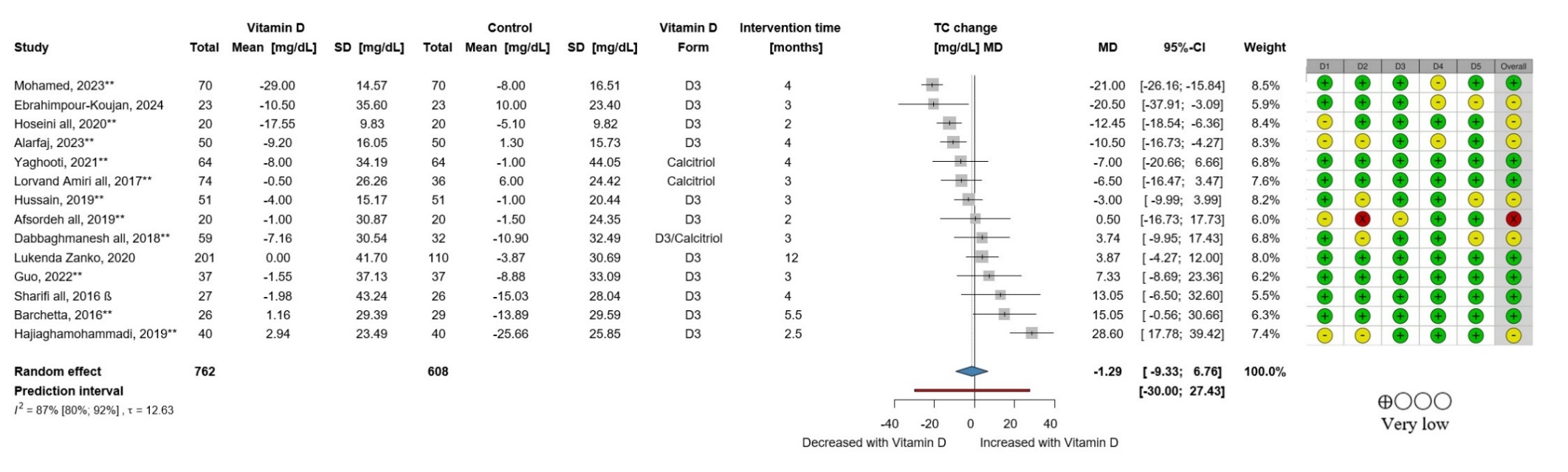


*Figure S4.1a. Forest plot showing total cholesterol change in vitamin D and control groups. CI: confidence interval; MD: mean difference; TC: total cholesterol; SD: standard deviation. If the study is indicated with **, then the change value is an estimated change value in that study. The β means that the mean and SD are estimated mean and SD in that study. See raw data and synthesis methods.*

*Figure S4.1b. Funnel plot for total cholesterol (p = 0.0291).*

In the case of TC, the visual inspection did not confirm potential publication bias but rather indicated high heterogeneity, both for high and low-sample-size studies.

*Figure S4.1c. Forest plot with leave-one-out analysis for total cholesterol.*

*Figure S4.1d. Baujat plot for total cholesterol.*


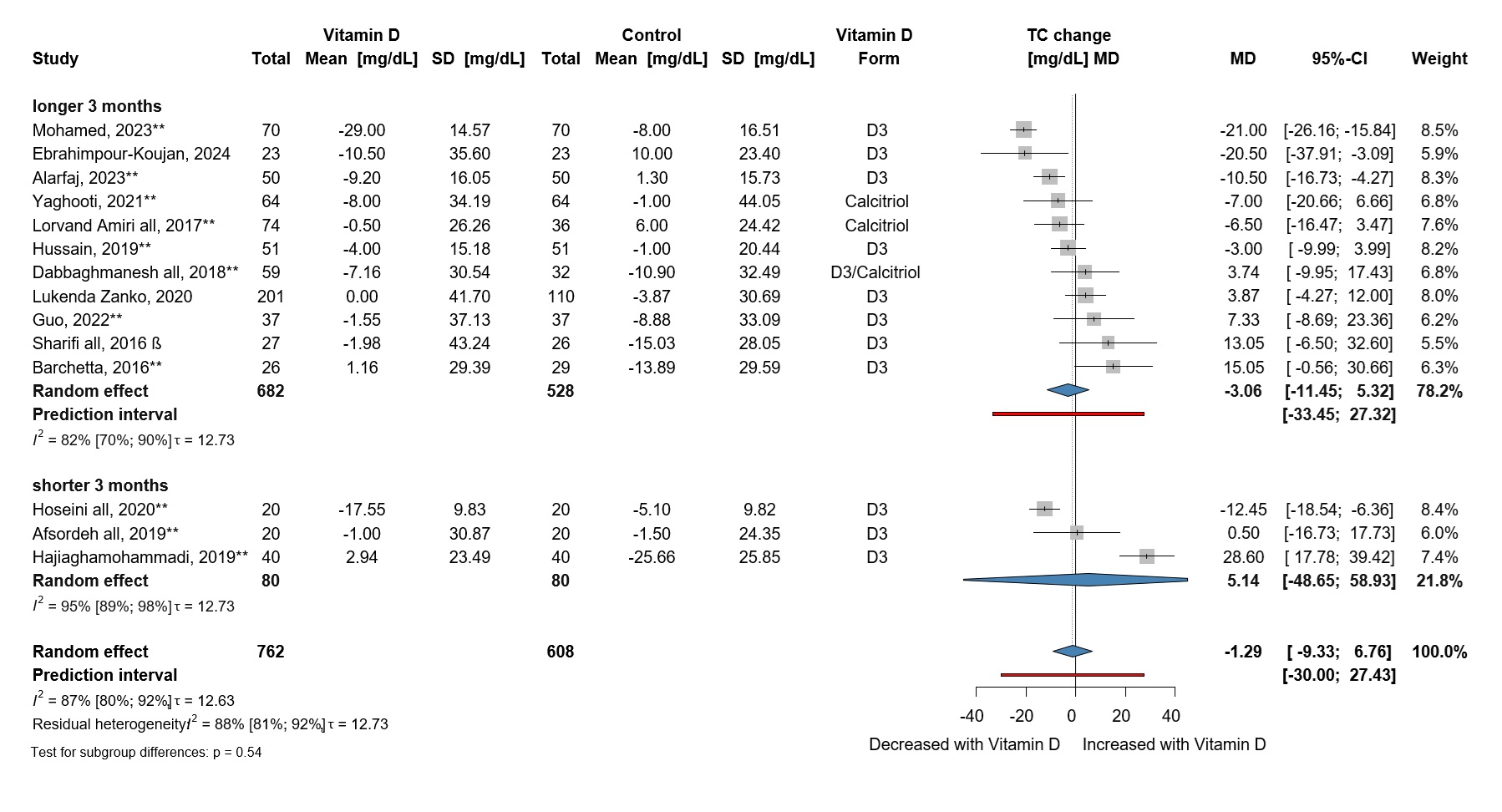


*Figure S4.2. Forest plot showing total cholesterol change in vitamin D and control groups by length of intervention. CI: confidence interval; MD: mean difference; TC: total cholesterol; SD: standard deviation. If the study is indicated with **, then the change value is an estimated change value in that study. The β means that the mean and SD are estimated mean and SD in that study. See raw data and synthesis methods.*


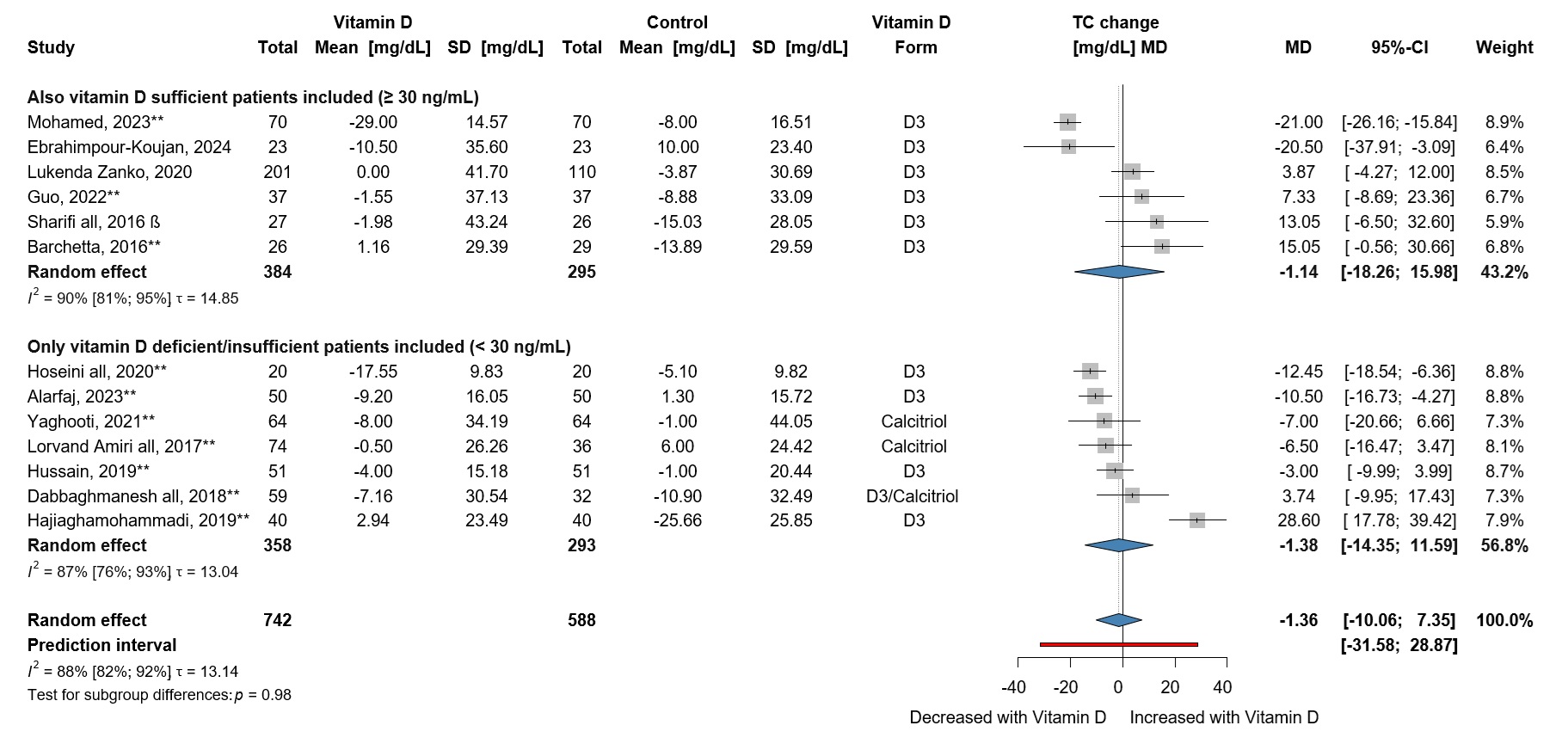


*Figure S4.3. Forest plot showing total cholesterol change in vitamin D and control groups divided into vitamin D deficient/insufficient (< 30 ng/mL) and sufficient (≥ 30 ng/mL) studies. CI: confidence interval; MD: mean difference; TC: total cholesterol; SD: standard deviation. If the study is indicated with **, then the change value is an estimated change value in that study. The β means that the mean and SD are estimated mean and SD in that study. See raw data and synthesis methods.*


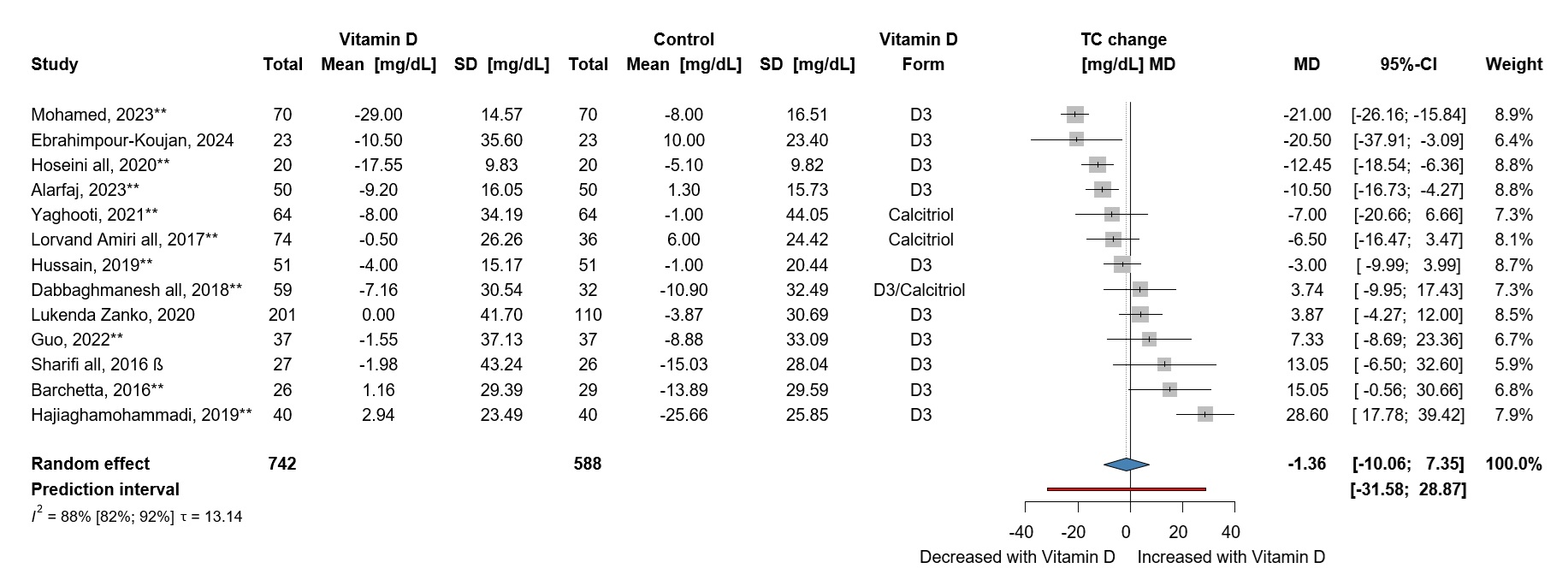


*Figure S4.4. Forest plot showing total cholesterol change in vitamin D and control groups excluding high-risk biased studies. CI: confidence interval; MD: mean difference; TC: total cholesterol; SD: standard deviation. If the study is indicated with **, then the change value is an estimated change value in that study. The β means that the mean and SD are estimated mean and SD in that study. See raw data and synthesis methods.*


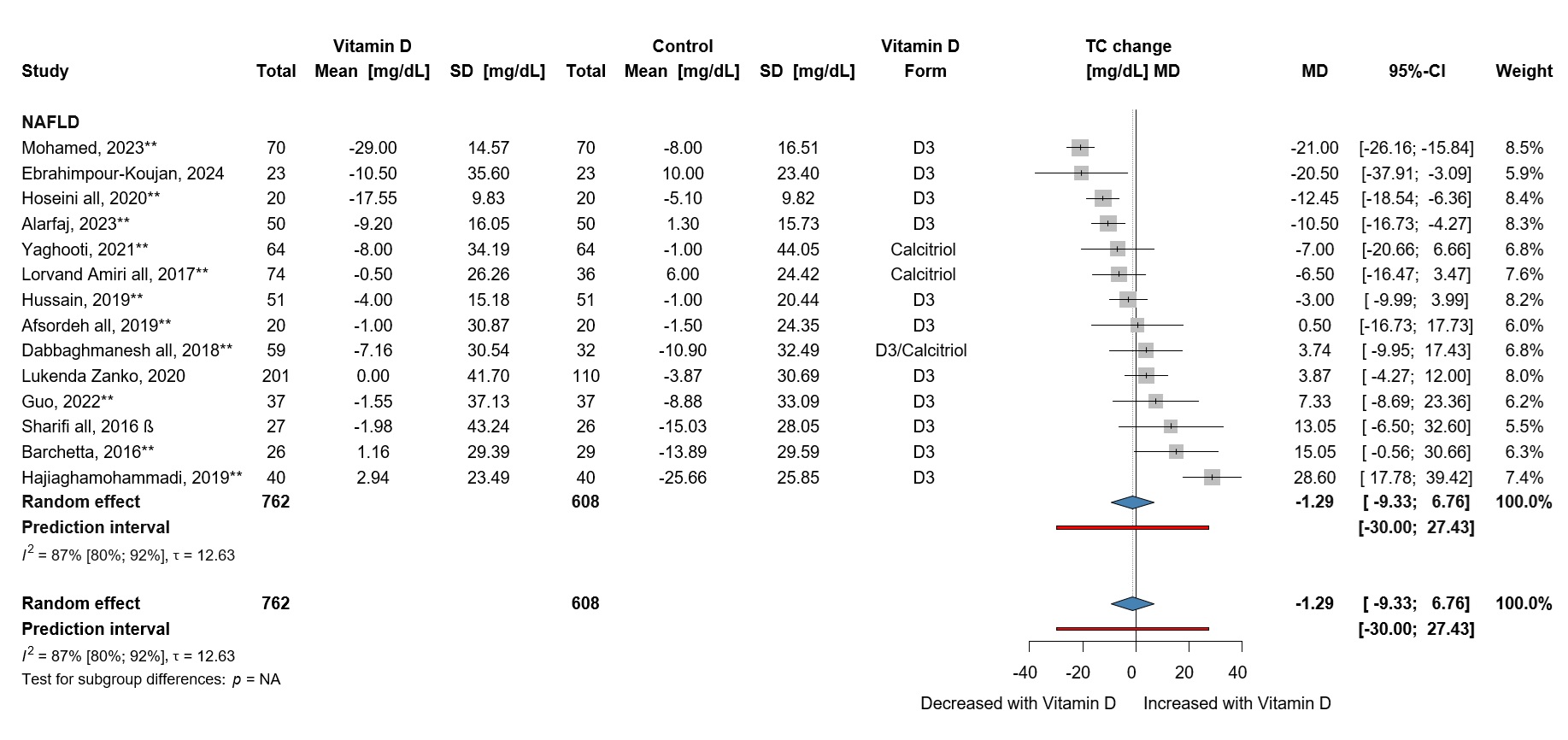


*Figure S4.5. Forest plot showing total cholesterol change in vitamin D and control groups by type of chronic liver disease. CI: confidence interval; MD: mean difference; TC: total cholesterol; SD: standard deviation. If the study is indicated with **, then the change value is an estimated change value in that study. The β means that the mean and SD are estimated mean and SD in that study. See raw data and synthesis methods.*

***Low density cholesterol (LDL)***


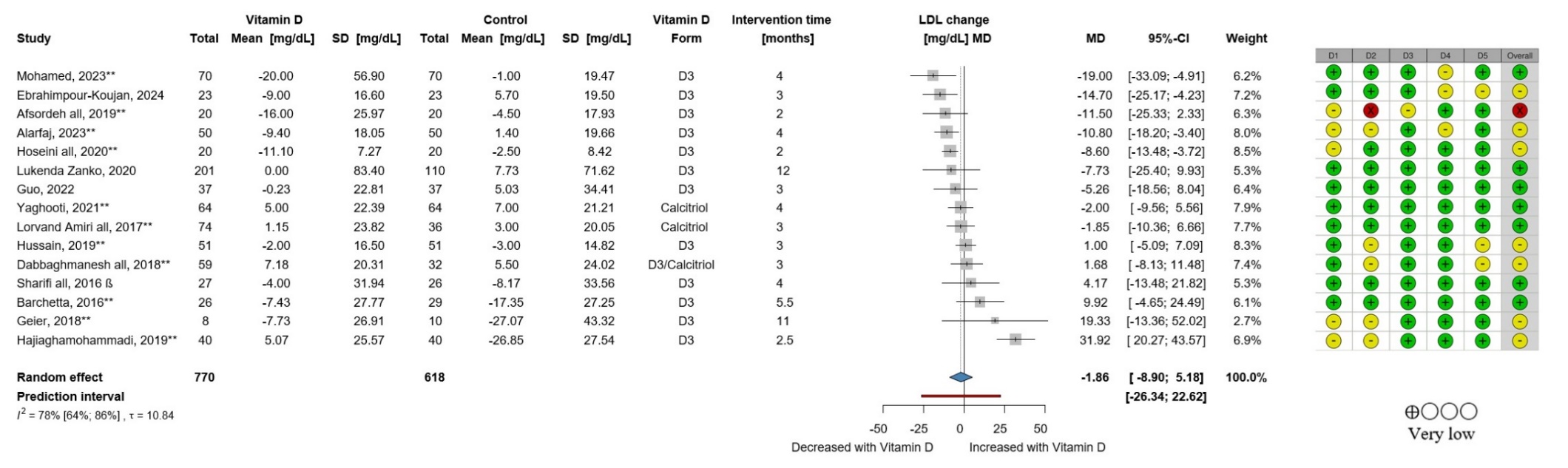


*Figure S4.6a. Forest plot showing low-density cholesterol (LDL) change in vitamin D and control groups. CI: confidence interval; LDL: low density cholesterol; MD: mean difference; SD: standard deviation. If the study is indicated with **, then the change value is an estimated change value in that study. The β means that the mean and SD are estimated mean and SD in that study. See raw data and synthesis methods.*

*Figure S4.6b. Funnel plot for LDL (p = 0.3799).*

*Figure S4.6c. Forest plot with leave-one-out analysis for LDL.*

*Figure S4.6d. Baujat plot for LDL.*


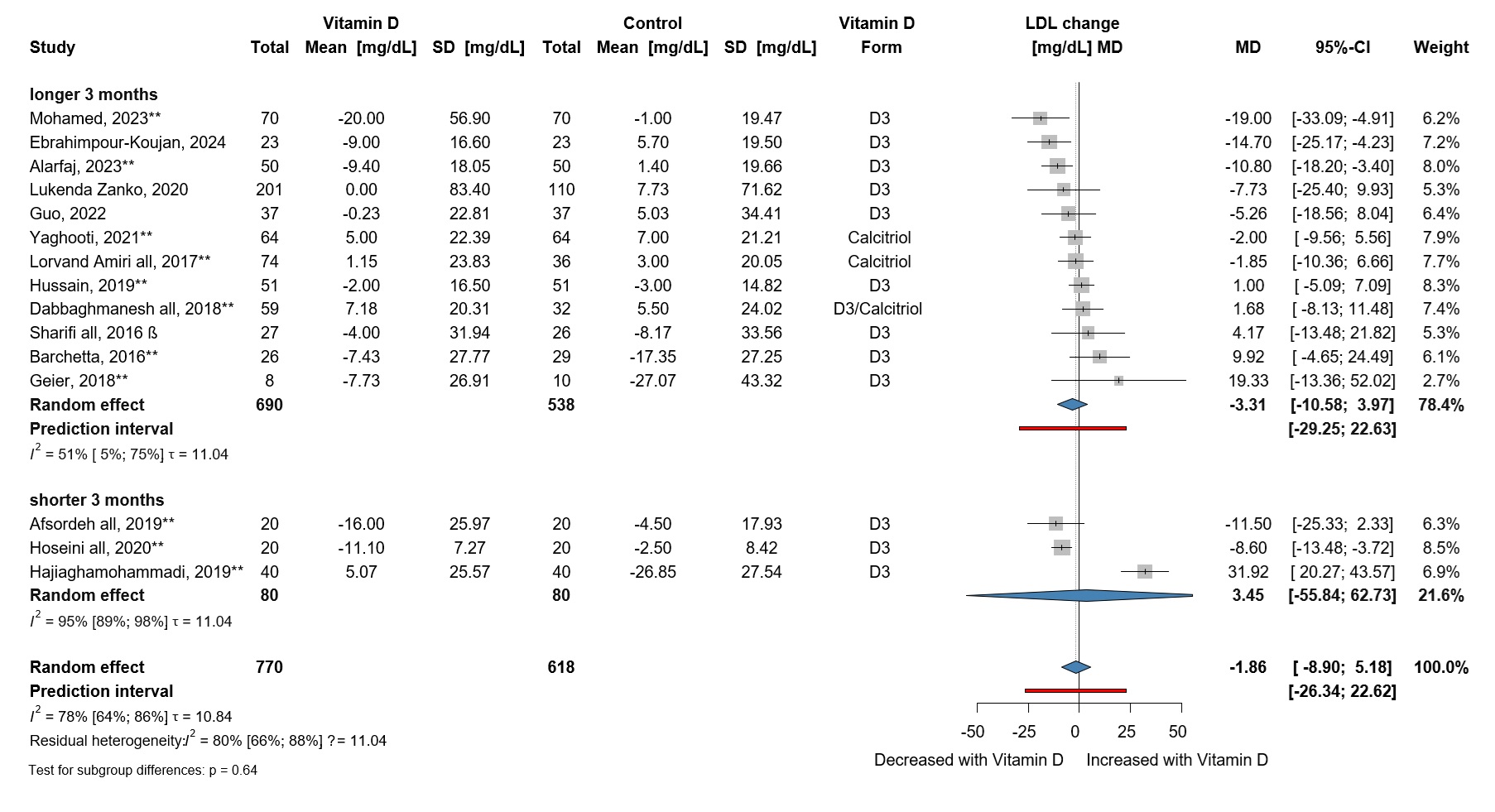


*Figure S4.7. Forest plot showing LDL change in vitamin D and control groups by length of intervention. CI: confidence interval; LDL: low density cholesterol; MD: mean difference; SD: standard deviation. If the study is indicated with **, then the change value is an estimated change value in that study. The β means that the mean and SD are estimated mean and SD in that study. See raw data and synthesis methods.*


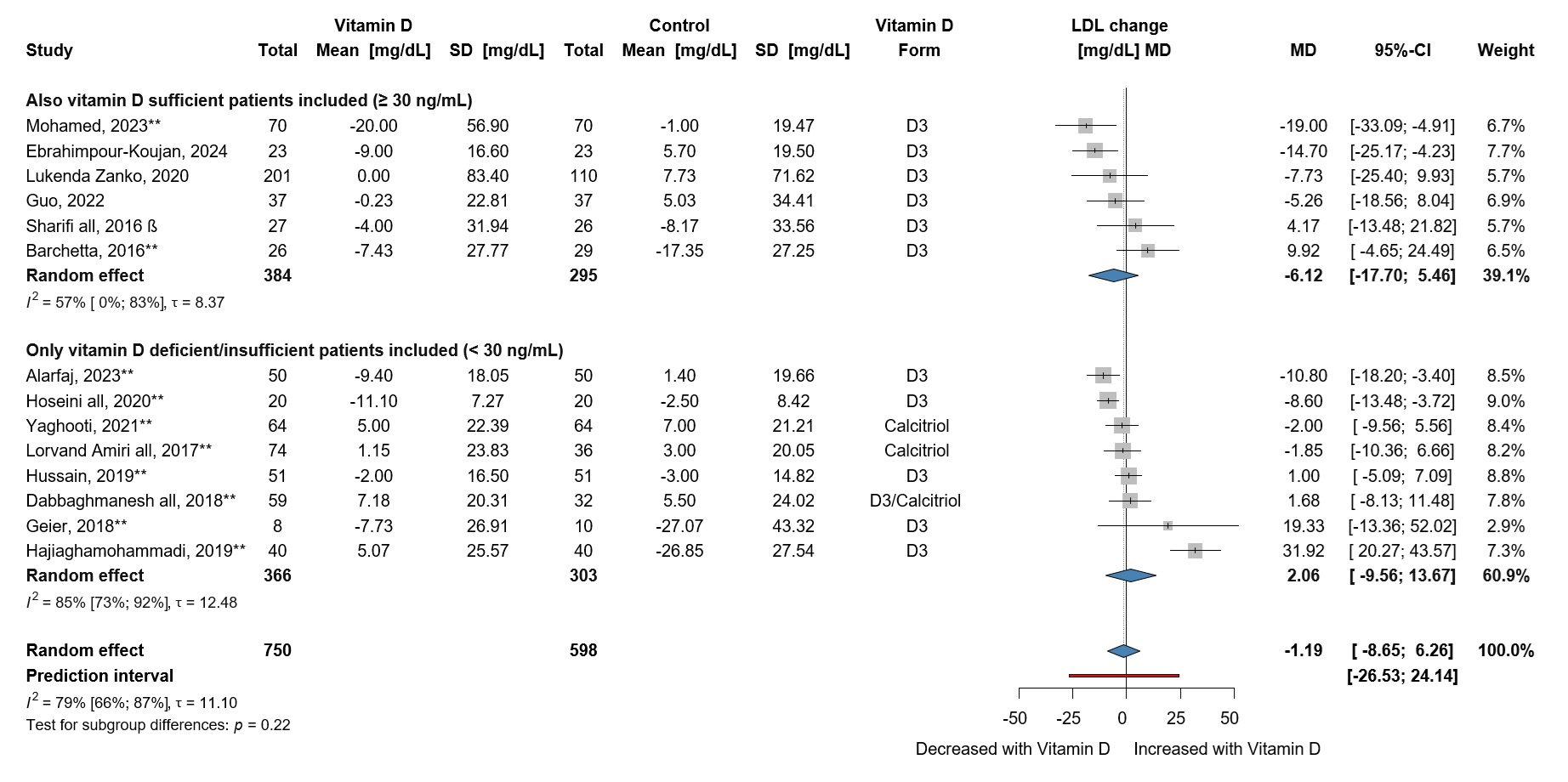


*Figure S4.8. Forest plots showing LDL change in vitamin D and control groups divided into vitamin D deficient/insufficient (< 30 ng/mL) and sufficient (≥ 30 ng/mL) studies. CI: confidence interval; LDL: low density cholesterol; MD: mean difference; SD: standard deviation. If the study is indicated with **, then the change value is an estimated change value in that study. The β means that the mean and SD are estimated mean and SD in that study. See raw data and synthesis methods.*


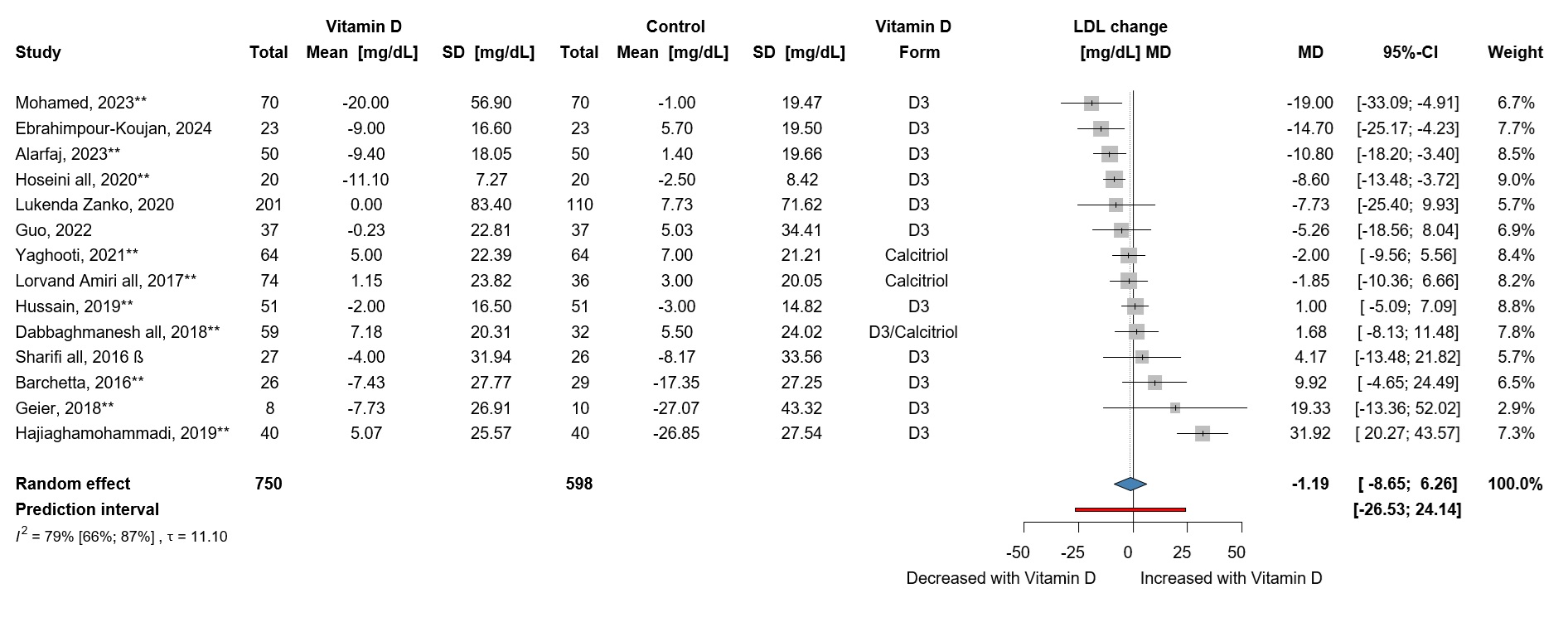


*Figure S4.9. Forest plot showing LDL change in vitamin D and control groups excluding high-risk biased studies. CI: confidence interval; LDL: low density cholesterol; MD: mean difference; SD: standard deviation. If the study is indicated with **, then the change value is an estimated change value in that study. The β means that the mean and SD are estimated mean and SD in that study. See raw data and synthesis methods.*


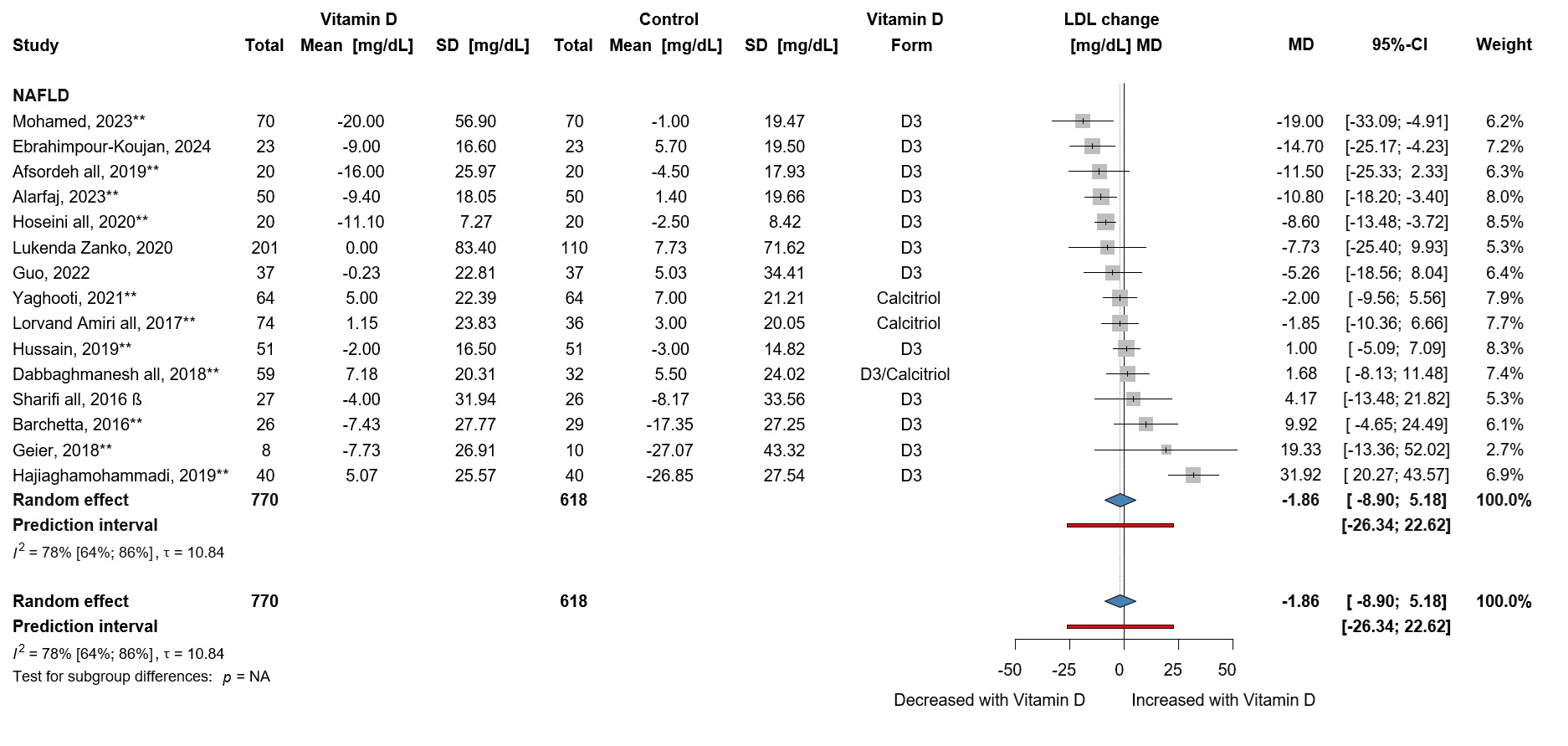


*Figure S4.10. Forest plot showing LDL change in vitamin D and control groups by type of chronic liver disease. CI: confidence interval; LDL: low density cholesterol; MD: mean difference; SD: standard deviation. If the study is indicated with **, then the change value is an estimated change value in that study. The β means that the mean and SD are estimated mean and SD in that study. See raw data and synthesis methods.*

***High density cholesterol (HDL)***


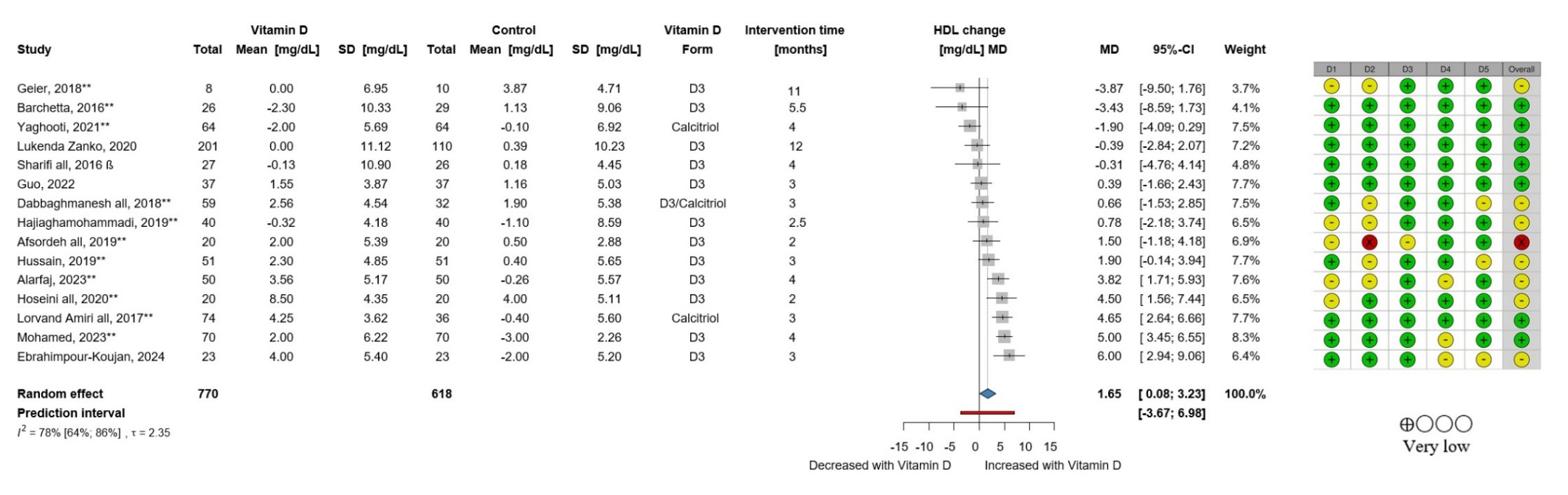


*Figure S4.11a. Forest plot showing high-density cholesterol (HDL) change in vitamin D and control groups. CI: confidence interval; HDL: high density cholesterol; MD: mean difference; SD: standard deviation. If the study is indicated with **, then the change value is an estimated change value in that study. The β means that the mean and SD are estimated mean and SD in that study. See raw data and synthesis methods.*

*Figure S4.11b. Funnel plot for HDL (p = 0.0963).*

In the case of HDL, visual inspection did not confirm potential publication bias but rather indicated high heterogeneity. However, if we assumed the presence of small study bias (as suggested by Geier et al. 2019 (40) and Barchetta et al. 2016 (33)), this would bias the combined effect toward a smaller MD, meaning the true effect might be somewhat higher than the observed effect. In summary, we believe that publication bias is unlikely, but even if present, it is not relevant based on the data.

*Figure S4.11c. Forest plot with leave-one-out analysis for HDL.*

*Figure S4.11d. Baujat plot for HDL.*


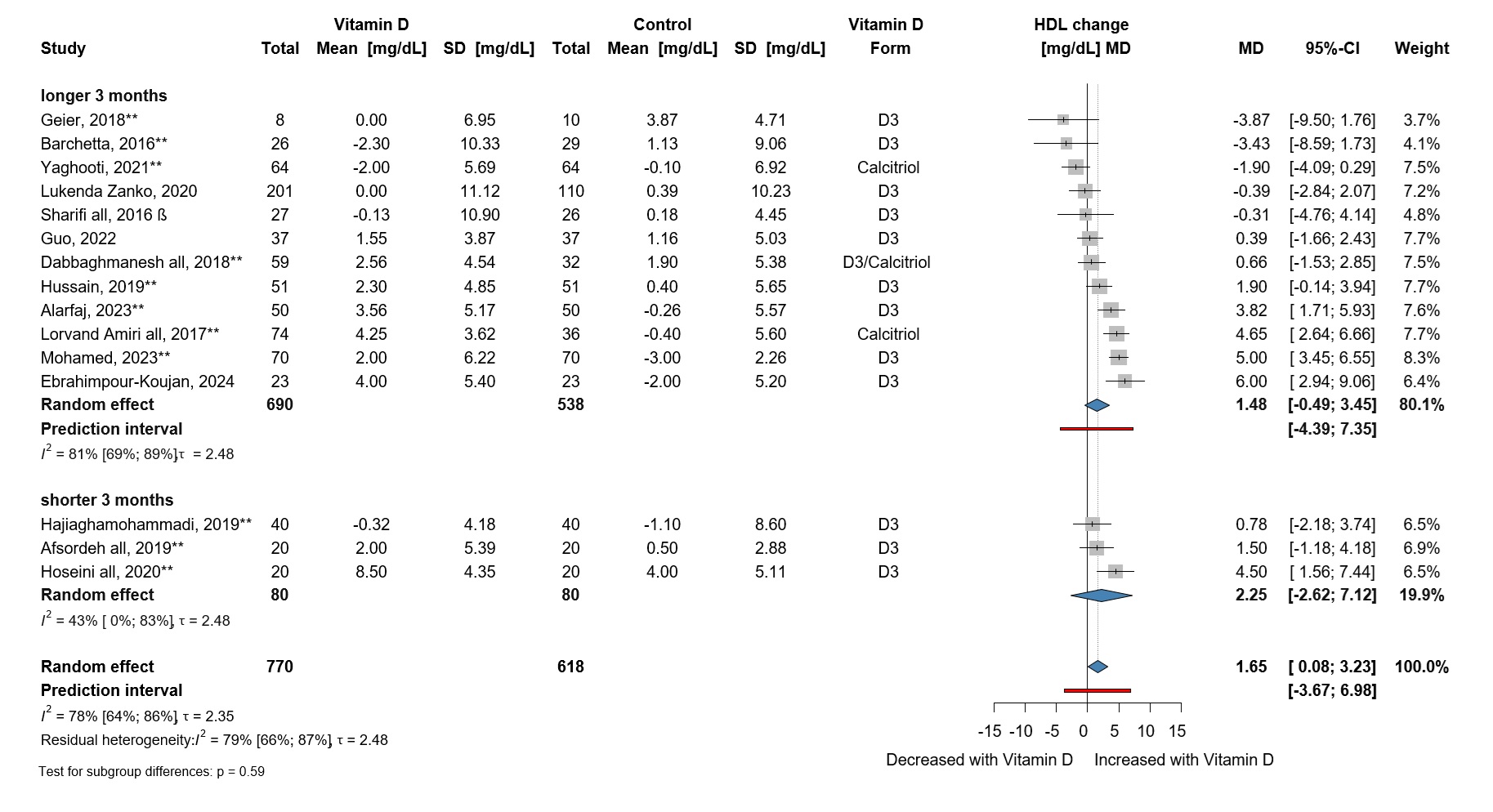


*Figure S4.12. Forest plot showing HDL change in vitamin D and control groups by length of intervention. CI: confidence interval; HDL: high density cholesterol; MD: mean difference; SD: standard deviation. If the study is indicated with **, then the change value is an estimated change value in that study. The β means that the mean and SD are estimated mean and SD in that study. See raw data and synthesis methods.*


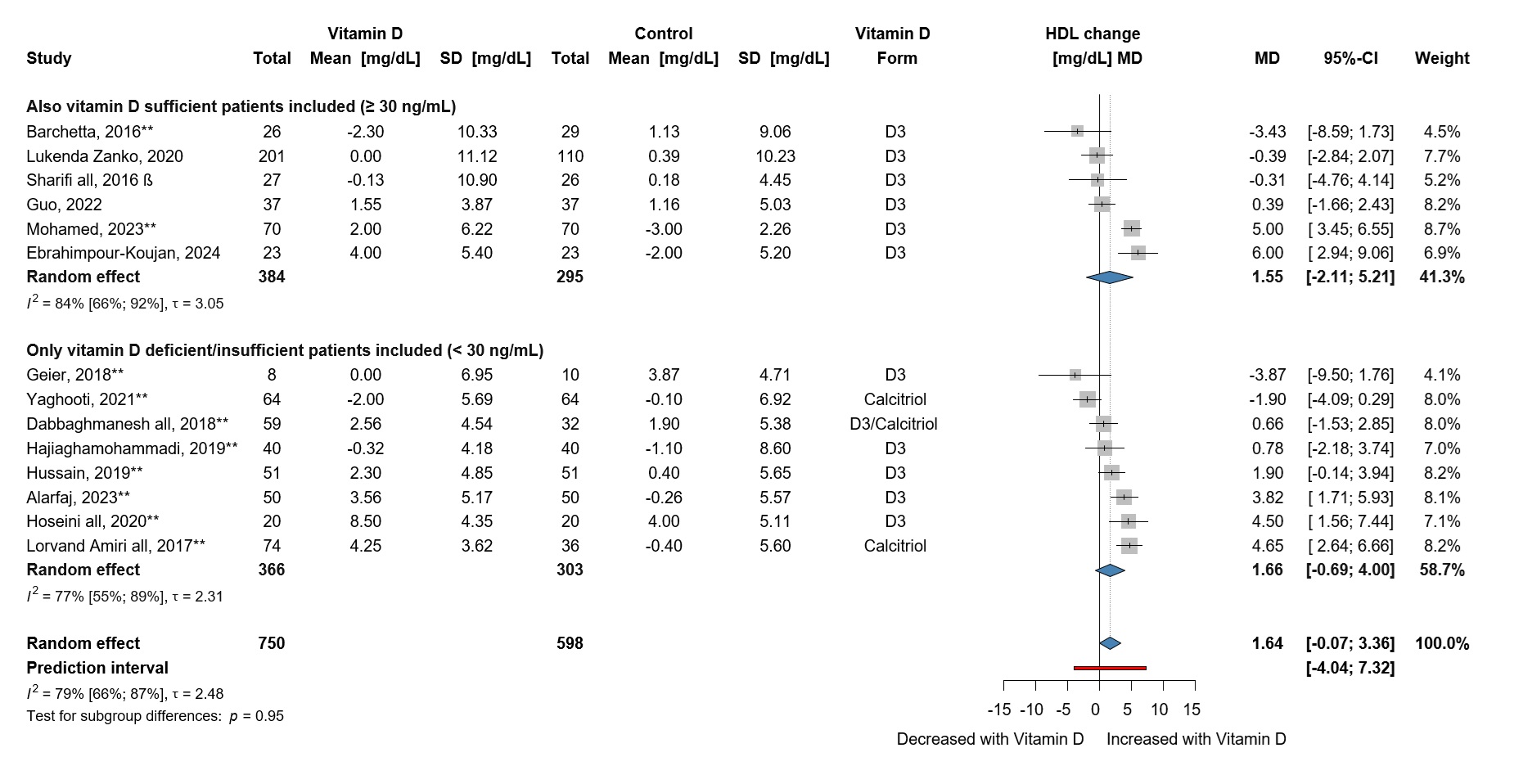


*Figure S4.13. Forest plot showing HDL change in vitamin D and control groups divided into vitamin D deficient/insufficient (< 30 ng/mL) and sufficient (≥ 30 ng/mL) studies. CI: confidence interval; HDL: high density cholesterol; MD: mean difference; SD: standard deviation. If the study is indicated with **, then the change value is an estimated change value in that study. The β means that the mean and SD are estimated mean and SD in that study. See raw data and synthesis methods.*


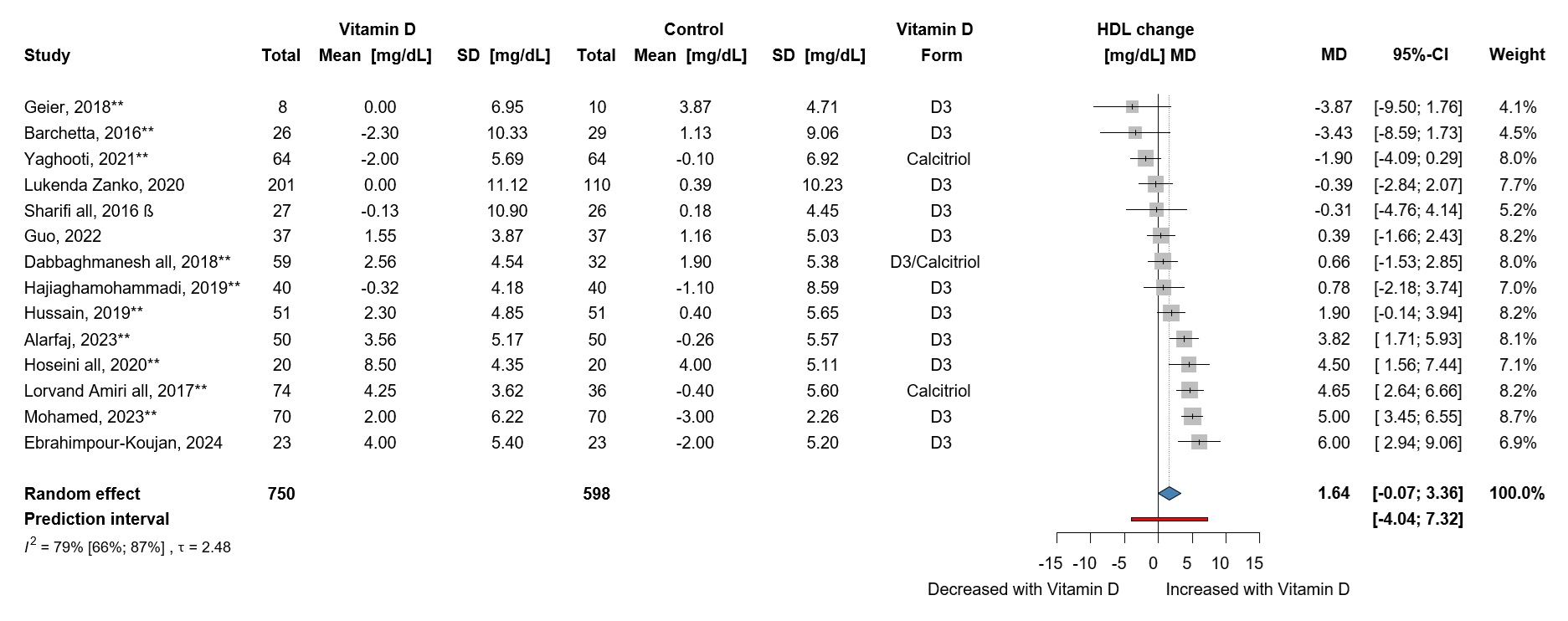


*Figure S4.14. Forest plot showing HDL change in vitamin D and control groups excluding high-risk biased studies. CI: confidence interval; HDL: high density cholesterol; MD: mean difference; SD: standard deviation. If the study is indicated with **, then the change value is an estimated change value in that study. The β means that the mean and SD are estimated mean and SD in that study. See raw data and synthesis methods.*


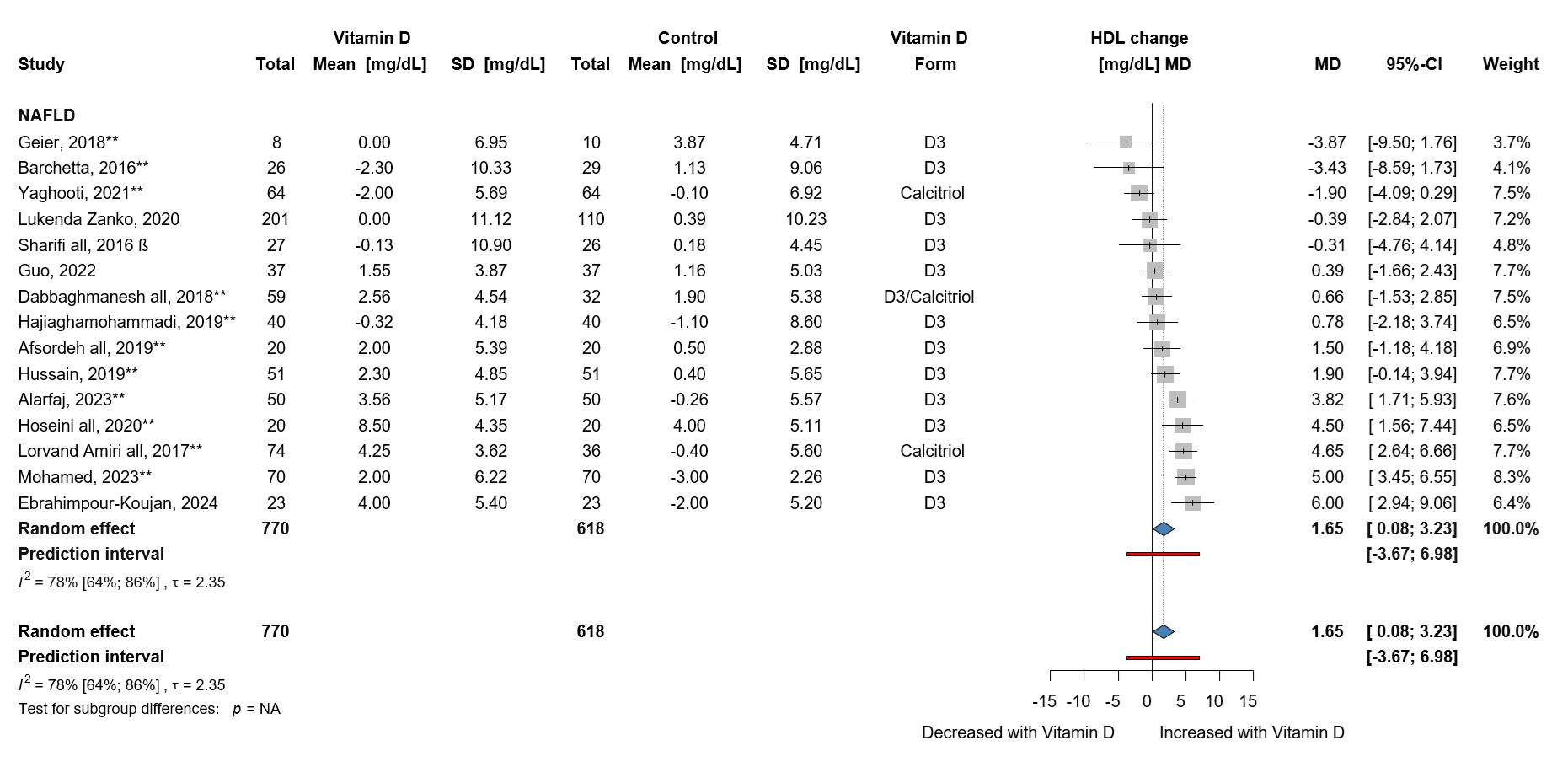


*Figure S4.15. Forest plot showing HDL change in vitamin D and control groups by type of chronic liver disease. CI: confidence interval; HDL: high density cholesterol; MD: mean difference; SD: standard deviation. If the study is indicated with **, then the change value is an estimated change value in that study. The β means that the mean and SD are estimated mean and SD in that study. See raw data and synthesis methods.*

***Triglycerides (TG)***


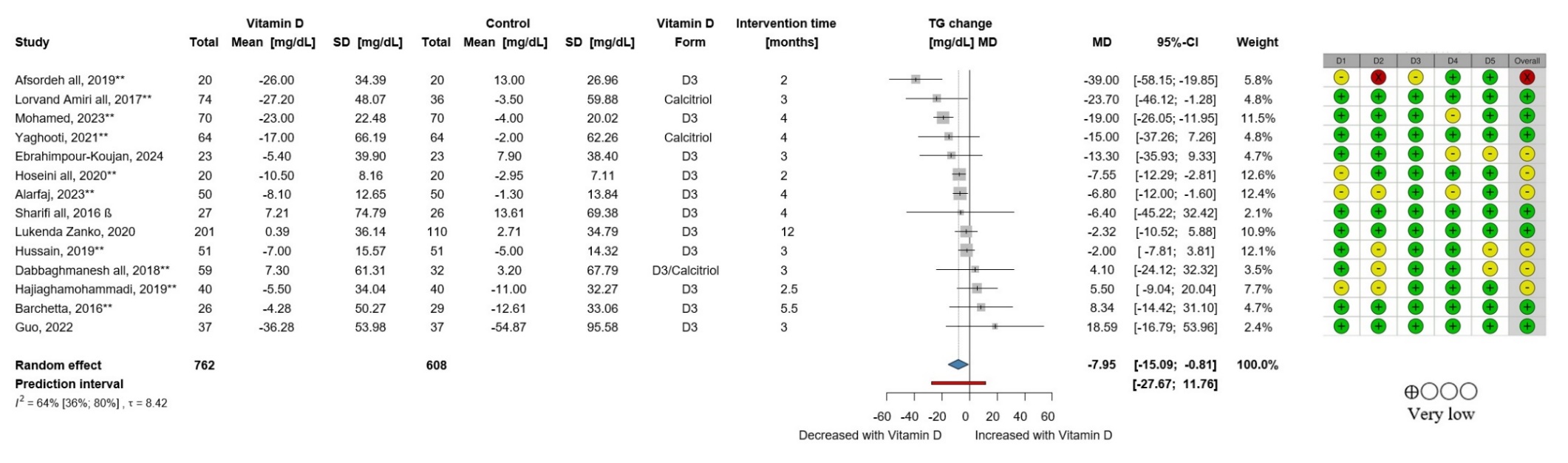


*Figure S4.16a. Forest plot showing total triglycerides change in vitamin D and control groups. CI: confidence interval; MD: mean difference; SD: standard deviation; TG: total triglycerides. If the study is indicated with **, then the change value is an estimated change value in that study. The β means that the mean and SD are estimated mean and SD in that study. See raw data and synthesis methods.*

*Figure S4.16b. Funnel plot for total triglycerides (p = 0.9023).*

*Figure S4.16c. Forest plot with leave-one-out analysis for total triglycerides.*

*Figure S4.16d. Baujat plot for total triglycerides.*


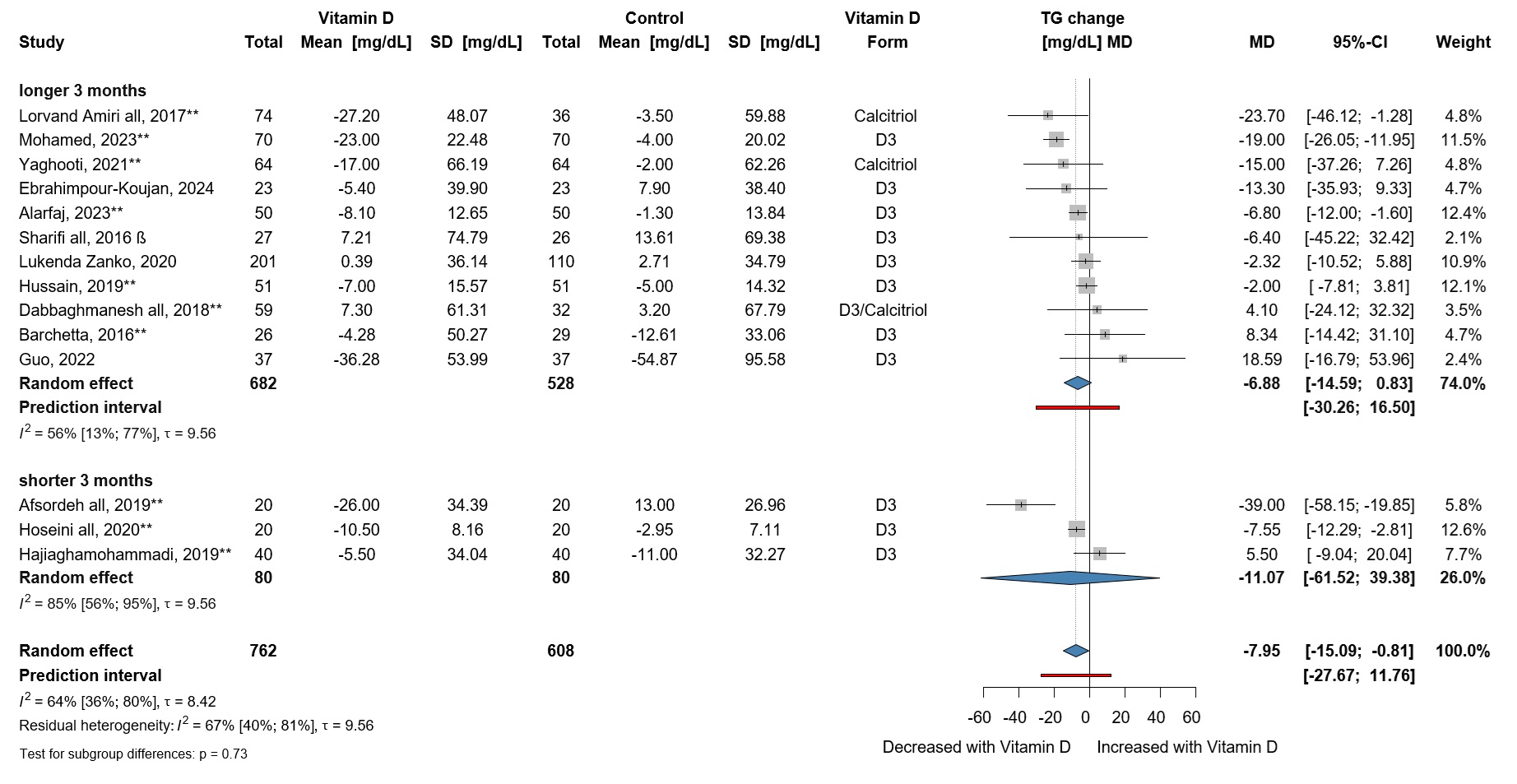


*Figure S4.17. Forest plot showing total triglycerides change in vitamin D and control groups by length of intervention. CI: confidence interval; MD: mean difference; SD: standard deviation; TG: total triglycerides. If the study is indicated with **, then the change value is an estimated change value in that study. The β means that the mean and SD are estimated mean and SD in that study. See raw data and synthesis methods.*


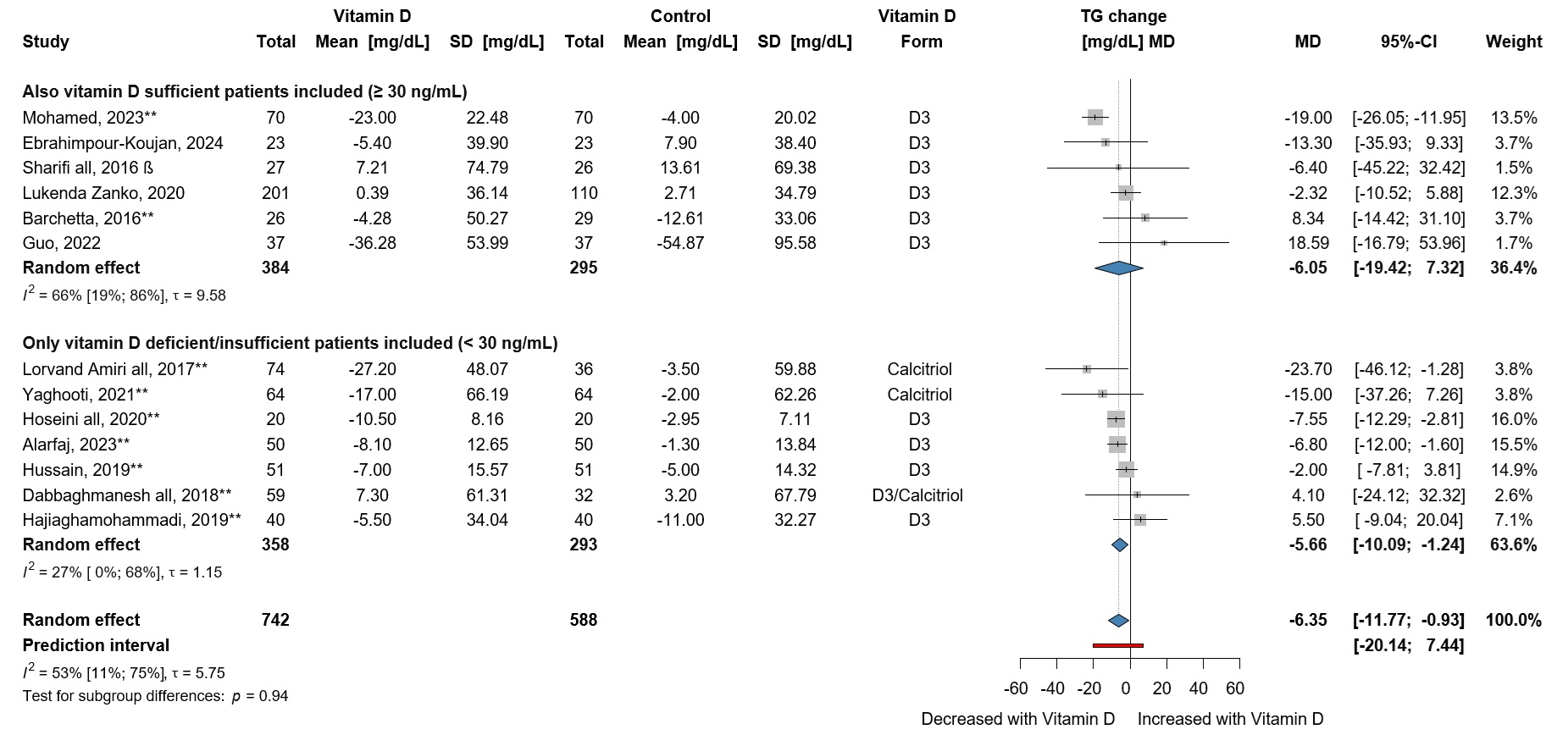


*Figure S4.18. Forest plot showing total triglycerides change in vitamin D and control groups divided into vitamin D deficient/insufficient (< 30 ng/mL) and sufficient (≥ 30 ng/mL) studies. CI: confidence interval; MD: mean difference; SD: standard deviation; TG: total triglycerides. If the study is indicated with **, then the change value is an estimated change value in that study. The β means that the mean and SD are estimated mean and SD in that study. See raw data and synthesis methods.*


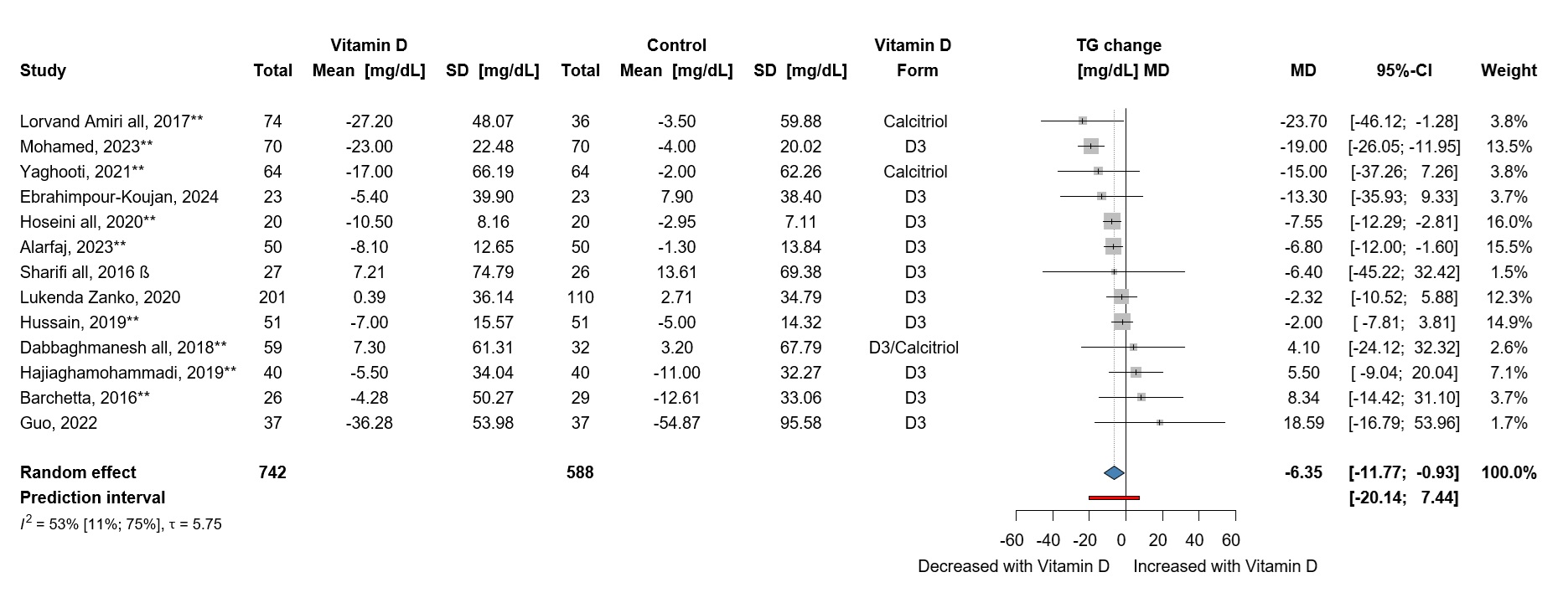


*Figure S4.19. Forest plot showing total triglycerides change in vitamin D and control groups excluding high-risk biased studies. CI: confidence interval; MD: mean difference; SD: standard deviation; TG: total triglycerides. If the study is indicated with **, then the change value is an estimated change value in that study. The β means that the mean and SD are estimated mean and SD in that study. See raw data and synthesis methods.*


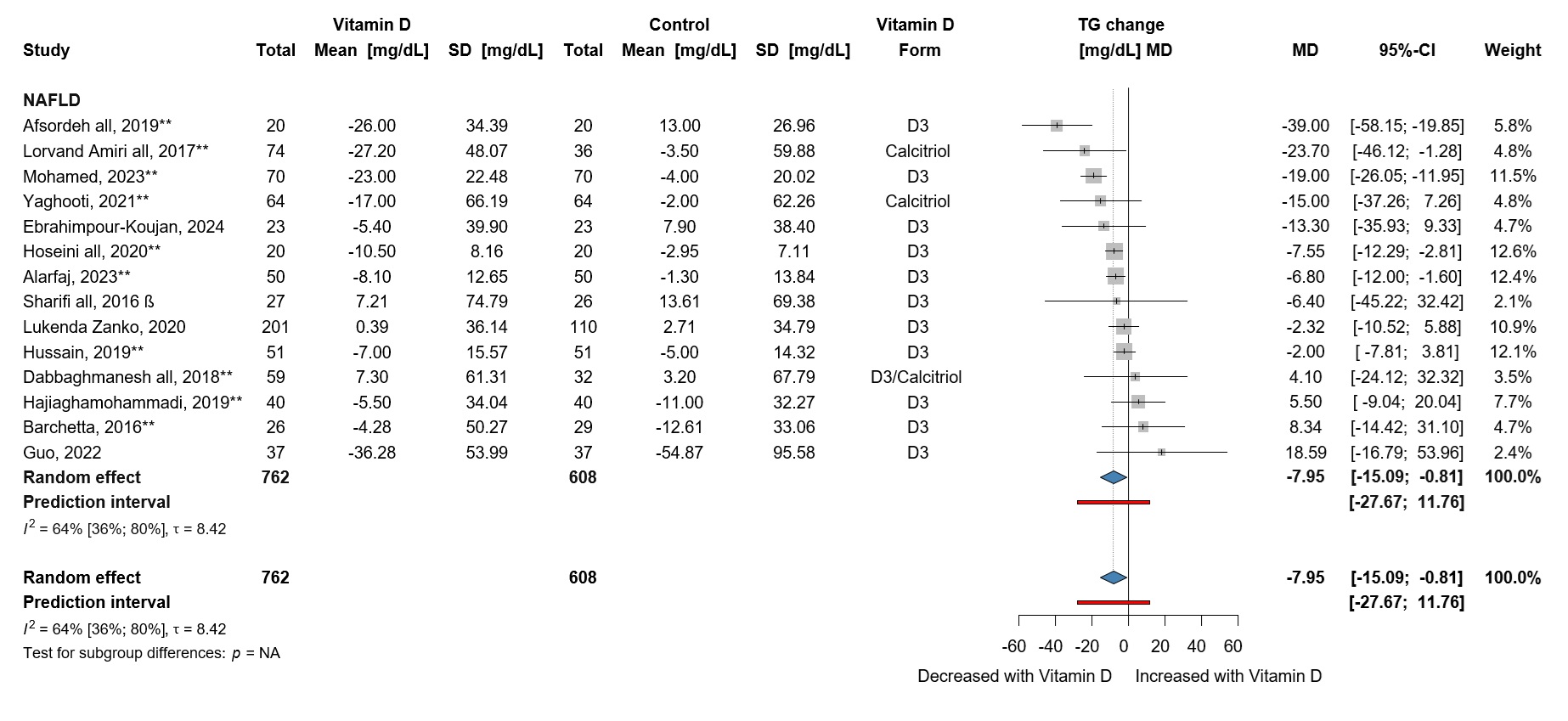


*Figure S4.20. Forest plot showing total triglycerides change in vitamin D and control groups by type of chronic liver disease. CI: confidence interval; MD: mean difference; SD: standard deviation; TG: total triglycerides. If the study is indicated with **, then the change value is an estimated change value in that study. The β means that the mean and SD are estimated mean and SD in that study. See raw data and synthesis methods.*


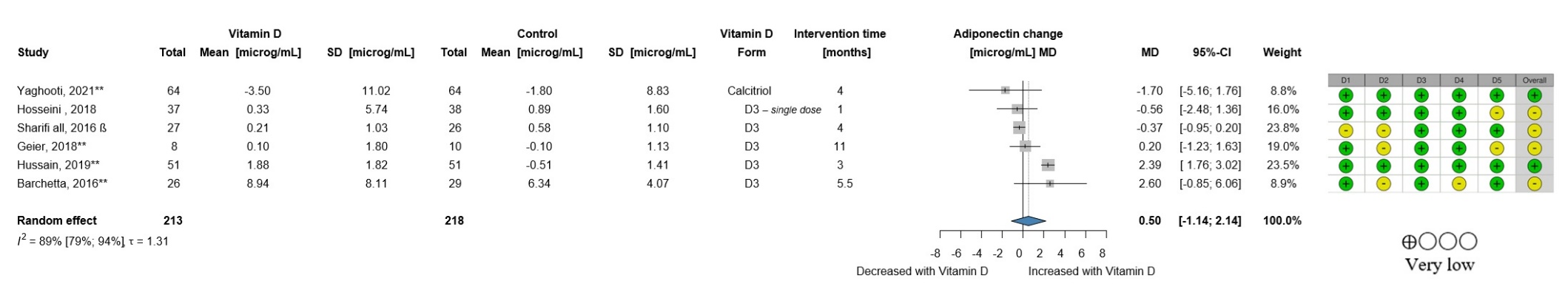


*Figure S4.21a. Forest plot showing adiponectin change in vitamin D and control groups. CI: confidence interval; MD: mean difference; SD: standard deviation. If the study is indicated with **, then the change value is an estimated change value in that study. The β means that the mean and SD are estimated mean and SD in that study. See raw data and synthesis methods.*

*Figure S4.21b. Funnel plot for adiponectin (p = 0.8074). Not enough articles (> 10) for appropriate analysis of publication bias.*

*Not run.*

*Figure S4.21c. Forest plot with leave-one-out analysis for adiponectin.*

*Figure S4.21d. Baujat plot for adiponectin.*


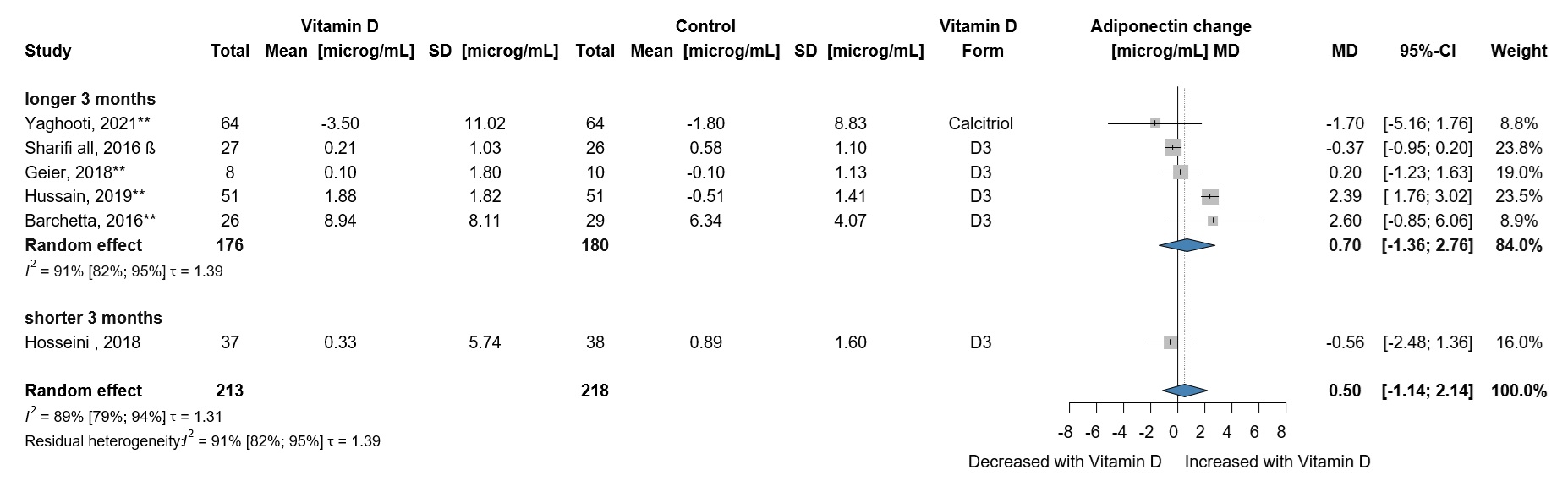


*Figure S4.22. Forest plot showing adiponectin change in vitamin D and control groups by length of intervention. CI: confidence interval; MD: mean difference; SD: standard deviation. If the study is indicated with **, then the change value is an estimated change value in that study. The β means that the mean and SD are estimated mean and SD in that study. See raw data and synthesis methods.*


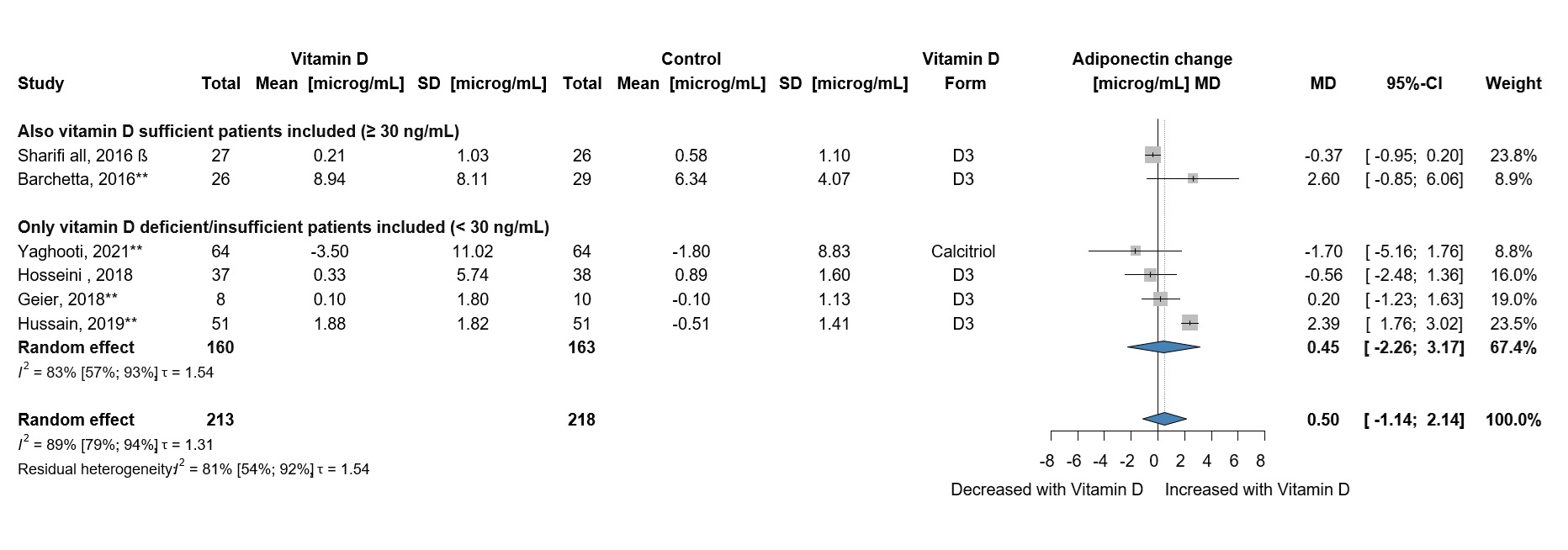


*Figure S4.23. Forest plot showing adiponectin change in vitamin D and control groups divided into vitamin D deficient/insufficient (< 30 ng/mL) and sufficient (≥ 30 ng/mL) studies. CI: confidence interval; MD: mean difference; SD: standard deviation. If the study is indicated with **, then the change value is an estimated change value in that study. The β means that the mean and SD are estimated mean and SD in that study. See raw data and synthesis methods.*


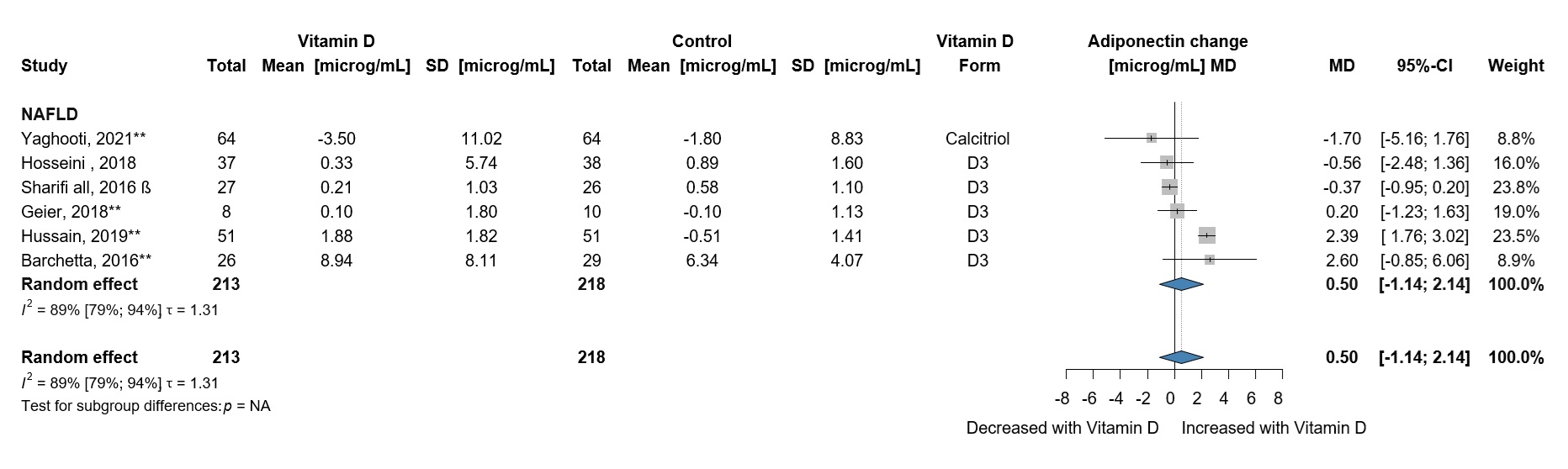


*Figure S4.24. Forest plot showing adiponectin change in vitamin D and control groups by type of chronic liver disease. CI: confidence interval; MD: mean difference; SD: standard deviation. If the study is indicated with **, then the change value is an estimated change value in that study. The β means that the mean and SD are estimated mean and SD in that study. See raw data and synthesis methods.*
